# Proteomics/phosphoproteomics of left ventricular biopsies from patients with surgical coronary revascularization and pigs with coronary occlusion/reperfusion: remote ischemic preconditioning

**DOI:** 10.1038/s41598-017-07883-5

**Published:** 2017-08-09

**Authors:** Nilgün Gedik, Marcus Krüger, Matthias Thielmann, Eva Kottenberg, Andreas Skyschally, Ulrich H. Frey, Elke Cario, Jürgen Peters, Heinz Jakob, Gerd Heusch, Petra Kleinbongard

**Affiliations:** 1 Institute for Pathophysiology, West German Heart and Vascular Center Essen, Universitätsklinikum Essen, Universität Duisburg-Essen, Essen, Germany; 2 Institute for Genetics Cologne Excellence Cluster on Cellular Stress Responses in Aging-Associated Diseases (CECAD), and University of Cologne, Cologne, Germany; 3 Department of Thoracic and Cardiovascular Surgery, West German Heart and Vascular Center Essen, Universitätsklinikum Essen, Universität Duisburg- Essen, Essen, Germany; 4 Klinik für Anästhesiologie und Intensivmedizin, Universitätsklinikum Essen, Universität Duisburg-Essen, Essen, Germany; 5 Experimental Gastroenterology, Department of Gastroenterology and Hepatology, Universitätsklinikum Essen, Universität Duisburg-Essen, Essen, Germany

## Abstract

Remote ischemic preconditioning (RIPC) by repeated brief cycles of limb ischemia/reperfusion reduces myocardial ischemia/reperfusion injury. In left ventricular (LV) biopsies from patients undergoing coronary artery bypass grafting (CABG), only the activation of signal transducer and activator of transcription 5 was associated with RIPC’s cardioprotection. We have now used an unbiased, non-hypothesis-driven proteomics and phosphoproteomics approach to analyze LV biopsies from patients undergoing CABG and from pigs undergoing coronary occlusion/reperfusion without (sham) and with RIPC. False discovery rate-based statistics identified a higher prostaglandin reductase 2 expression at early reperfusion with RIPC than with sham in patients. In pigs, the phosphorylation of 116 proteins was different between baseline and early reperfusion with RIPC and/or with sham. The identified proteins were not identical for patients and pigs, but in-silico pathway analysis of proteins with ≥2-fold higher expression/phosphorylation at early reperfusion with RIPC in comparison to sham revealed a relation to mitochondria and cytoskeleton in both species. Apart from limitations of the proteomics analysis per se, the small cohorts, the sampling/sample processing and the number of uncharacterized/unverifiable porcine proteins may have contributed to this largely unsatisfactory result.

## Introduction

Remote ischemic conditioning by brief episodes of ischemia/reperfusion in parenchymal organs or limbs before (pre-) or during (per-) sustained myocardial ischemia and subsequent reperfusion protects the myocardium from ischemia/reperfusion injury. The protection by remote ischemic conditioning has been confirmed in all species tested so far, including humans^1–4^. The reduction of myocardial ischemia/reperfusion injury by remote ischemic conditioning has been demonstrated in patients undergoing elective interventional^5^ or surgical coronary revascularization^6–9^, and in patients with acute myocardial infarction^10–15^. The protection was confirmed by a reduction in cardiac biomarker release^5–9, 14^ or by cardiac imaging^10, 12, 13, 15^, and it was associated with improved short-^8, 12^ and long-term clinical outcome^5, 7, 11^. In contrast, two recent large-scaled randomized trials in patients undergoing cardiac surgery, ERICCA and RIPHeart, failed to confirm reduced biomarker release and improved clinical outcome with remote ischemic preconditioning (RIPC)^16, 17^. The use of propofol anesthesia in the majority of patients in both trials may have abrogated the cardioprotective effect of RIPC^18, 19^. The magnitude of myocardial ischemia/reperfusion injury may also affect the extent of RIPC’s protection. In patients undergoing coronary artery bypass grafting (CABG), greater myocardial injury by longer cross-clamp time facilitated the manifestation of cardioprotection by RIPC^20^. Along this line, in patients undergoing transfemoral transcatheter aortic valve implantation with only short duration of peri-interventional ischemia and with less troponin I release, RIPC did not provide protection^21^.

In order to fully recruit the cardioprotection by RIPC and to improve patient outcome it is necessary to understand the signal transduction of RIPC. The signaling pathways recruited by remote and local ischemic conditioning maneuvers appear to be similar^22^. Conceptually, the signal transduction comprises triggers which activate intracellular mediator cascades to ultimately transmit the cardioprotective signal to end-effectors, notably the mitochondria^23^. Numerous studies using Western blot analysis, pharmacological agonist and antagonist approaches, and genetic approaches in various experimental models identified a number of signaling proteins, which were conceptually summarized as the nitric oxide synthase/protein kinase G pathway, the reperfusion injury salvage kinase pathway, and the survival activating factor enhancement pathway^24, 25^. The activation and expression of 22 signaling proteins that had previously been identified in experimental models in response to ischemic conditioning maneuvers were analyzed using Western blot analysis in left ventricular (LV) biopsies taken at early reperfusion after cardioplegic ischemic arrest from patients undergoing CABG under isoflurane anesthesia^26, 27^. Among these 22 proteins, only the activation of the signal transducer and activator of transcription 5 (STAT5) was associated with reduced biomarker release by RIPC^26^. In pigs, STAT3 but not STAT5 activation is causally involved in cardioprotection by RIPC^28^, reflecting species-specific differences in the signal transduction of RIPC^24^. The up- and downstream signals of STAT5/STAT3 as well as other, not previously described pathways in signal transduction of RIPC have not been identified yet. An unbiased, non-hypothesis-driven analysis of myocardial tissue may therefore provide new insights into the signal transduction and identify novel therapeutic targets of RIPC^29–32^. We have therefore now analyzed and compared the proteome and phosphoproteome of LV biopsies taken at early reperfusion after cardioplegic ischemic arrest from patients undergoing CABG without (sham) and with RIPC. Whereas proteome analysis at early reperfusion most likely reflects RIPC-related differences in proteolysis by ischemia rather than protein biosynthesis, the phosphoproteome analysis was aimed to identify the potential activation of cardioprotective proteins by RIPC. Indeed, the majority of cardioprotective proteins are regulated by posttranslational modifications, mainly by phosphorylation^33^. For comparison, we used the translational pig model with coronary occlusion/reperfusion without (sham) or with RIPC^28^, to analyze the proteome and phosphoproteome of LV biopsies taken at baseline before coronary occlusion/reperfusion and at early reperfusion. This animal model has less interindividual variability than that of patients and no co-morbidities and co-medications^34^.

## Results

### Cardioprotection by RIPC

**Figure 1:**
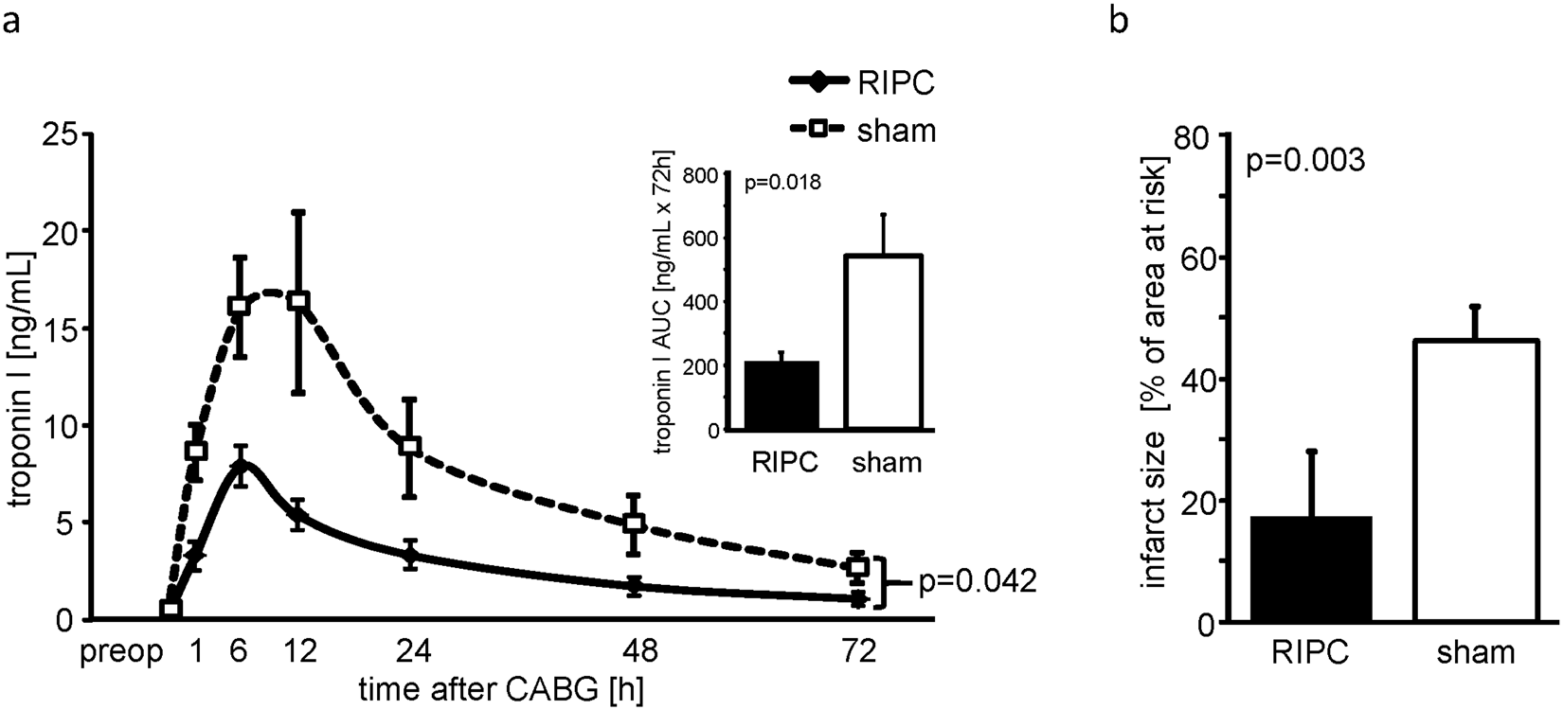
Serum concentration of troponin I of patients. (**a**) The serum concentration of troponin I before (preop) and over 72 h after coronary artery bypass grafting (CABG) in patients undergoing remote ischemic preconditioning (RIPC; black symbols/bars) or sham (white symbols/bars). Decreased troponin I concentrations confirmed protection by RIPC. Insert: area under the curve (AUC) for serum troponin I concentrations over 72 h. (**b**) Infarct size of pigs after 60 min coronary occlusion and 180 min reperfusion. Protection by RIPC was confirmed by infarct size reduction. Data are mean ± standard error of the mean. The data were compared using a 2-way (group, time) ANOVA for repeated measures or using unpaired Student’s t-test (AUC, infarct size).

In patients, demographics, baseline and intraoperative characteristics were not different between those with RIPC and with sham (Table 1). The preoperative serum troponin I concentration (cTnI) did not differ between patients with RIPC and with sham. The cTnI release was decreased by RIPC (area under the curve (AUC) over 72 h: 218 ± 28 versus 569 ± 134 ng/ml × 72 h, p = 0.018; Fig. 1a). In pigs, the area at risk (24 ± 2 versus 23 ± 2% of the LV) was not different between RIPC (24 ± 2% of the LV) and sham (23 ± 2%). Transmural blood flow within the area at risk was reduced at 5 min ischemia from baseline (RIPC: 0.023 ± 0.004 versus 0.780 ± 0.027 ml/min/g; sham: 0.023 ± 0.004 versus 0.718 ± 0.035 ml/min/g). RIPC reduced infarct size (Fig. 1b).

**Table 1:** Baseline and intraoperative characteristics of patients.

	RIPC (n = 11)	sham (n = 11)	p-value
**demographics**			
age (years)	64.5 ± 2.6	65.5 ± 3.0	0.802
sex (male)	10	10	1.000
body weight (kg)	79.5 ± 5.2	89.9 ± 5.1	0.169
**risk factors and co-morbidities**			
diabetes mellitus	0	1	0.500
hypertension	7	10	0.311
hyperlipidemia	3	7	0.114
peripheral vessel disease	1	1	1.000
COPD	1	1	1.000
renal disease (creatinine > 200 μmol/l)	3	1	0.586
**cardiac status**			
angina CCS III–IV	5	4	1.000
previous myocardial infarction	2	3	1.000
left ventricular ejection fraction (%)	47.6 ± 2.7	47.7 ± 2.2	0.959
**medication**			
aspirin	11	9	0.477
clopidogrel	3	5	0.421
β blockers	7	11	0.090
statins	7	11	0.090
ACE inhibitors or ARBs	7	9	0.635
**risk scores**			
additive EuroSCORE	4.2 ± 0.5	4.6 ± 0.4	0.517
logistic EuroSCORE (%)	3.4 ± 0.7	3.7 ± 0.5	0.660
EuroSCORE II (%)	1.0 ± 0.1	1.1 ± 0.1	0.566
**intraoperative characteristics**			
time from end of RIPC/sham to reperfusion (min)	141.9 ± 14.1	129.2 ± 5.7	0.413
aortic cross-clamp duration (min)	60.5 ± 6.5	73.5 ± 6.4	0.163
cardioplegia (ml)	1512 ± 69	1600 ± 65	0.373
reperfusion time (min)	30.9 ± 3.7	38.5 ± 4.8	0.230
number of bypass grafts	2.6 ± 0.3	3.4 ± 0.3	0.080
number of distal anastomoses	2.7 ± 0.2	3.3 ± 0.3	0.091
transit time graft flow (ml/min)	76.3 ± 11.0	81.5 ± 10.2	0.734

Data are mean ± standard error of the mean or number. Patient baseline and intraoperative characteristics were compared using unpaired Student’s t-test (continuous data) and 2-tailed Fisher’s exact test (categorical data). Chronic obstructive pulmonary disease (COPD), Canadian cardiovascular society score (CCS), angiotensin-converting enzyme (ACE), angiotensin-II-receptor blockers (ARBs), European system for cardiac operative risk evaluation (EuroSCORE), remote ischemic preconditioning (RIPC). Reperfusion time: time from release of aortic cross-clamp to end of cardiopulmonary bypass.

### Comparable sample composition in the individual LV biopsies

In human LV biopsies, the expressions of proteins characterizing the different cell types, i.e. CD31, collagen 3α, hemoglobin γ, enolase 2 and tropomyosin, were comparable between RIPC and sham when lysed with Tris/sodium dodecyl sulfate (SDS) and radioimmunoprecipitation assay (RIPA) buffer, respectively (Supplemental Figs S1a and S2a and b). In porcine LV biopsies, the expressions of these proteins were also not different between baseline and early reperfusion and between RIPC and sham, respectively (Supplemental Figs S1b and S3).

### Western blot analysis of STAT5 in human and STAT3 in porcine LV biopsies

**Figure 2:**
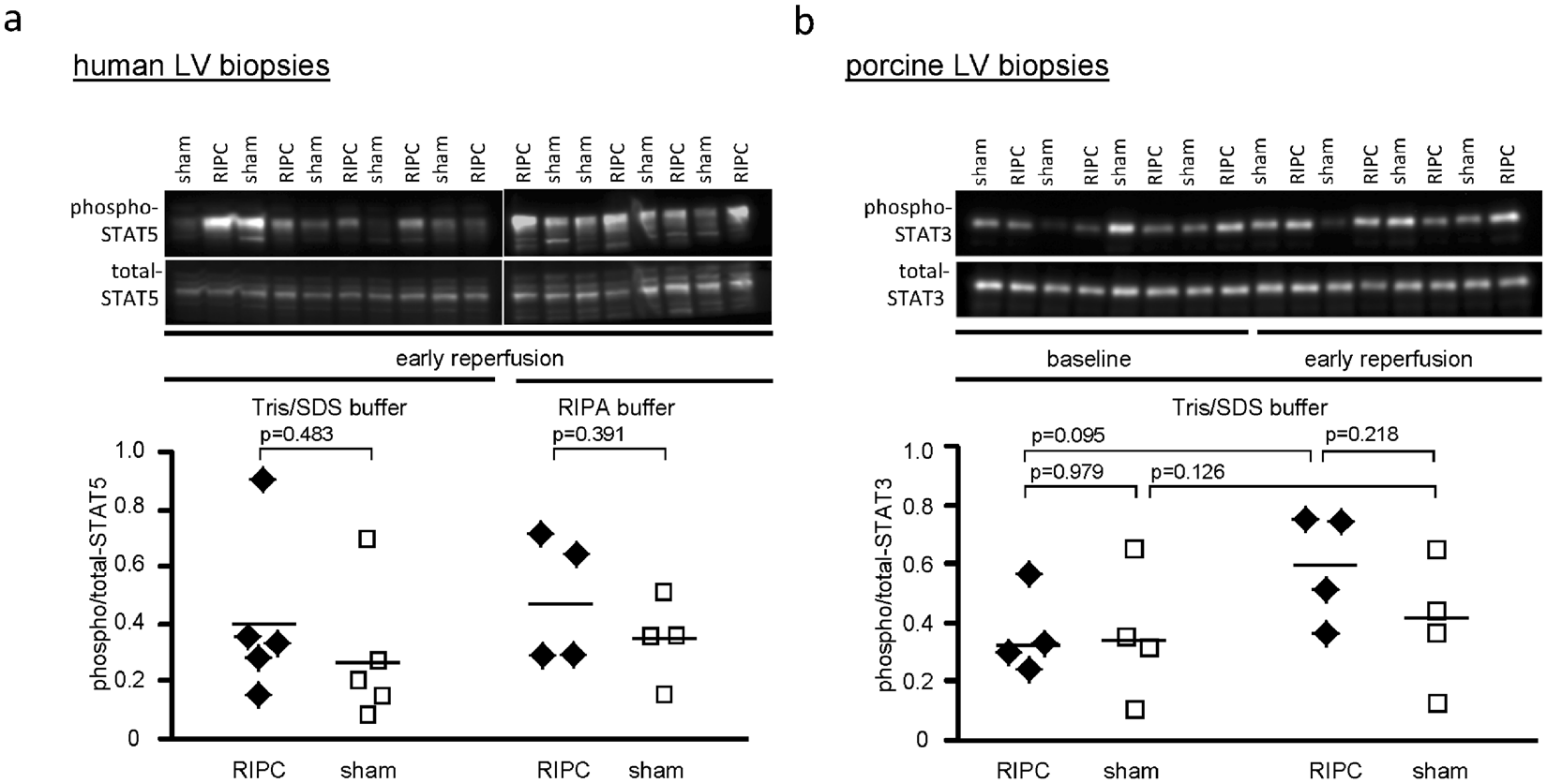
Western blot analysis of phosphorylation/expression of STAT5 in human and of STAT3 in porcine left ventricular biopsies. (**a**) The phosphorylation of signal transducer and activator of transcription 5 (STAT5) at tyr_694_ tended to be increased at early reperfusion with remote ischemic preconditioning (RIPC; black symbols) compared to that with sham (white symbols) in human left ventricular (LV) biopsies lysed in Tris/sodium dodecyl sulfate (SDS) or radioimmunoprecipitation assay (RIPA) buffer. (**b**) The phosphorylation of signal transducer and activator of transcription 3 (STAT3) at tyr_705_ tended to be increased from baseline to early reperfusion after coronary occlusion with RIPC, and tended to be greater with RIPC than with sham in porcine LV biopsies. Immunoreactivities of the phosphorylated proteins were normalized to the respective total forms and compared by unpaired (between RIPC and sham) or by paired (between baseline and early reperfusion in pigs) Student’s t-tests. The blots were cropped to display the relevant bands, full-length blots and Ponceau-S stainings are presented in Supplemental Fig. S4.

Confirming our prior reports^26, 27^, the STAT5 phosphorylation in human LV biopsies taken at early reperfusion tended to be higher with RIPC than with sham when lysed with Tris/SDS buffer and with RIPA buffer (Fig. 2a and Supplemental Fig. S4a). Also confirming our prior report^28^, the STAT3 phosphorylation in porcine LV biopsies tended to be increased from baseline to early reperfusion after coronary occlusion with RIPC, and STAT3 phosphorylation during early reperfusion tended to be higher in pigs with RIPC than with sham (Fig. 2b and Supplemental Fig. S4b).

### Proteome and phosphoproteome analysis of human and porcine LV biopsies

**Figure 3:**
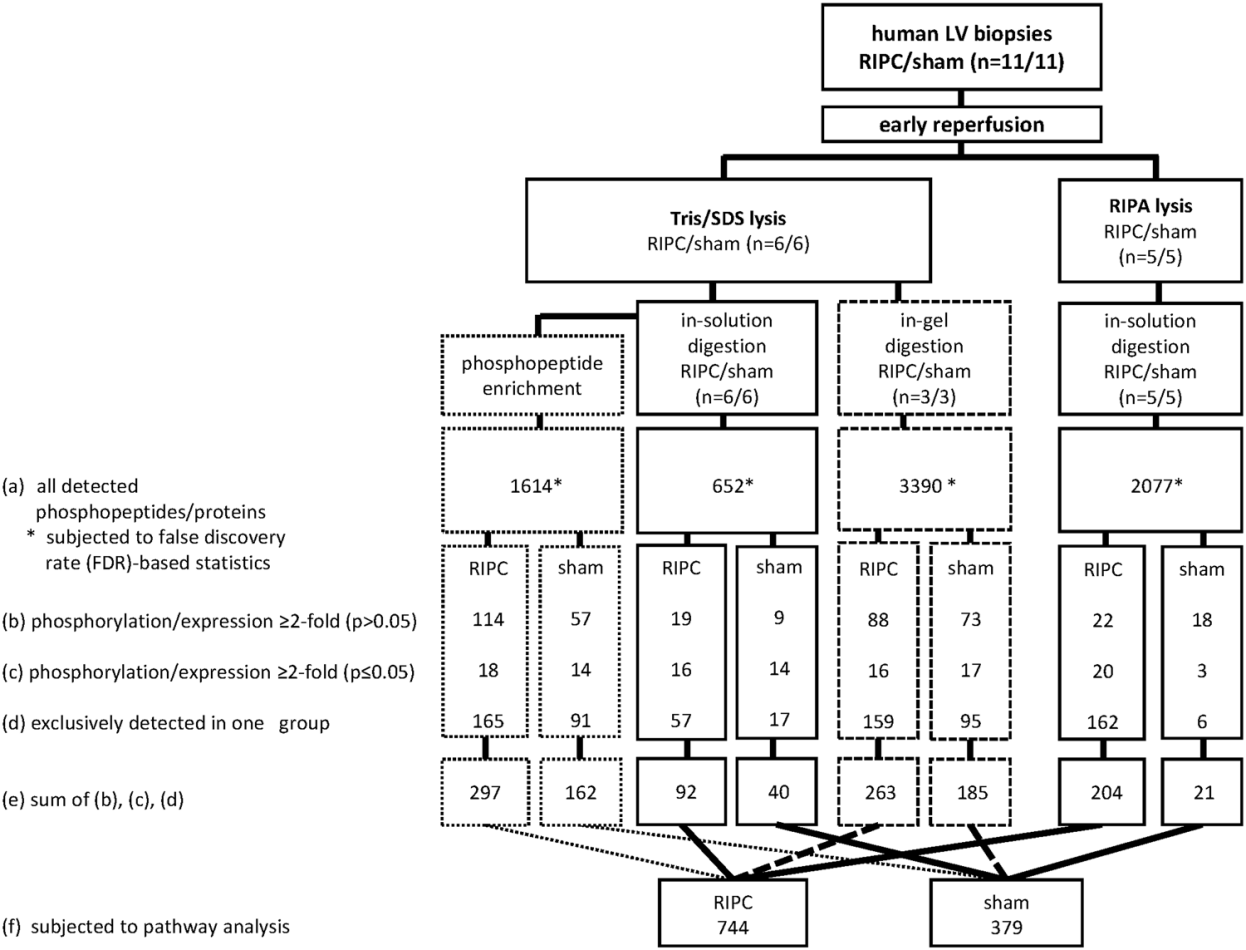
Flow chart of sampling and proteome analysis of human left ventricular biopsies. Human left ventricular (LV) biopsies from patients without (sham) or with remote ischemic preconditioning (RIPC) were lysed in Tris/sodium dodecyl sulfate (Tris/SDS) or in radioimmunoprecipitation assay (RIPA) buffer. Proteome analysis was performed after phosphopeptide enrichment, in-solution digestion, and in-gel digestion with Tris/SDS buffer-lysed biopsies and after in-solution digestion with RIPA buffer-lysed biopsies, respectively. The numbers of all detected phosphopeptides/proteins were displayed in line (a), those with ≥2-fold higher phosphorylation/expression in line (b) and with significant (p < 0.05) ≥2-fold higher phosphorylation/expression with RIPC versus with sham in line (c), as well as those exclusively detected with RIPC or with sham in line (d). The sum of line (b), (c) and (d) was displayed in line (e). All detected phosphopeptides/proteins (line (a)) were subjected to false discovery rate (FDR)-based statistical analysis. Independently of lysis and digestion methods, all proteins detected with a ≥2-fold higher phosphorylation/expression with RIPC versus with sham and those exclusively detected in one group, respectively, were considered (line (f)) for an in-silico pathway analysis.

**Figure 4:**
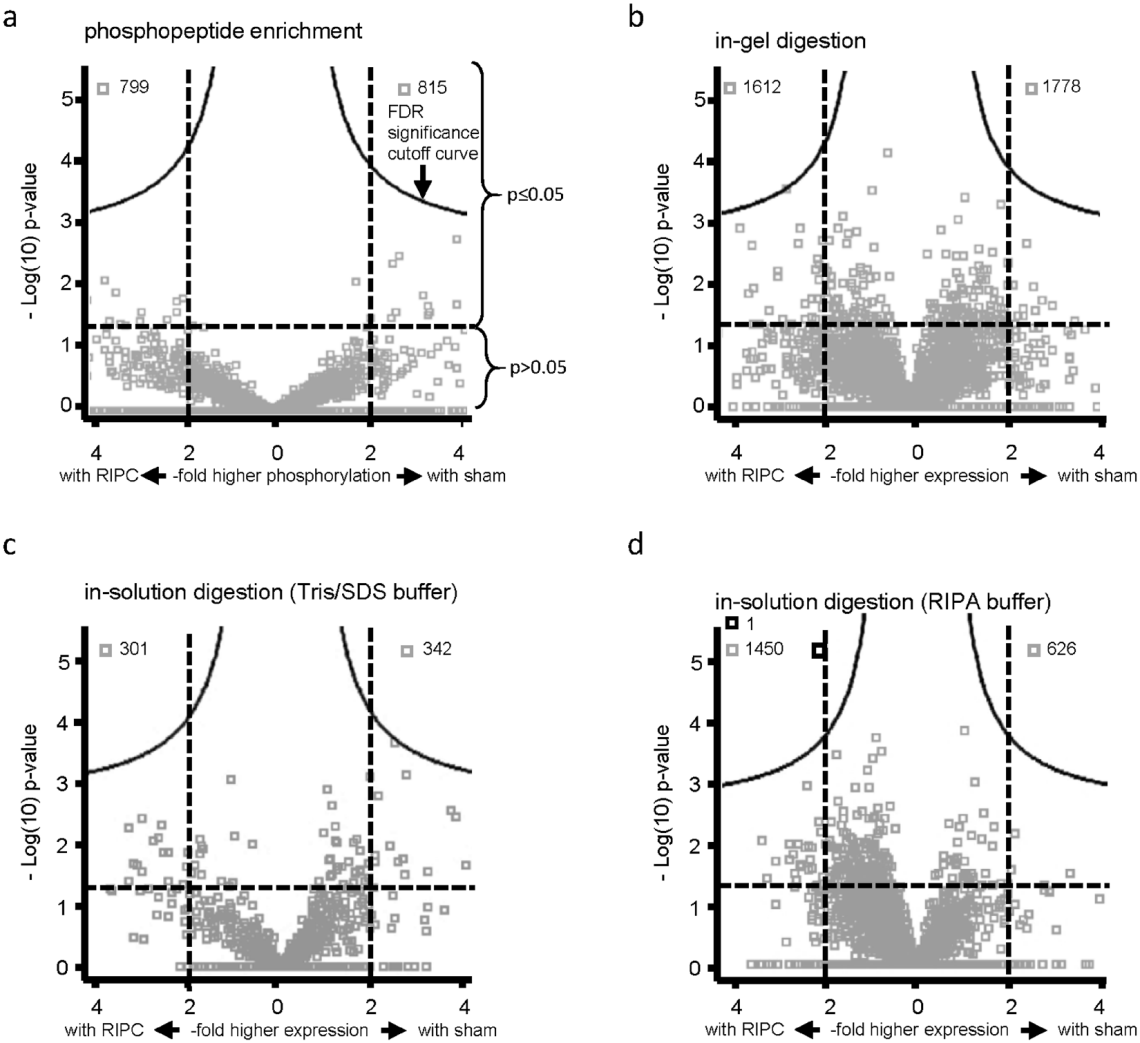
Vulcano plots of all detected proteins in the phosphoproteome/proteome analysis in human left ventricular biopsies. Vulcano plots of −Log(10)p-value over -fold higher phosphorylation/expression between groups (remote ischemic preconditioning (RIPC)/sham) of all proteins detected after (**a**) phosphopeptide enrichment, (**b**) in-gel digestion, (**c**) in-solution digestion of Tris/sodium dodecyl sulfate (Tris/SDS) and (**d**) radioimmunoprecipitation assay (RIPA) buffer-lysed human left ventricular biopsies taken at early reperfusion. A −Log(10)p-value of ≥1.3 corresponds to a p-value of ≤0.05. The false discovery rate (FDR) significance cut-off curve indicates only a significant increase of prostaglandin reductase 2 with RIPC than with sham in human biopsies lysed in RIPA buffer. Grey squares: phosphopeptides/proteins without FDR-based statistical difference between RIPC and sham. Black square: protein with FDR-based statistical difference between RIPC and sham.

The proteome analysis of human LV biopsies taken at early reperfusion and lysed in Tris/SDS buffer detected 652 proteins after in-solution digestion and 1614 phosphorylation sites after phosphopeptide enrichment. Among these phosphopeptides, 1470 phosphorylation sites were associated with 391 proteins. The proteome analysis detected 3390 proteins after in-gel digestion and 2077 proteins after RIPA lysis and in-solution digestion (Fig. 3, line (a)). The false discovery rate (FDR)-based statistical analysis of all detected proteins/phosphopeptides in human LV biopsies did not identify a different protein expression/activation pattern with RIPC versus sham after phosphopeptide enrichment, in-gel digestion and in-solution digestion (Tris/SDS buffer), respectively (Fig. 4a–c). However, in RIPA buffer-lysed human LV biopsies taken at early reperfusion, there was a shift towards a greater number of proteins which had higher expression with RIPC than with sham (Fig. 4d). The FDR-based statistical analysis identified prostaglandin reductase 2 at higher expression at early reperfusion with RIPC than with sham.

**Figure 5:**
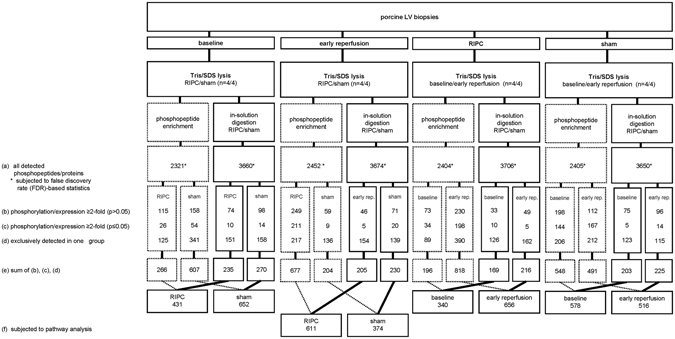
Flow chart of sampling and proteome analysis of porcine left ventricular biopsies. Porcine left ventricular (LV) biopsies from pigs without (sham) or with remote ischemic preconditioning (RIPC) were taken at baseline and at early reperfusion and lysed in Tris/sodium dodecyl sulfate buffer (Tris/SDS). Proteome analysis was performed after phosphopeptide enrichment and in-solution digestion. The numbers of all detected phosphopeptides/proteins were displayed in line (a), those with ≥2-fold higher phosphorylation/expression in line (b), these with significant (p < 0.05) ≥2-fold higher phosphorylation/expression between groups (RIPC/sham) or between time points (baseline/early reperfusion), respectively, in line (c), and those exclusively detected in one group (RIPC/sham) or at one time point (baseline/early reperfusion) in line (d). The sum of lines (b), (c) and (d) was displayed in line (e). All detected phosphopeptides/proteins (line (a)) were subjected to false discovery rate (FDR)-based statistical analysis. All proteins detected at early reperfusion with a ≥2-fold higher phosphorylation/expression with RIPC than with sham (line (f)), were considered for an in-silico pathway analysis.

The proteome analysis of porcine LV biopsies taken at baseline and early reperfusion detected 3660 and 3674 proteins after in-solution digestion and 2321 and 2452 phosphorylation sites after phosphopeptide enrichment, respectively. The comparison of the proteome analysis between RIPC and sham revealed 3706 and 3650 proteins after in-solution digestion and 2404 and 2405 phosphorylation sites after phosphopeptide enrichment (Fig. 5, line (a)), respectively.

**Figure 6:**
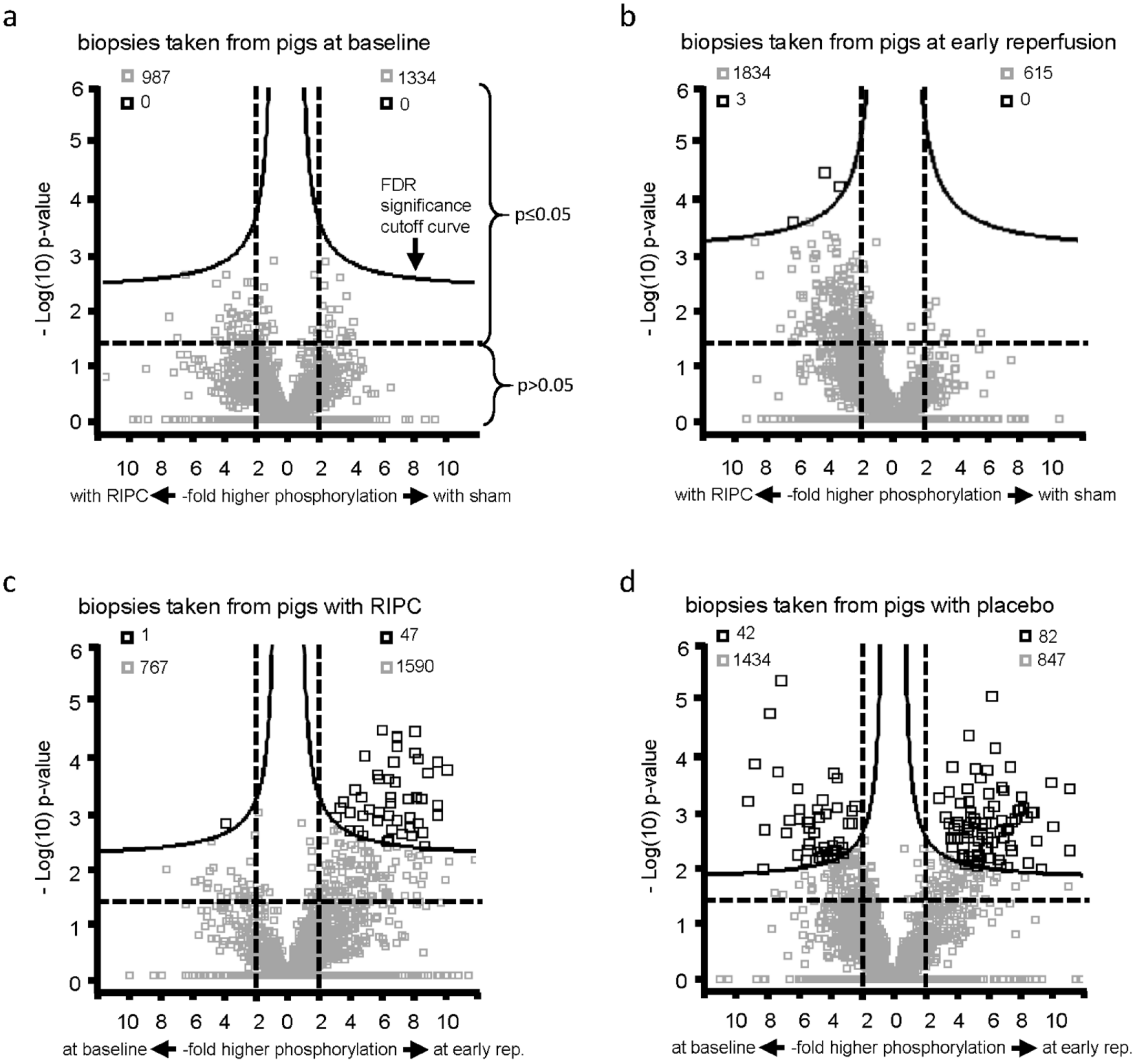
Vulcano plots of all detected phosphopeptides after phosphoproteome phosphopeptide enrichement of porcine left ventricular biopsies. Vulcano plots of −Log(10)p-value over -fold higher phosphorylation of all proteins between groups (remote ischemic preconditioning (RIPC)/sham) detected after phosphopeptide enrichment of porcine left ventricular biopsies taken (**a**) at baseline and (**b**) at early reperfusion. Vulcano plots of −Log(10)p-value over -fold higher phosphorylation of all proteins in the comparison between time points (baseline versus early reperfusion) (**c**) with remote ischemic preconditioning (RIPC) and (**d**) with sham. A −Log(10)p-value of ≥1.3 corresponds to a p-value of ≤0.05. The false discovery rate (FDR) significance cut-off curve indicates differences in protein phosphorylation between RIPC and sham at early reperfusion and between time points (baseline/early reperfusion) with RIPC and with sham, respectively. Grey squares: phosphopeptides without FDR-based statistical difference between RIPC and sham or between baseline and early reperfusion. Black squares: phosphopeptides with FDR-based statistical difference between RIPC and sham or between baseline and early reperfusion.

In porcine LV biopsies, there was no FDR-based statistical difference in the protein expression between baseline and early reperfusion or between RIPC and sham, respectively (Supplemental Fig. S5). There was also no difference in the protein phosphorylation at baseline between RIPC and sham (Fig. 6a and Table 2). The FDR-based statistical analysis revealed a higher phosphorylation of 3 proteins at early reperfusion with RIPC than with sham (Fig. 6b and Table 2). In pigs with RIPC, the comparison between baseline and early reperfusion revealed a decrease in 1 and an increase in phosphorylation of 47 proteins from baseline to early reperfusion (Fig. 6c and Table 2). In pigs with sham, the phosphorylation of 42 proteins was decreased and that of 82 proteins increased from baseline to early reperfusion (Fig. 6d and Table 2). Independent of the FDR-based statistical analysis, there was obviously a shift towards a greater number of proteins with increased phosphorylation at early reperfusion with RIPC than with sham (Fig. 6b and c).

**Table 2:** Phosphoproteins in porcine LV biopsies identified with a difference in FDR-based statistical analysis.

increased/higher phosphorylation	protein name	phosphorylation site	protein ID	-fold higher phosphorylation	p-value
at baseline with RIPC					
	—	—	—	—	—
at baseline with sham					
	—	—	—		—
at early reperfusion with RIPC					
	microtubule-associated protein 1B	1324	P15205	4.28	0.0000
	mitogen-activated protein kinase 3	134	P27361	3.30	0.0001
	myosin-7	415; 1222; 1221	P12883	6.19	0.0003
at early reperfusion with sham					
	—	—	—	—	—
with RIPC at baseline					
	synaptopodin 2-like protein	893	Q9H988	3.94	0.0014
with RIPC at early reperfusion					
	actin-binding LIM protein 1	286	O14639	4.80	0.0001
	adenylyl cyclase-associated protein 1	202	P40123	7.65	0.0017
	ADP/ATP translocase 1	42	P12235	6.36	0.0002
	α-crystallin B chain	59	P02511	6.45	0.0006
	α-crystallin B chain	19	P02512	6.38	0.0030
	ankyrin-2	3732	Q01484	6.52	0.0018
	ankyrin-2	3848	Q01484	5.72	0.0002
	BAG family molecular chaperone regulator 3	387	O95817	3.94	0.0020
	Bcl-2-like protein 13	344	Q9BXK5	8.34	0.0005
	β-taxilin	554	Q8N3L3	5.93	0.0000
	F-actin capping protein beta subunit variant II	263;263	P47756	4.41	0.0020
	galectin 3	25	P17931	7.93	0.0000
	glycogen [starch] synthase	727	P13807	8.12	0.0015
	host cell factor 1	666	P51610	6.89	0.0001
	kinesin-like protein	930	P33176	8.76	0.0002
	LIM and calponin homology domains-containing protein 1	718	Q9UPQ0	3.53	0.0006
	LIM domain-binding protein 3	189; 190; 267	O75112	17.65	0.0009
	mitogen-activated protein kinase 1	90; 152	P28482	4.17	0.0003
	mitogen-activated protein kinase 3	134	P27361	4.60	0.0005
	myosin-7	210; 629	P12883	7.19	0.0023
	PGC-1 and ERR-induced regulator in muscle protein 1	138	Q5SV97	5.17	0.0008
	phosphatidylethanolamine-binding protein 1	185	P30086	9.38	0.0011
	proteasome inhibitor PI31 subunit	152	Q92530	5.59	0.0012
	protein phosphatase 1G	526	O15355	4.75	0.0011
	rho guanine nucleotide exchange factor 2	619	Q92974	6.37	0.0031
	selenocysteine lyase	17	Q96I15	8.13	0.0001
	synaptopodin 2-like protein	722	Q9H987	3.30	0.0008
	synaptopodin-2	250	Q9UMS6	9.41	0.0001
	synemin	1250	O15061	7.67	0.0011
	synemin	1020	O15062	6.75	0.0002
	trafficking kinesin-binding protein 1	377	Q9UPV9	6.28	0.0031
	uncharacterized protein	58	F1RZW0	6.40	0.0005
	uncharacterized protein	66	F1RZW0	6.80	0.0000
	uncharacterized protein	45	F1SC49	6.32	0.0010
	uncharacterized protein	477	F1SE25	4.71	0.0024
	uncharacterized protein	46; 230	F1SMV6	5.58	0.0002
	uncharacterized protein	223	I3L8Q0	7.94	0.0001
	uncharacterized protein	31	I3LAR5	8.06	0.0005
	uncharacterized protein	297	I3LAR5	6.88	0.0000
	uncharacterized protein	422	I3LAR5	5.77	0.0009
	uncharacterized protein	384	I3LI59	6.63	0.0001
	uncharacterized protein	1205	I3LKE2	3.94	0.0009
	uncharacterized protein	443; 283	I3LPY1	9.99	0.0001
	uncharacterized protein	432; 272	I3LRX7	9.39	0.0006
	uncharacterized protein	1343	I3LUY9	6.84	0.0013
	uncharacterized protein	234; 593	I3LUY9	7.77	0.0025
	UV excision repair protein RAD23 homolog B	160	P54727	7.57	0.0005
with sham at baseline					
	actin-binding LIM protein 1	471	O14639	3.71	0.0036
	afadin	1274	P55196	4.92	0.0021
	α-1-syntrophin	201	Q13424	6.94	0.0000
	α-1-syntrophin	189	Q13425	7.12	0.0002
	α-endosulfine	101; 109	O43768	5.29	0.0044
	α-endosulfine	59; 67; 62	O43768	5.29	0.0014
	AP-4 complex accessory subunit Tepsin	402	Q96N21	2.29	0.0007
	Arf-GAP domain and FG repeat-containing protein 1	95	P52594	5.84	0.0012
	ATP-binding cassette sub-family F member 1	228	Q8NE71	2.74	0.0014
	Bcl-2-associated transcription factor 1	397	Q9NYF8	3.75	0.0055
	β-2-syntrophin	95	Q13425	4.57	0.0011
	calnexin	565	P27824	4.41	0.0006
	calumenin	47	O43852	8.94	0.0006
	cAMP-dependent protein kinase type I-alpha regulatory subunit	82	P10644	3.45	0.0007
	C-type lectin domain family 14 member A	493	Q86T13	4.09	0.0056
	E3 ubiquitin-protein ligase MGRN1	516	O60291	2.53	0.0015
	formin-like protein 3	774; 745; 584	Q8IVF7	6.57	0.0022
	heat shock protein beta-8	24	Q9UJY1	8.52	0.0001
	MICOS complex subunit MIC60	88	Q16891	5.77	0.0008
	microtubule-associated protein 1B	1324	P46821	4.05	0.0047
	mitochondrial import inner membrane translocase subunit TIM44	134	O43615	7.66	0.0000
	myopalladin	928	Q86TC9	4.17	0.0010
	myosin-7	415; 1222; 1221	P12883	5.93	0.0003
	nexilin	16; 80	Q0ZGT2	3.37	0.0032
	nexilin	300; 364	Q0ZGT2	6.35	0.0013
	nuclear receptor-binding protein	433	Q9UHY1	5.36	0.0067
	protein LBH	64	Q53QV2	4.49	0.0047
	protocadherin-7	975	O60245	5.20	0.0018
	R3H domain-containing protein 2	425	Q9Y2K5	4.35	0.0057
	RNA-binding protein 20	686; 690	Q5T481	4.61	0.0062
	serine/threonine-protein kinase SIK3	461	Q9Y2K2	4.25	0.0039
	solute carrier family 16. member 1 (Monocarboxylic acid transporter 1) tv1	484	P53985	3.69	0.0002
	SUN domain-containing protein 2	55	Q9UH99	5.21	0.0034
	supervillin	305; 264	O95425	7.98	0.0097
	thioredoxin-related transmembrane protein 1	245	Q9H3N1	3.56	0.0044
	uncharacterized protein	37	F1RVC9	2.50	0.0008
	uncharacterized protein	179; 182	F1S2M0	7.95	0.0019
	uncharacterized protein	5	F1S5R6	4.67	0.0008
	uncharacterized protein	1368	I3L8Q0	3.19	0.0039
	uncharacterized protein	887; 1219; 1221	I3LA95	5.90	0.0056
	uncharacterized protein	254	I3LBD0	3.41	0.0002
	zinc finger Ran-binding domain-containing protein 2	153	O95218	4.13	0.0048
	14 kDa phosphohistidine phosphatase	36	Q9NRX4	8.38	0.0074
with RIPC at early reperfusion					
	adenylyl cyclase-associated protein	202	P40123	4.95	0.0012
	ADP/ATP translocase 1	42	P12235	7.23	0.0001
	ankyrin repeat and MYND domain-containing protein 2	403; 439	Q8IV38	5.79	0.0002
	ankyrin-1	12	P16157	6.98	0.0019
	band 4.1-like protein 1	667	Q9H4G0	4.63	0.0017
	Bcl-2-like protein 13	344	Q9BXK5	8.83	0.0031
	β-taxilin	554	Q8N3L3	3.67	0.0001
	calpastatin	312; 244; 222	P20810	5.08	0.0014
	caprin-1	306	Q14444	5.53	0.0017
	clustered mitochondria protein homolog	1253	O75153	5.00	0.0072
	coiled-coil domain-containing protein 124	141	Q96CT7	4.36	0.0054
	desmin	45	P01019	6.72	0.0013
	DNA-(apurinic or apyrimidinic site) lyase	136	P27695	6.03	0.0063
	E3 ubiquitin-protein ligase HUWE1	3756; 3758; 83	Q7Z6Z7	6.30	0.0001
	F-actin capping protein beta subunit variant II	263	P47756	3.64	0.0039
	fragile X mental retardation syndrome-related protein 2	654	P51116	2.83	0.0005
	galectin	25	Q3ZCW2	4.69	0.0000
	glutamate 5-kinase 1	581	Q98EZ4	9.18	0.0098
	glycogen [starch] synthase	727	P13807	4.58	0.0003
	heat shock 70 kDa protein 4	76	O88600	10.88	0.0046
	heat shock protein beta-1	178	P04792	5.38	0.0002
	heterogeneous nuclear ribonucleoproteins A2/B1	29	P22626	8.01	0.0008
	kinesin-like protein	930	P33176	4.68	0.0058
	LIM domain-binding protein 3	190	O75115	8.60	0.0009
	LIM domain-binding protein 3	267	O75116	7.28	0.0014
	LIM domain-binding protein 3	116	O75117	5.00	0.0039
	MAPK-interacting and spindle-stabilizing protein-like	15	Q8NDC0	4.95	0.0015
	melanoma-associated antigen D2	82	Q9UNF1	5.25	0.0054
	mitochondrial antiviral-signaling protein	220	Q7Z434	6.11	0.0000
	mitochondrial antiviral-signaling protein	202	Q7Z434	6.08	0.0032
	myeloid leukemia factor 1	33	P58340	9.82	0.0016
	myocardial zonula adherens protein	39	P0CAP1	5.22	0.0068
	myotilin	145	Q9UBF9	6.82	0.0004
	myotilin	496	Q9UBF9	5.25	0.0074
	nexilin	248; 312	Q0ZGT2	9.77	0.0003
	nucleosome assembly protein 1-like 4	5	Q99733	5.81	0.0026
	PGC-1 and ERR-induced regulator in muscle protein 1	138	Q149B8	5.64	0.0041
	phosphatidylethanolamine-binding protein 1	185	P31044	7.54	0.0012
	phospholipase A-2-activating protein	324	Q9Y263	6.76	0.0026
	pinin	66	Q9H307	4.54	0.0019
	progesterone receptor membrane component 2	104; 46	O15173	10.87	0.0004
	proteasome inhibitor PI31 subunit	152	Q92530	7.05	0.0091
	protein NDRG2	330	Q9UN36	6.59	0.0082
	protein phosphatase 1 regulatory subunit 7	24	Q15435	5.33	0.0029
	rho guanine nucleotide exchange factor 2	619	Q92974	4.14	0.0006
	selenocysteine lyase	17	Q96I15	6.71	0.0003
	sequestosome-1	272	Q13501	3.97	0.0028
	sodium/potassium-transporting ATPase subunit alpha	15; 16	P05023	6.62	0.0021
	starch-binding domain-containing protein 1	173	O95210	7.92	0.0005
	striatin-3	276	Q13033	3.59	0.0026
	supervillin	1091; 961	O95425	5.14	0.0054
	synaptopodin 2-like protein	939	Q9H987	3.27	0.0011
	synemin	738	O15063	7.83	0.0008
	synemin	580	O15064	6.23	0.0007
	synemin	1250	O15065	6.08	0.0015
	synemin	873	O15066	3.73	0.0029
	transforming acidic coiled-coil-containing protein 2	1594	O95359	4.72	0.0069
	transforming acidic coiled-coil-containing protein 2	2420; 2375; 602	O95359	3.42	0.0004
	transgelin	172	P37802	4.56	0.0021
	uncharacterized protein	597; 915; 828	F1RZW0	4.05	0.0015
	uncharacterized protein	91	F1SMV6	7.23	0.0037
	uncharacterized protein	21	I3L5C0	5.93	0.0004
	uncharacterized protein	223;223	I3L8Q0	8.23	0.0011
	uncharacterized protein	31	I3L8Q0	7.72	0.0008
	uncharacterized protein	63	I3L9T1	5.50	0.0022
	uncharacterized protein	297	I3L9T1	5.08	0.0001
	uncharacterized protein	934	I3L9T1	6.52	0.0065
	uncharacterized protein	384	I3LAR5	4.80	0.0011
	uncharacterized protein	1205	I3LAR5	4.25	0.0063
	uncharacterized protein	443; 283	I3LAR5	7.15	0.0017
	uncharacterized protein	1738	I3LBD6	5.92	0.0009
	uncharacterized protein	355	I3LGU7	4.40	0.0026
	uncharacterized protein	265	I3LGU7	8.57	0.0009
	uncharacterized protein	2451	I3LKE2	4.81	0.0009
	uncharacterized protein	2227	I3LU02	4.71	0.0006
	uncharacterized protein	234; 593	I3LUY9	5.72	0.0015
	uncharacterized protein	186	I3LUY9	3.96	0.0013
	uncharacterized protein	182	I3LUY9	3.71	0.0018
	UV excision repair protein RAD23 homolog B	160	P54727	5.80	0.0042
	xin actin-binding repeat-containing protein 1	205	Q702N8	3.52	0.0028
	Y-box-binding protein 3	45	P16989	7.29	0.0044

All phosphoproteins with a false discovery rate (FDR)-based statistical difference between remote ischemic preconditioning (RIPC) and sham at baseline or at early reperfusion, respectively, or difference between baseline and early reperfusion with RIPC or with sham after phosphopeptide enrichment of porcine left ventricular biopsies. All proteins were compared by unpaired (between RIPC and sham) Student’s t-tests.

The FDR-based statistically identified 175 phosphorylation sites which were associated with 116 different proteins, 39 of which were phosphorylation sites of 20 previously uncharacterized proteins (Table 2). There was an overlap of 48 phosphorylation sites of 21 identified proteins at early reperfusion with both, RIPC and sham, suggesting that phosphorylation of these proteins was induced by coronary occlusion/reperfusion per se. Fourty of these identified proteins were previously not described in cardiac cells, however, 45 proteins were previously described in cardiac cells, among them 4 in the context of ischemia/reperfusion, and 7 in relation to cardioprotection or ischemic conditioning maneuvers (Table 3).

**Table 3:** Proteins identified in human and porcine LV biopsies with a difference in FDR-based statistical analysis and their role in myocardial ischemia/reperfusion and in cardioprotection.

human LV biopsies						
protein	higher expression with	cellular localization	function	species/model	role in myocardial ischemia/reperfusion	role in cardioprotection
prostaglandin reductase 2	RIPC at early reperfusion	cytoplasm	- catalyzes the reaction of essentially inactive 15-keto- prostaglandin E2 to the active 15-keto-13,14-dihydro-prostaglandin E2	pigs/ isolated perfused rat hearts	—	-pharmacological inhibition of prostaglandin synthesis abrogated the cardioprotection by local ischemic preconditioning/postconditioning^61^
**porcine LV biopsies**						
protein, phosphorylation site	higher phosphorylation with	cellular localization	function	species/model	role in myocardial ischemia/reperfusion	role in cardioprotection
α-crystallin B, ser_59_	RIPC at early reperfusion	cytoplasm, nucleus, mitochondria	- heat shock protein with chaperone-like activity	pigs	-decreased α-crystallin B expression after 90/120 min ischemia/reperfusion	-preserved α-crystallin B expression with ischemic postconditioning^62^
			-prevents aggregation of various proteins under stress conditions	mice	-increased α-crystallin B phosphorylation (at Ser_59_) and expression in myofibrils and in mitochondria after 25/10 min ischemia/reperfusion	-infarct size reduction by α-crystallin B peptide administration^62^
				rat ventricular cardiomyocytes	—	-phosphorylation of α-crystallin B at ser_59_ mediates the protection in response to mitogen-activated protein kinase 6^63^
BAG family molecular chaperone regulator 3 (BAG3), pro_387_	RIPC at early reperfusion	cytoplasm, nucleus	-serves as cochaperone with members of the heat shock family to regulate protein quality control	neonatal mouse ventricular cardiomyocytes	-decreased BAG3 expression after 14/4 h hypoxia/reoxygenation	—
			-interacts with Bcl-2 to inhibit apoptosis	mice	—	- infarct size reduction by infection with BAG3-expressing adenovirus^64^
			-maintains the structural integrity of the sarcomere by linking filaments with the Z-disc			
calpastatin, ser_222_, pro_244_, Ile_312_	sham at early reperfusion	cytosol, endoplasmic reticulum, mitochondria, membrane	-specific inhibitior of calpain, which can contribute to induction of myocardial ischemia/reperfusion injury by the generation and release of proapoptotic factors from mitochondria^65^	isolated perfused rat hearts	-decreased calpastatin expression after 25/25 min ischemia/reperfusion	-altered myocardial calpain or calpastatin protein levels not associated with exercise-induced infarct size reduction^66^
			-involved in muscle protein degradation in living tissue	isolated perfused rat hearts	-decreased calpastatin expression after 30/120 min ischemia/reperfusion	-preserved calpastatin expression with cardioprotection by berbamine^67^
desmin, ser_45_	sham at early reperfusion	cytoplasm, cell membrane	-muscle-specific, intermediate filament	isolated perfused rat hearts	-decreased desmin expression after 30/120 min ischemia/reperfusion	-preserved desmin expression with cardioprotection by berbamine^67^
			-integrates the sarcolemma, Z disk, and nuclear membrane in sarcomeres			
			-regulates sarcomere architecture			
galectin 3, ser_25_	RIPC/sham at early reperfusion	cytoplasm, nucleus, mitochondria	-pleiotropic lectin	mice	- increased galactin 3 gene expression after 30 min/7 d ischemia/reperfusion^68^	—
			-involved in cell adhesion, cell activation and chemoattraction, cell growth and differentiation, cell cycle, and apoptosis			
MICOS complex subunit MIC60, met_88_	sham at baseline	membrane, mitochondria	-maintains crista junctions, inner membrane architecture	isolated perfused rat hearts	—	-increased MICOS complex subunit Mic60expression, when protection by ischemic postconditioning abrogated by mitoKATP blockade^69^
mitogen-activated protein kinase 3/mitogen-activated protein kinase 1 (ERK1/2), lys_134_/ile_90_, pro_152_	RIPC at early reperfusion	cytoplasm, nucleus, mitochondria	-protein-serine/threonine kinases that participate in the Ras-Raf-MEK-ERK signal transduction cascade	isolated perfused rat hearts	-decreased phosphorylation of ERK1/2 at thr_202_/tyr_204_ after 30/120 min ischemia/reperfusion	-phosphorylation of ERK1/2 at thr_202_/tyr_204_ causally involved in cardioprotection by RIPC^28^
			-involved in cell adhesion, cell cycle progression, cell migration, cell survival, differentiation, metabolism, proliferation, and transcription	pigs/patients	-increased phosphorylation of ERK1/2 at thr_202_/tyr_204_ after ischemia/reperfusion	-phosphorylation of ERK1/2 at thr_202_/tyr_204_ not associated with cardioprotection by RIPC^26, 28^
			-central component of the reperfusion injury salvage kinase (RISK) pathway^70^			
nexilin, Lys_16_, Ser_80_, Lys_300_, ile_364_/lys_248_, ile_312_	sham at baseline/early reperfusion	cytoplasm, cytoskeleton	-filamentous actin-binding protein	neonatal rat ventricular cardiomyocytes	-decreased nexilin expression after 2/3 h hypoxia/reoxygenation^71^	—
			-involved cell adhesion and migration			
sequestosome-1 (p62), thr_269_/ser_272_	sham at early reperfusion	cytoplasm, endoplasmic reticulum, endosomes, lysosomes, nucleus, mitochondria	-autophagosome cargo protein	isolated perfused rat hearts	- increased p62 expression after 30/30 min ischemia/reperfusion	-decreased expression of p62 by ischemic preconditioning^72^
			- targets other proteins for selective autophagy			- p62 recruitment to mitochondria associated with infarct size reduction by ischemic preconditioning^73^
			-decrease of p62 expression is associated with activation of autophagy	patients	-expression did not change after early reperfusion	- p62 expression not different between RIPC and sham^27^
sodium/potassium-transporting ATPase subunit α, val_15_, ser_16_	sham at early reperfusion	cell membrane, membrane	- catalytic component of the active enzyme, which catalyzes the hydrolysis of ATP coupled with the exchange of sodium and potassium ions across the plasma membrane	isolated perfused rat hearts	- decreased activity and expression after 45/20-40 min ischemia/reperfusion^74^	—
xin actin-binding repeat-containing protein 1, ser_205_	sham at early reperfusion	cell junction	- protects actin filaments during depolymerization	isolated perfused mice hearts	- upregulated gene expression after 25/45 min ischemia/reperfusion^75^	—

### Comparison and verification of proteome/phosphoproteome analysis with Western blot analysis

**Figure 7:**
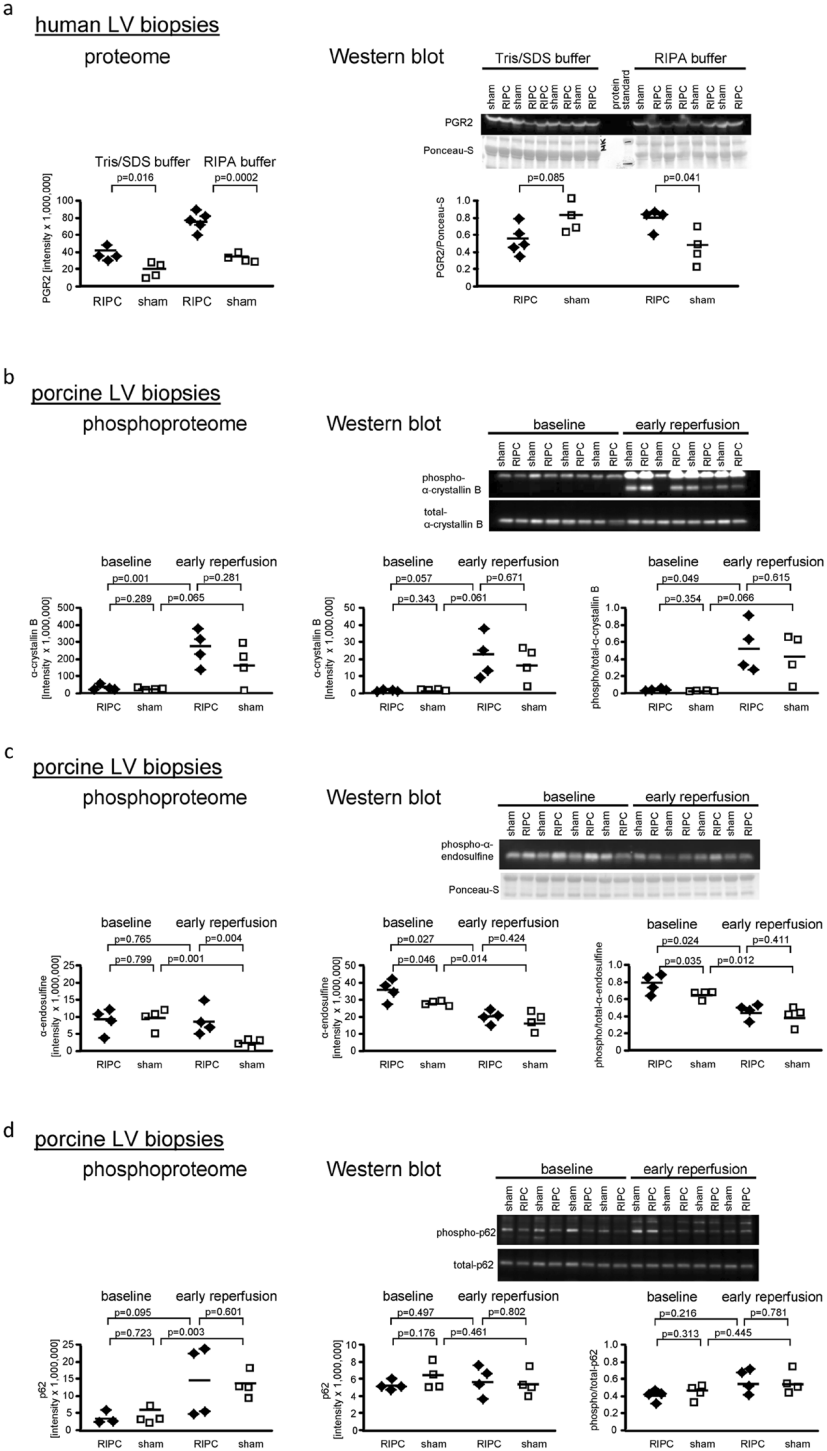
Comparison between proteome/phosphoproteome and Western blot analysis of prostaglandin reductase 2, α-crystallin B, α-endosulfine and p62 expression/phosphorylation. (**a**) The expression of prostaglandin reductase 2 (PGR2) in Tris/sodium dodecyl sulfate (Tris/SDS) and in radioimmunoprecipitation assay (RIPA) buffer lysed human left ventricular (LV) biopsies with remote ischemic preconditioning (RIPC; black symbols) and with sham (white symbols) in the proteome and in the Western blot analysis. The higher expression of PGR2 with RIPC than with sham in RIPA buffer-lysed human LV biopsies was confirmed by Western blot analysis. The immunoreactivity of PGR2 was normalized to Ponceau-S staining. The phosphorylation of (**b**) α-crystallin B at ser_59_, (**c**) α-endosulfine at ser_67_ and (**d**) p62 at thr_269_/ser_272_ in the phosphoproteome and in the Western blot analysis with RIPC and with sham in porcine left ventricular biopsies taken at baseline and at early reperfusion. The phosphoproteome analysis was verified by Western blot analysis for α-crystallin B and p62, but not for α-endosulfine. The immunoreactivities of the phosphorylated proteins were normalized to the respective total forms and compared by unpaired (between RIPC and sham) or by paired (between baseline and early reperfusion) Student’s t-tests. Total α-endosulfine was not detectable, therefore phosphorylated α-endosulfine was normalized to Ponceau-S staining. The blots were cropped to display the relevant bands, full-length blots and Ponceau-S stainings are presented in Supplemental Figs S6 and S7.

In human LV biopsies taken at early reperfusion and lysed with RIPA buffer, the FDR-based statistical analysis identified prostaglandin reductase 2 at ≥2-fold higher expression with RIPC than with sham after in-solution digestion. This ≥2-fold higher expression of prostaglandin reductase 2 with RIPC than with sham was also detected in the Tris/SDS buffer-lysed biopsies after in-solution digestion, but without FDR-based statistical significance (Fig. 7a and Supplemental Table S1 (ID: Q8N8N7)). The higher expression of prostaglandin reductase 2 with RIPC than with sham in RIPA buffer-lysed human LV biopsies was confirmed by Western blot analysis, whereas its expression in Tris/SDS buffer-lysed biopsies was not significantly higher with RIPC than with sham, in line with the FDR-based statistical analysis (Fig. 7a and Supplemental Fig. S6).

In porcine LV biopsies, the FDR-based statistical analysis identified a total of 175 phosphorylation sites, which were different between RIPC and sham at early reperfusion or between baseline and early reperfusion with RIPC or sham, respectively (Table 2). However, only 3 antibodies were commercially available to detect the respective phosphorylation sites, i.e. for: α-crystallin B, α-endosulfine, and p62.

The phosphorylation of α-crystallin B at ser_59_ was increased in the time course from baseline to early reperfusion with RIPC. This increased phopsphorylation was confirmed by Western blot analysis. However also with sham, phosphoproteome and Western blot analysis identified an increased phosphorylation of α-crystallin from baseline to early reperfusion by trend (Fig. 7b and Supplemental Fig. S7). The proteome and the Western blot analysis did not reveal any difference of α-crystallin B expression between baseline and early reperfusion and between RIPC and sham, respectively.

The decrease in the phosphorylation of α-endosulfine at ser_67_ from baseline to early reperfusion with sham by phosphoproteome analysis was confirmed by Western blot analysis. However, different from the phosphoproteome analysis, the phosphorylation of α-endosulfine was also decreased from baseline to early reperfusion with RIPC and was higher with RIPC than with sham at baseline in the Western blot analysis (Fig. 7c and Supplemental Fig. S7). The expression of α-endosulfine was not detectable with the used antibody in the Western blot analysis; its expression was also not detected in the proteome analysis.

Increased phosphorylation of p62 at ser_272_ at from baseline to early reperfusion with sham was confirmed via Western blot analysis by trend, when normalized to total p62 (Fig. 7d and Supplemental Fig. S7). However, this antibody detected phosphorylation of p62 not only at ser_272_, but also at thr_269_. The proteome analysis did not detect p62 expression per se.

### In-silico pathway analysis of human and porcine LV biopsies: relation to mitochondria and cytoskeleton

AIl proteins detected in human and in porcine LV biopsies taken at early reperfusion which had ≥2-fold higher phosphorylation/expression with RIPC than with sham (Figs 3 and 5, line (f)), respectively, were considered for an in-silico pathway analysis. Almost none of these proteins displayed conformity between humans and pigs; only 15% and 8% of the proteins were detected at early reperfusion in both species with RIPC or with sham, respectively.

In human LV biopsies, we identified 774 proteins which had ≥2-fold higher expression/phosphorylation with RIPC than with sham and 379 proteins which had ≥2-fold higher expression/phosphorylation with sham than with RIPC (Fig. 3, line (f)). A comparison between these proteins identified in more than one lysis or digestion method just 30 of the total 774 proteins at higher expression with RIPC and 6 of the total 379 proteins at higher expression with sham, respectively (Supplemental Tables S1–S4).

**Figure 8:**
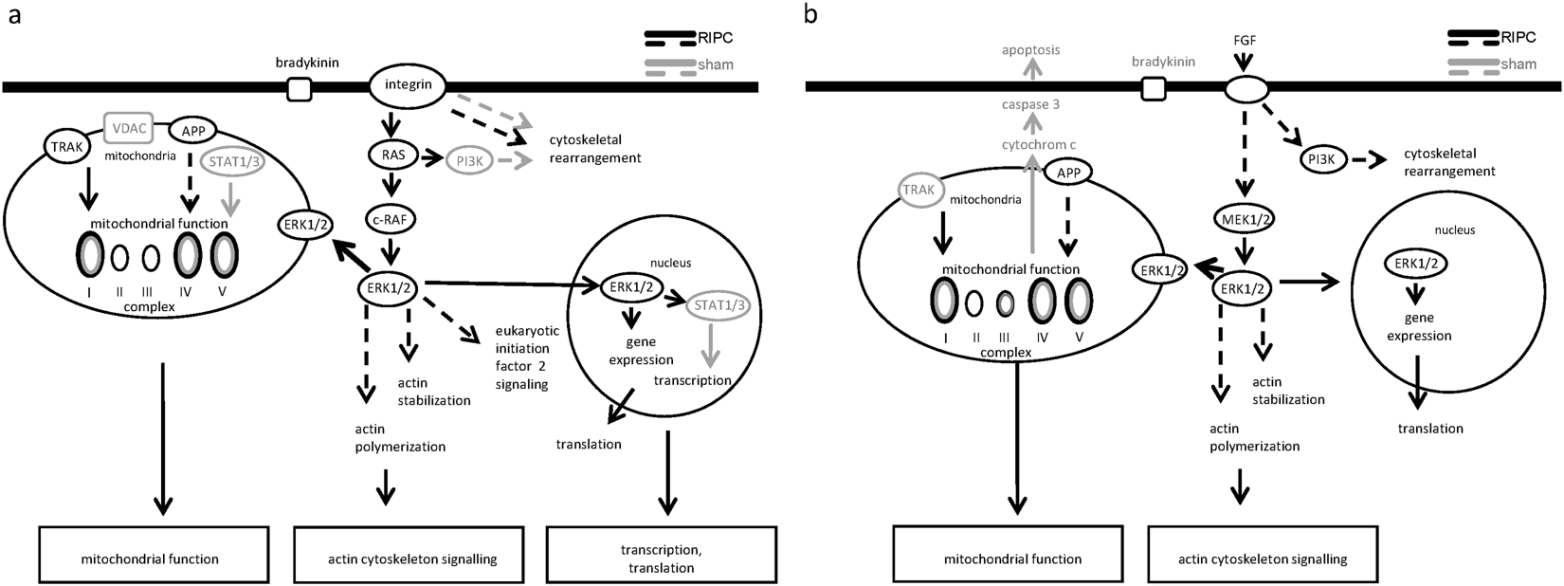
In-silico pathway analysis. Independently of lysis and digestion methods, all proteins detected at a ≥2-fold higher expression/phosphorylation with RIPC versus with sham and those exclusively detected in one group (RIPC/sham) at early reperfusion in human and porcine LV biopsies were considered for an Ingenuity pathway analysis. This pathway analysis does not distinguish between activation (phosphorylation) or expression of the detected proteins. (**a**) Proteins having higher expression/phosphorylation with RIPC than with sham in human LV biopsies at early reperfusion after cardioplegic ischemic arrest are related to mitochondrial function, cytoskeleton and transcription/translation. (**b**) Proteins having higher expression/phosphorylation with RIPC than with sham in porcine LV biopsies at early reperfusion after coronary occlusion are related to mitochondrial function and cytoskeleton. Proteins marked in black had higher expression/phosphorylation with RIPC than with sham, and proteins marked in grey had higher expression/phosphorylation with sham than with RIPC. Continuous arrows are reflecting direct relations and broken arrows are reflecting indirect relations. APP: amyloid precursor protein, c-RAF: rapidly accelerated fibrosarcoma, ERK1/2: mitogen-activated protein kinase 3/mitogen-activated protein kinase 1, STAT1/3: signal transducer and activator of transcription 1/3, TRAK: trafficking kinesin protein, VDAC: voltage-dependent anion channel.

In porcine LV biopsies taken at early reperfusion, 611 proteins had ≥2-fold higher expression/phosphorylation with RIPC than with sham and 374 proteins had ≥2-fold higher expression/phosphorylation with sham than with RIPC (Fig. 5, line (f), Supplemental Tables S5 and S4), but 115 (RIPC) and 117 (sham) proteins were labeled as uncharacterized proteins, and therefore could not be analyzed further. In-silico analysis revealed similar pathways for both species: proteins having higher expression/phosphorylation at early reperfusion with RIPC than with sham were related to mitochondrial function, cytoskeleton and epithelial adherens junction signaling, proteins having higher expression/phosphorylation with sham than with RIPC were related to tight junction signaling (Fig. 8 and Table 4).

**Table 4:** In-silico pathway analysis.

	in-silico identified pathways (identified proteins of Ingenuity pathway analysis listed proteins)
	human LV biopsies
**RIPC**	cytoskeleton (30 of 228)
	epithelial adherens junction signaling (20 of 146)
	eukaryotic initiation factor 2 signaling (24 of 194)
	integrin signaling (25 of 219)
	mitochondrial function (24 of 171)
**sham**	epithelial adherens junction signaling (12 of 146)
	melatonin (8 of 71)
	protein kinase A signaling (12 of 146)
	tight junction signaling (12 of 167)
	**porcine LV biopsies**
**RIPC**	calcium signaling (26 of 178)
	cytoskeleton (25 of 228)
	epithelial adherens junction signaling (21 of 146)
	mitochondrial function (18 of 171)
	protein kinase A signaling (35 of 392)
**sham**	cytoskeleton (21 of 228)
	integrin linked kinase signaling (20 of 196)
	mitochondrial function (17 of 171)
	tight junction signaling (17 of 167)

In-silico pathway analysis was performed with all identified proteins having ≥2-fold higher expression/phosphorylation with remote ischemic preconditioning (RIPC) versus with sham in human left ventricular (LV) biopsies taken at early reperfusion after cardioplegic ischemic arrest and in porcine LV biopsies taken at early reperfusion after coronary occlusion using Ingenuity pathway analysis software.

## Discussion

The current unbiased, non-hypothesis-driven proteomics approach to analysis of human LV biopsies taken at early reperfusion after cardioplegic ischemic arrest identified only prostaglandin reductase 2, but no other established or previously unknown differences in protein expression by cardioprotection with RIPC. The phosphoproteome analysis of porcine LV biopsies, however, identified some phosphoproteins, which may be potential candidates for further analysis of cardioprotective signals.

In both species, the protection as such was confirmed in phosphoproteome/proteome analysis by higher tissue expression/activation of troponin I and troponin T with RIPC than with sham (Supplemental Tables S1 and S3).

A differential expression of total protein in LV biopsies from patients undergoing RIPC has not been reported so far^26, 27^, and indeed biosynthesis of new proteins is unlikely in the short time interval between the end of the RIPC/sham maneuver and the biopsy sampling at early reperfusion after cardioplegic ischemic arrest (Table 1). Therefore, changes in protein expression most likely reflect changes in proteolysis induced by ischemia. However, the FDR-based statistical analysis only identified prostaglandin reductase 2 at higher expression with RIPC than with sham in human LV biopsies. Prostaglandins are associated with cardioprotection by local ischemic preconditioning in pigs^35^ (Table 3). Thus, prostaglandin signaling may indeed be involved in cardioprotection by RIPC in the human myocardium. Again, however, a decreased proteolysis of prostaglandin reductase 2 by RIPC could cause this difference in expression, and the relative increase of prostaglandin reductase 2 in myocardium with RIPC than with sham may not at all reflect an increased prostaglandin concentration. Unfortunately, we were unable to determine changes in the prostaglandin concentration in the lysates from the available LV biopsies.

In porcine LV biopsies, the FDR-based statistical analysis did not identify any difference in protein expression between RIPC and sham or between baseline and early reperfusion, and thus did not confirm a higher prostaglandin reductase 2 expression at early reperfusion with RIPC than with sham.

Cardioprotective proteins are mainly regulated by phosphorylation^33^. Previously, we and others have used Western blot analysis and reported an increased phosphorylation of STAT5 in patients^26, 27, 36^ and of STAT3 in pigs^28^ at early reperfusion with protection by RIPC. Western blot analysis of the LV biopsies in the present study revealed a trend of increased STAT5/STAT3 phosphorylation at early reperfusion with RIPC than with sham, respectively, thus confirming our prior results. However, neither the expression nor the phosphorylation of STAT5 in patients and of STAT3 in pigs, respectively, were detected by the present proteome and phosphoproteome analysis. The extraction of high abundance-proteins and their detection via mass spectrometry^37^ may have hampered the detection of potentially small amounts of STAT5 or STAT3 in the proteome and phosphoproteome analysis.

In human LV biopsies, FDR-based statistical analysis did not reveal any difference in protein phosphorylation between RIPC and sham at early reperfusion. The reason for this unsatisfactory result may be related to the only small cohort of patients with its uneven distribution of co-morbidities and co-medications (Table 1), which both, potentially interfere with protection by ischemic conditioning maneuvers^29, 34^.

In contrast to patients, our translational pig model had less interindividual variability and no co-morbidities and co-medications^34^. In pigs, LV biopsies were also taken at baseline and at early reperfusion, allowing an intraindividual analysis of time course. This intraindividual comparison resulted in a relatively large yield of FDR-based statistically identified differences of protein phosphorylation between baseline and early reperfusion with RIPC and/or with sham, respectively (Table 2). Unfortunately, these identified proteins included 18% uncharacterized proteins as well as 35% candidates about which nothing is known for their role in cardiac cells. A verification of the characterized proteins via Western blot with commercially available antibodies was only possible for 3 candidates. From these 3 proteins, we indeed verified the phosphoproteome results for α-crystallin B and p62, but not for α-endosulfine. Alpha-endosulfine is a cytoplasmic, highly conserved cAMP-regulated phosphoprotein and regulator of KATP-channels, which modulates insulin secretion in the pancreas^38^. However, a role of α-endosulfine in cardiac cells is entirely unclear.

Among the identified proteins, 11 proteins have already been described in relation to myocardial ischemia/reperfusion and/or cardioprotection. These prior studies analyzed almost exclusively the expression and not the phosphorylation of these candidate proteins; therefore, nothing is known yet about the role of the here identified phosphorylation sites. These proteins may, however, be potential novel candidates as cardioprotective signals induced by RIPC than with sham (Table 3), and it may be worthwhile to analyze them further.

Independent of the FDR-based statistical analysis, the phosphoproteome analysis of porcine LV biopsies revealed a shift towards a greater number of proteins with increased phosphorylation at early reperfusion with RIPC than with sham (Fig. 6b and c). Such shift was not seen after phosphoproteome analysis of human LV biopsies.

Almost all identified proteins with a different expression/activation at early reperfusion were different between humans and pigs, reflecting and confirming the established species-specific differences. However, without differentiation between expression and phosphorylation of all detected proteins, the Ingenuity knowledge base identified mitochondria and cytoskeleton in association with RIPC at early reperfusion in both species. These associations may or may not relate to their causal involvement in cardioprotection by ischemic conditioning strategies (Fig. 8).

Mitochondria are well established end-effectors of cardioprotective strategies, and the preservation of mitochondrial function after ischemia/reperfusion is decisive for the survival of cardiomyocytes and thus salvage of myocardium^22, 39^. RIPC preserved mitochondrial respiration after ischemic cardioplegic arrest compared to sham in right atrial appendages of patients undergoing CABG^40, 41^. The plasma transfer from pigs, which had undergone RIPC, to isolated perfused rat hearts reduced infarct size and improved mitochondrial function at early reperfusion after global ischemia^42^.

The protection by simulated ischemic preconditioning was associated with attenuated osmotic fragility through cytochalasin D–sensitive stabilization of the actin cytoskeleton in isolated rabbit cardiomyocytes after simulated ischemia for 3 h^43^. Also, the phosphorylation of sarcomeric Z-disc associated proteins was increased immediately after the RIPC procedure compared to that after sham procedure in mice hearts^44^. Pharmacological interventions induced long-term expression changes of proteins related to cytoskeletal regulation in pig hearts^45^.

There are several limitations to our current analysis: 1) We analyzed biopsies from only a small cohort of patients with an uneven distribution of co-morbidities and co-medications (Table 1), which both, potentially interfere with ischemic conditioning maneuvers^29, 34^. To overcome this issue and to increase the power of a proteome analysis, the inclusion of more patients with a more balanced set of co-morbidities and co-medications would be required. 2) The size of human and porcine biopsies was small and precluded a parallel extraction of proteins from one sample with different lysis and digestion methods. A small sample size may result in a sampling error and a high variability of its cellular composition. 3) Given the small sample size, we analyzed total myocardial proteins and did not distinguish between different cellular and subcellular compartments. Proteome analysis targeting different, isolated cellular and subcellular compartments may decipher more complex, multivalent signals^37^. 4) We analyzed only one posttranslational protein modification, i.e. protein phosphorylation. Although cardioprotective proteins are mainly regulated by phosphorylation^33^, we cannot exclude differences by RIPC in other posttranslational modifications. Sufficient tissue sampling for additional analysis of acetylation, O-linked β-N-acetylglucosaminylation, S-nitrosylation, etc. was not possible. 5) Although we identified 116 proteins with a difference in phosphorylation mainly between baseline and early reperfusion in pigs with RIPC and/or with sham, only 3 antibodies were commercially available against proteins with the identified phosphorylation sites to verify the phosphoproteome data by Western blot analysis. 6) We analyzed human LV biopsies at only one time point, i.e. at early reperfusion after cardioplegic ischemic arrest. Biopsies taken at baseline after RIPC/sham, but before cardioplegic ischemic arrest were not available. Thus, in patients, we cannot distinguish between changes from baseline to early reperfusion after cardioplegic ischemic arrest induced by RIPC versus sham and, thus, between biosynthesis versus proteolysis. 6) The protein data repositories for pigs are not complete yet. In fact, 39 phosphorylation sites were identified with FDR-based statistical differences particularly between baseline and early reperfusion and had to be defined as uncharacterized proteins.

In conclusion, the current proteome and phosphoproteome analysis of human LV biopsies from patients undergoing CABG identified only prostaglandin reductase 2, but no other established difference in protein expression and activation by cardioprotection with RIPC at early reperfusion after cardioplegic ischemic arrest. However, the higher expression of prostaglandin reductase 2 with RIPC than with sham may point towards an involvement of prostaglandin metabolism in cardioprotective signaling in patients. In contrast, the phosphoproteome analysis of porcine LV biopsies taken at baseline and early reperfusion after coronary occlusion identified some previously unknown differences in protein phosphorylation between RIPC and sham, which are potential candidates for further investigation. The present patient cohort with its uneven distribution of co-morbidities and co-medications, the individual sampling, the sample processing for this approach, the number of uncharacterized or unverifiable porcine proteins, the method of proteome and phosphoproteome analysis and its bioinformatical evaluation per se may all have contributed to this unsatisfactory result.

In the future, specific phosphoproteome rather than proteome analysis of different, isolated cellular and subcellular compartments of myocardial biopsies taken at baseline and early reperfusion may reveal more insights into the signal transduction of cardioprotection by RIPC. Furthermore, independent analysis methods must be used for the characterization and validation of potentially identified pathways.

## Methods

### Materials

Chemicals were of the highest quality available, and all solutions were freshly prepared using MilliQ® water or high quality analytical grade organic solvents. Materials were obtained from Sigma-Aldrich (Deisenhofen, Germany) or purchased as indicated.

### Patient study

The inclusion and exclusion criteria for as well as the results of the clinical trial (ClinicalTrials.gov NCT01406678, date of registration: December 1, 2009) have been reported^7^. The study conforms to the principles of the Declaration of Helsinki. With approval of the local ethics committee (Germany: Institutional Review Board, University of Duisburg-Essen, no. 08–3683) and patients’ written informed consent, LV biopsies were harvested in a subgroup of patients undergoing elective isolated first-time CABG, who were enrolled in this randomized, prospective, double-blind, placebo-controlled study without (sham) or with RIPC.

Anesthesia was induced with sufentanil (1 µg/kg), etomidate (0.3 mg/kg) and rocuronium (0.6 mg/kg) and maintained with isoflurane (0.6–1.0% end-tidal). The RIPC protocol consisted of 3 cycles of 5 min left upper arm ischemia/5 min reperfusion and was compared to sham (cuff left deflated for 30 min). Surgical revascularization was performed in all patients using median sternotomy, mild systemic hypothermia (>32 °C) and antegrade cold crystalloid Bretschneider (Köhler Chemie GmbH, Bensheim, Germany) cardioplegia with additional topical cooling and single aortic cross-clamping for all distal anastomoses^7^.

#### Human LV biopsies

Transmural LV biopsies of 2–5 mg were available from 22 patients (n = 11/11 RIPC/sham). LV biopsies were taken at 5–10 min reperfusion following aortic unclamping from the perfusion territory undergoing revascularization using a Tru-Cut R biopsy needle (Cardinal Health, Dublin, OH, USA). Biopsies were quickly frozen in liquid nitrogen and stored at −80 °C until subsequent analysis.

#### Serum troponin I

Venous blood samples were drawn from each patient on the day before surgery and postoperatively at 1, 6, 12, 24, 48, and 72 h and analyzed for serum cTnI. The AUC for serum cTnI was calculated according to the trapezoidal rule. Missing values were replaced by linear inter- and extrapolation^7^.

### Pig studies

In a translational pig model, we analyzed the proteome and phosphoproteome of residual lysates of LV biopsies from pigs which had undergone coronary occlusion/reperfusion without (sham) or with RIPC in prior studies^28^. The experimental protocol was approved by the Landesamt für Natur, Umwelt und Verbraucherschutz Nordrhein-Westfalen, Germany (B1322/12) and the investigation conforms to the Guide for the Care and Use of Laboratory Animals published by the US National Institutes of Health (NIH Publication No. 85-23, revised 1996).

Male Göttinger minipigs were anesthetized with etomidate (0.3 mg/kg, Hypnomidat; Janssen-Cilag, Neuss) and sufentanil (1 μg/kg IV, Sufenta; Janssen-Cilag, Neuss, Germany), and anesthesia was maintained with isoflurane (2%) in oxygen-enriched air. The RIPC protocol consisted of 4 cycles of 5 min left hindlimb ischemia/5 min reperfusion (n = 4) and was compared to sham (n = 4; tourniquet not fixed for 40 min). After a left lateral thoracotomy, a silk suture was placed around the left anterior descending coronary artery (LAD) distal to its second diagonal branch for coronary occlusion. The suture around the LAD was carefully tightened against a soft silicone plate for 60 min. Reperfusion was induced by release and quick removal of the suture. For details see^28^.

#### Porcine LV biopsies

LV biopsies were sampled at baseline (after RIPC/sham and before 60 min LAD occlusion) and at 10 min reperfusion with a modified dental drill. Biopsies were quickly frozen in liquid nitrogen and stored at −80 °C until subsequent analysis.

#### Infarct size in pig hearts

At the end of each experiment, the LAD was re-occluded, and 5 ml blue dye (Patentblau V, Guerbet GmbH, Sulzbach, Germany) was quickly injected into the left atrium to delineate the area at risk as remaining unstained. The heart was then arrested by electrical induction of fibrillation, removed from the chest and sectioned from base to apex into 5 transverse slices. Slices were photographed from each side, and their shape and the unstained area at risk were traced manually on transparent film. Slices were then immersed in 0.09 mol/l sodium phosphate buffer containing 1% triphenyl tetrazolium chloride and 8% dextran for 20 min at 37 °C to demarcate viable from infarcted tissue. The infarcted areas were traced on the same transparent film as the area at risk. The total slice area, the area at risk, and the infarcted area were measured by computer-assisted planimetry. The area at risk was calculated as a fraction of the LV, and the infarct size was calculated as a fraction of the area at risk.

### Sample processing of LV biopsies

Human LV biopsies (n = 11/11 RIPC/sham) were randomly split into two subsets and lysed either in the Tris/SDS (n = 6/6 RIPC/sham) or the RIPA (n = 5/5 RIPC/sham) buffer to obtain a broad range of solubilized proteins of all cellular components. Tris/SDS buffer, with SDS as ionic detergent, was used to gain a high protein yield of all cellular components, whereas RIPA buffer was used to extract predominantly nuclear, mitochondrial, cytoplasmic and intracellular proteins and to a lesser extent membrane, cytoskeletal and extracellular proteins^46^. The frozen LV biopsies were homogenized using a tissue homogenizer (Ultra-Turrax, IKA, Staufen, Germany) either in 0.1 mol/l Tris and 2% (w/v) SDS buffer and incubation for 5 min at 95 °C or in 1 × RIPA buffer (Cell Signaling, Danvers, MA, USA) supplemented with 1 × complete protease inhibitor cocktail (Roche, Basel, Switzerland). All samples were centrifuged at 16,000 g for 5 min. The protein concentration of the supernatant was determined using a protein assay (Bradford method, Biorad, Hercules, CA, USA) with bovine serum albumin (BSA) as standard (Thermo Scientific, Waltham, MA, USA).

The protein yield of the Tris/SDS buffer-lysed LV biopsies was sufficient to perform subsequent in-solution digestion and phosphopeptide enrichment (n = 6/6 RIPC/sham), respectively. For in-gel digestion, the protein yield of only n = 3/3 RIPC/sham lysates was sufficient. The protein yield of RIPA buffer-lysed LV biopsies (n = 5/5 RIPC/sham) was too low and precluded phosphopeptide enrichment and in-gel digestion. Thus, these lysates were exclusively used for in-solution digestion. Separate biopsies from the same pigs (n = 4/4) were taken at baseline and at early reperfusion, respectively, lysed in Tris/SDS buffer, and used for in-solution digestion and phosphopeptide enrichment.

#### In-solution digestion

Lysates (15 µg protein) were precipitated overnight with ice-cold acetone at −20 °C and then centrifuged (15,000 g, 10 min at 4 °C). Protein pellets were washed with 90% ice-cold acetone, centrifuged again (15,000 g, 10 min at 4 °C) and dissolved with 6 mol/l urea, 2 mol/l thiourea, 10 mmol/l 4-(2-hydroxyethyl)-1-piperazineethanesulfonic acid (HEPES), pH 8. Disulfide bonds on cysteine were reduced by adding 10 mmol/l dithiothreitol and alkylated by adding 55 mmol/l iodoacetamide. The endoproteinase Lys-C (Wako Chemicals, Osaka, Japan) was added at an enzyme:substrate ratio of 1:100 and incubated at room temperature for 120-180 min. The lysates were diluted with 50 mmol/l ammonium bicarbonate, and trypsin (Promega, Fitchburg, WI, USA) was added at an enzyme:substrate ratio of 1:100 and incubated at room temperature overnight. The digests were quenched by trifluoroacetic acid, and samples containing tryptic peptides were desalted and concentrated with “Stop and Go extraction” tips filled with C-18 (C-18 Empore Disks, 3 M, Minneapolis, MN, USA)^47, 48^.

#### Phosphopeptide enrichment

Phosphopeptide enrichment was performed using titanium dioxide, which enables robust and reproducible isolation of phosphopeptides from small amounts of tissue/cell material^49^. Samples were acidified by 6% trifluoroacetic acid after in-solution trypsin digestion. Titanium dioxide beads (0.1 mg/µl in 60% acetonitrile/6% trifluoroacetic acid) were added to the samples with a peptide:beats ratio of 1:3, incubated for 20 min on a rotor wheel and centrifuged (500 g, 1 min, room temperature). With the supernatant, the latter steps were repeated three times. Beads were transferred to C-8 columns, washed three times with 60% acetonitrile/1% trifluoroacetic acid and then with 80% acetonitrile/0.5% formic acid. Phosphopeptides were eluted with 40% acetonitrile/3.75% ammonium hydroxide, pH 10.5 and 80% acetonitrile. Samples were vacuum-centrifuged for 90 min at 30 °C until the volume of the samples was ~2 µl and filled up to 10 µl with 60% acetonitrile/6% trifluoroacetic acid.

#### Gel electrophoresis and in-gel digestion

LV biopsies were lysed in Tris/SDS buffer and loaded on a 4%–12% Bis-Tris gel (NuPAGE, Invitrogen, Carlsbad, CA, USA). After colloidal blue staining, evenly sized gel pieces were excised from the gel and digested with trypsin. Briefly, gel pieces were reduced with 10 mmol/l dithiothreitol, alkylated with 55 mmol/l iodoacetamide and digested with trypsin overnight. Peptides were extracted from the gel using an increasing acetonitrile concentration. Collected peptide mixtures were concentrated and desalted using the “Stop and Go extraction” tips^47^.

### Liquid chromatography and mass spectrometry

A binary buffer system consisting of buffer A (0.1% formic acid) and buffer B (80% acetonitrile, 0.1% formic acid) was used for peptide separation on an Easy nano-flow high performance liquid chromatography 1000 system (Thermo Scientific, Waltham, MA, USA), which was coupled via a nano electrospray ionization source to a QExactive or a linear trap Quadropole Velos mass spectrometer (Thermo Scientific, Waltham, MA, USA). Peptide elution from the in-house packed 20 cm (3 mm beads, ID: 75 mm, Dr Maisch, Ammerbuch, Germany) or 50 cm (1.8 mm beads, ID: 75 mm, Dr Maisch, Ammerbuch, Germany) column was achieved by increasing the relative amount of B from 7% to 38% in a linear gradient within 150 min or 240 min^50, 51^.

#### QExactive and linear trap quadropole Velos

Mass spectrometry spectra were recorded at 70,000 resolution (200 m/z, 3E6 ions as the automatic gain control target) within a maximum injection time of 20 ms. Acquisition of tandem mass spectrometry (MS/MS) spectra in a data-dependent mode after higher-energy collisional dissociation fragmentation (Top10) was carried out at 17,500 (200 m/z) using 1E6 ions as the automatic gain control target and 60 ms for maximal injection time. The separation width was set to 1.7 m/z^51^.

For the identification of phosphopeptides, a linear trap quadropole-Orbitrap Velos mass spectrometer was used, and MS/MS spectra were generated by higher C-trap dissociation. Briefly, 30,000 ions were accumulated in the c-trap, and MS/MS spectra were detected in the Orbitrap at a resolution of 7,500^52, 53^.

### Data processing and analysis

Acquired raw files were processed using the MaxQuant software tool (version 1.3.7.4; Max Planck Institute of Biochemistry, Martinsried, Germany). A maximum of two missed cleavages and a mass tolerance of 4.5 ppm and 7 ppm for MS/MS first and main search were set, respectively. A minimal peptide length of seven amino acids after Lys-C specificity for protein assignment and a minimal ratio count of two for quantification were required. For further data analysis, proteins/phosphopeptides were defined as detected proteins/phosphopeptides when measured at least in 50% of the samples in one group, respectively. For a number of proteins, which were identified as uncharacterized proteins in porcine LV biopsies by FDR-based statistical analysis, the corresponding IDs for humans were identified by the gene name, when available in the universal protein database UniProt (www.uniprot.org).

### Pathway analysis

Independently of lysis and digestion methods, all proteins detected at early reperfusion in humans (Fig. 3, line (f)) and in pigs (Fig. 5, line (f)) with a ≥2-fold higher expression/phosphorylation with RIPC or sham, respectively, and those exclusively detected with RIPC or sham were considered for a pathway analysis; the pathway analysis does not distinguish between activation (phosphorylation) and expression.

In pigs, we restricted this pathway analysis to all proteins with higher expression/phosphorylation detected at early reperfusion with RIPC or sham, respectively. Cardioprotective signals must be present at early reperfusion to be causally involved in final infarct size reduction.

The in-silico pathway analysis was performed with Ingenuity pathway analysis software (Ingenuity Systems, Redwood City, CA, USA)^54^.

### Western blot analysis

Residual LV biopsy lysates were used to compare the cellular composition of LV biopsies. Protein aliquots of 30 µg were electrophoretically separated on precasted SDS-polyacrylamide gels (BioRad, Munich, Germany) and transferred to polyvinylidene fluoride membranes. After Ponceau-S staining membranes were blocked and then incubated with antibodies directed against proteins encoding different cell types: CD1 (#3528) as endothelial cell marker^55^, enolase 2 (#8171) as neuronal marker^56^, hemoglobin γ (#39386; Cell Signaling Technology, Cambridge, UK) as erythrocyte marker^57^, collagen 3α (NBP2-15946, Novus Biological, Littleton, CO, USA) as fibroblast marker^58^ and tropomyosin (#T9283, Sigma-Aldrich, Deisenhofen, Germany) as cardiomyocyte marker^59^. The phosphorylation/expression of STAT5 in residual lysates from human LV biopsies and the phosphorylation/expression of STAT3 in residual lysates from porcine LV biopsies were analyzed with specific antibodies against phosphorylated STAT5 at tyr_694_ (#4322) and STAT3 at tyr_705_ (#9138) and the respective total forms of STAT5 (#9363) and STAT3 (#12640; Cell Signaling Technology, Cambridge, UK) to confirm our prior results^26–28^. The expression of prostaglandin reductase 2 (#ab84711, Abcam, Cambridge, UK), i.e. the only protein with a differential expression in human LV biopsies (see results), as well as the phosphorylation/expression of α-crystallin B (#LS-B3696; #LS-C22453, LifeSpan Biosciences, Seattle, WA, USA), α-endosulfine (#5240; #8770), and p62 (#13121; #5114, Cell Signaling Technology, Cambridge, UK), i.e. proteins where antibodies were commercially available against the identified phosphorylation site with a difference in protein phosphorylation between baseline and early reperfusion with RIPC or sham, were also analyzed.

After incubation with the respective secondary antibody, immunoreactive signals were detected by chemiluminescence and quantified with ChemoCam/LabImage1D software (INTAS, Göttingen, Germany). The immunoreactivity of PGR2 was normalized to Ponceau-S staining. The immunoreactivities of the phosphorylated proteins were normalized to the respective total forms. Total α-endosulfine was not detectable, therefore phosphorylated α-endosulfine was normalized to Ponceau-S staining. Full-length blot and Ponceau-S staining are presented in Supplemental Figs S2, S3, S4, S6 and S7, respectively.

### Literature data search

The relation to heart, ischemia and reperfusion and cardioprotection of all identified phosphopeptides/proteins with a FDR-based statistical difference was analyzed from the literature using the electronic database pubmed up to May 2017. Search keywords were “heart” or “cardiac” combined with the protein name as indicated in UniProt, if applicable also with the alternative name(s) of the respective protein. These keywords were further combined with “ischemia” and “reperfusion” or “cardioprotection” or “conditioning”.

### Statistics

Data are expressed as mean ± standard error of the mean (SEM). Statistics were performed using SigmaStat software (SigmaStat 2.03, SPSS Inc., Chicago, IL, USA). Patient baseline and intraoperative characteristics were compared using unpaired Student’s t-test (continuous data) and 2-tailed Fisher’s exact test (categorical data). Serum cTnI of patients was analyzed by 2-way (group, time) ANOVA for repeated measures. The AUC for the serum cTnI over 72 h was compared by unpaired Student’s t-test. Immunoreactivities on the blot were compared by unpaired (between RIPC and sham) or by paired (between baseline and early reperfusion in pigs) Student’s t-tests. Proteome data visualization was performed using the statistical environment R and Perseus (Max Planck Institute of Biochemistry, Martinsried, Germany)^60^. Proteome and phosphoproteome data were compared by unpaired (between RIPC and sham) or by paired (between baseline and early reperfusion in pigs) Student’s t-tests. The FDR-based statistical analysis was performed with number of randomization = 500, exchangeability factor s0 = 0.1 and q-value = 0.05. Differences were considered significant at the level of p < 0.05.

## Electronic supplementary material

Supplementary Information
