# Supplementary material for: Proteomics/phosphoproteomics of left ventricular biopsies from patients with surgical coronary revascularization and pigs with coronary occlusion/reperfusion: remote ischemic preconditioning
Source: Sci Rep. 2017 Aug 9;7:7629. doi: 10.1038/s41598-017-07883-5 (PMC5550488; doi:10.1038/s41598-017-07883-5)
Supplement: Supplementary file 1 — Supplementary Information [file 41598_2017_7883_MOESM1_ESM.docx]

Supplementary Information

**Proteomics/phosphoproteomics of left ventricular biopsies from patients with surgical coronary revascularization and pigs with coronary occlusion/reperfusion: remote ischemic preconditioning**

Nilgün Gedik, Marcus Krüger, Matthias Thielmann, Eva Kottenberg, Andreas Skyschally, Ulrich H. Frey, Elke Cario, Jürgen Peters, Heinz Jakob, Gerd Heusch, Petra Kleinbongard

**
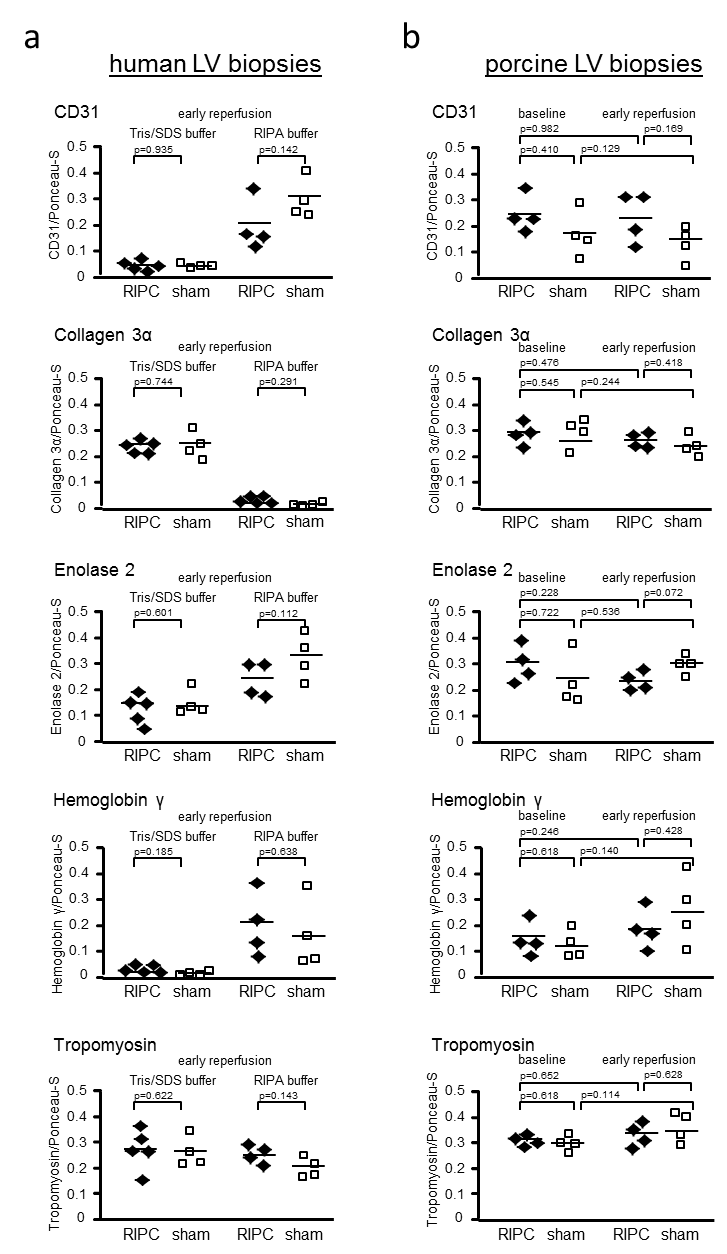
**

**Supplemental Figure S1. Expressions of proteins characterizing different cell types in human and porcine left ventricular biopsies.** (a) The expressions of CD1 (endothelial cell marker), collagen 3α (fibroblast marker), enolase 2 (neuronal marker), hemoglobin γ (erythrocyte marker) and tropomyosin (cardiomyocyte marker) were comparable between remote ischemic preconditioning (RIPC; black symbols) and sham (white symbols) in human left ventricular biopsies lysed in Tris/sodium dodecyl sulfate (SDS) or radioimmunoprecipitation assay (RIPA) buffer. (b) The expressions of different cell types were also comparable 1. between baseline and early reperfusion and 2. between RIPC and sham in porcine left ventricular biopsies. The immunoreactivities of the proteins were normalized to Ponceau-S staining and compared by unpaired (between RIPC and sham) or by paired (between baseline and early reperfusion in pigs) Student's t-tests. The full-length blots and Ponceau-S stainings are presented in Supplemental Fig. S2 and S3.

a


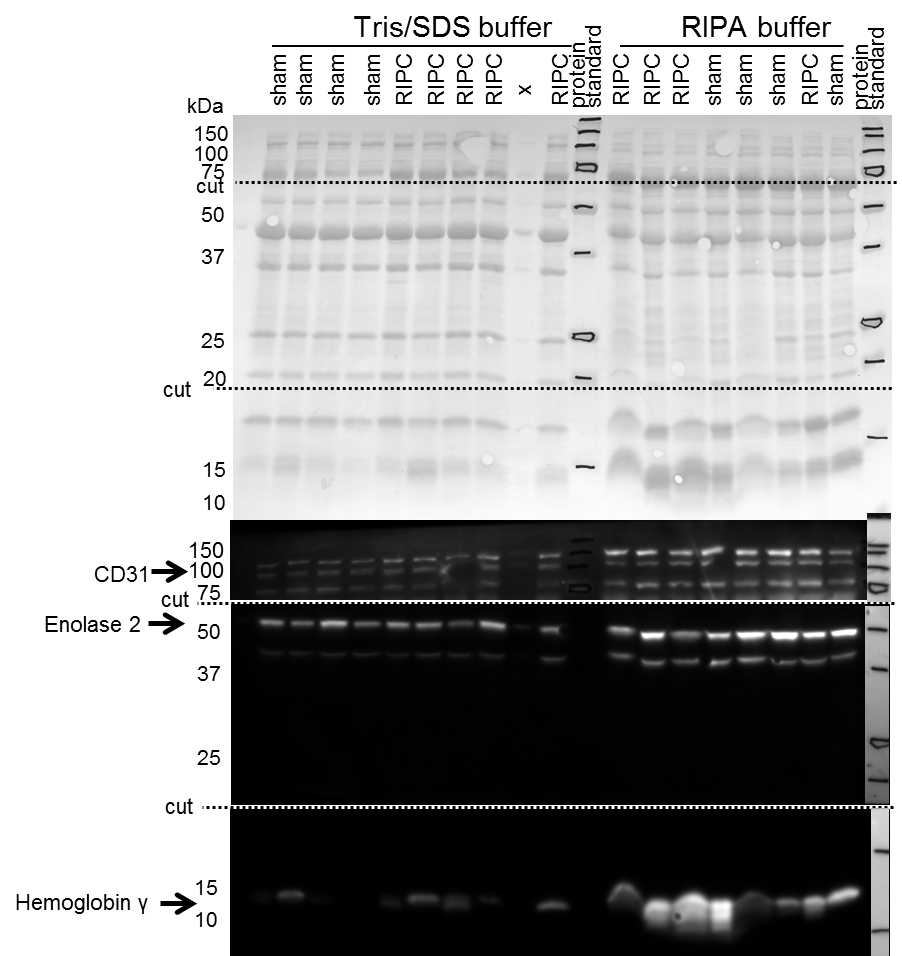


Supplemental Fig. S2 continued

b


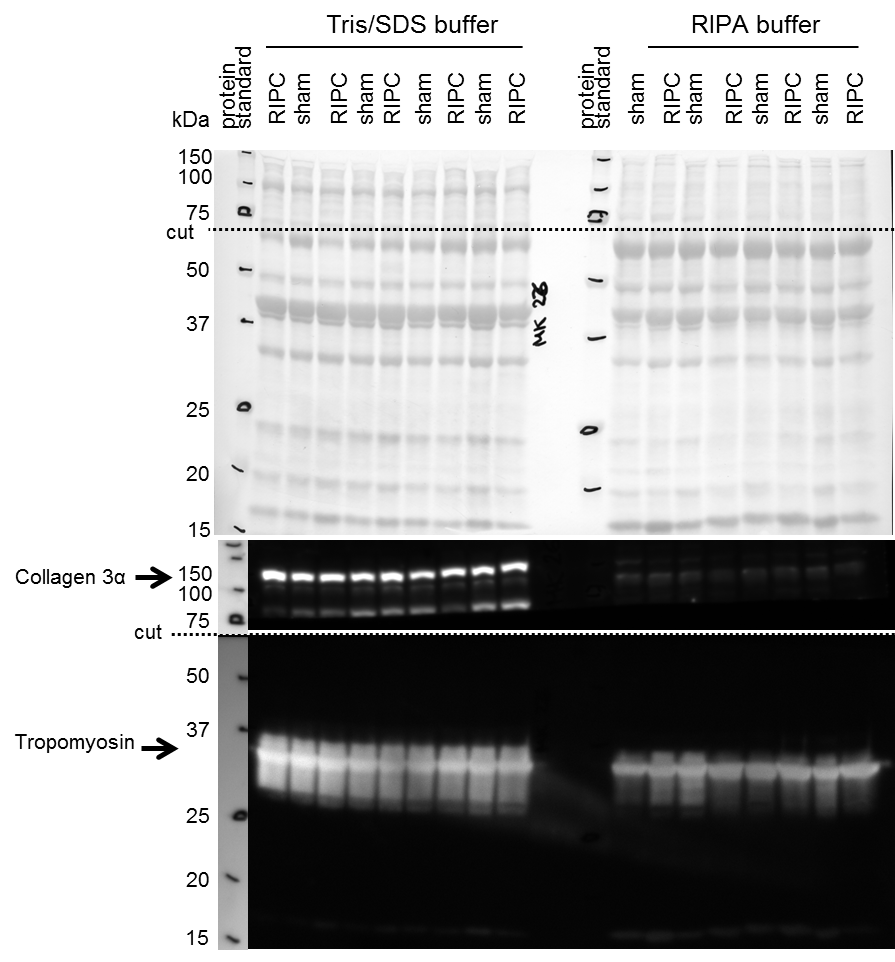


**Supplemental Figure S2. Western blot analysis of protein expressions of proteins characterizing different cell types in human left ventricular biopsies taken at early reperfsuion.** Ponceau-S stainings and full-length blots from human left ventricular biopsies. The expressions of (a) CD1 (endothelial cell marker), enolase 2 (neuronal marker), hemoglobin γ (erythrocyte marker) and (b) collagen 3α (fibroblast marker) and tropomyosin (cardiomyocyte marker) were analyzed in human left ventricular biopsies lysed in Tris/sodium dodecyl sulfate (SDS) or radioimmunoprecipitation assay (RIPA) buffer after remote ischemic preconditioning (RIPC) or sham. The protein yields of the samples were limited, therefore membranes were cut as indicated to analyze the immunoreactivities of different proteins in parallel.


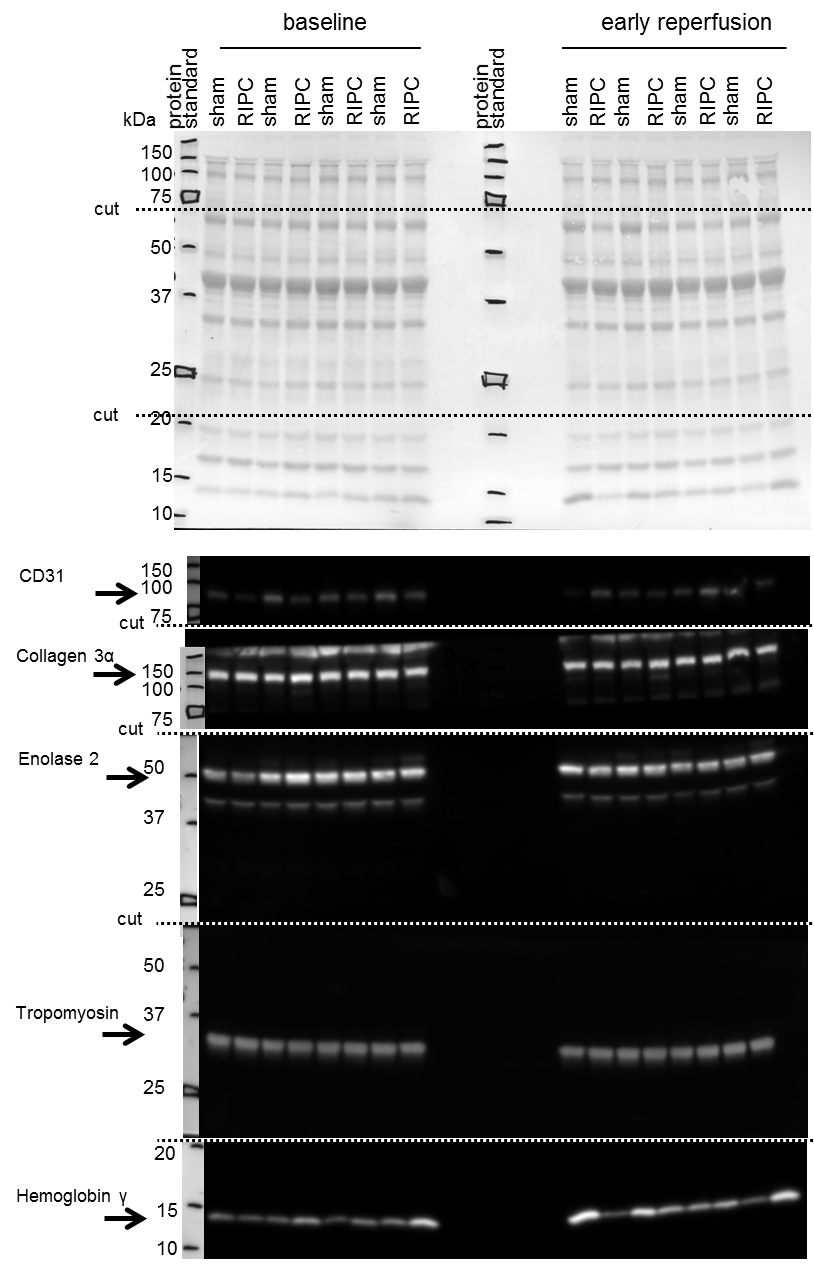


**Supplemental Figure S3. Western blot analysis of protein expressions of proteins characterizing different cell types in porcine left ventricular biopsies taken at baseline and at early reperfusion** **after coronary occlusion.** Ponceau-S staining and full-length blot. The expressions of CD1 (endothelial cell marker), collagen 3α (fibroblast marker), enolase 2 (neuronal marker), hemoglobin γ (erythrocyte marker) and tropomyosin (cardiomyocyte marker) were analyzed in porcine left ventricular biopsies taken at baseline and at early reperfusion after coronary occlusion with remote ischemic preconditioning (RIPC) or sham. The protein yields of the samples were limited, therefore membranes were cut as indicated to analyze the immunoreactivities of different proteins in parallel.

a


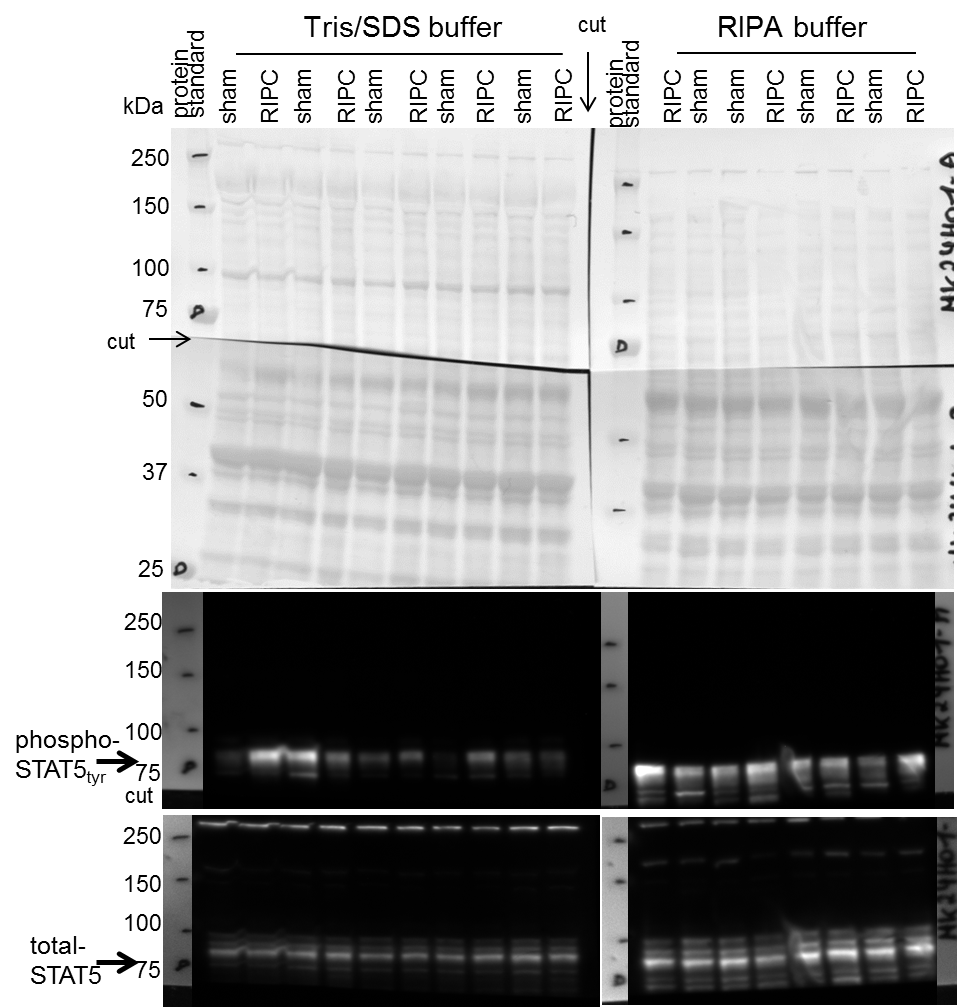


Supplemental Fig. S4 continued

b


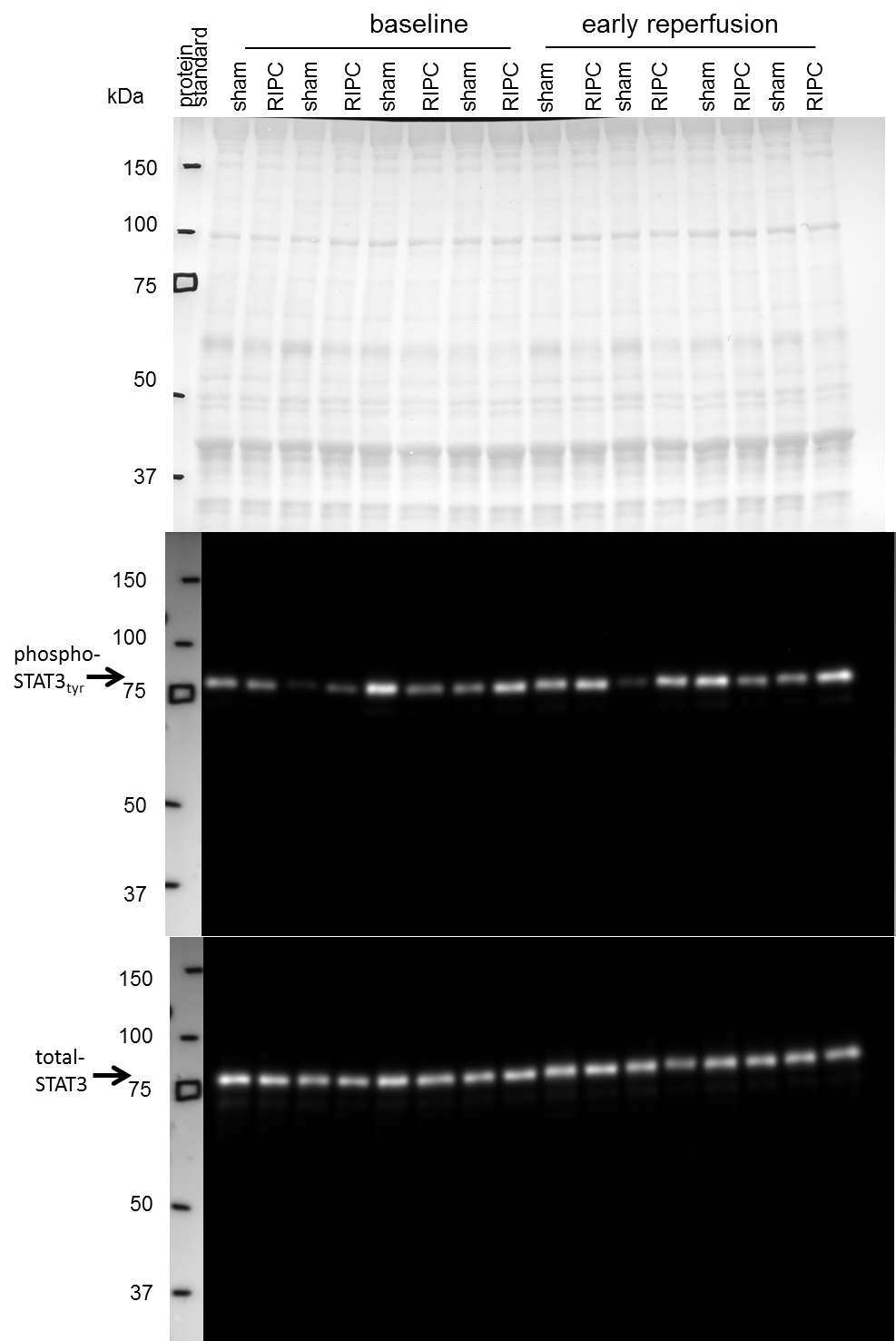


**Supplemental Figure 4. Western blot analysis of phosphorylation/expression of STAT5 in human and STAT3 in porcine left ventricular biopsies.** (a) Ponceau-S staining and full-length blot of phosphorylated and total signal transducer and activator of transcription 5 (STAT5). The phosphorylation/expression of STAT5 was analyzed in human left ventricular biopsies lysed in Tris/sodium dodecyl sulfate (SDS) or in radioimmunoprecipitation assay (RIPA) buffer with remote ischemic preconditioning (RIPC) or sham. (b) Ponceau-S staining and full-length blot of phosphorylated and total signal transducer and activator of transcription 3 (STAT3). The phosphorylation/expression of STAT3 was analyzed in porcine left ventricular biopsies taken at baseline and at early reperfusion after RIPC or sham.


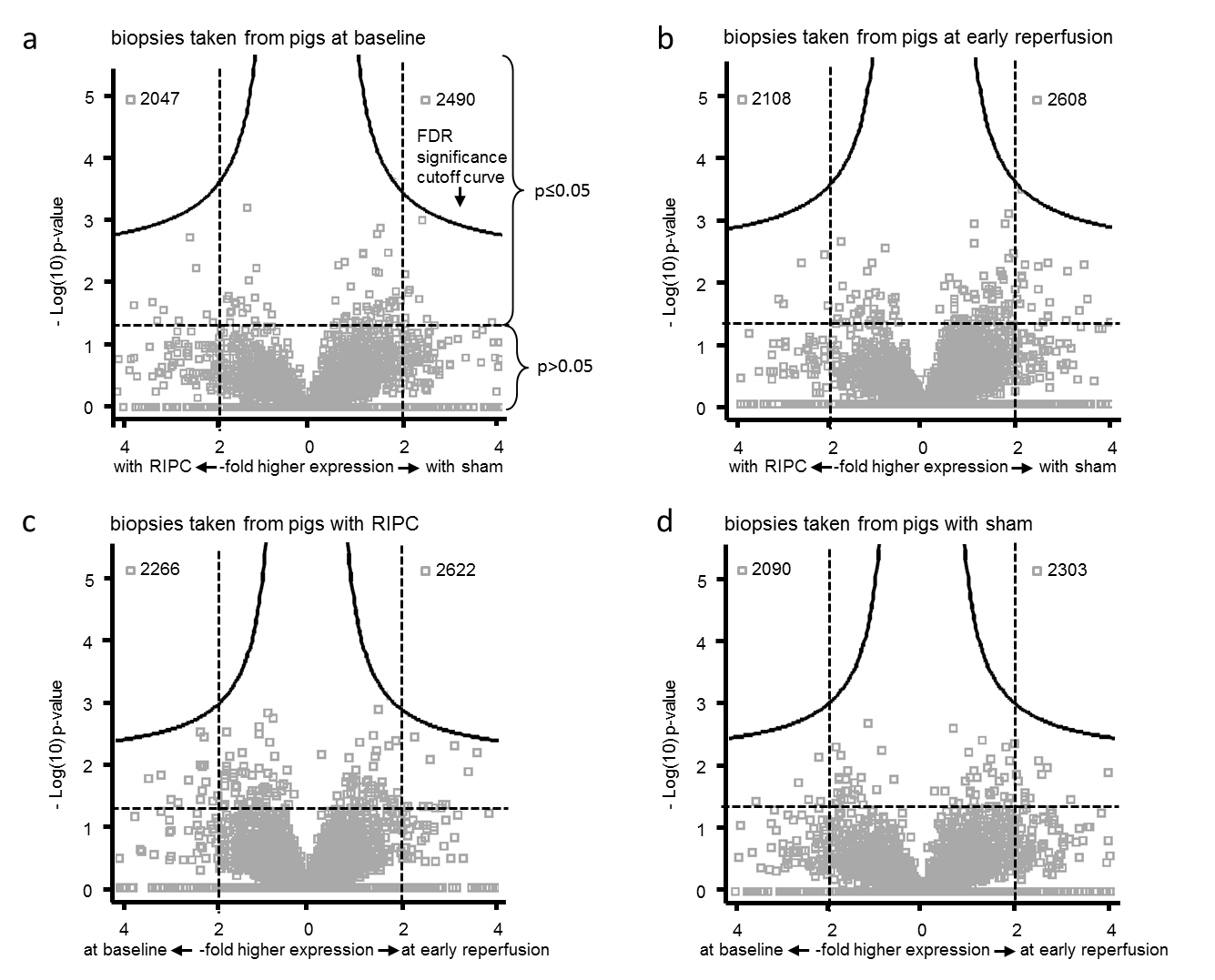


**Supplemental Figure S5: Vulcano plots of all detected proteins after in-solution digestion of porcine left ventricular biopsies.** Vulcano plots of -Log(10)p-value over -fold higher expression of all proteins between groups (remote ischemic preconditioning (RIPC)/sham) detected after in-solution digestion of porcine left ventricular biopsies taken (a) at baseline and (b) at early reperfusion. Vulcano plots of -Log(10)p-value over -fold higher expression of all proteins in the comparison between time points (baseline versus early reperfusion) (c) with remote ischemic preconditioning (RIPC) and (d) with sham. A Log(10)p-value of ≥1.3 corresponds to a p-value of ≤0.05. The false discovery rate (FDR) significance cut-off curve indicates no difference in protein expression between RIPC and sham and between baseline and early reperfusion with RIPC and with sham, respectively. Grey squares: proteins without FDR-based statistical difference between RIPC and sham or between baseline and early reperfusion.

**
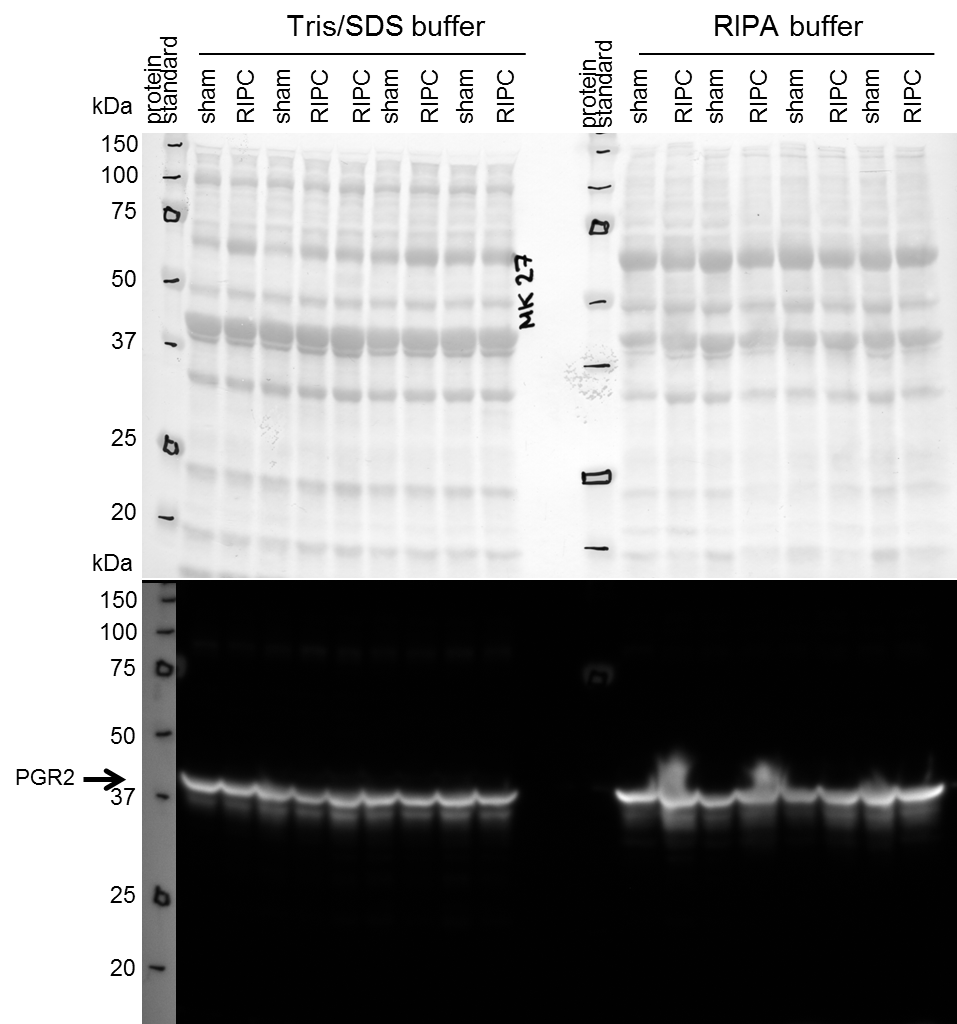
**

**Supplemental Figure S6. Western blot analysis of prostaglandin reductase 2 in human left ventricular biopsies taken at early reperfsuion.** Ponceau-S staining and full-length blot of prostaglandin reductase 2. The expression of prostaglandin reductase 2 (PGR2) was analyzed in human left ventricular biopsies lysed in Tris/sodium dodecyl sulfate (SDS) or radioimmunoprecipitation assay (RIPA) buffer after remote ischemic preconditioning (RIPC) or sham.

**
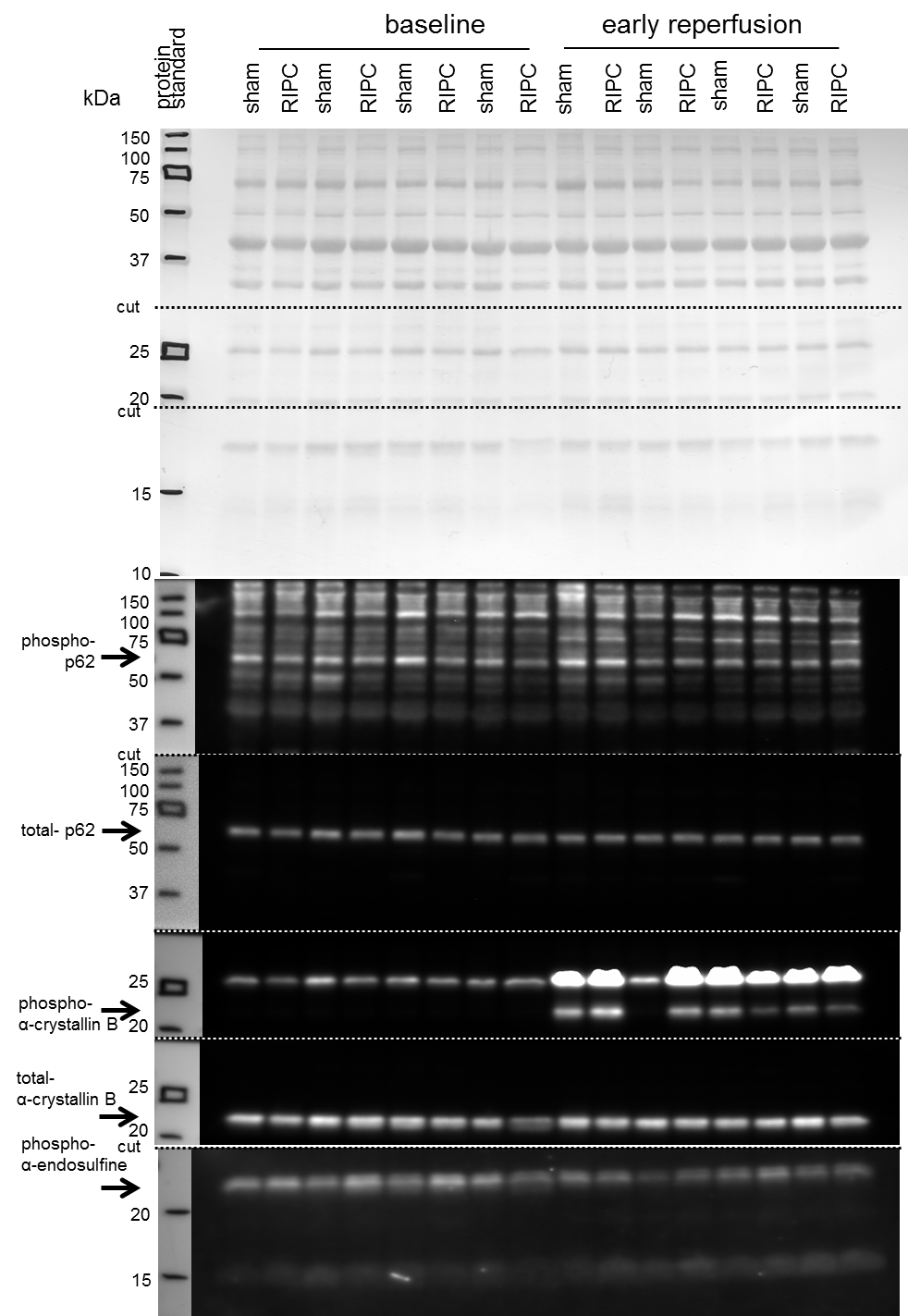
**

**Supplemental Figure S7. Western blot analysis of phosphorylation/expression of p62, α-crystallin B and phosphorylated α-endosulfine in porcine left ventricular biopsies taken at baseline and at early reperfsuion.** Ponceau-S staining and full-length blot of phosphorylated and total p62, α-crystallin B and phosphorylated α-endosulfine. The phosphorylation/expression was analyzed in porcine left ventricular biopsies taken at baseline and early reperfusion after remote ischemic preconditioning (RIPC) or sham. The protein yields of the samples were limited, therefore membranes were cut as indicated to analyze the immunoreactivities of different proteins in parallel.

**Supplemental Table S1: All proteins having ≥2-fold higher expression with remote ischemic preconditioning than with sham in human left ventricular biopsies** **at early reperfusion after cardioplegic ischemic arrest**

|  | . | -fold higher expression | p-value | protein ID | -fold higher expression | p-value | proteins exclusively detected with RIPC | | |
| --- | --- | --- | --- | --- | --- | --- | --- | --- | --- |
| Tris/SDS in-solution digestion | A8K541 | 3.14 | 0.348 | P02461 | 2.01 | 0.306 | B4DE93 | O75396 | P49321-4 |
|  | B4DT77 | 2.62 | 0.159 | P06396-2 | 2.95 | 0.032 | B7Z9L2 | O95782-2 | P49748-2 |
|  | B7Z254 | 2.97 | 0.005 | P07355 | 2.25 | 0.064 | D6RE05 | P01877 | P51452 |
|  | C9JUF0 | 2.71 | 0.011 | P22105-3 | 2.09 | 0.028 | E9PGT6 | P02794 | P56597 |
|  | E7ES19 | 2.36 | 0.107 | P35609 | 3.73 | 0.066 | E9PJ81 | P04114 | P63279 |
|  | E9PLG2 | 2.59 | 0.084 | P49593 | 2.15 | 0.052 | F8W9S7 | P07919 | Q08378-4 |
|  | F5H6X6 | 3.27 | 0.007 | P62158 | 2.98 | 0.067 | G3V2D6 | P08123 | Q14126 |
|  | F8VX58 | 2.04 | 0.076 | P63010-3 | 5.17 | 0.001 | G3XAJ6 | P08133-2 | Q5TBA9 |
|  | G3V3A0 | 2.92 | 0.371 | P63316 | 13.42 | 0.042 | G5E9Q4 | P08572 | Q6NUK1 |
|  | H7C1A5 | 3.17 | 0.025 | P68402-3 | 2.50 | 0.017 | H0YAW4 | P13639 | Q6P2E9 |
|  | K7EJE8 | 2.12 | 0.193 | Q13542 | 2.12 | 0.132 | I3L2I2 | P13667 | Q711Q0 |
|  | O00748-2 | 2.02 | 0.034 | Q5SV97 | 2.40 | 0.168 | I3L397 | P14854 | Q86UE4 |
|  | O15273 | 2.34 | 0.071 | Q5T457 | 2.01 | 0.315 | J3KSW8 | P19429 | Q86WV6 |
|  | O76041 | 2.08 | 0.314 | Q8N8N7 | 2.08 | 0.016 | M0QXC3 | P19652 | Q8NBF2-2 |
|  | O95810 | 2.79 | 0.064 | Q9BR39 | 2.55 | 0.006 | M0R1V7 | P25311 | Q8NE71-2 |
|  | P01859 | 2.41 | 0.016 | Q9BX66 | 2.07 | 0.124 | O00159-2 | P30050-2 | Q96AJ9-1 |
|  | P01876 | 2.59 | 0.010 | Q9H987 | 7.10 | 0.043 | O43707 | P31025 | Q99460-2 |
|  | P02452 | 5.04 | 0.067 |  |  |  | O43896 | P32119 | Q9BYV6-2 |
|  |  |  |  |  |  |  | O60313 | P46821 | Q9Y4X5 |
| in-gel digestion | A6NGJ0 | 2.424 | 0.118 | P55789 | 2.032 | 0.074 | A0PJW6 | P01621 | Q6Q759 |
|  | B4DJA5 | 2.125 | 0.232 | P60602 | 2.321 | 0.207 | A1A4S6 | P01781 | Q6UWY5 |
|  | C9J712 | 2.338 | 0.108 | P61254 | 3.138 | 0.065 | A6NGS4 | P05067-10 | Q70HW3-2 |
|  | E9PBP2 | 3.313 | 0.163 | P61916 | 2.584 | 0.120 | A8K2U0 | P05161 | Q7Z7L7 |
|  | E9PIC0 | 2.479 | 0.079 | P62070 | 2.228 | 0.139 | A8MT72 | P08195-3 | Q86TW2-2 |
|  | F6U1T9 | 2.447 | 0.064 | P62736 | 2.294 | 0.390 | B0V0T3 | P08246 | Q86U42-2 |
|  | F8W181 | 2.556 | 0.001 | P63208 | 2.042 | 0.010 | B1AJV7 | P09001 | Q86UP3 |
|  | O14561 | 2.200 | 0.089 | P63302 | 3.338 | 0.005 | B4DXV1 | P09132 | Q8IVS2 |
|  | O14949 | 2.210 | 0.054 | P69891 | 2.216 | 0.539 | B4DYF3 | P09234 | Q8N4P3-2 |
|  | O15239 | 3.078 | 0.122 | P80404 | 3.059 | 0.186 | B5ME91 | P14555 | Q8N4V1 |
|  | O43633 | 2.227 | 0.260 | P82909 | 2.005 | 0.121 | B7Z5J4 | P15289 | Q8NBM8 |
|  | O43677 | 4.328 | 0.083 | P84098 | 2.050 | 0.175 | B7Z6F8 | P16455 | Q8TAE8 |
|  | O60783 | 2.067 | 0.168 | P84243 | 2.090 | 0.026 | C9J4M6 | P16885 | Q8TE77 |
|  | O60869-2 | 2.069 | 0.067 | Q00765 | 2.056 | 0.164 | C9J4Z3 | P19388 | Q8WUK0 |
|  | O75223 | 2.103 | 0.316 | Q01995 | 2.223 | 0.105 | C9JA28 | P21127-12 | Q8WUX9 |
|  | O75380 | 2.057 | 0.209 | Q07020 | 3.632 | 0.003 | C9JAV2 | P26927 | Q92520 |
|  | O75891 | 2.543 | 0.098 | Q14714 | 2.410 | 0.328 | C9JX84 | P35244 | Q92820 |
|  | O95816 | 2.043 | 0.066 | Q15286 | 2.180 | 0.096 | D6RA88 | P42766 | Q92890 |
|  | O96033 | 2.104 | 0.050 | Q15813 | 2.302 | 0.120 | E7EQS3 | P49006 | Q92930 |
|  | P01040 | 2.532 | 0.263 | Q16778 | 2.943 | 0.099 | E7EUU1 | P51153 | Q96A49 |
|  | P02452 | 2.231 | 0.571 | Q2UY09 | 2.340 | 0.296 | E9PDS2 | P51580 | Q96ED9-2 |
|  | P03928 | 2.639 | 0.053 | Q3SYB4 | 2.008 | 0.296 | E9PEE1 | P51610-2 | Q96LD8 |
|  | P05089 | 2.762 | 0.483 | Q5VTU8 | 3.266 | 0.065 | E9PRV2 | P59768 | Q96NA2 |
|  | P05556 | 2.018 | 0.236 | Q5VXJ5 | 2.181 | 0.254 | F5GWH3 | P61421 | Q9BQ48 |
|  | P06748-2 | 2.001 | 0.008 | Q6Y1H2 | 2.537 | 0.220 | F5GWT4 | P62304 | Q9BTE1 |
|  | P07451 | 2.506 | 0.249 | Q6ZVX7 | 3.755 | 0.479 | F5GZC0 | P62312 | Q9BZL1 |
|  | P07476 | 2.057 | 0.633 | Q71UM5 | 2.965 | 0.084 | F5GZZ9 | P78524 | Q9C0D5-2 |
|  | P07602 | 2.266 | 0.087 | Q7LBR1 | 2.261 | 0.380 | F5H0B0 | Q01118 | Q9C0E2 |
|  | P07858 | 2.144 | 0.077 | Q8N5G0-2 | 2.904 | 0.039 | F5H0L8 | Q05707-2 | Q9H903-1 |
|  | P08514 | 2.558 | 0.237 | Q8N983-4 | 2.229 | 0.245 | F5H1E5 | Q08722-2 | Q9HBH1 |
|  | P09525 | 2.059 | 0.111 | Q8WUY1 | 2.746 | 0.012 | F5H2G5 | Q08752 | Q9HCU5 |
|  | P10176 | 3.369 | 0.198 | Q8WW22 | 2.009 | 0.069 | F5H5Y5 | Q0VGL1 | Q9HD26-3 |
|  | P10619 | 2.981 | 0.294 | Q8WWM9 | 2.558 | 0.187 | F5H711 | Q13030 | Q9NS18 |
|  | P11441 | 2.740 | 0.011 | Q96AQ8 | 2.894 | 0.072 | G3V4F7 | Q13445 | Q9NUP9 |
|  | P14406 | 2.155 | 0.404 | Q96C01 | 2.565 | 0.072 | H0YEL5 | Q13496 | Q9NUQ6-2 |
|  | P15104 | 2.238 | 0.524 | Q96HJ9 | 3.422 | 0.023 | H0YIV9 | Q13620-3 | Q9NUQ7 |
|  | P15954 | 3.468 | 0.169 | Q96MF6-2 | 2.047 | 0.162 | O00291 | Q13951 | Q9NV35 |
|  | P17900 | 2.894 | 0.287 | Q9BQJ4 | 2.103 | 0.147 | O00401 | Q14141-2 | Q9NXU5 |
|  | P18621 | 2.836 | 0.106 | Q9BTV4 | 2.223 | 0.031 | O14647-2 | Q14151 | Q9NYJ1 |
|  | P22735 | 5.796 | 0.431 | Q9BV57 | 2.015 | 0.026 | O14732-2 | Q15819 | Q9NZH8-2 |
|  | P24311 | 8.273 | 0.057 | Q9GZT6-2 | 2.052 | 0.153 | O43670-3 | Q3KQV9 | Q9P2F8 |
|  | P26373 | 2.861 | 0.094 | Q9H0X4 | 2.591 | 0.074 | O43684-2 | Q496Y1 | Q9UBJ2 |
|  | P27482 | 2.639 | 0.683 | Q9NR31 | 2.335 | 0.004 | O43766 | Q4VXY0 | Q9UIV8 |
|  | P31025 | 3.741 | 0.387 | Q9NZT1 | 3.243 | 0.386 | O43924 | Q53EU6 | Q9UJC5 |
|  | P31944 | 2.331 | 0.439 | Q9P0U1 | 5.485 | 0.112 | O60240 | Q53SF7-4 | Q9ULQ1 |
|  | P33316 | 2.561 | 0.334 | Q9UKL6 | 3.118 | 0.298 | O60437 | Q59GN2 | Q9UM00-2 |
|  | P34913 | 3.464 | 0.310 | Q9UMX0 | 2.047 | 0.215 | O60830 | Q5EBL4-3 | Q9UM54-5 |
|  | P40306 | 2.411 | 0.164 | Q9Y3B7 | 2.224 | 0.137 | O75165 | Q5EBL8 | Q9UP83-3 |
|  | P49327 | 3.285 | 0.198 | Q9Y3U8 | 2.131 | 0.444 | O75884 | Q5T6V5 | Q9Y282-2 |
|  | P51911 | 3.284 | 0.019 | Q9Y5J7 | 2.057 | 0.099 | O76070 | Q5VIR6-2 | Q9Y2V2 |
|  | P53992 | 2.297 | 0.002 | Q9Y5K6 | 2.175 | 0.333 | O94819 | Q5VSL9-2 | Q9Y2V7 |
|  | P55769 | 2.270 | 0.223 |  |  |  | O95163 | Q5VT66 | Q9Y3C4 |
|  |  |  |  |  |  |  | P01602 | Q5W111-2 |  |
| RIPA  in-solution digestion | E9PK80 | 2.23 | 0.019 |  |  |  | O60783 | Q9BQS8 | Q8N8S7-3 |
|  | J3KPV7 | 2.18 | 0.019 |  |  |  | P82675 | P07902 | Q9NUQ9 |
|  | O00159 | 2.15 | 0.149 |  |  |  | P21953 | R4GMU1 | Q8NCA5-2 |
|  | O14841 | 2.34 | 0.034 |  |  |  | P49406 | E9PP14 | Q9Y570 |
|  | O43772 | 3.02 | 0.054 |  |  |  | E7ESL0 | P11413 | H0YA52 |
|  | O75153 | 2.17 | 0.039 |  |  |  | Q9BRJ2 | P09488 | E5RG17 |
|  | O95782-2 | 3.14 | 0.016 |  |  |  | P62266 | F5H0U5 | O43143 |
|  | P00325 | 2.86 | 0.131 |  |  |  | H0YMV8 | P36959 | Q53FA7 |
|  | P00352 | 2.09 | 0.069 |  |  |  | Q00013 | F5H5I6 | B7Z7F3 |
|  | P02649 | 2.50 | 0.024 |  |  |  | K7ELC4 | Q14353 | Q7L523 |
|  | P05546 | 2.55 | 0.017 |  |  |  | J3KT73 | B1ANH5 | Q9HB90 |
|  | P07738 | 2.18 | 0.219 |  |  |  | B0UXB6 | Q9Y450 | Q6IQ22 |
|  | P12955 | 2.18 | 0.083 |  |  |  | Q9BPX5 | P52597 | Q969Q5 |
|  | P15374 | 2.06 | 0.088 |  |  |  | O95372 | B7Z4B8 | Q15493 |
|  | P16157-11 | 2.12 | 0.478 |  |  |  | P36404 | Q9BW72 | A8MT72 |
|  | P25311 | 2.06 | 0.098 |  |  |  | Q2M3C7-2 | P79483 | O95197-3 |
|  | P25787 | 2.01 | 0.083 |  |  |  | O00116 | Q29974 | H0YMG7 |
|  | P36269-2 | 2.05 | 0.167 |  |  |  | H3BV90 | P54868 | P09455 |
|  | P39059 | 2.13 | 0.074 |  |  |  | P03950 | P01613 | P49247 |
|  | P45974-2 | 2.06 | 0.134 |  |  |  | Q96CW1-2 | F5H4G7 | P51812 |
|  | P52758 | 2.06 | 0.008 |  |  |  | E5RJ68 | P53990-2 | O43464 |
|  | P52788 | 2.43 | 0.058 |  |  |  | O94911-3 | P29622 | E9PFR3 |
|  | P61626 | 2.18 | 0.197 |  |  |  | Q16740 | Q6YP21-3 | P62136 |
|  | P62701 | 2.47 | 0.071 |  |  |  | Q9BYV7-4 | Q9NRN7 | P50453 |
|  | P62805 | 2.23 | 0.105 |  |  |  | Q3LXA3 | Q16644 | P50454 |
|  | P69891 | 2.14 | 0.388 |  |  |  | O60502-2 | C9JS50 | P37108 |
|  | Q330K2-2 | 2.41 | 0.017 |  |  |  | F6U1T9 | B4DUX5 | Q9Y5X1 |
|  | Q58FF8 | 2.33 | 0.037 |  |  |  | Q6NUK1 | Q9NZJ7-2 | K7ENG2 |
|  | Q86YB7 | 2.04 | 0.012 |  |  |  | P31323 | Q9Y5J7 | O95721 |
|  | Q8N8N7 | 2.01 | 0.000 |  |  |  | G3V153 | Q9NZB8-7 | P61764 |
|  | Q92905 | 2.28 | 0.019 |  |  |  | P22748 | Q7Z406 | O00560 |
|  | Q96EY8 | 2.04 | 0.087 |  |  |  | E3W994 | Q5TEU4 | Q9UGI8-2 |
|  | Q96HY7 | 2.38 | 0.034 |  |  |  | Q96ER9 | P03886 | Q9H1E5 |
|  | Q99797 | 2.85 | 0.030 |  |  |  | Q9Y6G5 | Q9BXJ9 | Q8IYQ7 |
|  | Q9BRG1 | 2.44 | 0.059 |  |  |  | R4GN54 | Q9UMX5 | P20290-2 |
|  | Q9BU61 | 2.06 | 0.229 |  |  |  | P31146 | Q96TA1-2 | G8JLA8 |
|  | Q9BV79 | 2.26 | 0.002 |  |  |  | Q9NRP2 | Q96T66-2 | Q99598 |
|  | Q9NRG7-2 | 2.47 | 0.047 |  |  |  | P00395 | A8MXV4 | P54577 |
|  | Q9NVI7-2 | 2.19 | 0.029 |  |  |  | P24310 | Q9H6K4 | P62312 |
|  | Q9UDR5 | 2.56 | 0.029 |  |  |  | O75891 | Q9BXW6-2 | Q9UHD9 |
|  | Q9UJZ1 | 2.24 | 0.041 |  |  |  | Q9H773 | F5GZ78 | Q9Y4E8 |
|  | Q9Y6B6 | 2.02 | 0.052 |  |  |  | Q9UDY4 | P78356 | Q96K76 |
|  |  |  |  |  |  |  | Q9C0C9 | O60256 | Q9BSL1 |
|  |  |  |  |  |  |  | Q5T4S7-3 | Q96BW5-2 | H0Y9V2 |
|  |  |  |  |  |  |  | O94874 | B1ALH6 | P61086 |
|  |  |  |  |  |  |  | Q9BUP0 | Q9GZP4 | H0Y614 |
|  |  |  |  |  |  |  | Q96C19 | Q9HBL7 | Q05086-2 |
|  |  |  |  |  |  |  | Q96HE7 | E9PQY2 | G5E9I3 |
|  |  |  |  |  |  |  | Q9H0W9-2 | Q96I59 | Q709C8-4 |
|  |  |  |  |  |  |  | O75821 | Q9H3G5 | Q9UK41 |
|  |  |  |  |  |  |  | O75822 | Q9BUL8 | Q9UN37 |
|  |  |  |  |  |  |  | P14324-2 | I3NI21 | Q9H0V9 |
|  |  |  |  |  |  |  | Q9NYY8 | Q5QPM7 | H0YN81 |
|  |  |  |  |  |  |  | B4DXZ6 | Q9C005 | R4GN55 |
|  |  |  |  |  |  |  |  |  |  |

All proteins having ≥2-fold higher expression with remote ischemic preconditioning (RIPC) than with sham and those detected exclusively with RIPC in human left ventricular biopsies after in-solution digestion (after lysis in Tris/sodium dodecyl sulfate (Tris/SDS) buffer), in-gel digestion and in-solution digestion (after lysis in radioimmunoprecipitation assay (RIPA) buffer), respectively. When proteins were exclusively detected in samples with RIPC, -fold higher expression and p-value are not indicated. -fold higher expression and p-value versus sham. All proteins were compared by unpaired (between RIPC and sham) Student's t-tests.

**Supplemental Table S2: All proteins having ≥2-fold higher phosphorylation with remote ischemic preconditioning than with sham in human left ventricular biopsies** **at early reperfusion after cardioplegic ischemic arrest**

|  | protein ID | phosphorylation site | - fold higher phosphorylation | p-value | phosphopeptides exclusively detected with RIPC | |
| --- | --- | --- | --- | --- | --- | --- |
|  |  |  |  |  | protein ID | phosphorylation site |
| phopho-peptide enrichment | A4UGR9-2 | 1575;1622;1797 | 4.05 | 0.082 | A4UGR9-2 | 1580;1627;1627 |
|  | A4UGR9-2 | 2920;2967;2967;3142 | 2.15 | 0.357 | A4UGR9-2 | 2271;2318;2318;2493 |
|  | A6NNK5 | 1417;1462;1467 | 2.26 | 0.398 | A6NNK5 | 385;380 |
|  | A6NNK5 | 1415;1460;1465 | 2.26 | 0.398 | B4DS13 | 459;498;503 |
|  | B0QYF8 | 4 | 2.56 | 0.122 | B4DTZ6 | 354;693 |
|  | B1AHF3 | 123;124;146;180 | 2.22 | 0.242 | B4E1N6 | 406;500 |
|  | B4DGU4 | 184;191 | 3.02 | 0.199 | C9J453 | 459 |
|  | B4E2T8 | 446;554;589 | 6.77 | 0.132 | C9JXF9 | 194 |
|  | B4E2T8 | 475;583;618 | 6.30 | 0.242 | C9K0K7 | 227;83;141;187 |
|  | C9J813 | 202;196;207 | 7.68 | 0.168 | E7EVD1 | 570;619 |
|  | C9JJX6 | 573;579;636;642 | 2.31 | 0.041 | E7EW31 | 470;865;820 |
|  | D6RGZ6 | 1129;2116 | 2.31 | 0.345 | E9PAV3 | 916 |
|  | E7EPW4 | 233;234;236 | 2.08 | 0.090 | E9PDL6 | 296;75;113;285 |
|  | E7EV56 | 65 | 2.43 | 0.325 | E9PGF5 | 1114;1122;1135 |
|  | E9PGF5 | 338;463;406;114;369 | 2.41 | 0.420 | E9PI52 | 27 |
|  | E9PGF5 | 1156;1164;1177;315 | 3.57 | 0.261 | E9PJF4 | 102 |
|  | E9PMU9 | 139;154;163;296;325 | 2.15 | 0.206 | E9PMG1 | 143;129 |
|  | E9PNJ4 | 445;618;380 | 2.26 | 0.370 | E9PMS6 | 1017;1091 |
|  | E9PQX8 | 17;29;146 | 2.81 | 0.031 | E9PMS6 | 1016;1090 |
|  | F5GXU9 | 318;347;381;350 | 22.26 | 0.302 | E9PQA1 | 17 |
|  | F5GXU9 | 309;338;372;341 | 3.40 | 0.118 | E9PQA1 | 15 |
|  | F5GZ78 | 301;303;52 | 3.45 | 0.084 | F5GZ78 | 300;302;51 |
|  | F5H1N8 | 278;565;581;645 | 2.00 | 0.230 | F5GZP6 | 532 |
|  | H3BPX2 | 5740;4783;4783;5298 | 2.09 | 0.117 | F5H7R4 | 298;730;248 |
|  | H3BQZ7 | 161 | 2.17 | 0.147 | F8W845 | 552;655 |
|  | J3KTG7 | 208 | 2.61 | 0.412 | H0Y6F6 | 366;808;417 |
|  | K7EJ06 | 13 | 3.72 | 0.053 | H0YDJ3 | 432;838;852 |
|  | K7EQW8 | 58 | 2.03 | 0.565 | H0YFA4 | 98;114;188 |
|  | K7ERP6 | 239;332;339 | 6.32 | 0.011 | H0YGL9 | 1853;1869;1867 |
|  | K7ERP6 | 151;189;196;179 | 4.44 | 0.113 | H0YGL9 | 2792;2808;2806 |
|  | K7ERP6 | 153;191;198;181 | 3.21 | 0.128 | H0YGL9 | 4344;4360;4366 |
|  | M0QZ43 | 359;359 | 5.11 | 0.185 | H0YJ34 | 292;339;339;339 |
|  | O14558 | 16 | 4.40 | 0.200 | H0YL34 | 143;428;429;429 |
|  | O14579-2 | 152;203;226 | 2.08 | 0.265 | H3BQP2 | 31 |
|  | O15273 | 161;137 | 7.99 | 0.016 | J3QL89 | 59;596;605 |
|  | O43583 | 73 | 2.08 | 0.237 | J3QRP6 | 171;290;134 |
|  | O43719 | 616 | 3.23 | 0.220 | K7EK60 | 202;624 |
|  | O75112-4 | 129 | 2.69 | 0.143 | M0QZ43 | 142 |
|  | O75112-4;O75112-7 | 167 | 2.58 | 0.273 | M0QZ43 | 221;221 |
|  | O76041 | 953;175;209 | 5.78 | 0.240 | O00159-2 | 701;712;717;736 |
|  | O94875-10 | 344;69;165;63;22 | 5.88 | 0.136 | O14974-5 | 420;507;202;111 |
|  | O94875-10 | 741;562;921;1017 | 2.80 | 0.329 | O15273 | 39 |
|  | O94875-10 | 528;349;181;277;223 | 2.18 | 0.198 | O43150-2 | 701 |
|  | O94875-10 | 78 | 3.20 | 0.163 | O43847 | 94 |
|  | O94875-10 | 439;260;158;346;306 | 3.01 | 0.324 | O60237 | 687 |
|  | O95810 | 32 | 4.34 | 0.021 | O60240 | 382 |
|  | P01042-3 | 296;332 | 2.16 | 0.342 | O60343-3 | 672;672;704;315 |
|  | P04075 | 39;93 | 2.87 | 0.390 | O76041 | 949;171;205 |
|  | P04792 | 15 | 5.67 | 0.072 | O94811 | 160 |
|  | P04792 | 65 | 2.65 | 0.225 | O94979-3 | 799;794 |
|  | P07951 | 283 | 2.14 | 0.022 | O95365 | 525 |
|  | P07951 | 87;68;129;11 | 2.47 | 0.135 | O95365 | 526 |
|  | P08651-5 | 330;339;306 | 2.16 | 0.139 | O95425-3 | 288;315 |
|  | P12883 | 1269 | 2.77 | 0.068 | O95425-3 | 50 |
|  | P12883 | 619 | 2.96 | 0.146 | O95810 | 204 |
|  | P17540 | 319;318;349 | 2.18 | 0.153 | O95810 | 24 |
|  | P17661 | 68 | 4.42 | 0.260 | O95817 | 289;231 |
|  | P17661 | 32 | 3.65 | 0.177 | P00568 | 38;54 |
|  | P17661 | 28 | 3.62 | 0.147 | P02452 | 1125 |
|  | P21397 | 383;250 | 2.08 | 0.134 | P06732 | 337 |
|  | P23497-7 | 122;132;157 | 4.30 | 0.156 | P06732 | 24 |
|  | P26678 | 17 | 2.16 | 0.451 | P08559-3 | 264;295;302 |
|  | P26678 | 16 | 5.16 | 0.079 | Q8TCJ2 | 499 |
|  | P29966 | 150 | 2.17 | 0.189 | Q8WX93 | 641;641;641;259 |
|  | P32249 | 347 | 6.04 | 0.048 | Q8WZ42-3 | 4771 |
|  | P33527-8 | 815;871;874;839;930 | 2.29 | 0.191 | Q8WZ42-3 | 4570 |
|  | P35609 | 433;433 | 2.12 | 0.167 | Q8WZ42-3 | 24032;24157 |
|  | P46821 | 832 | 2.22 | 0.398 | Q8WZ42-6 | 19674;19799;19866 |
|  | P46937-5 | 61 | 2.28 | 0.191 | Q8WZ42-6 | 25779;25904 |
|  | P48681 | 1286 | 4.39 | 0.201 | Q969E4 | 65 |
|  | P54296 | 951;376 | 2.63 | 0.234 | Q96IZ0 | 230 |
|  | P55822 | 166;55 | 3.63 | 0.042 | Q96PE2 | 142 |
|  | P55822 | 169;58;58 | 2.51 | 0.073 | Q99442 | 155 |
|  | P60709 | 249;949;251;250 | 2.42 | 0.320 | Q99959-2 | 197;197 |
|  | P62070-2 | 109;151;186;192 | 2.16 | 0.120 | Q9BW62 | 415 |
|  | P68032;P68133 | 370;335;280;282 | 2.18 | 0.205 | Q9BX66 | 1051;1005 |
|  | P78536 | 791 | 3.35 | 0.047 | Q9BX66 | 1182;821 |
|  | Q02952 | 627;522;529 | 3.17 | 0.439 | Q9BX66-11 | 33 |
|  | Q02952 | 286;181;188 | 2.94 | 0.147 |  |  |
|  | Q09666 | 5782 | 2.85 | 0.043 | Q9C0C2 | 429 |
|  | Q13425 | 110 | 2.15 | 0.141 | Q9H987 | 111;42 |
|  | Q14315 | 2233;2200 | 2.66 | 0.308 | Q9H987 | 180 |
|  | Q14896 | 286 | 96.41 | 0.028 | Q9HBA9 | 30 |
|  | Q14896 | 602 | 2.36 | 0.121 | Q9HBU9 | 333 |
|  | Q2M3C7-2 | 1025 | 2.39 | 0.094 | Q9NZB2-4 | 383 |
|  | Q5BKX8 | 19 | 7.91 | 0.021 | Q9P2P5 | 48 |
|  | Q5BKX8 | 20 | 7.91 | 0.021 | Q9UHY1 | 433;441 |
|  | Q5HY54 | 2112;2144;2152;285 | 3.61 | 0.253 | Q9UK76-3 | 41;87 |
|  | Q5JSH3-2 | 50 | 2.02 | 0.019 | Q9UMS6 | 724;755;707 |
|  | Q5R372-2 | 82;119 | 5.42 | 0.089 | Q9UMS6 | 698;729;681 |
|  | Q5SV97 | 242;128 | 2.46 | 0.416 | Q9UPQ0 | 975;974;815 |
|  | Q5T457 | 72;99 | 5.57 | 0.001 | Q9Y2D5 | 778;867;1009 |
|  | Q5T6N2 | 556;654;338;263;526 | 2.03 | 0.452 | Q9Y4G6 | 2172;1086 |
|  | Q6P5Q4 | 392 | 2.27 | 0.486 | Q9Y608 | 328;96;168 |
|  | Q6QEF8-4 | 190;238;424 | 2.41 | 0.166 | Q9Y6I3-3 | 444;470;556 |
|  | Q6UXY1-2 | 479;493 | 8.41 | 0.039 | S4R303 | 326;257;317;194 |
|  | Q702N8 | 208 | 2.05 | 0.337 | P09651 | 6 |
|  | Q702N8 | 481 | 2.01 | 0.171 | P11055 | 949;952;950;954 |
|  | Q702N8 | 295 | 3.22 | 0.313 | P11055 | 1480;1483;1479 |
|  | Q702N8 | 138 | 2.63 | 0.258 | P11532-4 | 3492;3496 |
|  | Q86TV6 | 160;58 | 2.25 | 0.380 | P11532-4 | 3048 |
|  | Q86YP9 | 144;239;331;2746 | 2.51 | 0.200 | P12882 | 1144;1146 |
|  | Q8WZ42-12 | 25589;25714;25781 | 2.11 | 0.170 | P12883 | 215 |
|  | Q8WZ42-12 | 4585;4710;4777 | 2.57 | 0.098 | P12883 | 70;1140;1142 |
|  | Q8WZ42-3 | 25629;25754;25821 | 3.45 | 0.015 | P12883 | 1470;1472;1630;1632 |
|  | Q8WZ42-3 | 7237;6828;6920 | 5.03 | 0.062 | P13224 | 191;396 |
|  | Q8WZ42-3 | 25634;25759 | 7.05 | 0.109 | P15924 | 2024;1425;1581 |
|  | Q8WZ42-3 | 3497 | 2.23 | 0.246 | P17302 | 325;250 |
|  | Q8WZ42-3 | 3761;3886;3953 | 3.18 | 0.262 | P17540 | 142;151 |
|  | Q8WZ42-4 | 26190;26315 | 2.44 | 0.375 | P28482-2 | 187 |
|  | Q8WZ42-6 | 4111 | 2.12 | 0.031 | P35609 | 179 |
|  | Q8WZ42-6 | 10638;10229 | 2.27 | 0.194 | P41208 | 20 |
|  | Q92614-2 | 1611;1942 | 4.65 | 0.117 | P46527 | 10;10 |
|  | Q96CX2 | 187 | 2.37 | 0.215 | P48681 | 934 |
|  | Q96R28 | 132 | 2.71 | 0.205 | P48681 | 842 |
|  | Q9BUB1 | 80 | 2.38 | 0.147 | P52179 | 58 |
|  | Q9BUB1 | 99 | 2.51 | 0.200 | P53985 | 467 |
|  | Q9BX66 | 945;967;899;796;915 | 2.39 | 0.281 | P54296 | 59 |
|  | Q9GZT3-2 | 100;102 | 2.90 | 0.157 | P54296 | 1046;471 |
|  | Q9H1E3 | 223;183 | 2.22 | 0.448 | P61978 | 116;47 |
|  | Q9H1E5 | 251 | 2.89 | 0.212 | Q01082 | 2107;2107;2094 |
|  | Q9H3Z4-2 | 10 | 4.95 | 0.186 | Q06190-2 | 71;692 |
|  | Q9H987 | 427;203 | 2.60 | 0.075 | Q07002 | 72 |
|  | Q9H987 | 400;176 | 2.55 | 0.063 | Q07666-3 | 18 |
|  | Q9H987 | 891;667 | 3.54 | 0.257 | Q08170 | 431 |
|  | Q9H987 | 892;668 | 4.32 | 0.296 | Q09666 | 212 |
|  | Q9HBU9 | 361;22 | 2.41 | 0.430 | Q12802-4 | 2391;2411;2415;656 |
|  | Q9NPC6 | 116 | 3.71 | 0.069 | Q12802-4 | 1912;1930;1934;175 |
|  | Q9UPQ0 | 469;469;310;469 | 2.81 | 0.079 | Q13409-6 | 101;95;113 |
|  | Q9Y2D5 | 720;809;809;951 | 2.61 | 0.124 | Q13554 | 395;152;266;332 |
|  | Q9Y2X3 | 502 | 3.10 | 0.201 | Q14247-3 | 364;401;364 |
|  | Q9Y5S2 | 1690 | 2.47 | 0.203 | Q15149-7 | 878;888;896 |
|  |  |  |  |  | Q15772-1 | 453;453;349 |
|  |  |  |  |  | Q15811-6 | 973;936;941 |
|  |  |  |  |  | Q2M3C7-2 | 1485;1485 |
|  |  |  |  |  | Q2V2M9-4 | 366;366;366;165;366 |
|  |  |  |  |  | Q53EL6-2 | 446;457 |
|  |  |  |  |  | Q53GG5-2 | 170 |
|  |  |  |  |  | Q53T59 | 249;42;68 |
|  |  |  |  |  | Q53TN4-3 | 190;248 |
|  |  |  |  |  | Q5BKX8 | 302 |
|  |  |  |  |  | Q5QNZ2 | 168;229 |
|  |  |  |  |  | Q5STZ8 | 109;108;11 |
|  |  |  |  |  | Q5VST9-3 | 6345;3464 |
|  |  |  |  |  | Q68CZ2-2 | 540;883;780 |
|  |  |  |  |  | Q702N8 | 1737;420 |
|  |  |  |  |  | Q702N8 | 465 |
|  |  |  |  |  | Q7L8J4 | 358;326 |
|  |  |  |  |  | Q7RTP6 | 977;8 |
|  |  |  |  |  | Q7Z4V5-2 | 630;630;59;144;126 |
|  |  |  |  |  | Q7Z7B0-2 | 138;138 |
|  |  |  |  |  | Q86TI0-2 | 5;5;5;5 |
|  |  |  |  |  | Q86W92-2 | 371;387;509;540 |
|  |  |  |  |  | Q8IUG5 | 112 |
|  |  |  |  |  | Q8N1G4 | 518 |
|  |  |  |  |  | Q8N3K9 | 2686 |
|  |  |  |  |  | Q8NBV4 | 62 |
|  |  |  |  |  | Q8NI27-2 | 238;1417;153;6;13 |
|  |  |  |  |  |  |  |

All proteins having ≥2-fold higher phosphorylation with remote ischemic preconditioning (RIPC) than with sham and those detected exclusively with RIPC in human left ventricular biopsies after phosphopeptide enrichment. When proteins were exclusively detected in samples with RIPC, -fold higher phosphorylation and p-value are not indicated, -fold higher phosphorylation and p-value versus sham. All proteins were compared by unpaired (between RIPC and sham) Student's t-tests.

**Supplemental Table S3: All proteins having ≥2-fold higher expression with sham than with remote ischemic preconditioning in human left ventricular biopsies at early reperfusion after cardioplegic ischemic arrest**

|  | protein ID | -fold higher expression | p-value | protein ID | -fold higher expression | p-value | proteins exclusively detected with sham | |
| --- | --- | --- | --- | --- | --- | --- | --- | --- |
| Tris/SDS  in-solution digestion | A4UGR9-2 | 3.72 | 0.004 | O60237 | 2.03 | 0.059 | P08133-2 | Q9NUJ1 |
|  | A6NC17 | 2.07 | 0.018 | P02788-2 | 2.60 | 0.038 | Q92499 | H0Y9M8 |
|  | B4DLA0 | 3.11 | 0.117 | P12235 | 3.07 | 0.281 | K7EIE5 | C9JD73 |
|  | B4DTA3 | 2.08 | 0.023 | P21796 | 2.07 | 0.227 | P50461 | P55735-2 |
|  | B7Z6B8 | 2.62 | 0.001 | P30086 | 2.49 | 0.024 | K7ESM3 | Q68EM7-7 |
|  | E9PG83 | 3.10 | 0.032 | P42765 | 2.36 | 0.251 | D6RE05 | Q96FV2 |
|  | G3V1U3 | 2.33 | 0.013 | P68133 | 2.41 | 0.174 | P12882 |  |
|  | G8JLH9 | 3.88 | 0.005 | Q14BN4-5 | 3.03 | 0.189 | P15259 |  |
|  | H0YNN4 | 2.12 | 0.002 | Q5VTT5 | 3.54 | 0.129 | Q5JXI8 |  |
|  | J3QL77 | 4.61 | 0.018 | Q96HC4-6 | 2.01 | 0.153 | Q92538 |  |
|  | O43583 | 2.42 | 0.000 | Q9UBY9 | 4.19 | 0.026 | P0CG06 |  |
|  | O43678 | 2.56 | 0.020 |  |  |  |  |  |
| in-gel digestion | A5YM72 | 2.23 | 0.287 | Q13813-3 | 2.01 | 0.001 | A2A2Y4-4 | Q06033-2 |
|  | A6NNS2 | 3.02 | 0.148 | Q14019 | 2.30 | 0.390 | A8K2U1 | Q13315 |
|  | A8MZF9 | 3.57 | 0.057 | Q14165 | 2.04 | 0.063 | A8MT72 | Q13813 |
|  | C9J8Z4 | 2.38 | 0.364 | Q14393-3 | 2.10 | 0.175 | B4DGZ6 | Q15042 |
|  | D6RBB6 | 2.02 | 0.038 | Q15477 | 2.05 | 0.072 | B4DNI9 | Q15058 |
|  | D6RD17 | 2.06 | 0.239 | Q562R1 | 3.41 | 0.401 | B4DUD1 | Q15102 |
|  | E7EQB2 | 2.60 | 0.073 | Q5T8I0 | 3.43 | 0.200 | B4E1N2 | Q15274 |
|  | E9PBV3 | 6.17 | 0.150 | Q5TA50 | 2.74 | 0.185 | B8ZZF8 | Q15843 |
|  | E9PH06 | 2.19 | 0.290 | Q5TFE4 | 2.30 | 0.048 | B8ZZQ6 | Q16222-2 |
|  | F5H816 | 4.16 | 0.552 | Q5XKP0 | 2.24 | 0.074 | C9IY40 | Q29974 |
|  | H0YBG0 | 7.34 | 0.152 | Q63HQ2-2 | 2.25 | 0.074 | D3DQ30 | Q59GT6 |
|  | O43264 | 2.22 | 0.016 | Q6ICL3 | 2.23 | 0.187 | D3DR31 | Q5T0N5-3 |
|  | O60888-3 | 2.78 | 0.028 | Q71DI3 | 11.47 | 0.277 | D6R9H7 | Q5THU2 |
|  | O75368 | 2.05 | 0.046 | Q7L5D6 | 2.24 | 0.166 | D6RCD0 | Q6DKK2 |
|  | P01766 | 2.08 | 0.174 | Q7Z2W9 | 2.49 | 0.140 | E5RH64 | Q6P161 |
|  | P01877 | 2.40 | 0.667 | Q7Z3D6 | 13.95 | 0.408 | E7EMI0 | Q6P2Q9 |
|  | P02656 | 2.99 | 0.034 | Q8N3E9 | 2.45 | 0.004 | E9PB95 | Q7Z7H5-3 |
|  | P02792 | 2.53 | 0.450 | Q8NF91-4 | 2.61 | 0.041 | E9PC52 | Q86UP3 |
|  | P04839 | 2.15 | 0.475 | Q92626 | 2.32 | 0.435 | E9PF24 | Q8IVP5 |
|  | P05164-2 | 2.85 | 0.104 | Q92882 | 2.00 | 0.354 | E9PFH4 | Q8N0U4 |
|  | P05386 | 4.90 | 0.045 | Q93077 | 2.33 | 0.502 | F5H669 | Q8N2H3 |
|  | P09471-2 | 2.24 | 0.064 | Q96B49 | 3.41 | 0.190 | F5H871 | Q8NBM8 |
|  | P10636 | 2.49 | 0.253 | Q96B96 | 3.84 | 0.077 | H0Y9V2 | Q8NHV1 |
|  | P12273 | 2.73 | 0.555 | Q96BW5-2 | 2.60 | 0.017 | H0YH87 | Q8WUP2-3 |
|  | P12829 | 2.41 | 0.048 | Q96DA0 | 668.99 | 0.057 | O15305 | Q92817 |
|  | P13796 | 3.52 | 0.295 | Q99426 | 2.07 | 0.037 | O60234 | Q92934 |
|  | P16104 | 2.39 | 0.037 | Q99439 | 5.43 | 0.094 | O75600 | Q95604 |
|  | P16401 | 2.25 | 0.385 | Q99541 | 3.45 | 0.091 | O96019-2 | Q96CT7 |
|  | P19021-6 | 2.27 | 0.079 | Q9BW72 | 2.04 | 0.182 | P00746 | Q96L96 |
|  | P24158 | 7.16 | 0.317 | Q9BYV7-4 | 2.01 | 0.101 | P01611 | Q96Q89-4 |
|  | P26232-2 | 12.54 | 0.167 | Q9GZY4 | 2.11 | 0.366 | P01717 | Q96RQ1 |
|  | P30622 | 2.00 | 0.156 | Q9H446 | 2.44 | 0.209 | P08195-3 | Q96S82 |
|  | P31146 | 2.32 | 0.384 | Q9H6F2 | 2.09 | 0.085 | P08246 | Q9BQS8 |
|  | P41567 | 2.35 | 0.095 | Q9NP58-4 | 2.72 | 0.118 | P13760 | Q9BRX2 |
|  | P50135 | 2.17 | 0.111 | Q9NQ88 | 2.08 | 0.074 | P15927 | Q9C0B1 |
|  | P54725 | 2.24 | 0.195 | Q9NWS8 | 2.59 | 0.179 | P19387 | Q9H2P9-3 |
|  | P54868 | 2.25 | 0.386 | Q9NXE4-2 | 2.02 | 0.136 | P19623 | Q9H3Z4 |
|  | P55083 | 2.28 | 0.102 | Q9NZ08 | 2.13 | 0.008 | P31321 | Q9HC77 |
|  | P55263 | 2.12 | 0.089 | Q9UBV2 | 4.99 | 0.016 | P48729 | Q9NRF8 |
|  | P61326 | 2.01 | 0.099 | Q9UHY7 | 2.07 | 0.059 | P49407-2 | Q9NS18 |
|  | P61626 | 2.79 | 0.493 | Q9UL46 | 2.12 | 0.016 | P49913 | Q9NSK0 |
|  | P62328 | 55.96 | 0.277 | Q9Y2P4 | 3.37 | 0.079 | P53609 | Q9NSK7-3 |
|  | P78559 | 2.18 | 0.242 | Q9Y333 | 2.13 | 0.082 | P58107 | Q9NV35 |
|  | Q00G26 | 2.06 | 0.091 | Q9Y3D5 | 2.69 | 0.072 | P80511 | Q9NZ43 |
|  | Q13509 | 2.82 | 0.233 | Q9Y6N1 | 2.84 | 0.470 | Q06136 | Q9T9Y2 |
|  |  |  |  |  |  |  | Q06787-8 | Q9Y295 |
|  |  |  |  |  |  |  | Q08379-2 | Q9Y3C4 |
| RIPA in-solution digestion | H0Y7A7 | 2.05 | 0.012 | P49207 | 2.26 | 0.297 | Q13030 | Q9Y3D0 |
|  | H7C5B8 | 2.30 | 0.182 | P61224-3 | 2.58 | 0.080 | K7EJV5 | Q8WVI7 |
|  | O95425-4 | 2.06 | 0.132 | Q05707-2 | 2.82 | 0.316 | Q8WW22 | J3QL05 |
|  | P04264 | 2.68 | 0.082 | Q16363-2 | 2.67 | 0.088 | P50440-3 | P50454 |
|  | P07996 | 3.86 | 0.110 | Q9H987 | 2.57 | 0.069 | Q9UJY1 |  |
|  | P35443 | 2.15 | 0.267 | Q9HD34 | 2.01 | 0.041 | P13746 |  |

All proteins having ≥2-fold higher expression with sham than with remote ischemic preconditioning (RIPC) and those detected exclusively with sham in human left ventricular biopsies after in-solution digestion (after lysis in Tris/sodium dodecyl sulfate (Tris/SDS) buffer), in-gel digestion and in-solution digestion (after lysis in radioimmunoprecipitation assay (RIPA) buffer), respectively. When proteins were exclusively detected in samples with sham, -fold higher expression and p-value are not indicated, -fold higher expression and p-value versus RIPC. All proteins were compared by unpaired (between RIPC and sham) Student's t-tests.

**Supplemental Table S4: All proteins having ≥2-fold higher phosphorylation with sham than with remote ischemic preconditioning in human left ventricular biopsies** **at early reperfusion after cardioplegic ischemic arrest**

|  | protein ID | phosphorylation site | -fold higher phosphorylation | p-value | phosphopeptides exclusively detected with sham | |
| --- | --- | --- | --- | --- | --- | --- |
|  |  |  |  |  | protein ID | phosphorylation site |
| phopho-peptide enrichment | B4DJ84 | 407;193;400 | 2.31 | 0.199 | A8K541 | 444;501;468 |
|  | B4DR80 | 4 | 2.13 | 0.157 | B4DHJ7 | 93 |
|  | B7Z7A9 | 48;76 | 2.52 | 0.083 | B4DS13 | 465;504;509 |
|  | D6REM6 | 195 | 3.03 | 0.188 | B7Z4M2 | 256;277 |
|  | E9PAV3 | 855 | 4.57 | 0.059 | E7EMN6 | 95;121 |
|  | E9PAV3 | 802 | 2.22 | 0.169 | E7EMZ9 | 1902;1947 |
|  | E9PGF5 | 907;66 | 2.10 | 0.358 | E7EW31 | 236 |
|  | E9PHI6 | 394;510 | 2.17 | 0.038 | E9PAV3 | 1620 |
|  | E9PN66 | 16 | 4.31 | 0.002 | E9PGF5 | 1118;1126 |
|  | E9PN66 | 14 | 2.05 | 0.154 | E9PK52 | 645;715;167 |
|  | F5GY11 | 238;255;284 | 2.82 | 0.116 | F8W781 | 164;1208 |
|  | F5H1N8 | 647;663;727 | 3.95 | 0.087 | H0Y9H2 | 61;355 |
|  | F5H1N8 | 573;589;653 | 3.66 | 0.363 | H0YAH0 | 664;974 |
|  | G8JL88 | 293;311;327 | 2.33 | 0.129 | H0YIR4 | 18;146;176 |
|  | H0YL34 | 758;1043;1044 | 2.08 | 0.144 | H0YL34 | 143;428;429 |
|  | H3BM18 | 106;110 | 2.61 | 0.309 | J3QLA8 | 123;99;110 |
|  | H3BTK3 | 30 | 6.99 | 0.001 | M0QZ43 | 358 |
|  | I3L4J1 | 121;97 | 2.19 | 0.159 | M0QZ43 | 356 |
|  | K7EQH1 | 66 | 2.01 | 0.220 | M0R201 | 126;853;552 |
|  | M0R116 | 217;228 | 2.43 | 0.150 | O00206-3 | 241;401;441 |
|  | O43674-2 | 170;182;199 | 2.90 | 0.222 | O00505 | 56 |
|  | O43719 | 702 | 2.97 | 0.125 | O14974-5 | 609;696 |
|  | O75112-4 | 173 | 8.30 | 0.116 | O43896 | 1033 |
|  | O75112-4 | 135 | 2.35 | 0.603 | O60237 | 646 |
|  | O75475 | 106 | 2.23 | 0.128 | O60240 | 382 |
|  | O75643-2 | 225 | 2.10 | 0.251 | O75190-3 | 315 |
|  | O94875-10 | 420;390 | 2.29 | 0.269 | O76024 | 32 |
|  | O94875-2 | 9 | 29.04 | 0.003 | O94804 | 438 |
|  | P02545 | 390;291 | 5.44 | 0.328 | O94811 | 160 |
|  | P02765 | 330;331 | 2.20 | 0.202 | O94875-10 | 415;236;134 |
|  | P04406-2 | 168;210;135 | 4.27 | 0.024 | O95218-2 | 153 |
|  | P04406-2 | 169;211;136 | 4.27 | 0.024 | O95810 | 203 |
|  | P04792 | 82 | 3.46 | 0.611 | P00338-4 | 252;310;339 |
|  | P05060 | 130 | 2.80 | 0.251 | P04114 | 4048 |
|  | P10636 | 519;537;173 | 3.00 | 0.161 | P06396-2 | 511;519 |
|  | P12883 | 738 | 2.43 | 0.272 | P06732 | 327 |
|  | P12883 | 1019 | 2.01 | 0.439 | P06733 | 419;326 |
|  | P16403 | 2 | 2.29 | 0.228 | P11055 | 949;952 |
|  | P18206-2 | 290 | 2.44 | 0.397 | P16066 | 395 |
|  | P31629 | 2364 | 7.68 | 0.223 | P17661 | 432 |
|  | P33241 | 252;190;243 | 2.19 | 0.180 | P46019 | 729 |
|  | P78559 | 504;742 | 4.23 | 0.107 | P46527 | 10 |
|  | Q07157 | 622;626 | 2.21 | 0.082 | P46937-5 | 131 |
|  | Q14896 | 423;424 | 3.46 | 0.031 | P52594-2 | 181 |
|  | Q2M3C7-2 | 1220 | 2.55 | 0.039 | P53999 | 17 |
|  | Q5T6N2 | 423;483;167 | 2.49 | 0.256 | P54296 | 1461;886 |
|  | Q5T6N2 | 156;216 | 2.16 | 0.296 | P60174-1 | 195;232;113 |
|  | Q702N8 | 205 | 33.89 | 0.285 | P60709 | 53 |
|  | Q8NAN2-2 | 44 | 2.50 | 0.360 | P82909 | 61 |
|  | Q8WX93 | 766;384;55 | 2.61 | 0.159 | Q02952 | 1587;1482 |
|  | Q8WZ42-3 | 729;729;775 | 2.41 | 0.459 | Q13554 | 463 |
|  | Q8WZ42-6 | 4142 | 2.35 | 0.187 | Q13813 | 1291;1291 |
|  | Q96CX2 | 183 | 2.26 | 0.168 | Q14515 | 59 |
|  | Q99959-2 | 294 | 4.26 | 0.222 | Q15811-6 | 973;936;941 |
|  | Q9BX66 | 242;173;233 | 2.66 | 0.031 | Q2V2M9-4 | 438 |
|  | Q9BX66 | 86 | 3.24 | 0.131 | Q4L180-2 | 797;551;551 |
|  | Q9H1E3 | 58 | 2.59 | 0.006 | Q5BKX8 | 336 |
|  | Q9H1E3 | 61 | 3.40 | 0.035 | Q5JSH3-2 | 7 |
|  | Q9H1E3 | 79;39 | 2.49 | 0.129 | Q5U651 | 419 |
|  | Q9H1E3 | 19 | 2.75 | 0.276 | Q6UX71-3 | 128;457;506 |
|  | Q9H1E3 | 75;35 | 2.39 | 0.337 | Q702N8 | 134 |
|  | Q9H307 | 347;214 | 2.15 | 0.096 | Q7L8J4 | 358;326 |
|  | Q9NQC3 | 12 | 2.02 | 0.472 | Q7RTP6 | 685;204;685 |
|  | Q9UI47 | 650 | 3.68 | 0.109 | Q86TC9 | 818;543 |
|  | Q9UKA4 | 1242 | 2.45 | 0.246 | Q8N3L3 | 549 |
|  | Q9UMS6 | 637 | 2.72 | 0.004 | Q8NBV4 | 62 |
|  | Q9UMS6 | 195;226;178 | 3.43 | 0.025 | Q8TCJ2 | 498 |
|  | Q9UMS6 | 746;777;729 | 2.21 | 0.247 | Q8WZ42-3 | 4217;4342;4409 |
|  | Q9UPQ0 | 995 | 3.29 | 0.017 | Q96CX2 | 176 |
|  | Q9UQ35 | 1083 | 2.00 | 0.406 | Q9BUB1 | 104;104 |
|  | Q9Y2M0 | 692 | 2.11 | 0.054 | Q9BX66 | 326 |
|  |  |  |  |  | Q9BX66 | 1051 |
|  |  |  |  |  | Q9BX66-11 | 1180;819 |
|  |  |  |  |  | Q9BX66-3 | 491 |
|  |  |  |  |  | Q9C0D5-2 | 341;447 |
|  |  |  |  |  | Q9H4A3-4 | 1605;1764 |
|  |  |  |  |  | Q9H987 | 111;42 |
|  |  |  |  |  | Q9H987 | 95;26 |
|  |  |  |  |  | Q9HBA9 | 30 |
|  |  |  |  |  | Q9P225-2 | 2893 |
|  |  |  |  |  | Q9P2P5 | 48 |
|  |  |  |  |  | Q9UIG0-2 | 1464;1468 |
|  |  |  |  |  | Q9UMS6 | 224;193;224;176 |
|  |  |  |  |  | Q9UQ35 | 1318 |
|  |  |  |  |  | Q9Y478 | 108 |

All proteins having ≥2-fold higher phosphorylation with sham than with remote ischemic preconditioning (RIPC) and those detected exclusively with sham in human left ventricular biopsies after phosphopeptide enrichment. When proteins were exclusively detected in samples with RIPC, -fold higher phosphorylation and p-value are not indicated, -fold higher phosphorylation and p-value versus sham. All proteins were compared by unpaired (between RIPC and sham) Student's t-tests.

**Supplemental Table S5: All proteins having ≥2-fold higher expression between groups (remote ischemic preconditioning/sham) at baseline and at eary reperfusion or between time points (baseline/early reperfusion) with remote ischemic preconditioning or with sham**

|  | increased expression with | protein ID | -fold higher expression | p-value | protein ID | -fold higher expression | p-value | proteins exclusively detected in one group or at one time point | | |
| --- | --- | --- | --- | --- | --- | --- | --- | --- | --- | --- |
| baseline | RIPC | A6NKH3 | 2.79 | 0.219 | [P05230](http://www.uniprot.org/uniprot/P05165) | 2.11 | 0.132 | [A8K787](http://www.uniprot.org/uniprot/Q4G0W2) | I3LQ54 | [P82921](http://www.uniprot.org/uniprot/Q9NZB2) |
|  |  | [A7E2Y1](http://www.uniprot.org/uniprot/P35237) | 4.49 | 0.250 | [P06576](http://www.uniprot.org/uniprot/Q5TGZ0) | 2.43 | 0.062 | [B2MUB8](http://www.uniprot.org/uniprot/P07951) | I3LS97 | P82979 |
|  |  | [A8MXV4](http://www.uniprot.org/uniprot/Q8WZ42) | 2.91 | 0.151 | [P07195](http://www.uniprot.org/uniprot/O95716) | 2.89 | 0.100 | [B4DSZ2](http://www.uniprot.org/uniprot/P04406) | I3LU60 | [Q02153](http://www.uniprot.org/uniprot/Q9UKX3) |
|  |  | [D2HEV8](http://www.uniprot.org/uniprot/B4DRU9) | 2.35 | 0.280 | [P07919](http://www.uniprot.org/uniprot/P10636) | 2.39 | 0.289 | [D2H042](http://www.uniprot.org/uniprot/P61960) | I3MPD8 | [Q02978](http://www.uniprot.org/uniprot/Q3MHD2) |
|  |  | [E2RNN6](http://www.uniprot.org/uniprot/P49407) | 2.29 | 0.288 | [P10809](http://www.uniprot.org/uniprot/P00505) | 3.67 | 0.322 | [E1BGA0](http://www.uniprot.org/uniprot/P61803) | I3MWE6 | [Q0GAA4](http://www.uniprot.org/uniprot/Q9BT73) |
|  |  | [F1RKH4](http://www.uniprot.org/uniprot/Q96JD6) | 2.46 | 0.002 | [P11532](http://www.uniprot.org/uniprot/P30041) | 2.81 | 0.220 | E1U3L5 | I3NBD0 | Q0Z8R0 |
|  |  | [F1RL20](http://www.uniprot.org/uniprot/Q5T653) | 2.06 | 0.110 | P14868 | 2.13 | 0.100 | E5RH91 | I3NBJ2 | [Q13137](http://www.uniprot.org/uniprot/Q9BVM4) |
|  |  | [F1RM45](http://www.uniprot.org/uniprot/Q16777) | 2.47 | 0.153 | [P15924](http://www.uniprot.org/uniprot/P08246) | 2.81 | 0.197 | E7EQ72 | K7GRY0 | [Q13636](http://www.uniprot.org/uniprot/Q99442) |
|  |  | [F1RRX1](http://www.uniprot.org/uniprot/P49585) | 2.55 | 0.393 | [P31937](http://www.uniprot.org/uniprot/P51648) | 2.50 | 0.349 | F1PBY1 | M3YXR9 | [Q14031](http://www.uniprot.org/uniprot/P36269) |
|  |  | [F1S0L8](http://www.uniprot.org/uniprot/B4DUT8) | 2.10 | 0.328 | [P40925](http://www.uniprot.org/uniprot/P0C263) | 2.31 | 0.734 | F1RIJ5 | [O14925](http://www.uniprot.org/uniprot/Q9BRX2) | Q14203 |
|  |  | [F1SDE3](http://www.uniprot.org/uniprot/P63261) | 2.69 | 0.040 | [P42766](http://www.uniprot.org/uniprot/P00505) | 2.37 | 0.391 | F1RME2 | [O19062](http://www.uniprot.org/uniprot/Q9Y5S9) | [Q15119](http://www.uniprot.org/uniprot/Q9Y375) |
|  |  | [F1SLW6](http://www.uniprot.org/uniprot/Q9Y291) | 2.54 | 0.096 | [P49821](http://www.uniprot.org/uniprot/Q8WZ42) | 4.29 | 0.587 | F1RND9 | [O60826](http://www.uniprot.org/uniprot/Q08380) | [Q16891](http://www.uniprot.org/uniprot/Q99442) |
|  |  | [F7FRK6](http://www.uniprot.org/uniprot/Q9Y478) | 3.79 | 0.159 | P50552 | 2.83 | 0.116 | F1S939 | [O60830](http://www.uniprot.org/uniprot/Q8WZA0) | [Q2QC87](http://www.uniprot.org/uniprot/Q9Y263) |
|  |  | [G1LYB6](http://www.uniprot.org/uniprot/P82921) | 2.66 | 0.095 | [P54886](http://www.uniprot.org/uniprot/P19404) | 2.73 | 0.086 | F1SA12 | [O75369](http://www.uniprot.org/uniprot/P08571) | [Q53EU6](http://www.uniprot.org/uniprot/P02144) |
|  |  | G1M700 | 2.64 | 0.193 | P55809 | 2.00 | 0.225 | F1SB76 | O75663 | [Q53QV2](http://www.uniprot.org/uniprot/Q9C0K7) |
|  |  | G1SB55 | 2.06 | 0.420 | Q01844 | 2.46 | 0.275 | F1SCH0 | [O94855](http://www.uniprot.org/uniprot/P49368) | [Q6FHJ5](http://www.uniprot.org/uniprot/P09012) |
|  |  | G3M5S9 | 2.08 | 0.557 | Q14714 | 2.15 | 0.303 | [F1SCY0](http://www.uniprot.org/uniprot/Q15843) | [O95363](http://www.uniprot.org/uniprot/P34897) | [Q6PKG0](http://www.uniprot.org/uniprot/Q13451) |
|  |  | [G3VYK9](http://www.uniprot.org/uniprot/P14868) | 3.12 | 0.277 | Q16082 | 2.19 | 0.111 | F1SEN4 | [O95571](http://www.uniprot.org/uniprot/P63302) | [Q6Q298](http://www.uniprot.org/uniprot/P25686) |
|  |  | [H0VNA2](http://www.uniprot.org/uniprot/Q8IX12) | 6.33 | 0.379 | [Q16795](http://www.uniprot.org/uniprot/P21912) | 2.90 | 0.298 | F1SJT1 | [O95793](http://www.uniprot.org/uniprot/Q9NWT6) | [Q6Z8B5](http://www.uniprot.org/uniprot/Q8NFU3) |
|  |  | H0XGT6 | 2.01 | 0.162 | Q1T7A8 | 3.09 | 0.288 | F1SMI8 | [O95989](http://www.uniprot.org/uniprot/Q02153) | [Q7Z7G0](http://www.uniprot.org/uniprot/Q99829) |
|  |  | [H0XN85](http://www.uniprot.org/uniprot/P82932) | 2.87 | 0.099 | Q5D144 | 2.02 | 0.376 | [F1SN67](http://www.uniprot.org/uniprot/Q9BRT3) | [P00505](http://www.uniprot.org/uniprot/Q96HP0) | [Q8MGX3](http://www.uniprot.org/uniprot/Q8N5M9) |
|  |  | [H9C5C5](http://www.uniprot.org/uniprot/Q16891) | 2.04 | 0.269 | [Q5RI15](http://www.uniprot.org/uniprot/Q53H82) | 2.05 | 0.042 | F1SN81 | [P02144](http://www.uniprot.org/uniprot/Q9H993) | Q8N0U8 |
|  |  | [H9CWF1](http://www.uniprot.org/uniprot/Q9H4M9) | 3.79 | 0.023 | [Q6NVY1](http://www.uniprot.org/uniprot/P06733) | 2.58 | 0.279 | [F1SP27](http://www.uniprot.org/uniprot/P24666) | [P02144](http://www.uniprot.org/uniprot/Q8N2H3) | [Q8N0X4](http://www.uniprot.org/uniprot/P15311) |
|  |  | [I3L9H6](http://www.uniprot.org/uniprot/P56378) | 2.15 | 0.032 | [Q8IXJ6](http://www.uniprot.org/uniprot/O14521) | 2.01 | 0.428 | F6S3D3 | [P02647](http://www.uniprot.org/uniprot/P24311) | [Q8N1G4](http://www.uniprot.org/uniprot/Q9NSD9) |
|  |  | [I3LCE9](http://www.uniprot.org/uniprot/P22612) | 2.03 | 0.350 | [Q8K575](http://www.uniprot.org/uniprot/Q9Y6K0) | 3.00 | 0.027 | F6SJM8 | [P02675](http://www.uniprot.org/uniprot/Q969Z3) | [Q8N3E9](http://www.uniprot.org/uniprot/P37108) |
|  |  | [I3LEJ9](http://www.uniprot.org/uniprot/E9PK47) | 2.30 | 0.171 | [Q8N142](http://www.uniprot.org/uniprot/Q9H0A8) | 2.10 | 0.486 | F6TB66 | P02770 | [Q8WVJ2](http://www.uniprot.org/uniprot/Q5VYX0) |
|  |  | [I3LLH8](http://www.uniprot.org/uniprot/P49407) | 4.88 | 0.313 | Q8WZ42 | 2.52 | 0.135 | F6UGL6 | P04062 | [Q8WXH0](http://www.uniprot.org/uniprot/P20336) |
|  |  | [I3LLQ8](http://www.uniprot.org/uniprot/P05090) | 3.91 | 0.262 | Q8WZ42 | 2.82 | 0.222 | F6V1W9 | [P04839](http://www.uniprot.org/uniprot/Q14353) | Q8WZ42 |
|  |  | [I3M0Z1](http://www.uniprot.org/uniprot/Q8IWA5) | 2.41 | 0.391 | [Q99700](http://www.uniprot.org/uniprot/Q96MG2) | 2.28 | 0.098 | F6ZDK3 | P07437 | Q969Y2 |
|  |  | [I3MUB6](http://www.uniprot.org/uniprot/P31937) | 2.36 | 0.006 | [Q9NQP4](http://www.uniprot.org/uniprot/Q9Y6I3) | 3.27 | 0.020 | F7BD45 | [P11172](http://www.uniprot.org/uniprot/O15126) | [Q96B36](http://www.uniprot.org/uniprot/Q9NUQ6) |
|  |  | [J3KNV4](http://www.uniprot.org/uniprot/Q9UL25) | 2.50 | 0.120 | Q9NVV0 | 2.34 | 0.448 | F7EP98 | P11172 | [Q96BS2](http://www.uniprot.org/uniprot/Q8IXB1) |
|  |  | [J9JHZ3](http://www.uniprot.org/uniprot/Q01484) | 3.87 | 0.175 | Q9UM00 | 2.09 | 0.115 | F7FV41 | [P13473](http://www.uniprot.org/uniprot/Q8IV08) | Q96BW1 |
|  |  | [K7ED20](http://www.uniprot.org/uniprot/P17655) | 2.20 | 0.322 |  |  |  | F7GF97 | [P15531](http://www.uniprot.org/uniprot/Q92930) | [Q96HP4](http://www.uniprot.org/uniprot/O14828) |
|  |  | [K7EE38](http://www.uniprot.org/uniprot/O43598) | 2.08 | 0.103 |  |  |  | G1L321 | [P24752](http://www.uniprot.org/uniprot/P12277) | Q96RF0 |
|  |  | [K7GMN1](http://www.uniprot.org/uniprot/O75366) | 4.27 | 0.168 |  |  |  | G1Q0L6 | [P25705](http://www.uniprot.org/uniprot/P81605) | Q9C0K7 |
|  |  | [K7GST0](http://www.uniprot.org/uniprot/P46778) | 2.16 | 0.161 |  |  |  | G1TKX3 | [P28331](http://www.uniprot.org/uniprot/Q5U651) | [Q9H1H9](http://www.uniprot.org/uniprot/Q9HAN9) |
|  |  | [K9ISI8](http://www.uniprot.org/uniprot/Q9H2W6) | 2.69 | 0.132 |  |  |  | G1U8R2 | [P31689](http://www.uniprot.org/uniprot/Q12931) | [Q9H4B0](http://www.uniprot.org/uniprot/Q9UMY4) |
|  |  | [L8HKW1](http://www.uniprot.org/uniprot/Q8WYQ3) | 3.19 | 0.560 |  |  |  | G3I0L8 | P31948 | Q9NQC7 |
|  |  | L8INU0 | 2.16 | 0.285 |  |  |  | G3TSG8 | [P36871](http://www.uniprot.org/uniprot/Q499L9) | [Q9NRG7](http://www.uniprot.org/uniprot/Q5JRA6) |
|  |  | L9KZG3 | 5.78 | 0.084 |  |  |  | G3WAY9 | [P46783](http://www.uniprot.org/uniprot/Q9NX05) | [Q9NX55](http://www.uniprot.org/uniprot/A7E2Y1) |
|  |  | [M3UZ93](http://www.uniprot.org/uniprot/P16403) | 3.16 | 0.040 |  |  |  | G7MQE4 | [P49590](http://www.uniprot.org/uniprot/Q9Y5M8) | Q9NYL9 |
|  |  | [M3WE04](http://www.uniprot.org/uniprot/Q15018) | 2.08 | 0.320 |  |  |  | [G8ENM3](http://www.uniprot.org/uniprot/P30408) | [P50148](http://www.uniprot.org/uniprot/P10916) | Q9NZ01 |
|  |  | [M3WRM3](http://www.uniprot.org/uniprot/Q86XE5) | 5.29 | 0.139 |  |  |  | H0V2G7 | [P52272](http://www.uniprot.org/uniprot/P12883) | [Q9NZZ3](http://www.uniprot.org/uniprot/A0FGR8) |
|  |  | M3XFS6 | 2.70 | 0.062 |  |  |  | H9H6U6 | [P54920](http://www.uniprot.org/uniprot/P24310) | Q9UHD9 |
|  |  | O14521 | 3.28 | 0.319 |  |  |  | I0FS69 | [P55769](http://www.uniprot.org/uniprot/P61006) | [Q9Y4X5](http://www.uniprot.org/uniprot/P48449) |
|  |  | [O14548](http://www.uniprot.org/uniprot/P49821) | 2.02 | 0.211 |  |  |  | I3LG15 | [P61923](http://www.uniprot.org/uniprot/P07954) | Q9Y6I3 |
|  |  | [O43707](http://www.uniprot.org/uniprot/Q9Y241) | 3.04 | 0.225 |  |  |  | I3LH91 | P62310 | S9XWM0 |
|  |  | [O60869](http://www.uniprot.org/uniprot/P51888) | 2.22 | 0.099 |  |  |  | I3LI80 | P63092 | S9YBV6 |
|  |  | [O95178](http://www.uniprot.org/uniprot/Q8WZ42) | 3.00 | 0.465 |  |  |  | I3LIA4 | [P63167](http://www.uniprot.org/uniprot/P61952) | W5PG52 |
|  |  | [O95861](http://www.uniprot.org/uniprot/O15511) | 2.50 | 0.109 |  |  |  | [I3LND7](http://www.uniprot.org/uniprot/Q99447) | [P63167](http://www.uniprot.org/uniprot/Q16363) |  |
|  |  | P04406 | 3.37 | 0.108 |  |  |  | I3LNT1 | [P68082](http://www.uniprot.org/uniprot/Q9H0N5) |  |
|  |  |  |  |  |  |  |  |  |  |  |
|  | sham | A0A024RAM4 | 7.38 | 0.178 | P07437 | 2.10 | 0.067 | A0A024R3J7 | O43747 | Q15493 |
|  |  | A0A087X1B7 | 2.19 | 0.169 | P08185 | 2.13 | 0.007 | A0A087WZA9 | O43813 | Q15843 |
|  |  | A1L0T0 | 2.06 | 0.147 | P08246 | 2.55 | 0.064 | A0A091CK67 | O43837 | Q16363 |
|  |  | A1XQT4 | 2.52 | 0.161 | P09493 | 2.03 | 0.331 | A0A0S2Z4P8 | O60832 | [Q3UJV6](http://www.uniprot.org/uniprot/Q03252) |
|  |  | A6NMZ7 | 2.14 | 0.186 | P0C0S5 | 2.07 | 0.221 | A2ABU4 | O75781 | Q53EU6 |
|  |  | B3KN37 | 2.03 | 0.099 | P10916 | 2.21 | 0.254 | A3KMH1 | O95376 | Q58FF8 |
|  |  | B4DW31 | 2.64 | 0.157 | [P11310](http://www.uniprot.org/uniprot/P01042) | 2.22 | 0.113 | A4US67 | O95881 | Q5JTV8 |
|  |  | B5U2X4 | 2.21 | 0.257 | P13533 | 2.04 | 0.442 | A5GFX6 | P00156 | Q5NDL2 |
|  |  | D2HE44 | 2.04 | 0.343 | P17540 | 2.19 | 0.295 | A6QLB7 | P00338 | Q6NSJ2 |
|  |  | E5RGR0 | 3.86 | 0.058 | P18206 | 2.25 | 0.355 | B2RE06 | P00973 | Q6NUK1 |
|  |  | F1PJ01 | 2.38 | 0.188 | P19827 | 2.14 | 0.046 | [B4DSZ2](http://www.uniprot.org/uniprot/Q92643) | P00973 | Q6Q298 |
|  |  | F1SPB4 | 2.24 | 0.140 | P22352 | 2.29 | 0.196 | B4E1G1 | P01009 | Q6TFL4 |
|  |  | F6Q903 | 2.29 | 0.171 | P29728 | 2.23 | 0.161 | B4E341 | P02144 | Q86TD4 |
|  |  | F6QDS8 | 2.06 | 0.524 | P30536 | 3.71 | 0.069 | C9JGT6 | [P02751](http://www.uniprot.org/uniprot/P30044) | Q86U42 |
|  |  | F6RGG7 | 3.01 | 0.197 | P31146 | 2.43 | 0.142 | [C9JRD2](http://www.uniprot.org/uniprot/O14925) | P04406 | Q86UX2 |
|  |  | F6XF06 | 4.36 | 0.224 | [P31415](http://www.uniprot.org/uniprot/P01011) | 3.41 | 0.160 | C9JUP3 | P05165 | Q8IX12 |
|  |  | F6YZV6 | 2.25 | 0.602 | P39060 | 2.42 | 0.001 | C9K0I3 | P05166 | Q8WV93 |
|  |  | F8W0P7 | 2.56 | 0.086 | P47989 | 2.81 | 0.098 | D2WL18 | P05556 | Q8WWI1 |
|  |  | G1MCU6 | 2.51 | 0.039 | P49748 | 2.50 | 0.519 | E2RDW3 | P06733 | [Q8WWI5](http://www.uniprot.org/uniprot/P11142) |
|  |  | G1P0V8 | 2.67 | 0.132 | P50453 | 2.24 | 0.190 | [E7ERU0](http://www.uniprot.org/uniprot/P13533) | P07195 | Q969V3 |
|  |  | G1PRD8 | 2.17 | 0.014 | P50991 | 3.18 | 0.079 | F1M614 | P13533 | Q969Y2 |
|  |  | G1QLG3 | 2.72 | 0.260 | [P51553](http://www.uniprot.org/uniprot/Q9NQS7) | 2.59 | 0.443 | F1P9P5 | P13647 | Q96DV4 |
|  |  | G1SVG3 | 2.07 | 0.118 | P52565 | 2.38 | 0.018 | F1RL48 | P13716 | Q96F86 |
|  |  | G3T1C5 | 2.20 | 0.448 | P55884 | 2.05 | 0.019 | F1SJU4 | P16403 | Q96QK1 |
|  |  | G3T7Z5 | 2.47 | 0.174 | P60891 | 2.24 | 0.063 | F6U6H7 | P19623 | Q96QR8 |
|  |  | G3TZD9 | 2.77 | 0.272 | P61970 | 2.16 | 0.093 | F6UXQ1 | [P22612](http://www.uniprot.org/uniprot/Q9NRG7) | Q96S66 |
|  |  | G3WGT2 | 2.31 | 0.107 | Q00325 | 2.76 | 0.306 | F6XI41 | P26232 | Q96T51 |
|  |  | G7MFY5 | 2.40 | 0.090 | Q01449 | 4.14 | 0.042 | F7BD45 | P28331 | Q99643 |
|  |  | H0WGT6 | 4.26 | 0.164 | Q01995 | 2.07 | 0.119 | F7BM46 | P28331 | Q9BWM7 |
|  |  | H0XTM2 | 3.02 | 0.214 | Q04446 | 2.11 | 0.070 | F7F6K6 | P31025 | Q9BXP5 |
|  |  | H2PM20 | 4.72 | 0.341 | [Q13158](http://www.uniprot.org/uniprot/P29400) | 2.25 | 0.185 | F7HSV9 | P31151 | Q9H330 |
|  |  | [I0FRX6](http://www.uniprot.org/uniprot/P47989) | 2.55 | 0.111 | Q13496 | 2.06 | 0.269 | F8VU34 | P31948 | Q9H490 |
|  |  | I3L6U3 | 2.53 | 0.121 | Q14195 | 2.06 | 0.019 | G1M4Y1 | P31949 | Q9H9S3 |
|  |  | I3LCC2 | 2.12 | 0.036 | Q15555 | 2.17 | 0.233 | G1TKX3 | P32418 | Q9HCP6 |
|  |  | I3LK34 | 2.11 | 0.347 | Q16363 | 2.51 | 0.249 | G1U147 | P35749 | Q9HDC5 |
|  |  | J3KT73 | 2.61 | 0.250 | Q5HYJ1 | 4.20 | 0.091 | G3UC75 | P36269 | Q9NZB2 |
|  |  | J9P969 | 2.04 | 0.118 | Q5JRX3 | 2.23 | 0.071 | G3VGN2 | P36542 | Q9UBG0 |
|  |  | K7ECB7 | 4.78 | 0.137 | Q5JRX3 | 2.32 | 0.110 | G9KGS2 | P37108 | Q9UBQ0 |
|  |  | L8AXK3 | 2.34 | 0.063 | Q5T6V5 | 2.78 | 0.207 | H0WCW9 | P47755 | Q9UHY7 |
|  |  | L8B0W5 | 2.98 | 0.084 | Q5VST9 | 2.33 | 0.419 | H0WMC0 | P49588 | Q9UI14 |
|  |  | [L8B180](http://www.uniprot.org/uniprot/Q16775) | 2.26 | 0.273 | Q6JQN1 | 5.99 | 0.018 | H0WNY6 | P50454 | Q9UJU6 |
|  |  | M3XPN1 | 2.83 | 0.101 | Q7YQ91 | 2.01 | 0.255 | H0XAC3 | P51911 | Q9UL15 |
|  |  | O00754 | 2.32 | 0.194 | Q7Z7Q0 | 2.37 | 0.175 | H0XMU5 | P54886 | Q9Y3B7 |
|  |  | O75155 | 4.79 | 0.134 | Q86UT6 | 2.67 | 0.047 | I3LLW3 | P62851 | Q9Y5A7 |
|  |  | O95169 | 4.35 | 0.088 | [Q8IVD9](http://www.uniprot.org/uniprot/P02768) | 2.09 | 0.168 | I3MNP9 | P62854 | S9XWM0 |
|  |  | P00387 | 2.59 | 0.167 | Q8WZ42 | 2.49 | 0.417 | L5K156 | Q00169 | S9YQH0 |
|  |  | P00505 | 2.31 | 0.128 | Q95KR6 | 2.19 | 0.093 | L5KUF8 | Q01082 |  |
|  |  | P01024 | 2.38 | 0.081 | Q9H4M3 | 3.82 | 0.194 | L8B149 | Q01484 |  |
|  |  | P02144 | 2.28 | 0.498 | Q9NRG7 | 2.57 | 0.060 | M3W219 | Q02539 |  |
|  |  | P04083 | 2.35 | 0.182 | [Q9UH62](http://www.uniprot.org/uniprot/O75600) | 2.19 | 0.109 | M3W9K0 | Q08289 |  |
|  |  | [P04792](http://www.uniprot.org/uniprot/Q9Y2Q3) | 2.54 | 0.414 | Q9UI30 | 2.08 | 0.103 | M3WAS7 | [Q13217](http://www.uniprot.org/uniprot/Q7L0Y3) |  |
|  |  | [P05164](http://www.uniprot.org/uniprot/P10916) | 2.17 | 0.072 | Q9Y4G6 | 2.33 | 0.119 | M3WAS7 | Q13325 |  |
|  |  | P05413 | 4.32 | 0.572 | Q9Y512 | 2.05 | 0.050 | M3YIS8 | Q13425 |  |
|  |  | P05976 | 2.20 | 0.013 | Q9Y5M8 | 4.39 | 0.167 | O14530 | Q14141 |  |
|  |  | P06702 | 2.41 | 0.401 | Q9Y5U8 | 2.14 | 0.117 | O14734 | Q15119 |  |
|  |  | P06744 | 2.45 | 0.140 | S9YX05 | 2.52 | 0.536 | O43324 | Q15388 |  |
|  |  |  |  |  |  |  |  |  |  |  |
| early reperfusion | RIPC | D2HW06 | 2.31 | 0.295 |  |  |  | [A0FGR8](http://www.uniprot.org/uniprot/A0FGR8) | [P07951](http://www.uniprot.org/uniprot/P07951) | [Q5T653](http://www.uniprot.org/uniprot/Q5T653) |
|  |  | F1SA62 | 4.19 | 0.391 |  |  |  | [A7E2Y1](http://www.uniprot.org/uniprot/A7E2Y1) | [P07954](http://www.uniprot.org/uniprot/P07954) | [Q5U651](http://www.uniprot.org/uniprot/Q5U651) |
|  |  | F1SFG7 | 2.52 | 0.272 |  |  |  | [B4DRU9](http://www.uniprot.org/uniprot/B4DRU9) | [P08571](http://www.uniprot.org/uniprot/P08571) | Q5VWP3 |
|  |  | F1SVD5 | 2.74 | 0.354 |  |  |  | [B4DUT8](http://www.uniprot.org/uniprot/B4DUT8) | [P09012](http://www.uniprot.org/uniprot/P09012) | [Q5VYX0](http://www.uniprot.org/uniprot/Q5VYX0) |
|  |  | G1M5S6 | 2.85 | 0.333 |  |  |  | E2R5W6 | [P10916](http://www.uniprot.org/uniprot/P10916) | Q66K74 |
|  |  | G7MHF2 | 2.96 | 0.111 |  |  |  | E2RHL0 | [P12277](http://www.uniprot.org/uniprot/P12277) | [Q86XE5](http://www.uniprot.org/uniprot/Q86XE5) |
|  |  | I3LPU4 | 2.43 | 0.211 |  |  |  | E7ETR0 | [P12883](http://www.uniprot.org/uniprot/P12883) | [Q8IV08](http://www.uniprot.org/uniprot/Q8IV08) |
|  |  | J9NV69 | 2.51 | 0.134 |  |  |  | [E9PK47](http://www.uniprot.org/uniprot/E9PK47) | [P14868](http://www.uniprot.org/uniprot/P14868) | [Q8IWA5](http://www.uniprot.org/uniprot/Q8IWA5) |
|  |  | M3UZ63 | 2.38 | 0.106 |  |  |  | F1PPZ7 | P15175 | [Q8IX12](http://www.uniprot.org/uniprot/Q8IX12) |
|  |  | [O14521](http://www.uniprot.org/uniprot/O14521) | 2.79 | 0.288 |  |  |  | F1RG57 | [P15311](http://www.uniprot.org/uniprot/P15311) | [Q8IXB1](http://www.uniprot.org/uniprot/Q8IXB1) |
|  |  | [O15511](http://www.uniprot.org/uniprot/O15511) | 2.18 | 0.143 |  |  |  | F1RQN4 | [P17655](http://www.uniprot.org/uniprot/P17655) | [Q8N2H3](http://www.uniprot.org/uniprot/Q8N2H3) |
|  |  | [O95716](http://www.uniprot.org/uniprot/O95716) | 2.05 | 0.104 |  |  |  | F1RR89 | [P20336](http://www.uniprot.org/uniprot/P20336) | [Q8N5M9](http://www.uniprot.org/uniprot/Q8N5M9) |
|  |  | [P17174](http://www.uniprot.org/uniprot/P00505) | 2.34 | 0.123 |  |  |  | F1RY70 | [P22612](http://www.uniprot.org/uniprot/P22612) | [H0UI37](http://www.uniprot.org/uniprot/Q8NFU3) |
|  |  | [P00505](http://www.uniprot.org/uniprot/P00505) | 2.33 | 0.660 |  |  |  | F1S066 | [P24310](http://www.uniprot.org/uniprot/P24310) | [Q8WYQ3](http://www.uniprot.org/uniprot/Q8WYQ3) |
|  |  | [P05165](http://www.uniprot.org/uniprot/P05165) | 2.21 | 0.103 |  |  |  | F1S2W6 | [P24311](http://www.uniprot.org/uniprot/P24311) | [Q8WZA0](http://www.uniprot.org/uniprot/Q8WZA0) |
|  |  | [P06733](http://www.uniprot.org/uniprot/P06733) | 2.47 | 0.144 |  |  |  | F1S6C3 | [P24666](http://www.uniprot.org/uniprot/P24666) | [Q92930](http://www.uniprot.org/uniprot/Q92930) |
|  |  | P07951 | 3.26 | 0.406 |  |  |  | F1SKM6 | [P25686](http://www.uniprot.org/uniprot/P25686) | [Q969Z3](http://www.uniprot.org/uniprot/Q969Z3) |
|  |  | [P08246](http://www.uniprot.org/uniprot/P08246) | 3.28 | 0.172 |  |  |  | F6YAV8 | [P30408](http://www.uniprot.org/uniprot/P30408) | [Q96HP0](http://www.uniprot.org/uniprot/Q96HP0) |
|  |  | [P0C263](http://www.uniprot.org/uniprot/P0C263) | 2.67 | 0.155 |  |  |  | F6ZYR7 | [P34897](http://www.uniprot.org/uniprot/P34897) | [Q99442](http://www.uniprot.org/uniprot/Q99442) |
|  |  | [P10636](http://www.uniprot.org/uniprot/P10636) | 2.15 | 0.561 |  |  |  | F7HSY5 | P35580 | [Q99442](http://www.uniprot.org/uniprot/Q99442) |
|  |  | [P16403](http://www.uniprot.org/uniprot/P16403) | 3.11 | 0.024 |  |  |  | G1L4J7 | [P36269](http://www.uniprot.org/uniprot/P36269) | [Q99447](http://www.uniprot.org/uniprot/Q99447) |
|  |  | [P19404](http://www.uniprot.org/uniprot/P19404) | 3.62 | 0.305 |  |  |  | G1U147 | [P37108](http://www.uniprot.org/uniprot/P37108) | [Q99829](http://www.uniprot.org/uniprot/Q99829) |
|  |  | [P21912](http://www.uniprot.org/uniprot/P21912) | 2.42 | 0.182 |  |  |  | G1U8R2 | [P46778](http://www.uniprot.org/uniprot/P46778) | [Q9BRT3](http://www.uniprot.org/uniprot/Q9BRT3) |
|  |  | [P30041](http://www.uniprot.org/uniprot/P30041) | 4.76 | 0.093 |  |  |  | G3SL42 | [P48449](http://www.uniprot.org/uniprot/P48449) | [Q9BRX2](http://www.uniprot.org/uniprot/Q9BRX2) |
|  |  | [P31937](http://www.uniprot.org/uniprot/P31937) | 2.18 | 0.188 |  |  |  | G3TSG8 | [P49368](http://www.uniprot.org/uniprot/P49368) | [Q9BT73](http://www.uniprot.org/uniprot/Q9BT73) |
|  |  | [P35237](http://www.uniprot.org/uniprot/P35237) | 2.43 | 0.092 |  |  |  | H0UY16 | [P49407](http://www.uniprot.org/uniprot/P49407) | [Q9BVM4](http://www.uniprot.org/uniprot/Q9BVM4) |
|  |  | [P49821](http://www.uniprot.org/uniprot/P49821) | 3.31 | 0.313 |  |  |  | H0VAA7 | [P49407](http://www.uniprot.org/uniprot/P49407) | [Q9C0K7](http://www.uniprot.org/uniprot/Q9C0K7) |
|  |  | [P51648](http://www.uniprot.org/uniprot/P51648) | 2.40 | 0.347 |  |  |  | H0VNA2 | [P49585](http://www.uniprot.org/uniprot/P49585) | [Q9H0N5](http://www.uniprot.org/uniprot/Q9H0N5) |
|  |  | [P51888](http://www.uniprot.org/uniprot/P51888) | 2.26 | 0.646 |  |  |  | H0XBU7 | [P61006](http://www.uniprot.org/uniprot/P61006) | [Q9H2W6](http://www.uniprot.org/uniprot/Q9H2W6) |
|  |  | [P56378](http://www.uniprot.org/uniprot/P56378) | 3.24 | 0.309 |  |  |  | H0XMU5 | [P61803](http://www.uniprot.org/uniprot/P61803) | Q9H3P7 |
|  |  | [P63261](http://www.uniprot.org/uniprot/P63261) | 12.72 | 0.347 |  |  |  | H2NHD6 | [P61952](http://www.uniprot.org/uniprot/P61952) | [Q9H993](http://www.uniprot.org/uniprot/Q9H993) |
|  |  | [Q16777](http://www.uniprot.org/uniprot/Q16777) | 3.08 | 0.119 |  |  |  | H9CWF1 | [P61960](http://www.uniprot.org/uniprot/P61960) | [Q9HAN9](http://www.uniprot.org/uniprot/Q9HAN9) |
|  |  | [Q16891](http://www.uniprot.org/uniprot/Q16891) | 3.94 | 0.081 |  |  |  | H9H6B2 | [P63302](http://www.uniprot.org/uniprot/P63302) | [Q9NSD9](http://www.uniprot.org/uniprot/Q9NSD9) |
|  |  | [Q53H82](http://www.uniprot.org/uniprot/Q53H82) | 2.59 | 0.007 |  |  |  | I3LGM5 | P68371 | [Q9NUQ6](http://www.uniprot.org/uniprot/Q9NUQ6) |
|  |  | Q5TEC6 | 2.12 | 0.266 |  |  |  | I3LHU9 | [P81605](http://www.uniprot.org/uniprot/P81605) | Q9NW15 |
|  |  | [Q5TGZ0](http://www.uniprot.org/uniprot/Q5TGZ0) | 2.45 | 0.101 |  |  |  | I3LI96 | [P82921](http://www.uniprot.org/uniprot/P82921) | [Q9NWT6](http://www.uniprot.org/uniprot/Q9NWT6) |
|  |  | [Q8WZ42](http://www.uniprot.org/uniprot/Q8WZ42) | 3.25 | 0.113 |  |  |  | I3LLW3 | [P82932](http://www.uniprot.org/uniprot/P82932) | [Q9NX05](http://www.uniprot.org/uniprot/Q9NX05) |
|  |  | [Q8WZ42](http://www.uniprot.org/uniprot/Q8WZ42) | 2.98 | 0.261 |  |  |  | I3LNT1 | [Q01484](http://www.uniprot.org/uniprot/Q01484) | Q9NYF8 |
|  |  | [Q8WZ42](http://www.uniprot.org/uniprot/Q8WZ42) | 2.05 | 0.313 |  |  |  | I3LNV3 | [Q02153](http://www.uniprot.org/uniprot/Q02153) | [Q9NZB2](http://www.uniprot.org/uniprot/Q9NZB2) |
|  |  | Q8WZ42 | 2.35 | 0.355 |  |  |  | I3LPU4 | [Q08380](http://www.uniprot.org/uniprot/Q08380) | [Q9UKX3](http://www.uniprot.org/uniprot/Q9UKX3) |
|  |  | [Q96JD6](http://www.uniprot.org/uniprot/Q96JD6) | 3.00 | 0.349 |  |  |  | K7E6Y2 | [Q12931](http://www.uniprot.org/uniprot/Q12931) | [Q9UMY4](http://www.uniprot.org/uniprot/Q9UMY4) |
|  |  | [Q96MG2](http://www.uniprot.org/uniprot/Q96MG2) | 2.31 | 0.530 |  |  |  | K9K3F7 | [Q13451](http://www.uniprot.org/uniprot/Q13451) | [Q9Y263](http://www.uniprot.org/uniprot/Q9Y263) |
|  |  | [Q9H0A8](http://www.uniprot.org/uniprot/Q9H0A8) | 2.37 | 0.208 |  |  |  | L8IM86 | Q13683 | [Q9Y291](http://www.uniprot.org/uniprot/Q9Y291) |
|  |  | [Q9H4M9](http://www.uniprot.org/uniprot/Q9H4M9) | 3.00 | 0.029 |  |  |  | L8INU0 | [Q14353](http://www.uniprot.org/uniprot/Q14353) | [Q9Y375](http://www.uniprot.org/uniprot/Q9Y375) |
|  |  | Q9UKY7 | 2.07 | 0.126 |  |  |  | O14734 | [Q15018](http://www.uniprot.org/uniprot/Q15018) | Q9Y3D5 |
|  |  | [Q9UL25](http://www.uniprot.org/uniprot/Q9UL25) | 2.29 | 0.217 |  |  |  | [O14828](http://www.uniprot.org/uniprot/O14828) | Q15119 | [Q9Y478](http://www.uniprot.org/uniprot/Q9Y478) |
|  |  | [Q9Y241](http://www.uniprot.org/uniprot/Q9Y241) | 2.00 | 0.144 |  |  |  | [O15126](http://www.uniprot.org/uniprot/O15126) | [Q15843](http://www.uniprot.org/uniprot/Q15843) | [Q9Y5M8](http://www.uniprot.org/uniprot/Q9Y5M8) |
|  |  | [Q9Y6I3](http://www.uniprot.org/uniprot/Q9Y6I3) | 2.16 | 0.005 |  |  |  | [O43598](http://www.uniprot.org/uniprot/O43598) | [Q16363](http://www.uniprot.org/uniprot/Q16363) | [Q9Y5S9](http://www.uniprot.org/uniprot/Q9Y5S9) |
|  |  | [Q9Y6K0](http://www.uniprot.org/uniprot/Q9Y6K0) | 2.13 | 0.031 |  |  |  | [O75366](http://www.uniprot.org/uniprot/O75366) | [Q3MHD2](http://www.uniprot.org/uniprot/Q3MHD2) | S9YQH0 |
|  |  | W5P8Z5 | 2.35 | 0.241 |  |  |  | [P02144](http://www.uniprot.org/uniprot/P02144) | [Q499L9](http://www.uniprot.org/uniprot/Q499L9) | W5Q9V9 |
|  |  | W5QAH5 | 2.95 | 0.127 |  |  |  | [P04406](http://www.uniprot.org/uniprot/P04406) | [Q4G0W2](http://www.uniprot.org/uniprot/Q4G0W2) |  |
|  |  |  |  |  |  |  |  |  |  |  |
|  | sham | A4D1B8 | 2.32 | 0.032 | P04406 | 2.53 | 0.148 | A0A024R3J7 | O43715 | Q14019 |
|  |  | A8K787 | 2.64 | 0.146 | P05161 | 2.31 | 0.234 | B4DUT8 | O43847 | Q14031 |
|  |  | B7ZMF0 | 2.20 | 0.388 | P05413 | 3.34 | 0.419 | D2HJA7 | O43852 | Q14197 |
|  |  | D2I822 | 2.01 | 0.31 | P07478 | 2.33 | 0.100 | D3ZAS1 | O60443 | Q14669 |
|  |  | E9PAV3 | 2.27 | 0.184 | P0C263 | 2.07 | 0.024 | F1RG57 | O75390 | Q15119 |
|  |  | F1PGM1 | 2.16 | 0.08 | [P10916](http://www.uniprot.org/uniprot/P10916) | 3.19 | 0.244 | F1RV76 | O75629 | Q15382 |
|  |  | F1PJ01 | 2.29 | 0.28 | P17540 | 2.01 | 0.273 | F1RW61 | O75879 | Q15843 |
|  |  | F1RN76 | 3.37 | 0.04 | P18124 | 2.28 | 0.096 | F1RWH6 | P01920 | Q16363 |
|  |  | F1RUQ0 | 3.59 | 0.02 | P21333 | 3.15 | 0.134 | F1S429 | P04844 | Q16647 |
|  |  | F1SFI6 | 2.43 | 0.01 | P22392 | 2.09 | 0.283 | F1SCC9 | P09105 | Q2M2I8 |
|  |  | F1SK65 | 2.09 | 0.17 | P22392 | 2.10 | 0.112 | F1SHK6 | P09455 | Q2TB90 |
|  |  | F6QBU5 | 2.55 | 0.25 | [P29400](http://www.uniprot.org/uniprot/P29400) | 3.03 | 0.051 | F1SKM6 | [P11142](http://www.uniprot.org/uniprot/P11142) | Q3UJV6 |
|  |  | F6QDS8 | 2.47 | 0.21 | P36542 | 2.66 | 0.557 | F1SQY2 | P11387 | Q5VST9 |
|  |  | F6U6H7 | 2.42 | 0.20 | P40925 | 2.55 | 0.278 | F6ZFT7 | P12235 | Q5VTT5 |
|  |  | G1L2C8 | 2.49 | 0.11 | P46939 | 2.06 | 0.115 | F6ZYR7 | [P13533](http://www.uniprot.org/uniprot/P13533) | Q66K74 |
|  |  | G1LG30 | 2.62 | 0.05 | [P47989](http://www.uniprot.org/uniprot/P47989) | 3.93 | 0.068 | F7BD45 | P15121 | Q6DD88 |
|  |  | G1T2A9 | 2.13 | 0.19 | P49748 | 2.29 | 0.416 | F7GC91 | P15531 | Q6Q298 |
|  |  | G3W5K4 | 2.06 | 0.40 | P50135 | 2.44 | 0.017 | F7I6G8 | P19367 | [Q7L0Y3](http://www.uniprot.org/uniprot/Q7L0Y3) |
|  |  | I3L723 | 2.05 | 0.017 | P62273 | 2.19 | 0.007 | G1LMW5 | P19404 | Q86TU7 |
|  |  | I3L818 | 2.33 | 0.10 | P69905 | 2.98 | 0.291 | G1T0G9 | P20290 | Q86U42 |
|  |  | I3M0Z1 | 2.16 | 0.19 | P84095 | 2.15 | 0.011 | G1TNP7 | P21399 | Q8N1G4 |
|  |  | I3MZS6 | 3.78 | 0.44 | Q13126 | 2.13 | 0.186 | G1U2S7 | P27169 | Q8N3K9 |
|  |  | K7ECB7 | 3.50 | 0.01 | Q13361 | 2.56 | 0.152 | G3RPH6 | P27918 | Q8NDY3 |
|  |  | K9IWS2 | 3.02 | 0.22 | [Q16775](http://www.uniprot.org/uniprot/Q16775) | 2.82 | 0.158 | G3UYV7 | P29728 | [Q92643](http://www.uniprot.org/uniprot/Q92643) |
|  |  | L8B0V2 | 2.01 | 0.16 | Q16795 | 2.95 | 0.244 | G9KGS2 | [P30044](http://www.uniprot.org/uniprot/P30044) | Q92804 |
|  |  | L8B0W4 | 2.64 | 0.01 | Q5JRX3 | 2.22 | 0.432 | H0VAA7 | P30566 | Q95KR5 |
|  |  | M3WYH4 | 2.34 | 0.17 | Q7L9L4 | 2.66 | 0.103 | H0WIC5 | P41439 | Q96LL9 |
|  |  | M3XUF3 | 2.13 | 0.01 | Q7Z3Y5 | 2.53 | 0.055 | H0WM58 | P48047 | Q96RF0 |
|  |  | M3XZ28 | 2.05 | 0.13 | Q86UX2 | 2.03 | 0.169 | H2NHD6 | P48454 | Q99700 |
|  |  | M3YIS8 | 2.06 | 0.23 | Q92974 | 2.29 | 0.094 | H2PM20 | P50148 | Q99829 |
|  |  | O60763 | 4.01 | 0.068 | Q96CM8 | 2.05 | 0.354 | H9CWF1 | P50402 | Q9BSD7 |
|  |  | O60925 | 4.32 | 0.055 | Q96DG6 | 2.91 | 0.247 | H9H6U6 | P52434 | Q9GZP4 |
|  |  | O75190 | 2.12 | 0.437 | Q9H0B6 | 2.11 | 0.028 | I3LNT1 | P53396 | Q9GZS3 |
|  |  | O75533 | 2.45 | 0.064 | Q9H1A4 | 4.72 | 0.116 | I3MNA1 | P53680 | Q9H490 |
|  |  | [O75600](http://www.uniprot.org/uniprot/O75600) | 2.02 | 0.063 | Q9HCP6 | 3.07 | 0.009 | K7GMW0 | P61769 | Q9HB19 |
|  |  | O95678 | 2.19 | 0.104 | Q9NPC6 | 2.46 | 0.382 | K9J1X2 | P68400 | Q9HB71 |
|  |  | P00973 | 5.96 | 0.037 | [Q9NQS7](http://www.uniprot.org/uniprot/Q9NQS7) | 2.29 | 0.187 | K9J3H5 | P80108 | Q9HBK9 |
|  |  | [P01011](http://www.uniprot.org/uniprot/P01011) | 2.31 | 0.299 | Q9NRN7 | 2.16 | 0.084 | L8AXL3 | P81605 | [Q9NRG7](http://www.uniprot.org/uniprot/Q9NRG7) |
|  |  | [P01042](http://www.uniprot.org/uniprot/P01042) | 2.39 | 0.107 | Q9NRX4 | 16.04 | 0.117 | L8B0Y0 | P82932 | Q9NZJ6 |
|  |  | P02647 | 2.15 | 0.001 | Q9NUV9 | 2.06 | 0.112 | L8B173 | P82933 | Q9P0M6 |
|  |  | P02652 | 3.40 | 0.064 | Q9NV70 | 2.66 | 0.370 | M3UZ93 | P99999 | Q9UDY4 |
|  |  | P02766 | 2.18 | 0.014 | Q9UBQ0 | 2.04 | 0.122 | M3W9K0 | Q00341 | Q9UMY4 |
|  |  | [P02768](http://www.uniprot.org/uniprot/P02768) | 3.06 | 0.114 | [Q9Y2Q3](http://www.uniprot.org/uniprot/Q9Y2Q3) | 2.45 | 0.270 | M3WDI2 | Q01082 | S9XWM0 |
|  |  | P03928 | 2.68 | 0.027 | Q9Y4G6 | 2.64 | 0.044 | M3Y4B9 | [Q03252](http://www.uniprot.org/uniprot/Q03252) | S9YQH0 |
|  |  | P04040 | 2.03 | 0.606 | W5PHJ7 | 4.70 | 0.21 | O00567 | Q08289 | U3FHN3 |
|  |  | P04114 | 2.89 | 0.114 |  |  |  | O14618 | Q12974 |  |
|  |  |  |  |  |  |  |  |  |  |  |
| RIPC | baseline | A5A8W4 | 2.01 | 0.054 |  |  |  | A0A061I3X4 | G1R057 | M3UZ72 |
|  |  | D2GVA5 | 2.03 | 0.124 |  |  |  | A0A088Q0A9 | G1R5L2 | M3UZ93 |
|  |  | D2H042 | 3.42 | 0.297 |  |  |  | A1L573 | G3GW79 | M3VWN3 |
|  |  | D2H5D5 | 2.05 | 0.196 |  |  |  | A1XQV9 | G3I0L8 | M3WAU6 |
|  |  | D2I822 | 2.78 | 0.102 |  |  |  | B4DSZ2 | G3IM06 | M3WWI9 |
|  |  | F1RKH4 | 2.13 | 0.006 |  |  |  | B8XZY6 | G3R546 | M3Y4T8 |
|  |  | F1RRT2 | 2.10 | 0.154 |  |  |  | C9JM82 | G3RNJ8 | O62680 |
|  |  | F1RSQ0 | 2.75 | 0.054 |  |  |  | D2H7N1 | G3S235 | P02196 |
|  |  | F1S1A9 | 2.79 | 0.151 |  |  |  | D2HC73 | G3SEI8 | P68083 |
|  |  | F1S395 | 2.11 | 0.030 |  |  |  | D3GGC9 | G3TRP2 | Q1JPD3 |
|  |  | F6R6I2 | 2.79 | 0.400 |  |  |  | E1BGA0 | G3X9Q4 | Q29433 |
|  |  | F7GC91 | 2.25 | 0.123 |  |  |  | E1U3L5 | G5BEA9 | Q29558 |
|  |  | G1T2A9 | 2.21 | 0.005 |  |  |  | E7EQ72 | G5BHB5 | Q2HJ88 |
|  |  | G2HEB0 | 2.25 | 0.084 |  |  |  | F1PBY1 | G9K746 | Q56VQ1 |
|  |  | G3HD84 | 2.11 | 0.375 |  |  |  | F1RGD8 | G9KGS2 | Q862K7 |
|  |  | G3IMT1 | 2.21 | 0.017 |  |  |  | F1RIJ5 | G9KIR6 | Q8MGX3 |
|  |  | G3VWR9 | 9.78 | 0.159 |  |  |  | F1RIY8 | H0VV89 | Q8VDM6 |
|  |  | G5AM64 | 2.19 | 0.242 |  |  |  | F1RMZ0 | H0YBW1 | Q95KR6 |
|  |  | G5BWW9 | 2.19 | 0.054 |  |  |  | F1RND9 | H2PBX9 | Q95LK3 |
|  |  | H0V2G7 | 2.35 | 0.382 |  |  |  | F1RR05 | I3L0S1 | Q9DCM0 |
|  |  | H0VNA2 | 4.04 | 0.364 |  |  |  | F1RS78 | I3L9W8 | Q9MZU5 |
|  |  | H0Y0P4 | 2.59 | 0.057 |  |  |  | F1RV76 | I3LAQ4 | Q9WUS8 |
|  |  | I3L945 | 2.17 | 0.018 |  |  |  | F1S4P4 | I3LDA4 | R9S082 |
|  |  | I3LAQ3 | 2.19 | 0.143 |  |  |  | F1S8H4 | I3LDF4 | S7MGL6 |
|  |  | I3LEJ9 | 2.23 | 0.169 |  |  |  | F1S939 | I3LH91 | S7N9K0 |
|  |  | I3LLI0 | 3.47 | 0.277 |  |  |  | F1SCC9 | I3LLH8 | S7NBA1 |
|  |  | I3LTB8 | 2.41 | 0.145 |  |  |  | F1SCH0 | I3LP69 | S7PFN9 |
|  |  | I3M0Z1 | 2.73 | 0.149 |  |  |  | F1SEN4 | I3LU60 | S7QAQ6 |
|  |  | K7CFW7 | 2.74 | 0.345 |  |  |  | F1SJT1 | I3LUJ3 | S9WDM7 |
|  |  | K9IW43 | 2.95 | 0.077 |  |  |  | F1SMI8 | I3LXB8 | S9WXV1 |
|  |  | M3XUF3 | 2.02 | 0.136 |  |  |  | F1SN67 | K7CSL4 | S9XHD5 |
|  |  | M3YIT4 | 2.18 | 0.040 |  |  |  | F6QCQ2 | K7GRK7 | S9XWM0 |
|  |  | Q0Z8R0 | 2.15 | 0.017 |  |  |  | F6S3D3 | K7GT02 | T0MGN3 |
|  |  | Q3MHJ9 | 2.39 | 0.028 |  |  |  | F6UGL6 | K9IV14 | U3CLF7 |
|  |  | Q5R9G9 | 2.51 | 0.379 |  |  |  | F6YB76 | K9J6J4 | U6DJM8 |
|  |  | Q8K575 | 2.26 | 0.306 |  |  |  | F6Z006 | L5KE51 | W5NWG5 |
|  |  | Q8WP14 | 2.02 | 0.055 |  |  |  | F6Z4C3 | L5L114 | W5PAT8 |
|  |  | S7N9H1 | 2.16 | 0.256 |  |  |  | F6ZDK3 | L5MAB8 | W5PW12 |
|  |  | S9WRT4 | 2.94 | 0.024 |  |  |  | F7D1P9 | L5MIN4 | W5Q922 |
|  |  | S9WZP7 | 3.11 | 0.328 |  |  |  | F7DBE9 | L8ISB4 | W5QI15 |
|  |  | S9XHQ7 | 2.20 | 0.551 |  |  |  | F7FWZ0 | L9J8R9 |  |
|  |  | W5PZB9 | 3.25 | 0.026 |  |  |  | G1NW81 | L9KSR8 |  |
|  |  | W5Q9V9 | 3.57 | 0.095 |  |  |  | G1NZ80 | M3TYG2 |  |
|  |  |  |  |  |  |  |  |  |  |  |
|  | early reperfusion | A0A061HWM5 | 2.98 | 0.009 |  |  |  | A0A023PN04 | F8WF48 | L5KUF8 |
|  |  | A1XQT4 | 2.06 | 0.328 |  |  |  | A2BGI8 | F8WIB1 | L5LN23 |
|  |  | D3ZC28 | 2.44 | 0.140 |  |  |  | A5GFT5 | G1NXH2 | L8HRZ8 |
|  |  | D4A9P9 | 2.01 | 0.102 |  |  |  | A5GFX6 | G1P583 | L8IHJ2 |
|  |  | E2RHL0 | 2.82 | 0.106 |  |  |  | A6QLB7 | G1PVK0 | L8IMM8 |
|  |  | E5RGR0 | 2.47 | 0.064 |  |  |  | A6QLN6 | G1Q0L6 | L8IS35 |
|  |  | E7CF08 | 2.80 | 0.061 |  |  |  | A7XUJ6 | G1SCR3 | L8Y6R9 |
|  |  | F1RIA7 | 2.72 | 0.069 |  |  |  | A8DUV1 | G1SN11 | L9JEV2 |
|  |  | F1RM24 | 2.61 | 0.416 |  |  |  | B0CM47 | G3HDL7 | L9KQD6 |
|  |  | F1RX51 | 2.09 | 0.108 |  |  |  | B4E1G1 | G3HDU1 | L9KVN3 |
|  |  | F1SD23 | 2.02 | 0.136 |  |  |  | D2HJU3 | G3N0W8 | M1EAY6 |
|  |  | F1SD73 | 2.41 | 0.185 |  |  |  | D2WL18 | G3RJ38 | M3VH35 |
|  |  | F1SI48 | 2.98 | 0.187 |  |  |  | D4A3X0 | G3TA18 | M3VYT4 |
|  |  | F1SJR8 | 2.72 | 0.062 |  |  |  | E2RIP2 | G3TA49 | M3X7V2 |
|  |  | F1SNU4 | 2.43 | 0.122 |  |  |  | E2RSF2 | G3TDU0 | M3XTQ6 |
|  |  | F6Q903 | 2.08 | 0.088 |  |  |  | F1PPZ7 | G3TKK9 | M3Y757 |
|  |  | F6U6H7 | 6.54 | 0.101 |  |  |  | F1PTL1 | G3TXG8 | O77735 |
|  |  | F6W6G3 | 2.13 | 0.135 |  |  |  | F1RIW5 | G3X9J1 | P07633 |
|  |  | F6WBL7 | 2.01 | 0.300 |  |  |  | F1RJP8 | G5C9Y1 | P12273 |
|  |  | F6XF06 | 2.55 | 0.144 |  |  |  | F1RLQ5 | G7PWS5 | P50447 |
|  |  | F6YZV6 | 3.19 | 0.373 |  |  |  | F1RLR8 | G9KB37 | Q06AA3 |
|  |  | F7CSJ4 | 2.49 | 0.014 |  |  |  | F1RQN4 | H0UTP6 | Q0GAA4 |
|  |  | F7I2W7 | 2.07 | 0.330 |  |  |  | F1RU41 | H0WIC5 | Q0QEN9 |
|  |  | G1L321 | 4.34 | 0.399 |  |  |  | F1RVA1 | H0X4W0 | Q15KJ1 |
|  |  | G1MCU6 | 2.29 | 0.125 |  |  |  | F1RWK0 | H0XAC3 | Q1RMM8 |
|  |  | G1PW22 | 2.56 | 0.130 |  |  |  | F1RZQ8 | H0Y0B5 | Q2KHZ9 |
|  |  | G3QRD8 | 2.07 | 0.064 |  |  |  | F1S1G1 | H2NT61 | Q2TBI4 |
|  |  | G3SQ12 | 2.05 | 0.260 |  |  |  | F1S5Y7 | H2Q7H4 | Q31071 |
|  |  | G3TZD9 | 2.13 | 0.365 |  |  |  | F1S8U4 | H2R7U9 | Q3THQ5 |
|  |  | G5ALY4 | 2.01 | 0.312 |  |  |  | F1S912 | H6S1N5 | Q4R567 |
|  |  | G7MFY5 | 2.21 | 0.102 |  |  |  | F1S9I0 | I3L8X7 | Q58FF8 |
|  |  | H0WGT6 | 3.86 | 0.085 |  |  |  | F1SCY4 | I3LBW6 | Q5MX78 |
|  |  | H0XTM2 | 2.08 | 0.117 |  |  |  | F1SDP0 | I3LJE7 | Q5RJR9 |
|  |  | I3L5E9 | 2.12 | 0.065 |  |  |  | F1SEJ4 | I3LJW5 | Q5U1Y4 |
|  |  | I3L949 | 2.03 | 0.147 |  |  |  | F1SFG7 | I3LND3 | Q61344 |
|  |  | I3LK80 | 3.54 | 0.011 |  |  |  | F1SJ65 | I3LNV3 | Q641X5 |
|  |  | I3LS74 | 2.34 | 0.204 |  |  |  | F1SJU4 | I3LPB8 | Q8BFZ3 |
|  |  | K7GL27 | 2.92 | 0.358 |  |  |  | F1SM67 | I3LRS8 | Q9N0Y6 |
|  |  | K9J036 | 2.03 | 0.364 |  |  |  | F1SN95 | I3LZB1 | S4S5P7 |
|  |  | L5M286 | 2.22 | 0.006 |  |  |  | F1SSV5 | I3MFH2 | S7P5T2 |
|  |  | L5M7R9 | 3.07 | 0.088 |  |  |  | F1SSW3 | I3MFQ5 | S7P8I2 |
|  |  | L8AXK3 | 2.11 | 0.177 |  |  |  | F1SUH8 | I3MW48 | S7Q4C0 |
|  |  | L8B180 | 2.19 | 0.271 |  |  |  | F6RME4 | K7CKI3 | S9YCV0 |
|  |  | L9KR67 | 2.40 | 0.419 |  |  |  | F6S375 | K7EQ77 | S9YQH0 |
|  |  | M3X850 | 2.04 | 0.079 |  |  |  | F6TYW4 | K7ESX5 | U3F1R2 |
|  |  | P10173 | 3.32 | 0.021 |  |  |  | F6UR98 | K7GNQ2 | U6CZB6 |
|  |  | P80015 | 2.77 | 0.245 |  |  |  | F6UXQ1 | K9IVC2 | U6DBQ2 |
|  |  | Q2NNI9 | 2.27 | 0.329 |  |  |  | F6XI41 | K9J1X2 | U6DIF8 |
|  |  | Q6QR67 | 2.59 | 0.068 |  |  |  | F7BSA8 | K9J4W0 | U6DQU3 |
|  |  | Q6YT39 | 2.12 | 0.340 |  |  |  | F7F6K6 | K9KCX0 | U6DVI5 |
|  |  | Q9N251 | 2.10 | 0.261 |  |  |  | F7FK95 | K9KEM4 | W5NQX4 |
|  |  | S7NM73 | 2.39 | 0.066 |  |  |  | F7G117 | K9KF86 | W5NWM5 |
|  |  | S9Y253 | 2.19 | 0.376 |  |  |  | F7HSV9 | L5JP94 | W5PIG6 |
|  |  | W5QAH5 | 2.36 | 0.122 |  |  |  | F7I6G8 | L5K2L1 | W5Q0G8 |
|  |  |  |  |  |  |  |  |  |  |  |
| sham | baseline | A0A024RAM4 | 6.71 | 0.141 | I3LI20 | 2.13 | 0.094 | A0A024CBD6 | G1TP34 | L9L7C0 |
|  |  | B3CL07 | 3.15 | 0.476 | I3LK59 | 2.55 | 0.187 | A0A024R8D7 | G3HP24 | M1EJM9 |
|  |  | B6A7R0 | 2.04 | 0.254 | I3LLI0 | 5.72 | 0.364 | A0A091CK67 | G3R546 | M1ENF3 |
|  |  | D6BV38 | 2.10 | 0.166 | I3LRI1 | 6.69 | 0.078 | A0A096N7U8 | G3RJ38 | M3TYL1 |
|  |  | E2RIP2 | 2.02 | 0.415 | I3LSR0 | 2.01 | 0.284 | A6QPB5 | G3TA18 | M3UZ72 |
|  |  | E2RQF6 | 2.18 | 0.011 | I3LUN1 | 2.75 | 0.269 | A8DSD5 | G3U808 | M3VK46 |
|  |  | E5RGR0 | 2.83 | 0.201 | I3M9H9 | 2.72 | 0.314 | B5ATG0 | G3VGN2 | M3W219 |
|  |  | F1N2V9 | 2.73 | 0.079 | I3MFK6 | 3.98 | 0.334 | D2HLC5 | G5BWU8 | M3W520 |
|  |  | F1RNI5 | 2.04 | 0.229 | I3MFQ5 | 2.08 | 0.484 | D3GGC9 | G9L4Q8 | M3WAU6 |
|  |  | F1RRP1 | 2.24 | 0.240 | K6ZZM0 | 2.03 | 0.092 | D5K895 | H0UY16 | P11531 |
|  |  | F1RRR1 | 2.05 | 0.274 | K9IQI8 | 2.99 | 0.130 | E2RDW3 | H0W1J3 | Q08092 |
|  |  | F1RT83 | 2.18 | 0.060 | K9IUE2 | 2.61 | 0.378 | E2RHL0 | H0WNY6 | Q1RMM8 |
|  |  | F1S1J9 | 2.14 | 0.337 | L5L4V2 | 2.80 | 1.000 | F1P8K0 | H0X7X2 | Q2QC87 |
|  |  | F1S365 | 2.09 | 0.116 | L5M1M1 | 3.47 | 0.048 | F1PPZ7 | H0XBU7 | Q4JHS0 |
|  |  | F1S750 | 2.32 | 0.091 | L5M7R9 | 2.35 | 0.415 | F1RL20 | H0XD68 | Q4R567 |
|  |  | F1S9V7 | 5.57 | 0.073 | L5MJ38 | 15.66 | 0.232 | F1RP13 | H0XMU5 | Q5SUH7 |
|  |  | F1SA62 | 3.36 | 0.466 | L8IUT6 | 2.48 | 0.265 | F1RQW7 | H0Y0B5 | Q71DI1 |
|  |  | F1SEC0 | 2.31 | 0.150 | L8XZK5 | 2.45 | 0.380 | F1RR89 | H2NHD6 | Q95KL4 |
|  |  | F1SFE3 | 2.76 | 0.338 | L8Y053 | 2.08 | 0.356 | F1RVE8 | H7BWZ1 | Q9D6N4 |
|  |  | F1SHP6 | 2.26 | 0.122 | L8YEE5 | 2.36 | 0.393 | F1RW61 | H9CWF1 | S7MBZ8 |
|  |  | F1SLI3 | 2.05 | 0.576 | L9KQ79 | 2.17 | 0.201 | F1RY70 | I3L889 | S7NJQ2 |
|  |  | F6QW29 | 2.12 | 0.293 | L9L9X1 | 2.26 | 0.508 | F1S066 | I3LBZ1 | S9XYQ1 |
|  |  | F6RGG7 | 3.94 | 0.110 | M3W291 | 2.13 | 0.409 | F1S179 | I3LEI2 | S9YX05 |
|  |  | F7HSV9 | 2.99 | 0.521 | M3W9Z3 | 2.14 | 0.525 | F1S2W6 | I3LEJ9 | T0M673 |
|  |  | F7I9A0 | 2.35 | 0.046 | M3XPN1 | 2.04 | 0.219 | F1S4V8 | I3LGM5 | T0MFV8 |
|  |  | F8VU34 | 2.24 | 0.628 | M3Z7R1 | 2.20 | 0.152 | F1S6C3 | I3LGR2 | U3DN86 |
|  |  | F8W0P7 | 2.46 | 0.078 | P07633 | 2.41 | 0.329 | F1SD95 | I3LHU9 | U3U798 |
|  |  | G1MCU6 | 2.06 | 0.128 | P31151 | 3.70 | 1.000 | F1SJP9 | I3LJS5 | U6CU57 |
|  |  | G1PW22 | 2.03 | 0.310 | Q0KIY2 | 2.76 | 0.400 | F1SKA8 | I3LPU4 | U6DQU3 |
|  |  | G1SUY8 | 2.69 | 0.217 | Q1T7A9 | 2.11 | 0.422 | F1SPB1 | I3LQU0 | W5NQX4 |
|  |  | G3H2T7 | 2.65 | 0.254 | Q4R3G2 | 3.03 | 0.065 | F1SSM7 | I7KJP5 | W5NZC0 |
|  |  | G3T2W8 | 2.15 | 0.050 | Q58FF8 | 2.05 | 0.432 | F6Q4L6 | J9P1A8 | W5P5H0 |
|  |  | G3WSM9 | 2.85 | 0.274 | Q7YQ91 | 2.04 | 0.375 | F6UPL7 | K7E6Y2 |  |
|  |  | G5BEA9 | 3.57 | 0.280 | Q8BGH2 | 2.64 | 0.093 | F6UR98 | K7EIJ0 |  |
|  |  | H0WCW9 | 2.59 | 1.000 | S7N9H1 | 2.58 | 1.000 | F6YAV8 | K7GSD8 |  |
|  |  | H0WIB5 | 2.13 | 0.463 | S9WKW6 | 2.31 | 0.442 | F6ZME5 | K9K3F7 |  |
|  |  | H0X9Z9 | 2.20 | 0.170 | T0NGV6 | 2.22 | 0.231 | F7BM46 | K9KCX0 |  |
|  |  | H0XAC3 | 2.58 | 0.266 | T0NJM7 | 2.05 | 0.167 | F7BPT4 | L5LV74 |  |
|  |  | H2Q026 | 2.30 | 0.373 | U3CLF7 | 2.40 | 0.255 | F7FWZ0 | L5MCZ0 |  |
|  |  | H2RCL4 | 3.11 | 0.231 | W5NXP5 | 2.53 | 0.026 | F7HUG4 | L8B149 |  |
|  |  | H9H6B2 | 2.10 | 1.000 | W5PIG6 | 7.00 | 0.055 | F7IIA3 | L8IKI5 |  |
|  |  | I0FRX6 | 2.02 | 0.097 | W5PQL7 | 2.80 | 0.468 | F8WGB3 | L8INU0 |  |
|  |  | I3LAH2 | 3.11 | 0.100 |  |  |  | G1M4Y1 | L8Y790 |  |
|  |  |  |  |  |  |  |  |  |  |  |
|  | early reperfusion | A0A061ILH1 | 2.61 | 0.278 | I3LU39 | 2.04 | 0.382 | A0A087WZ32 | H0UTP6 | U6DAP2 |
|  |  | A0A075B7H9 | 2.07 | 0.336 | J7LGZ1 | 2.23 | 0.445 | A0A091CWN0 | H0VVQ3 | U6DR74 |
|  |  | A0A091DLV5 | 2.66 | 0.102 | J9JHZ3 | 2.90 | 0.449 | A0A091DE69 | H0WM58 | V9HVZ6 |
|  |  | A1XQS6 | 2.32 | 0.088 | K7DCT5 | 2.19 | 0.311 | B2B9A1 | H2NT61 | W5PP04 |
|  |  | A2AAJ9 | 2.45 | 0.395 | K7ED20 | 2.92 | 0.160 | B2KHW1 | H2P4G9 | W5Q0G8 |
|  |  | B7Z591 | 2.63 | 0.065 | K7GM47 | 2.29 | 0.244 | B2MUB8 | H2QQI6 |  |
|  |  | D2HTU0 | 2.20 | 0.401 | K7GPQ7 | 2.29 | 0.317 | B7NZQ0 | I3L8N7 |  |
|  |  | D2I4T8 | 2.27 | 0.263 | K7GQB8 | 2.05 | 0.482 | D2HF58 | I3LB64 |  |
|  |  | D3ZAS1 | 2.68 | 0.432 | K7GR72 | 2.60 | 0.098 | D2HGN1 | I3LCX2 |  |
|  |  | E2I6M4 | 2.17 | 0.287 | K7ZMG1 | 2.49 | 0.088 | D2HJA7 | I3LCY2 |  |
|  |  | F1MHT1 | 2.48 | 0.019 | K9ISI8 | 3.53 | 0.116 | E5RH91 | I3LDW8 |  |
|  |  | F1RGY5 | 2.01 | 0.194 | K9IVC2 | 2.01 | 0.308 | E5RIT6 | I3LI44 |  |
|  |  | F1RTR6 | 2.16 | 0.053 | K9KF86 | 2.67 | 0.591 | F1M4A6 | I3LJL2 |  |
|  |  | F1RU84 | 2.02 | 0.007 | L5K6H8 | 2.80 | 0.119 | F1RGS2 | I3LND3 |  |
|  |  | F1RUH5 | 2.06 | 0.383 | L5KRP6 | 2.24 | 0.291 | F1RGV8 | I3LNT1 |  |
|  |  | F1RXC1 | 2.89 | 0.191 | L5KUF8 | 2.73 | 0.450 | F1RIP3 | I3LPH5 |  |
|  |  | F1RZP9 | 2.30 | 0.384 | L9KMR2 | 2.00 | 0.162 | F1RJN4 | I3LQM0 |  |
|  |  | F1S0L8 | 3.51 | 0.118 | L9KN90 | 2.18 | 0.420 | F1RME2 | I3LS97 |  |
|  |  | F1S870 | 2.01 | 0.086 | L9KZG3 | 2.24 | 0.257 | F1RPY1 | I3LUJ3 |  |
|  |  | F1S9K3 | 2.47 | 0.211 | L9KZL5 | 2.29 | 0.130 | F1RRM1 | I3LUL5 |  |
|  |  | F1SDC9 | 2.16 | 0.213 | M3WE04 | 2.70 | 0.093 | F1RVD2 | I3MNA1 |  |
|  |  | F1SFI6 | 2.07 | 0.025 | M3WRM3 | 4.74 | 0.037 | F1RY63 | K9J6K1 |  |
|  |  | F1SI48 | 3.88 | 0.071 | M3X1S5 | 2.26 | 0.209 | F1RYA3 | K9KBX5 |  |
|  |  | F1SNN9 | 2.67 | 0.002 | M3X4Q9 | 2.14 | 0.171 | F1S268 | K9KEM4 |  |
|  |  | F1SNU4 | 4.06 | 0.182 | P00008 | 4.07 | 0.018 | F1S429 | L5JP94 |  |
|  |  | F6S6Y5 | 2.62 | 0.452 | P02770 | 3.20 | 0.193 | F1S6G2 | L5K2L1 |  |
|  |  | F6WTC6 | 2.46 | 0.230 | P05124 | 2.11 | 0.124 | F1S850 | L8B0Y0 |  |
|  |  | F6Z147 | 2.21 | 0.061 | P09905 | 2.51 | 0.204 | F1S8U4 | L8ISB4 |  |
|  |  | F7GQN4 | 2.06 | 0.099 | P18650 | 2.81 | 0.132 | F1S939 | L8XZ39 |  |
|  |  | F7IFU5 | 2.17 | 0.037 | P41541 | 3.35 | 0.182 | F1SB76 | L8Y6R9 |  |
|  |  | G1LG30 | 2.67 | 0.073 | P51525 | 2.10 | 0.249 | F1SEJ4 | L8Y9W5 |  |
|  |  | G1M291 | 2.29 | 0.281 | P80015 | 2.27 | 0.334 | F1SJ65 | L9JEV2 |  |
|  |  | G1NVD8 | 2.77 | 0.398 | P80310 | 3.07 | 0.045 | F1SJI1 | L9KWZ5 |  |
|  |  | G1Q0L6 | 4.04 | 0.388 | Q1A602 | 2.21 | 0.335 | F1SKM6 | M3UYX8 |  |
|  |  | G3I1S5 | 3.27 | 0.124 | Q29307 | 2.03 | 0.088 | F1SM67 | M3WBE1 |  |
|  |  | G3M5S9 | 2.63 | 0.338 | Q2NNI9 | 3.02 | 0.171 | F1SN81 | M3WDI2 |  |
|  |  | G3QRD8 | 2.11 | 0.581 | Q3THQ5 | 2.80 | 0.413 | F1ST94 | M3Y4B9 |  |
|  |  | G3SFX0 | 2.17 | 0.461 | Q3UE43 | 4.72 | 0.019 | F6R511 | M3YY71 |  |
|  |  | G3T702 | 2.07 | 0.447 | Q6QMZ5 | 2.86 | 0.292 | F6R6I2 | M3Z5G5 |  |
|  |  | G3VP51 | 4.63 | 0.236 | Q863Z0 | 2.14 | 0.065 | F6R9F7 | P12273 |  |
|  |  | G3VYK9 | 2.17 | 0.465 | Q8SPS7 | 2.13 | 0.702 | F6VCE9 | P56941 |  |
|  |  | G5B864 | 2.80 | 0.301 | Q9BGI3 | 2.51 | 0.081 | F6YB76 | Q08094 |  |
|  |  | G5BL65 | 2.37 | 0.094 | R4HZ39 | 2.10 | 0.264 | F6ZDM5 | Q0Z8R0 |  |
|  |  | G7MXP5 | 2.91 | 0.219 | S7NER6 | 2.08 | 0.157 | F7A8M7 | Q31071 |  |
|  |  | G9K813 | 2.25 | 0.232 | S7NJK1 | 2.12 | 0.238 | F7AZI9 | Q49AN9 |  |
|  |  | G9KCY4 | 2.57 | 0.532 | S9XEW3 | 2.29 | 0.177 | G0Z3A2 | Q5U1Y4 |  |
|  |  | G9KL39 | 2.17 | 0.041 | S9Y253 | 3.27 | 0.323 | G1L321 | Q6PWT7 |  |
|  |  | H0V7W3 | 2.30 | 0.104 | T0MDV7 | 2.13 | 0.487 | G1LMW5 | Q8HIH2 |  |
|  |  | H0WZ57 | 2.22 | 0.253 | U6DPK0 | 4.51 | 0.323 | G3GZL7 | Q8MGX3 |  |
|  |  | I3L723 | 2.58 | 0.015 | W5P254 | 2.13 | 0.372 | G3RPH6 | Q95KR5 |  |
|  |  | I3L810 | 2.40 | 0.012 | W5PHJ7 | 4.84 | 0.282 | G3SL42 | Q9HA11 |  |
|  |  | I3L9N1 | 2.02 | 0.119 | W5PK38 | 2.66 | 0.130 | G3TKK9 | S7Q4C0 |  |
|  |  | I3LLQ8 | 4.14 | 0.300 | W5Q777 | 2.37 | 0.314 | G3UYV7 | S9XHD5 |  |
|  |  | I3LLT5 | 2.04 | 0.020 | W5QEJ5 | 2.03 | 0.172 | G3WAY9 | S9YCV0 |  |
|  |  | I3LNE1 | 2.25 | 0.045 | W5QFE1 | 2.07 | 0.181 | G5E7G6 | U3DSC3 |  |

All proteins having ≥2-fold higher expression between groups (remote ischemic preconditioning (RIPC)/sham) at baseline and at eary reperfusion or between time points (baseline/early reperfusion) with RIPC or with sham and those detected exclusively in one group or timepoint in porcine left ventricular biopsies after in-solution digestion. When proteins were exclusively detected in one group or timepoint, -fold higher expression and p-value are not indicated, -fold higher expression and p-value versus sham. All proteins were compared by unpaired (between RIPC and sham) and paired (between baseline and early reperfusion) Student's t-tests.

**Supplemental Table S6: All proteins having ≥2-fold higher phosphorylation between groups (remote ischemic preconditioning/sham) at baseline and at eary reperfusion or between time points (baseline/early reperfusion) with remote ischemic preconditioning or with sham in porcine left ventricular biopsies**

|  | increased phoshorylation with | protein ID | phosphorylation  site | - fold higher phosphorylation | p-value | protein ID | phosphorylation site | - fold higher phosphorylation | p-value | protein ID | phosphorylation site | proteins exclusively detected in one group or at one time point | |
| --- | --- | --- | --- | --- | --- | --- | --- | --- | --- | --- | --- | --- | --- |
|  |  |  |  |  |  |  |  |  |  |  |  | protein ID | phosphorylation site |
| baseline | RIPC | A9LM01 | 153 | 3.64 | 0.033 | F1SQV8 | 363 | 2.27 | 0.332 | B6VNT8 | 102;150 | F1RHL9 | 57;65;46 |
|  |  | A9LM01 | 211 | 3.64 | 0.033 | F1SQW0 | 2 | 2.02 | 0.215 | F1RHL9 | 411 | F1RNZ1 | 38 |
|  |  | B0KYV5 | 365;135 | 2.81 | 0.094 | F1SRW8 | 334 | 2.33 | 0.089 | F1RKW6 | 18 | F1RP31 | 276 |
|  |  | C1PIG4 | 393 | 2.44 | 0.362 | F1SSY1 | 193 | 2.92 | 0.343 | F1RPT5 | 338 | F1RRS8 | 753 |
|  |  | C1PIG4 | 392 | 3.24 | 0.235 | F1SSY1 | 194 | 3.08 | 0.322 | F1RVD4 | 528 | F1RSZ3 | 440 |
|  |  | F1RFF0 | 633 | 2.47 | 0.072 | F1STJ9 | 2 | 2.09 | 0.159 | F1RZW0 | 432;272 | F1RUD6 | 40 |
|  |  | F1RFX9 | 203 | 2.54 | 0.018 | F1SU55 | 893 | 2.98 | 0.164 | F1S5B0 | 444 | F1RVL5 | 406 |
|  |  | F1RGP1 | 1283 | 2.61 | 0.009 | F1SUP1 | 42 | 2.02 | 0.102 | F1S5B0 | 29 | F1RWF5 | 94;111 |
|  |  | F1RHE6 | 186 | 2.09 | 0.397 | F1SV11 | 618 | 2.13 | 0.095 | F1SC49 | 205 | F1RYQ4 | 323 |
|  |  | F1RHN2 | 913 | 2.39 | 0.078 | F1SVB4 | 82 | 2.52 | 0.124 | F2Z5Q6 | 234;231 | F1S1H3 | 492 |
|  |  | F1RII7 | 73 | 2.02 | 0.046 | F2Z5B6 | 174 | 2.22 | 0.092 | I3LTN6 | 526 | F1S252 | 493;564 |
|  |  | F1RIN7 | 220 | 2.26 | 0.371 | F2Z5P0 | 17 | 3.38 | 0.266 | I3LTN6 | 527 | F1S4J0 | 2 |
|  |  | F1RIN7 | 295 | 2.18 | 0.313 | F6PSL2 | 130 | 2.26 | 0.287 | K7GNJ7 | 3564;3566 | F1S554 | 512 |
|  |  | F1RIN7 | 198 | 3.14 | 0.125 | F6PSL2 | 14 | 2.78 | 0.039 | P07802 | 5;5 | F1S5H5 | 252;273 |
|  |  | F1RK23 | 38 | 4.58 | 0.162 | I3L594 | 173;193 | 2.98 | 0.355 | P11607 | 648 | F1S5R6 | 200 |
|  |  | F1RK23 | 34 | 2.27 | 0.096 | I3L594 | 174;194 | 2.34 | 0.135 | Q3ZD69 | 632 | F1S5R6 | 199 |
|  |  | F1RK48 | 527 | 3.65 | 0.031 | I3L5B0 | 592 | 3.34 | 0.022 | F1RG67 | 196 | F1S620 | 657 |
|  |  | F1RLA7 | 508 | 2.65 | 0.130 | I3L5D5 | 583 | 2.07 | 0.464 | F1RGU5 | 1460 | F1S6V3 | 927 |
|  |  | F1RMN3 | 525 | 2.28 | 0.509 | I3L759 | 138;180 | 2.89 | 0.212 | F1RHL9 | 291;299;280 | F1S7K3 | 281 |
|  |  | F1RPD7 | 726 | 3.37 | 0.191 | I3LAR5 | 127 | 2.21 | 0.136 | F1RKG8 | 52 | F1S7X2 | 83 |
|  |  | F1RQC5 | 510 | 2.21 | 0.003 | I3LAR5 | 31 | 2.23 | 0.020 | F1RTX9 | 1230 | F1SA60 | 2315 |
|  |  | F1RQQ7 | 91 | 2.09 | 0.391 | I3LCW3 | 1153 | 6.27 | 0.326 | F1RXZ6 | 267 | F1SBY5 | 1107 |
|  |  | F1RQY8 | 1031 | 2.46 | 0.081 | I3LCX2 | 420 | 3.07 | 0.009 | F1S156 | 250 | F1SDC7 | 189 |
|  |  | F1RRS8 | 693 | 2.24 | 0.411 | I3LD72 | 438 | 2.13 | 0.356 | F1S1E0 | 320 | F1SEN8 | 41 |
|  |  | F1RSM9 | 75 | 2.84 | 0.043 | I3LDR9 | 302;147 | 2.46 | 0.250 | F1S448 | 792 | F1SEN8 | 217 |
|  |  | F1RVG6 | 551 | 2.39 | 0.358 | I3LFX8 | 155;187 | 3.80 | 0.065 | F1S4P0 | 245 | F1SJR2 | 818 |
|  |  | F1RVG6 | 538 | 3.73 | 0.037 | I3LGC6 | 2381 | 2.27 | 0.038 | F1S6W7 | 577 | F1SJR2 | 956 |
|  |  | F1RVL5 | 390 | 4.27 | 0.060 | I3LGJ5 | 978 | 2.13 | 0.335 | F1SBY5 | 1272 | F1SJR2 | 1664 |
|  |  | F1RWF5 | 86;103 | 2.37 | 0.183 | I3LHS7 | 73 | 2.79 | 0.149 | F1SGF0 | 709 | F1SLS8 | 420 |
|  |  | F1RXZ2 | 63;88 | 2.02 | 0.220 | I3LIB5 | 815 | 6.79 | 0.335 | F1SJE4 | 118 | F1SNY5 | 58 |
|  |  | F1RYQ4 | 350 | 2.04 | 0.531 | I3LIW3 | 2 | 2.99 | 0.172 | F1SK49 | 118 | P79293 | 1764 |
|  |  | F1RYS9 | 173 | 2.42 | 0.150 | I3LJJ6 | 94 | 2.03 | 0.256 | F1SMN5 | 1888 | F1SV22 | 2121 |
|  |  | F1RZN9 | 84;84 | 5.15 | 0.113 | I3LJN3 | 17 | 2.32 | 0.179 | F1SU85 | 459 | F2Z5G3 | 102 |
|  |  | F1RZY7 | 582 | 2.24 | 0.155 | I3LKI8 | 121 | 2.25 | 0.333 | F1SUT6 | 466 | F6PSL2 | 18 |
|  |  | F1S156 | 351 | 9.36 | 0.258 | I3LLN4 | 300 | 2.60 | 0.140 | I3L806 | 496 | I3L746 | 117 |
|  |  | F1S3R5 | 147 | 2.08 | 0.226 | I3LMC4 | 208 | 3.17 | 0.280 | I3LCA5 | 1078;1278 | I3LAR5 | 421 |
|  |  | F1S4Z9 | 200 | 3.90 | 0.131 | I3LS02 | 94 | 2.33 | 0.001 | I3LCA5 | 1080;1280 | I3LBS8 | 810 |
|  |  | F1S5B0 | 499 | 2.61 | 0.230 | I3LTH8 | 119;125 | 5.62 | 0.273 | I3LJE2 | 645 | I3LDU9 | 105 |
|  |  | F1S5Z3 | 508 | 2.12 | 0.064 | I3LU02 | 1196;1521 | 2.43 | 0.062 | I3LNK9 | 340 | I3LHI4 | 417;858 |
|  |  | F1S7K5 | 237 | 2.20 | 0.503 | I3LV12 | 81 | 2.29 | 0.409 | I3LNV3 | 83 | I3LHK5 | 415 |
|  |  | F1S814 | 117 | 3.14 | 0.335 | K7GKB2 | 213;253;298 | 2.45 | 0.075 | I3LSM7 | 296 | I3LS02 | 98 |
|  |  | F1S814 | 115 | 4.46 | 0.152 | K7GL02 | 141;142 | 5.05 | 0.083 | P79293 | 712;1519;1518 | I3LVD1 | 263;276 |
|  |  | F1S870 | 152 | 2.68 | 0.257 | K7GMN8 | 201;231;232 | 2.31 | 0.138 | P02189 | 4 | K7GL02 | 106;107 |
|  |  | F1S8T1 | 707 | 2.11 | 0.138 | K7GRF6 | 96;189 | 2.47 | 0.168 | P11607 | 38 | K7GLA8 | 939;929 |
|  |  | F1SA85 | 634 | 2.24 | 0.294 | P00355 | 331;107 | 3.09 | 0.103 | P36887 | 339 | P79293 | 172;979;978 |
|  |  | F1SAX3 | 672;673;637 | 2.46 | 0.288 | P02540 | 31 | 2.50 | 0.122 | Q5GN48 | 315;316;320 | P02540 | 461 |
|  |  | F1SAX3 | 482;483;454 | 2.66 | 0.016 | P02540 | 23 | 2.14 | 0.008 | F1RFR2 | 432 | P11607 | 555 |
|  |  | F1SB54 | 47 | 3.69 | 0.005 | P02540 | 23 | 2.14 | 0.008 | F1RW49 | 145 | P79293 | 756 |
|  |  | F1SCU6 | 140 | 2.80 | 0.130 | P08059 | 533 | 2.35 | 0.577 | F1RY63 | 627 | Q3ZD69 | 19 |
|  |  | F1SDT0 | 146 | 3.33 | 0.114 | P08059 | 251 | 2.47 | 0.493 | F1SPM7 | 243;261 | Q3ZD69 | 277 |
|  |  | F1SEJ4 | 1216 | 2.06 | 0.391 | P61013 | 16 | 4.91 | 0.120 | I3L5N6 | 16 | Q5GN48 | 1438 |
|  |  | F1SEJ4 | 115 | 2.54 | 0.014 | P67872 | 209;229 | 3.29 | 0.068 | B6VNT8 | 151 | Q5GN48 | 3541;528;355 |
|  |  | F1SEN8 | 190 | 2.67 | 0.111 | P79293 | 186;993;992 | 3.59 | 0.233 | F1RII7 | 71 | Q684M5 | 106 |
|  |  | F1SEN8 | 44 | 3.15 | 0.002 | P79293 | 422 | 2.26 | 0.176 | F1RN27 | 53 | Q6Q781 | 509;441;419 |
|  |  | F1SGD2 | 197 | 2.16 | 0.060 | P79293 | 914;1721 | 2.05 | 0.146 | F1RPT5 | 778 |  |  |
|  |  | F1SH10 | 142 | 2.72 | 0.086 | P79293 | 210 | 3.90 | 0.134 | F1S6Q7 | 139 |  |  |
|  |  | F1SI09 | 24 | 3.89 | 0.101 | P79293 | 1288;1287 | 2.64 | 0.121 | F1SK09 | 76 |  |  |
|  |  | F1SI65 | 668 | 3.88 | 0.208 | P79293 | 800;1607 | 3.27 | 0.104 | F1SV89 | 303;400 |  |  |
|  |  | F1SIB9 | 431;428 | 2.77 | 0.113 | P79293 | 703;1510 | 3.18 | 0.053 | I3L8Q0 | 292 |  |  |
|  |  | F1SID7 | 282 | 3.73 | 0.323 | P79293 | 103 | 3.18 | 0.031 | I3LDQ9 | 88 |  |  |
|  |  | F1SIJ0 | 47 | 2.31 | 0.294 | P79293 | 823;1630 | 2.47 | 0.026 | I3LDQ9 | 89 |  |  |
|  |  | F1SK49 | 119 | 2.20 | 0.144 | P79293 | 605;1412 | 3.98 | 0.023 | I3LJ14 | 52 |  |  |
|  |  | F1SKP8 | 423 | 2.65 | 0.100 | Q1KYT0 | 176 | 2.09 | 0.183 | I3LJC8 | 95 |  |  |
|  |  | F1SKQ0 | 603 | 2.57 | 0.492 | Q29092 | 504 | 2.06 | 0.190 | I3LJE2 | 27 |  |  |
|  |  | F1SKQ0 | 605 | 2.57 | 0.492 | Q5GN48 | 3612;599;426 | 2.07 | 0.227 | P79293 | 782;781 |  |  |
|  |  | F1SKX3 | 69 | 2.30 | 0.280 | Q6Q781 | 36 | 3.73 | 0.033 | Q6Q781 | 143;75;53 |  |  |
|  |  | F1SLE3 | 92 | 2.62 | 0.228 | Q95266 | 331 | 4.61 | 0.428 | A4Z6H0 | 13 |  |  |
|  |  | F1SM39 | 1108 | 2.36 | 0.421 | Q95266 | 330 | 3.58 | 0.110 | B6VNT8 | 156;204 |  |  |
|  |  | F1SMG3 | 66 | 2.14 | 0.313 | Q99028 | 182;266 | 2.01 | 0.539 | F1RGH4 | 924 |  |  |
|  |  | F1SMN5 | 2037 | 2.03 | 0.093 | Q9GLG4 | 179 | 4.18 | 0.015 | F1RGI7 | 195 |  |  |
|  |  | F1SNQ1 | 613 | 2.54 | 0.225 |  |  |  |  | F1RHF0 | 112 |  |  |
|  |  |  |  |  |  |  |  |  |  |  |  |  |  |
|  | sham | F1RFI1 | 312 | 2.42 | 0.091 | I3LAR5 | 141 | 3.23 | 0.087 | A5A769 | 64 | F1SKX3 | 42 |
|  |  | F1RFX9 | 205 | 2.36 | 0.071 | I3LAR5 | 580 | 2.07 | 0.148 | A9LM01 | 104;46;90 | F1SKZ8 | 86 |
|  |  | F1RFX9 | 202 | 2.21 | 0.113 | I3LAR5 | 111 | 3.33 | 0.261 | B6CVL5 | 92;88 | F1SL54 | 859 |
|  |  | F1RGH4 | 776 | 4.28 | 0.033 | I3LCC0 | 579 | 3.40 | 0.231 | D4P3C2 | 281 | F1SLI3 | 581 |
|  |  | F1RGH4 | 217 | 2.33 | 0.100 | I3LCL0 | 371 | 2.11 | 0.081 | F1RFY6 | 1092 | F1SLR5 | 1535 |
|  |  | F1RGK5 | 247 | 2.50 | 0.199 | I3LCL0 | 369 | 3.23 | 0.169 | F1RGE7 | 282 | F1SM75 | 1370;1174 |
|  |  | F1RGK5 | 246 | 2.35 | 0.340 | I3LCW3 | 1211 | 4.35 | 0.108 | F1RGH4 | 812;581 | F1SM86 | 444;463 |
|  |  | F1RHA0 | 303;339 | 2.01 | 0.065 | I3LCW3 | 2563 | 2.16 | 0.414 | F1RGI7 | 768 | F1SM86 | 488;461 |
|  |  | F1RHL9 | 433 | 5.01 | 0.150 | I3LDR9 | 216;61 | 2.33 | 0.206 | F1RGN8 | 124 | F1SMQ5 | 324 |
|  |  | F1RHM3 | 2267 | 8.80 | 0.002 | I3LDR9 | 388;232 | 3.78 | 0.449 | F1RGP4 | 302 | F1SMQ7 | 327 |
|  |  | F1RIP6 | 366 | 3.34 | 0.011 | I3LFI3 | 318 | 3.73 | 0.243 | F1RGQ6 | 460 | F1SN53 | 18 |
|  |  | F1RIT6 | 360;344 | 3.13 | 0.115 | I3LFI3 | 20 | 2.06 | 0.418 | F1RH92 | 145;230;234 | F1SNF8 | 1275 |
|  |  | F1RJU6 | 694 | 6.02 | 0.102 | I3LG67 | 117 | 2.91 | 0.073 | F1RH92 | 496; 225 | F1SNW4 | 161;49 |
|  |  | F1RJV5 | 145 | 2.92 | 0.302 | I3LGJ5 | 1373 | 2.81 | 0.178 | F1RH92 | 225 | F1SP54 | 123 |
|  |  | F1RK00 | 874 | 3.24 | 0.017 | I3LH91 | 228 | 2.58 | 0.419 | F1RHF0 | 110 | F1SP81 | 324 |
|  |  | F1RK48 | 63 | 2.74 | 0.156 | I3LI59 | 45 | 2.43 | 0.051 | F1RHG1 | 1253 | F1SPN5 | 53 |
|  |  | F1RKH4 | 24 | 2.35 | 0.081 | I3LIC8 | 292 | 2.11 | 0.451 | F1RHL9 | 151;159;140;132 | F1SQ46 | 286;354;45 |
|  |  | F1RKX9 | 184 | 2.40 | 0.090 | I3LII3 | 57 | 2.16 | 0.183 | F1RHU2 | 88;92 | F1SQK1 | 187;309 |
|  |  | F1RKX9 | 185 | 3.13 | 0.289 | I3LJU8 | 398 | 3.13 | 0.248 | F1RHU2 | 211 | F1SQP0 | 729 |
|  |  | F1RLJ4 | 76 | 2.20 | 0.078 | I3LKP0 | 722 | 3.51 | 0.082 | F1RI15 | 76;229;230 | F1SR84 | 18 |
|  |  | F1RLL1 | 148 | 2.97 | 0.235 | I3LKQ1 | 2 | 2.34 | 0.078 | F1RIF8 | 127 | F1SSL6 | 36 |
|  |  | F1RM62 | 16 | 3.59 | 0.019 | I3LLI0 | 5;5 | 4.76 | 0.305 | F1RIN7 | 372 | F1SSP0 | 15 |
|  |  | F1RM62 | 157 | 2.63 | 0.090 | I3LLN4 | 386;351 | 2.12 | 0.282 | F1RIP1 | 731 | F1ST03 | 1366 |
|  |  | F1RN59 | 956 | 3.57 | 0.143 | I3LLT6 | 495;492 | 3.26 | 0.318 | F1RJU6 | 512;700 | F1STQ3 | 788 |
|  |  | F1RN87 | 586 | 2.83 | 0.030 | I3LM30 | 474 | 2.25 | 0.102 | F1RK02 | 6 | F1SU55 | 440 |
|  |  | F1RNZ1 | 40 | 3.01 | 0.030 | I3LM30 | 1594 | 2.40 | 0.197 | F1RK48 | 473 | F1SUF2 | 172 |
|  |  | F1RP31 | 225 | 2.10 | 0.387 | I3LNK9 | 339;339 | 2.40 | 0.058 | F1RK90 | 1655 | F1SUP1 | 44 |
|  |  | F1RPS8 | 33;53 | 2.16 | 0.061 | I3LQF9 | 241 | 3.28 | 0.021 | F1RKJ3 | 13 | F1SUW3 | 73 |
|  |  | F1RRS8 | 536 | 4.00 | 0.086 | I3LR51 | 36 | 3.22 | 0.235 | F1RKX9 | 107 | F1SV22 | 3871;3211 |
|  |  | F1RRV6 | 2 | 3.02 | 0.005 | I3LRU5 | 476;487 | 2.04 | 0.030 | F1RLL1 | 602;429;565 | F1SV26 | 15 |
|  |  | F1RVG6 | 13 | 2.20 | 0.053 | I3LS66 | 246 | 2.16 | 0.190 | F1RLL1 | 603;430;566 | F1SV46 | 402;355;356 |
|  |  | F1RVL5 | 1088 | 7.28 | 0.077 | I3LSD1 | 112 | 2.05 | 0.205 | F1RLT0 | 841 | F2Z4X6 | 136 |
|  |  | F1RWD9 | 479;438 | 2.12 | 0.013 | I3LU02 | 585;903;816 | 2.12 | 0.126 | F1RM42 | 436 | F2Z4Z1 | 70 |
|  |  | F1RWD9 | 301;260 | 2.05 | 0.115 | I3LUL5 | 90 | 2.17 | 0.003 | F1RNP6 | 504 | F2Z5B6 | 162;215 |
|  |  | F1RWD9 | 745 | 3.06 | 0.186 | K7GMN8 | 262;292;293 | 2.87 | 0.324 | F1RNW3 | 142;143 | F2Z5H8 | 177 |
|  |  | F1RWK0 | 216 | 2.33 | 0.106 | K7GNW0 | 115 | 20.49 | 0.111 | F1RPS8 | 32;52 | I3L548 | 635;644 |
|  |  | F1RWW4 | 124 | 2.10 | 0.185 | O97580 | 241;279 | 2.47 | 0.029 | F1RQ09 | 108 | I3L5B0 | 11 |
|  |  | F1RZ24 | 520 | 2.98 | 0.414 | P02189 | 104 | 2.17 | 0.435 | F1RQC5 | 378 | I3L5C0 | 91 |
|  |  | F1RZ71 | 85 | 2.36 | 0.102 | P02540 | 433 | 5.06 | 0.295 | F1RQM9 | 2601 | I3L5H6 | 230 |
|  |  | F1RZU8 | 185 | 3.31 | 0.029 | P05383 | 264 | 2.21 | 0.271 | F1RQQ7 | 85 | I3L5S2 | 218 |
|  |  | F1RZW0 | 523;363 | 2.43 | 0.089 | P08059 | 109 | 2.88 | 0.222 | F1RQQ8 | 473 | I3L6C1 | 841 |
|  |  | F1RZW0 | 512;352 | 3.49 | 0.347 | P08059 | 532 | 2.27 | 0.455 | F1RR22 | 1038;1043 | I3L6E5 | 601 |
|  |  | F1S146 | 3732 | 3.24 | 0.028 | P11607 | 649 | 3.06 | 0.172 | F1RR78 | 2000;1595 | I3L6H7 | 218;220 |
|  |  | F1S1I0 | 36 | 2.65 | 0.035 | P19620 | 24 | 10.43 | 0.030 | F1RRJ1 | 295;296 | I3L6S5 | 313 |
|  |  | F1S1V5 | 397 | 2.49 | 0.526 | P21999 | 339 | 2.21 | 0.050 | F1RRM9 | 228 | I3L788 | 237 |
|  |  | F1S485 | 337;378;503 | 2.40 | 0.032 | P26234 | 567 | 2.03 | 0.358 | F1RRN1 | 587 | I3L8K4 | 182;113 |
|  |  | F1S4M3 | 975 | 2.05 | 0.190 | P29700 | 135 | 2.98 | 0.013 | F1RRS8 | 741 | I3L8Q0 | 829;1965 |
|  |  | F1S4N2 | 84 | 2.95 | 0.275 | P29700 | 323 | 2.24 | 0.072 | F1RS42 | 840;840 | I3L8Q0 | 255;800 |
|  |  | F1S4V5 | 396 | 4.66 | 0.022 | P79274 | 55 | 2.22 | 0.043 | F1RSR8 | 344 | I3L8X2 | 339 |
|  |  | F1S4V5 | 405 | 3.45 | 0.154 | P79293 | 1084;1891 | 76.72 | 0.048 | F1RSZ5 | 26 | I3L9T1 | 1612;1779 |
|  |  | F1S4V5 | 605 | 8.14 | 0.352 | P79293 | 255 | 9.87 | 0.061 | F1RT70 | 235;1220 | I3L9T1 | 2227;2267 |
|  |  | F1S4Z9 | 201 | 5.00 | 0.124 | P79293 | 70 | 3.84 | 0.257 | F1RU38 | 328 | I3L9T1 | 2503;265 |
|  |  | F1S554 | 370 | 2.10 | 0.006 | P79293 | 378 | 2.54 | 0.273 | F1RUR3 | 29;633 | I3L9T1 | 2704;2705 |
|  |  | F1S5L7 | 94 | 2.48 | 0.134 | P79293 | 445 | 3.39 | 0.283 | F1RUY2 | 470;144 | I3LA44 | 433 |
|  |  | F1S5M9 | 673 | 2.64 | 0.012 | Q06A94 | 6;13 | 2.31 | 0.020 | F1RVG6 | 731;797;67 | I3LA73 | 95;737 |
|  |  | F1S684 | 531 | 5.65 | 0.119 | Q06A98 | 26 | 3.56 | 0.185 | F1RVG6 |  | I3LAR5 | 63;415;360 |
|  |  | F1S7T1 | 871 | 2.70 | 0.165 | Q29101 | 296 | 3.46 | 0.544 | F1RVL5 | 89 | I3LB34 | 88;237 |
|  |  | F1S7W0 | 473 | 4.18 | 0.013 | Q2HYU1 | 148 | 3.29 | 0.102 | F1RW78 | 268 | I3LB64 | 520;520 |
|  |  | F1S7W0 | 1385 | 4.29 | 0.223 | Q2HYU1 | 368 | 2.28 | 0.208 | F1RWD9 | 739;741 | I3LBD6 | 192;21 |
|  |  | F1S7Y2 | 409 | 9.08 | 0.097 | Q2HYU2 | 775 | 2.16 | 0.045 | F1RWD9 | 245;231 | I3LC46 | 624 |
|  |  | F1S8L9 | 3 | 3.23 | 0.106 | Q2VTP6 | 9 | 3.72 | 0.002 | F1RWD9 | 520;479 | I3LCU9 | 63 |
|  |  | F1S8Y5 | 14 | 2.38 | 0.462 | Q3ZD69 | 22 | 3.19 | 0.059 | F1RWD9 | 304;263 | I3LCW3 | 1301;2380 |
|  |  | F1S995 | 859 | 2.87 | 0.016 | Q3ZD69 | 390 | 3.49 | 0.118 | F1RWE1 | 1163;1168 | I3LCW3 | 873;1077 |
|  |  | F1S995 | 564 | 3.13 | 0.077 | Q3ZD69 | 392 | 4.21 | 0.205 | F1RWF4 | 207;208 | I3LCW3 | 1310;1371 |
|  |  | F1S995 | 855 | 2.77 | 0.077 | Q3ZD69 | 143 | 2.15 | 0.279 | F1RWK0 | 461 | I3LEP9 | 319;333 |
|  |  | F1S995 | 488 | 6.27 | 0.078 | Q53DY5 | 2 | 6.81 | 0.081 | F1RWW4 | 171;183;41 | I3LEZ7 | 54 |
|  |  | F1S995 | 556 | 3.91 | 0.426 | Q53DY5 | 37 | 3.67 | 0.242 | F1RWX6 | 581 | I3LFQ5 | 103;778 |
|  |  | F1S9D1 | 413 | 2.24 | 0.036 | Q5GN48 | 3489;476 | 2.36 | 0.321 | F1RWZ2 | 236;239 | I3LG67 | 119;149 |
|  |  | F1S9W8 | 461 | 2.34 | 0.010 | Q5S1U1 | 84 | 3.05 | 0.418 | F1RYA4 | 47 | I3LGE5 | 758 |
|  |  | F1SA60 | 1240 | 2.13 | 0.013 | Q6S4N2 | 633 | 3.04 | 0.187 | F1RYL9 | 689 | I3LGJ5 | 1371 |
|  |  | F1SBU9 | 4 | 2.34 | 0.062 | Q8WNW3 | 665 | 2.08 | 0.158 | F1RYP5 | 825 | I3LGU7 | 182;186 |
|  |  | F1SBY5 | 1083 | 2.83 | 0.049 | Q95266 | 337 | 2.82 | 0.066 | F1RYQ4 | 299 | I3LHY2 | 211;214 |
|  |  | F1SBY5 | 1182 | 3.12 | 0.297 | Q95283 | 58 | 2.48 | 0.332 | F1RZ24 | 504 | I3LIB5 | 818 |
|  |  | F1SC49 | 1212 | 3.67 | 0.042 | Q9TUZ0 | 699 | 2.11 | 0.375 | F1RZD0 | 211 | I3LIC8 | 331 |
|  |  | F1SC51 | 91 | 2.53 | 0.137 |  |  |  |  | F1RZK8 | 456 | I3LJ91 | 42 |
|  |  | F1SDA4 | 643 | 2.65 | 0.170 |  |  |  |  | F1RZU8 | 186 | I3LKP0 | 603 |
|  |  | F1SDT0 | 101 | 3.57 | 0.008 |  |  |  |  | F1S0D6 | 111 | I3LLX3 | 467 |
|  |  | F1SE25 | 66 | 2.02 | 0.031 |  |  |  |  | F1S0W4 | 388 | I3LM30 | 2469 |
|  |  | F1SE25 | 54 | 2.80 | 0.305 |  |  |  |  | F1S146 | 1406 | I3LM43 | 374 |
|  |  | F1SEN8 | 267 | 12.57 | 0.012 |  |  |  |  | F1S2J5 | 352 | I3LMB2 | 600 |
|  |  | F1SEN8 | 116 | 2.36 | 0.474 |  |  |  |  | F1S2N4 | 534 | I3LMJ1 | 88 |
|  |  | F1SES6 | 209 | 2.47 | 0.444 |  |  |  |  | F1S2V7 | 5 | I3LMM6 | 152 |
|  |  | F1SGJ3 | 486 | 2.00 | 0.055 |  |  |  |  | F1S3M9 | 786;235 | I3LPF0 | 198 |
|  |  | F1SH84 | 420 | 3.18 | 0.018 |  |  |  |  | F1S3P0 | 93;95 | I3LPS9 | 33 |
|  |  | F1SID1 | 403 | 2.57 | 0.079 |  |  |  |  | F1S409 | 606 | I3LQ69 | 469 |
|  |  | F1SIW8 | 609 | 4.51 | 0.003 |  |  |  |  | F1S459 | 206 | I3LQ99 | 60 |
|  |  | F1SJC2 | 40 | 3.25 | 0.068 |  |  |  |  | F1S485 | 546;578 | I3LRZ4 | 346;360;377 |
|  |  | F1SJP2 | 377 | 2.57 | 0.103 |  |  |  |  | F1S485 | 475;784 | I3LSF0 | 461 |
|  |  | F1SJR2 | 542 | 2.98 | 0.063 |  |  |  |  | F1S485 | 784 | I3LSN9 | 64;458 |
|  |  | F1SLY4 | 155 | 2.06 | 0.006 |  |  |  |  | F1S4P0 | 276 | I3LU70 | 135 |
|  |  | F1SLY4 | 393 | 2.06 | 0.414 |  |  |  |  | F1S4V5 | 726 | I3LUE7 | 328;335 |
|  |  | F1SM75 | 82 | 2.55 | 0.025 |  |  |  |  | F1S4Z9 | 101 | I3LUY9 | 330;934 |
|  |  | F1SM75 | 1152;956 | 4.08 | 0.318 |  |  |  |  | F1S535 | 295 | K7GKF7 | 49;51 |
|  |  | F1SM75 | 116 | 2.90 | 0.352 |  |  |  |  | F1S5B0 | 637 | K7GM70 | 382;257 |
|  |  | F1SM86 | 421 | 4.65 | 0.034 |  |  |  |  | F1S5F9 | 2637;2639 | K7GMJ6 | 177;153;159 |
|  |  | F1SMD7 | 2 | 2.51 | 0.033 |  |  |  |  | F1S5R6 | 251;824 | K7GMN8 | 264;294;295 |
|  |  | F1SMG3 | 65 | 2.84 | 0.040 |  |  |  |  | F1S655 | 338;339 | K7GN99 | 82;82 |
|  |  | F1SMN1 | 72 | 2.07 | 0.256 |  |  |  |  | F1S6K5 | 66 | K7GNJ7 | 2594 |
|  |  | F1SMN5 | 1484 | 4.46 | 0.210 |  |  |  |  | F1S6R2 | 213 | K7GNJ7 | 3756;3758;83 |
|  |  | F1SMZ7 | 70;70 | 2.50 | 0.196 |  |  |  |  | F1S786 | 404;481 | K7GNZ0 | 28;133 |
|  |  | F1SN53 | 24 | 2.67 | 0.062 |  |  |  |  | F1S7W0 | 1474 | K7GNZ3 | 161 |
|  |  | F1SN99 | 2664 | 11.53 | 0.127 |  |  |  |  | F1S814 | 507 | K7GP58 | 264;268 |
|  |  | F1SNQ1 | 866 | 2.33 | 0.478 |  |  |  |  | F1S889 | 84;93 | K7GPA3 | 20 |
|  |  | F1SP82 | 34 | 49.18 | 0.164 |  |  |  |  | F1S939 | 765 | K7GRG2 | 73;73 |
|  |  | F1SPI2 | 37 | 2.04 | 0.247 |  |  |  |  | F1S9D1 | 704 | K7GS92 | 72;400 |
|  |  | F1SQK1 | 16 | 3.16 | 0.010 |  |  |  |  | F1S9W6 | 230;256 | O18734 | 128 |
|  |  | F1SQK1 | 367 | 2.14 | 0.313 |  |  |  |  | F1S9Z2 | 425 | P00336 | 238 |
|  |  | F1SQL3 | 306;191;200 | 2.86 | 0.620 |  |  |  |  | F1SA50 | 611 | P02540 | 59;60 |
|  |  | F1SRC5 | 658 | 2.48 | 0.198 |  |  |  |  | F1SB28 | 810 | P02543 | 56;426 |
|  |  | F1SS24 | 286 | 3.86 | 0.146 |  |  |  |  | F1SBF0 | 840 | P02543 | 419;420 |
|  |  | F1ST98 | 95 | 2.35 | 0.120 |  |  |  |  | F1SBY5 | 1274 | P02547 | 472;473 |
|  |  | F1SU55 | 722 | 2.60 | 0.013 |  |  |  |  | F1SC49 | 344;410 | P11708 | 242;179;74 |
|  |  | F1SU55 | 932 | 2.08 | 0.115 |  |  |  |  | F1SC49 | 1203 | P14287 | 214 |
|  |  | F1SUJ6 | 101 | 2.00 | 0.048 |  |  |  |  | F1SDA4 | 642 | P14287 | 210 |
|  |  | F1SUZ2 | 771 | 2.01 | 0.305 |  |  |  |  | F1SDC8 | 1402 | P26234 | 97 |
|  |  | F1SV46 | 115;78;79 | 2.81 | 0.092 |  |  |  |  | F1SDQ0 | 363;365 | P29269 | 19;19;19;24 |
|  |  | F2Z4Z1 | 71 | 2.86 | 0.238 |  |  |  |  | F1SDT0 | 97 | P62895 | 29;29 |
|  |  | F2Z523 | 137 | 2.22 | 0.026 |  |  |  |  | F1SDX6 | 60 | P67872 | 205;225 |
|  |  | F6PSL2 | 117 | 2.54 | 0.050 |  |  |  |  | F1SDY8 | 516 | P79293 | 706;1513;1512 |
|  |  | I3L548 | 618;627 | 3.76 | 0.050 |  |  |  |  | F1SEJ0 | 403;439 | P79293 | 351;1158;1157 |
|  |  | I3L548 | 676;685 | 7.59 | 0.112 |  |  |  |  | F1SEN8 | 282 | P79293 | 350;1157;1156 |
|  |  | I3L6D7 | 744;749 | 3.15 | 0.338 |  |  |  |  | F1SGD2 | 250 | Q0MUU2 | 47 |
|  |  | I3L7C4 | 713 | 2.84 | 0.120 |  |  |  |  | F1SGS6 | 306 | Q0MUU2 | 48 |
|  |  | I3L7C4 | 712 | 2.18 | 0.207 |  |  |  |  | F1SH92 | 225;223 | Q1KYT0 | 177 |
|  |  | I3L7S4 | 114; | 2.19 | 0.080 |  |  |  |  | F1SH92 | 705;701 | Q29092 | 306 |
|  |  | I3L816 | 103;104 | 2.01 | 0.045 |  |  |  |  | F1SI65 | 2083;2096 | Q29101 | 326 |
|  |  | I3L8D7 | 598 | 2.52 | 0.299 |  |  |  |  | F1SI69 | 148 | Q2HYU1 | 159 |
|  |  | I3L8Q0 | 274 | 3.07 | 0.318 |  |  |  |  | F1SID1 | 429 | Q2HYU1 | 417 |
|  |  | I3L8Q0 | 276 | 2.13 | 0.345 |  |  |  |  | F1SID7 | 1018 | Q3ZD69 | 301 |
|  |  | I3L8Q0 | 259 | 2.76 | 0.376 |  |  |  |  | F1SIK9 | 406 | Q5S1U1 | 178 |
|  |  | I3L9I6 | 581 | 2.16 | 0.051 |  |  |  |  | F1SJ90 | 281 | Q68J42 | 552 |
|  |  | I3L9I6 | 594 | 3.20 | 0.578 |  |  |  |  | F1SJG2 | 323 | Q68J42 | 554 |
|  |  | I3L9K5 | 181 | 2.31 | 0.003 |  |  |  |  | F1SJR2 | 502;533 | Q6DUB7 | 25 |
|  |  | I3L9T1 | 3246;3251 | 2.29 | 0.229 |  |  |  |  | F1SJR2 | 1395;1803 | Q6Q781 | 312;244;222 |
|  |  | I3LAK5 | 588;643 | 6.36 | 0.146 |  |  |  |  | F1SJR2 | 134;213 | Q7YS99 | 15;118;198 |
|  |  | I3LAR5 | 119 | 2.35 | 0.029 |  |  |  |  | F1SK12 | 25;1297;1335 | Q95266 | 490 |
|  |  | I3LAR5 | 319 | 8.45 | 0.060 |  |  |  |  | F1SKB1 | 361 | Q95266 | 276;277 |
|  |  | I3LAR5 | 55 | 2.09 | 0.086 |  |  |  |  | F1SKD6 | 450 | Q9MYT8 | 68 |
|  |  |  |  |  |  |  |  |  |  |  |  |  |  |
| early reperfusion | RIPC | A1XQV4 | 271 | 2.40 | 0.321 | F1SK44 | 86 | 2.20 | 0.245 | A5D9P0 | 140 |  |  |
|  |  | A1XQV4 | 283 | 2.78 | 0.277 | F1SK49 | 119 | 2.73 | 0.308 | A5GFS8 | 159 |  |  |
|  |  | A4GR69 | 109 | 2.92 | 0.289 | F1SKP8 | 423 | 2.00 | 0.101 | B6VNT8;P68137 | 103;151 |  |  |
|  |  | A5GFS8 | 158 | 5.34 | 0.009 | F1SKX3 | 69 | 2.10 | 0.275 | B6VNT8;P68137 | 102;150 |  |  |
|  |  | A9LM01 | 211;153 | 3.17 | 0.025 | F1SKY2 | 22 | 2.06 | 0.322 | F1REW5 | 1000 |  |  |
|  |  | B5U335 | 84 | 4.63 | 0.067 | F1SKZ8 | 92 | 2.14 | 0.211 | F1RFR2 | 432 |  |  |
|  |  | B6VNT8 | 370 | 2.46 | 0.016 | F1SLF0 | 219 | 3.25 | 0.010 | F1RG85 | 203 |  |  |
|  |  | B6VNT8 | 93;91 | 2.93 | 0.100 | F1SLI3 | 736 | 5.80 | 0.129 | F1RGH4 | 556 |  |  |
|  |  | B6VNT8 | 241;241;193 | 2.19 | 0.085 | F1SLI3 | 629 | 2.19 | 0.066 | F1RGH4 | 497 |  |  |
|  |  | B6VNT8 | 54;52 | 3.06 | 0.028 | F1SLI3 | 642 | 2.65 | 0.015 | F1RHG1 | 1252 |  |  |
|  |  | B6VNT8 | 251;203 | 3.57 | 0.013 | F1SLY4 | 15 | 5.14 | 0.011 | F1RHM3 | 1940 |  |  |
|  |  | B6VNT8 | 277;325 | 4.60 | 0.005 | F1SM39 | 1108 | 5.03 | 0.021 | F1RHM3 | 2908 |  |  |
|  |  | D2SQP3 | 95 | 3.05 | 0.205 | F1SM75 | 84 | 2.22 | 0.137 | F1RHU2 | 211 |  |  |
|  |  | F1RFB2 | 1155 | 3.48 | 0.024 | F1SM75 | 116 | 3.17 | 0.124 | F1RHU2 | 212 |  |  |
|  |  | F1RFX9 | 19 | 2.37 | 0.372 | F1SM75 | 418;316 | 2.47 | 0.012 | F1RHU2 | 88 |  |  |
|  |  | F1RFY6 | 1033 | 3.36 | 0.288 | F1SM86 | 89 | 3.03 | 0.069 | F1RHU2 | 92 |  |  |
|  |  | F1RG61 | 682 | 3.02 | 0.003 | F1SMB6 | 137 | 2.78 | 0.410 | F1RII7 | 71 |  |  |
|  |  | F1RG85 | 207 | 3.21 | 0.123 | F1SMN1 | 47 | 7.33 | 0.128 | F1RIN7 | 372 |  |  |
|  |  | F1RGH4 | 806 | 2.06 | 0.105 | F1SMN5 | 2597 | 2.27 | 0.217 | F1RIN7 | 170 |  |  |
|  |  | F1RGH4 | 304 | 3.23 | 0.005 | F1SNF8 | 1275 | 3.56 | 0.004 | F1RIN7 | 296 |  |  |
|  |  | F1RGI7 | 188 | 2.67 | 0.111 | F1SNQ1 | 866 | 2.15 | 0.219 | F1RIU9 | 45 |  |  |
|  |  | F1RGP1 | 1283 | 2.27 | 0.193 | F1SNQ1 | 613 | 6.60 | 0.157 | F1RK02 | 6 |  |  |
|  |  | F1RGP1 | 14 | 3.24 | 0.058 | F1SNW4 | 161 | 4.63 | 0.033 | F1RK23 | 38 |  |  |
|  |  | F1RHC6 | 55 | 2.36 | 0.202 | F1SNX8 | 55 | 2.18 | 0.077 | F1RKY1 | 37 |  |  |
|  |  | F1RHE6 | 186 | 3.09 | 0.024 | F1SP24 | 17 | 2.06 | 0.130 | F1RN75 | 191;357;395;400 |  |  |
|  |  | F1RHE6 | 188 | 3.72 | 0.014 | F1SP32 | 155 | 2.54 | 0.023 | F1RPT5 | 778 |  |  |
|  |  | F1RHL9 | 435;411;588 | 4.82 | 0.025 | F1SP36 | 1138;1086 | 2.23 | 0.013 | F1RPY5 | 420 |  |  |
|  |  | F1RHM3 | 3933 | 2.04 | 0.548 | F1SPN5 | 53 | 3.23 | 0.016 | F1RQM2 | 62 |  |  |
|  |  | F1RHM3 | 1937 | 3.73 | 0.068 | F1SQK1 | 747 | 2.51 | 0.138 | F1RQM9 | 4609 |  |  |
|  |  | F1RHM3 | 2396 | 4.87 | 0.026 | F1SQK1 | 367 | 2.74 | 0.100 | F1RQM9 | 4610 |  |  |
|  |  | F1RHM3 | 2388 | 5.30 | 0.002 | F1SQK1 | 889 | 2.47 | 0.092 | F1RQQ7 | 91 |  |  |
|  |  | F1RHN2 | 640 | 2.25 | 0.139 | F1SQK1 | 457 | 2.08 | 0.055 | F1RR78 | 874 |  |  |
|  |  | F1RHN2 | 913 | 2.10 | 0.044 | F1SQK1 | 870 | 7.56 | 0.026 | F1RRD7 | 128 |  |  |
|  |  | F1RIC1 | 18 | 2.39 | 0.074 | F1SQK1 | 309 | 4.37 | 0.004 | F1RRN1 | 609 |  |  |
|  |  | F1RIC1 | 47 | 5.90 | 0.017 | F1SQL3 | 342 | 2.33 | 0.121 | F1RS45 | 1513 |  |  |
|  |  | F1RIN7 | 198 | 3.04 | 0.081 | F1SQL3 | 308;193;202 | 2.60 | 0.044 | F1RS65 | 543;570 |  |  |
|  |  | F1RIN7 | 118 | 2.20 | 0.041 | F1SQS2 | 74 | 2.19 | 0.041 | F1RSR8 | 419 |  |  |
|  |  | F1RIP1 | 727 | 2.05 | 0.039 | F1SQW0 | 141 | 2.34 | 0.016 | F1RTX9 | 1230 |  |  |
|  |  | F1RIW3 | 260 | 2.73 | 0.268 | F1SRW8 | 334 | 3.92 | 0.014 | F1RVG6 | 882;154 |  |  |
|  |  | F1RIW3 | 223 | 2.15 | 0.031 | F1SS33 | 405;408 | 2.30 | 0.139 | F1RVG6 | 731 |  |  |
|  |  | F1RJI2 | 133 | 2.37 | 0.035 | F1SS61 | 952;616;141 | 2.35 | 0.002 | F1RVL5 | 406 |  |  |
|  |  | F1RJM2 | 67 | 2.32 | 0.016 | F1SS61 | 1379;1043;568 | 2.98 | 0.117 | F1RVZ0 | 596;604 |  |  |
|  |  | F1RJN0 | 217 | 3.26 | 0.051 | F1SS61 | 1370;559 | 3.11 | 0.093 | F1RW75 | 1511 |  |  |
|  |  | F1RK23 | 34 | 2.69 | 0.080 | F1SS64 | 1192;1192 | 3.12 | 0.075 | F1RWA1 | 407 |  |  |
|  |  | F1RK48 | 527 | 3.97 | 0.099 | F1SS64 | 1856;1856 | 2.71 | 0.018 | F1RWF5 | 94;111 |  |  |
|  |  | F1RK48 | 472 | 3.03 | 0.037 | F1SS77 | 59;67;62 | 5.18 | 0.004 | F1RXG7 | 10 |  |  |
|  |  | F1RK48 | 817 | 3.92 | 0.030 | F1ST81 | 299 | 2.85 | 0.122 | F1RY63 | 627 |  |  |
|  |  | F1RK48 | 851 | 2.85 | 0.022 | F1STF7 | 44 | 2.36 | 0.050 | F1RZB7 | 658 |  |  |
|  |  | F1RK48 | 785 | 3.70 | 0.014 | F1SU55 | 892 | 2.13 | 0.229 | F1RZQ6 | 112;76 |  |  |
|  |  | F1RKG9 | 324 | 2.36 | 0.264 | F1SU55 | 744 | 2.06 | 0.203 | F1RZV8 | 625 |  |  |
|  |  | F1RKH4 | 87 | 4.80 | 0.043 | F1SUH2 | 269 | 2.66 | 0.037 | F1S086 | 398;408 |  |  |
|  |  | F1RKJ3 | 11 | 2.02 | 0.055 | F1SV22 | 2102 | 2.05 | 0.010 | F1S087 | 81 |  |  |
|  |  | F1RLA7 | 508 | 4.56 | 0.023 | F2Z514 | 9 | 3.51 | 0.136 | F1S0D8 | 421 |  |  |
|  |  | F1RLE5 | 6 | 2.14 | 0.019 | F2Z5B6 | 261 | 2.28 | 0.178 | F1S0X4 | 539 |  |  |
|  |  | F1RM62 | 59;15 | 7.40 | 0.109 | F2Z5B6 | 252 | 2.30 | 0.035 | F1S269 | 196 |  |  |
|  |  | F1RM62 | 157 | 17.46 | 0.003 | F2Z5B6 | 61 | 2.10 | 0.012 | F1S2N4 | 534 |  |  |
|  |  | F1RMZ7 | 282 | 2.01 | 0.080 | F6PSL2 | 14 | 2.23 | 0.060 | F1S415 | 292 |  |  |
|  |  | F1RNP6 | 100 | 4.12 | 0.334 | I3L548 | 618;627 | 2.15 | 0.065 | F1S4P0 | 245 |  |  |
|  |  | F1RNW3 | 154 | 2.09 | 0.040 | I3L548 | 635;644 | 4.84 | 0.061 | F1S4V5 | 458 |  |  |
|  |  | F1RP31 | 225 | 2.21 | 0.238 | I3L548 | 729;738 | 3.09 | 0.047 | F1S5B0 | 444 |  |  |
|  |  | F1RPD7 | 726 | 4.75 | 0.140 | I3L594 | 173;193 | 4.13 | 0.047 | F1S5H5 | 252;273 |  |  |
|  |  | F1RPD7 | 574 | 2.17 | 0.038 | I3L594 | 174;194 | 3.33 | 0.003 | F1S5M9 | 677 |  |  |
|  |  | F1RPS7 | 66 | 2.08 | 0.119 | I3L5D4 | 23 | 8.72 | 0.013 | F1S5R6 | 200 |  |  |
|  |  | F1RQM2 | 64 | 2.40 | 0.106 | I3L5D5 | 583 | 6.01 | 0.099 | F1S5R6 | 199 |  |  |
|  |  | F1RQQ7 | 361 | 2.43 | 0.039 | I3L5D5 | 611 | 5.87 | 0.001 | F1S684 | 385 |  |  |
|  |  | F1RR22 | 1038;1043 | 2.88 | 0.182 | I3L5T3 | 155 | 3.46 | 0.048 | F1S786 | 406;483 |  |  |
|  |  | F1RR78 | 1031 | 3.36 | 0.131 | I3L6C1 | 841 | 2.79 | 0.113 | F1S7J6;F1SBU9 | 343;339 |  |  |
|  |  | F1RR78 | 2064 | 2.40 | 0.076 | I3L6Q5 | 2 | 2.46 | 0.069 | F1S7J6;F1SBU9 | 344;340 |  |  |
|  |  | F1RR78 | 1197 | 3.10 | 0.012 | I3L759 | 138;180 | 3.23 | 0.038 | F1S7K3 | 281 |  |  |
|  |  | F1RRE0 | 162 | 3.44 | 0.051 | I3L788 | 237 | 2.78 | 0.147 | F1S7X2 | 83 |  |  |
|  |  | F1RRN1 | 457 | 2.23 | 0.222 | I3L7U1 | 418 | 3.05 | 0.014 | F1S814 | 505 |  |  |
|  |  | F1RRS8 | 693 | 2.92 | 0.186 | I3L801 | 125 | 3.27 | 0.185 | F1S8A4 | 795 |  |  |
|  |  | F1RS36 | 632 | 2.19 | 0.134 | I3L8K4 | 107 | 2.00 | 0.262 | F1S8Y8 | 927 |  |  |
|  |  | F1RS42 | 840 | 11.82 | 0.407 | I3L8K4 | 164 | 2.47 | 0.005 | F1S995 | 490 |  |  |
|  |  | F1RSD1 | 60 | 2.02 | 0.180 | I3L8Q0 | 259;708;1548 | 3.59 | 0.041 | F1SA60 | 2315 |  |  |
|  |  | F1RT70 | 235 | 2.18 | 0.202 | I3L953 | 754 | 2.34 | 0.304 | F1SA85 | 634 |  |  |
|  |  | F1RTR6 | 468 | 2.04 | 0.149 | I3L9F5 | 435 | 3.45 | 0.001 | F1SAX3 | 219;220;189 |  |  |
|  |  | F1RU38 | 327 | 4.22 | 0.155 | I3L9K5 | 223 | 7.49 | 0.151 | F1SAX3 | 672;673;637 |  |  |
|  |  | F1RU40 | 604 | 2.25 | 0.308 | I3L9K5 | 214 | 2.30 | 0.032 | F1SB18 | 227 |  |  |
|  |  | F1RVD4 | 528 | 3.09 | 0.184 | I3L9K5 | 19 | 4.30 | 0.005 | F1SB63 | 551 |  |  |
|  |  | F1RVG6 | 797;67 | 2.04 | 0.235 | I3L9T1 | 1351;3108 | 2.52 | 0.040 | F1SBH1 | 197 |  |  |
|  |  | F1RVG6 | 387 | 2.39 | 0.169 | I3LA44 | 2 | 2.12 | 0.303 | F1SBQ0 | 76 |  |  |
|  |  | F1RVG6 | 253 | 4.16 | 0.162 | I3LAR5 | 421 | 3.99 | 0.361 | F1SBY5 | 1107 |  |  |
|  |  | F1RVG6 | 884;156 | 2.04 | 0.152 | I3LAR5 | 127 | 2.35 | 0.048 | F1SC49 | 1202 |  |  |
|  |  | F1RVG6 | 538 | 2.49 | 0.040 | I3LAR5 | 31 | 2.51 | 0.039 | F1SC51 | 131 |  |  |
|  |  | F1RVL5 | 390 | 2.20 | 0.347 | I3LAY6 | 16;80 | 2.31 | 0.014 | F1SCI4 | 186 |  |  |
|  |  | F1RVL5 | 89 | 2.34 | 0.228 | I3LAY6 | 95;159 | 8.69 | 0.002 | F1SDC7 | 189 |  |  |
|  |  | F1RVL5 | 364 | 3.60 | 0.006 | I3LBD0 | 254 | 2.43 | 0.023 | F1SDL5 | 394 |  |  |
|  |  | F1RVZ3 | 295 | 2.73 | 0.278 | I3LBS8 | 750 | 2.29 | 0.206 | F1SDV2 | 1067 |  |  |
|  |  | F1RW75 | 1031 | 2.49 | 0.241 | I3LC38 | 50;50 | 2.44 | 0.038 | F1SDY9 | 637 |  |  |
|  |  | F1RWC5 | 930 | 2.64 | 0.025 | I3LCU0 | 13;244 | 2.32 | 0.049 | F1SE30 | 888 |  |  |
|  |  | F1RWD9 | 1316;1186 | 2.27 | 0.392 | I3LCW3 | 2431 | 2.72 | 0.546 | F1SEN8 | 282 |  |  |
|  |  | F1RWD9 | 939;809 | 2.34 | 0.013 | I3LCW3 | 1370 | 2.68 | 0.017 | F1SEN8 | 115 |  |  |
|  |  | F1RWE1 | 1163;1168 | 2.43 | 0.216 | I3LCX2 | 420 | 3.79 | 0.001 | F1SF12 | 89 |  |  |
|  |  | F1RWW4 | 225 | 2.15 | 0.043 | I3LDR9 | 331 | 2.05 | 0.089 | F1SFZ8 | 977 |  |  |
|  |  | F1RWW4 | 123 | 3.41 | 0.001 | I3LDR9 | 446;290 | 2.98 | 0.085 | F1SFZ8 | 979 |  |  |
|  |  | F1RX74 | 29 | 2.05 | 0.329 | I3LDR9 | 215;60 | 2.47 | 0.057 | F1SGD2 | 82 |  |  |
|  |  | F1RX81 | 674;675 | 2.89 | 0.165 | I3LDS5 | 776;747;586 | 2.00 | 0.516 | F1SGF0 | 767 |  |  |
|  |  | F1RYA4 | 5 | 2.25 | 0.122 | I3LDS5 | 540;517;350 | 2.08 | 0.184 | F1SGJ6 | 148 |  |  |
|  |  | F1RYA4 | 125 | 2.74 | 0.062 | I3LDS5 | 1342;1305 | 2.28 | 0.105 | F1SH07 | 261 |  |  |
|  |  | F1RYQ4 | 300 | 2.81 | 0.006 | I3LDS5 | 273;278 | 4.53 | 0.053 | F1SHE5 | 869 |  |  |
|  |  | F1RYQ4 | 302 | 3.10 | 0.003 | I3LDS5 | 1297 | 2.72 | 0.038 | F1SI26 | 1391 |  |  |
|  |  | F1RYQ4 | 350 | 3.60 | 0.001 | I3LDU9 | 222 | 6.01 | 0.013 | F1SI85 | 481 |  |  |
|  |  | F1RYY6 | 237 | 2.05 | 0.187 | I3LDU9 | 95 | 2.61 | 0.006 | F1SIA4 | 74 |  |  |
|  |  | F1RZ24 | 22 | 3.09 | 0.046 | I3LE55 | 285 | 2.18 | 0.139 | F1SIB9 | 237 |  |  |
|  |  | F1RZC6 | 1288 | 2.31 | 0.068 | I3LEJ3 | 1018;1022 | 2.74 | 0.343 | F1SID1 | 495 |  |  |
|  |  | F1RZK8 | 456 | 2.67 | 0.572 | I3LEJ3 | 813;817 | 3.31 | 0.336 | F1SIZ4 | 242;247 |  |  |
|  |  | F1RZN9 | 85 | 2.20 | 0.007 | I3LEJ3 | 1053;1062 | 2.96 | 0.247 | F1SJR2 | 1804 |  |  |
|  |  | F1RZP9 | 824 | 3.59 | 0.021 | I3LEJ3 | 686;690 | 2.09 | 0.030 | F1SJX1 | 179 |  |  |
|  |  | F1RZQ6 | 195;159 | 2.38 | 0.173 | I3LFI3 | 20 | 2.39 | 0.104 | F1SK09 | 76 |  |  |
|  |  | F1RZR9 | 8 | 2.63 | 0.065 | I3LFV4 | 176 | 2.34 | 0.262 | F1SKX3 | 440 |  |  |
|  |  | F1RZU8 | 2603 | 2.20 | 0.491 | I3LG33 | 167 | 2.19 | 0.064 | F1SKX3 | 442 |  |  |
|  |  | F1RZW0 | 228;68 | 3.73 | 0.173 | I3LG67 | 144 | 2.66 | 0.079 | F1SLE3 | 92 |  |  |
|  |  | F1RZW0 | 89 | 2.31 | 0.170 | I3LG94 | 1117 | 3.30 | 0.003 | F1SLY4 | 45 |  |  |
|  |  | F1RZW0 | 739 | 2.92 | 0.037 | I3LGC6 | 2381 | 2.11 | 0.100 | F1SM00 | 359;313 |  |  |
|  |  | F1RZW0 | 443;283 | 2.11 | 0.013 | I3LGE5 | 1056 | 4.63 | 0.030 | F1SM75 | 224;122 |  |  |
|  |  | F1RZW0 | 163;3 | 2.26 | 0.010 | I3LH91 | 3 | 2.32 | 0.035 | F1SM75 | 353;251 |  |  |
|  |  | F1RZW0 | 328;168 | 3.89 | 0.005 | I3LHS7 | 96 | 2.28 | 0.168 | F1SM75 | 354;252 |  |  |
|  |  | F1RZY7 | 582 | 2.32 | 0.095 | I3LI59 | 45 | 2.51 | 0.068 | F1SM83 | 703 |  |  |
|  |  | F1S059 | 104;202 | 2.12 | 0.016 | I3LIB5 | 644 | 2.57 | 0.637 | F1SMN5 | 955 |  |  |
|  |  | F1S0V1 | 239;266 | 2.28 | 0.217 | I3LIB5 | 643 | 3.09 | 0.581 | F1SMN5 | 2295 |  |  |
|  |  | F1S146 | 3848 | 2.01 | 0.005 | I3LIB5 | 818 | 6.88 | 0.203 | F1SN53 | 92 |  |  |
|  |  | F1S146 | 3732 | 2.14 | 0.004 | I3LIB5 | 684 | 2.50 | 0.102 | F1SNW4 | 49 |  |  |
|  |  | F1S156 | 598 | 3.86 | 0.005 | I3LIB5 | 928 | 5.43 | 0.001 | F1SQK1 | 187 |  |  |
|  |  | F1S156 | 597 | 3.89 | 0.004 | I3LIQ0 | 75 | 2.19 | 0.105 | F1SQK1 | 869 |  |  |
|  |  | F1S1T6 | 306 | 2.36 | 0.013 | I3LIV0 | 25;25 | 2.62 | 0.003 | F1SQK9 | 254 |  |  |
|  |  | F1S261 | 344 | 2.35 | 0.011 | I3LJE2 | 647 | 2.76 | 0.050 | F1SQV8 | 363 |  |  |
|  |  | F1S269 | 268 | 2.55 | 0.143 | I3LJJ6 | 94 | 2.61 | 0.033 | F1SU55 | 191 |  |  |
|  |  | F1S288 | 197 | 2.02 | 0.227 | I3LJX4 | 123 | 2.56 | 0.080 | F1SU55 | 200 |  |  |
|  |  | F1S2Q4 | 816 | 2.75 | 0.043 | I3LK59 | 40 | 2.88 | 0.085 | F1SU55 | 440 |  |  |
|  |  | F1S2V7 | 50 | 2.03 | 0.065 | I3LKP0 | 873 | 2.38 | 0.190 | F1SU55 | 201 |  |  |
|  |  | F1S2Y2 | 199 | 2.19 | 0.106 | I3LKP0 | 1250 | 2.17 | 0.068 | F1SUP1 | 42 |  |  |
|  |  | F1S2Y2 | 511 | 3.27 | 0.002 | I3LKP0 | 603 | 2.73 | 0.017 | F1SUT6 | 466 |  |  |
|  |  | F1S357 | 87 | 2.62 | 0.093 | I3LLD6 | 120 | 3.45 | 0.030 | F1SUV1 | 284 |  |  |
|  |  | F1S357 | 130 | 2.92 | 0.042 | I3LLG1 | 56 | 2.08 | 0.063 | F1SUZ2 | 771 |  |  |
|  |  | F1S3M9 | 521 | 18.40 | 0.191 | I3LLI0 | 5;5 | 2.12 | 0.118 | F1SV04 | 45 |  |  |
|  |  | F1S3R5 | 147 | 2.25 | 0.025 | I3LLI8 | 116 | 2.16 | 0.207 | F1SV46 | 72 |  |  |
|  |  | F1S3Y2 | 64 | 3.51 | 0.007 | I3LM30 | 624 | 2.66 | 0.099 | F1SV89 | 303;400 |  |  |
|  |  | F1S409 | 608 | 2.61 | 0.066 | I3LM34 | 973 | 2.78 | 0.154 | F2Z5B6 | 247;247 |  |  |
|  |  | F1S4G7 | 718 | 2.63 | 0.007 | I3LMC4 | 208 | 4.07 | 0.200 | F2Z5E2 | 64 |  |  |
|  |  | F1S4M3 | 975 | 2.86 | 0.013 | I3LMH4 | 1080 | 4.59 | 0.006 | F6PSL2 | 18 |  |  |
|  |  | F1S4M3 | 990 | 2.54 | 0.003 | I3LNE5 | 226 | 2.16 | 0.029 | I3L5C0 | 93 |  |  |
|  |  | F1S4N2 | 150 | 3.10 | 0.036 | I3LPY1 | 496 | 3.33 | 0.012 | I3L5D5 | 797 |  |  |
|  |  | F1S4P0 | 190 | 2.63 | 0.023 | I3LR51 | 36 | 4.29 | 0.141 | I3L5H6 | 311 |  |  |
|  |  | F1S4V5 | 471 | 5.94 | 0.000 | I3LRP0 | 300 | 2.74 | 0.267 | I3L606 | 308;331 |  |  |
|  |  | F1S4Z9 | 181 | 2.03 | 0.197 | I3LS02 | 94 | 2.55 | 0.433 | I3L606 | 309;332 |  |  |
|  |  | F1S554 | 515 | 8.85 | 0.042 | I3LS18 | 246;279 | 2.35 | 0.068 | I3L6D7 | 694;699 |  |  |
|  |  | F1S554 | 512 | 4.98 | 0.015 | I3LS66 | 246 | 2.11 | 0.146 | I3L7S4 | 113 |  |  |
|  |  | F1S5R6 | 824 | 2.33 | 0.096 | I3LSF0 | 461;461 | 5.38 | 0.008 | I3L7U7 | 24 |  |  |
|  |  | F1S5R6 | 5 | 3.61 | 0.007 | I3LSG7 | 284;474 | 2.77 | 0.004 | I3LAK5 | 586;641 |  |  |
|  |  | F1S5Y7 | 482 | 2.63 | 0.408 | I3LSI3 | 83 | 3.15 | 0.042 | I3LAY6 | 504;568 |  |  |
|  |  | F1S684 | 397 | 2.23 | 0.249 | I3LT10 | 32 | 2.30 | 0.082 | I3LAY6 | 303;367 |  |  |
|  |  | F1S684 | 512 | 2.83 | 0.009 | I3LTU3 | 55;53 | 2.76 | 0.068 | I3LCA5 | 1078;1278 |  |  |
|  |  | F1S732 | 126 | 2.30 | 0.197 | I3LU02 | 825;1143 | 2.21 | 0.224 | I3LCA5 | 1080;1280 |  |  |
|  |  | F1S786 | 300;377 | 2.02 | 0.418 | I3LU02 | 250;552;484 | 3.11 | 0.150 | I3LCE6 | 1468 |  |  |
|  |  | F1S836 | 424 | 2.31 | 0.038 | I3LU60 | 18 | 2.29 | 0.148 | I3LCW3 | 1154 |  |  |
|  |  | F1S880 | 140 | 2.37 | 0.097 | I3LUR1 | 162 | 2.25 | 0.049 | I3LCW3 | 1075 |  |  |
|  |  | F1S8C1 | 25 | 2.06 | 0.093 | I3LV20 | 27;195 | 2.92 | 0.013 | I3LDQ9 | 88 |  |  |
|  |  | F1S8C6 | 464 | 2.70 | 0.264 | I3LVD1 | 370;383 | 2.60 | 0.391 | I3LDQ9 | 89 |  |  |
|  |  | F1S8T1 | 707 | 2.04 | 0.043 | I3LVK2 | 134 | 3.14 | 0.000 | I3LDS5 | 542;519;352 |  |  |
|  |  | F1S994 | 17 | 2.26 | 0.134 | K7GKB2 | 213;253;298 | 2.76 | 0.021 | I3LDU9 | 231 |  |  |
|  |  | F1S995 | 521 | 2.64 | 0.054 | K7GKW0 | 184;102 | 2.66 | 0.225 | I3LEG2 | 427 |  |  |
|  |  | F1S9D1 | 477 | 2.65 | 0.070 | K7GKW0 | 108;26 | 6.29 | 0.023 | I3LEP9 | 319;333 |  |  |
|  |  | F1S9K5 | 995 | 2.31 | 0.086 | K7GKW0 | 39 | 3.03 | 0.006 | I3LEU9 | 209 |  |  |
|  |  | F1SA50 | 611 | 8.31 | 0.035 | K7GL02 | 167;168 | 2.30 | 0.170 | I3LEW5 | 19 |  |  |
|  |  | F1SA60 | 39 | 2.89 | 0.023 | K7GLA8 | 468;476 | 11.40 | 0.023 | I3LG94 | 742 |  |  |
|  |  | F1SA60 | 40 | 2.89 | 0.023 | K7GLE2 | 182;338 | 2.11 | 0.057 | I3LGU8 | 519;847 |  |  |
|  |  | F1SA66 | 134 | 18.93 | 0.001 | K7GLK3 | 90;152 | 2.20 | 0.033 | I3LKF3 | 38;38 |  |  |
|  |  | F1SAQ4 | 14 | 2.31 | 0.443 | K7GMG7 | 128;180;105 | 2.36 | 0.051 | I3LKP0 | 783 |  |  |
|  |  | F1SB54 | 47 | 2.52 | 0.109 | K7GMH0;P79293 | 703; 915 | 4.85 | 0.005 | I3LLF1 | 3 |  |  |
|  |  | F1SB57 | 45 | 2.11 | 0.375 | K7GMH0;P79293 | 415;1222;1221 | 8.54 | 0.000 | I3LLI0 | 200;202 |  |  |
|  |  | F1SBM5 | 177;177 | 2.12 | 0.533 | K7GMJ6 | 177;153;159 | 4.64 | 0.021 | I3LM30 | 498;498 |  |  |
|  |  | F1SBM5 | 175;175 | 2.86 | 0.088 | K7GMJ9 | 93;193;230;275 | 2.18 | 0.250 | I3LM30;K7GNL9 | 2639;2517;105;821 |  |  |
|  |  | F1SBQ5 | 213 | 2.91 | 0.406 | K7GMN8 | 200;230;231 | 2.08 | 0.047 | I3LNF8 | 932 |  |  |
|  |  | F1SBQ5 | 467 | 3.41 | 0.001 | K7GMN8 | 201;231;232 | 8.15 | 0.001 | I3LNF8 | 611 |  |  |
|  |  | F1SC49 | 263 | 3.81 | 0.024 | K7GNZ3 | 166 | 7.28 | 0.021 | I3LNW5 | 1022 |  |  |
|  |  | F1SC49 | 58 | 2.40 | 0.004 | K7GP58 | 126;130 | 5.48 | 0.009 | I3LPM5 | 12 |  |  |
|  |  | F1SC49 | 205 | 5.65 | 0.001 | K7GRY0 | 820 | 2.13 | 0.452 | I3LPY1 | 526 |  |  |
|  |  | F1SC51 | 91 | 2.12 | 0.198 | P00355 | 182 | 2.04 | 0.587 | I3LPY1 | 479 |  |  |
|  |  | F1SCM9 | 351 | 4.14 | 0.055 | P00503 | 138 | 4.86 | 0.139 | I3LPY5 | 260 |  |  |
|  |  | F1SCU6 | 140 | 2.73 | 0.067 | P00503 | 312 | 4.59 | 0.024 | I3LQ42 | 7 |  |  |
|  |  | F1SDL5;I3LG33 | 468;213 | 3.02 | 0.019 | P02189 | 68 | 4.21 | 0.031 | I3LQU0 | 310 |  |  |
|  |  | F1SDT0 | 97 | 4.61 | 0.127 | P02189 | 71 | 3.98 | 0.025 | I3LR88 | 192 |  |  |
|  |  | F1SDT0 | 146 | 6.83 | 0.029 | P02540 | 31 | 2.85 | 0.273 | I3LS02 | 98 |  |  |
|  |  | F1SDX6 | 68 | 2.63 | 0.072 | P02540 | 425 | 2.04 | 0.148 | I3LSM1 | 363;906 |  |  |
|  |  | F1SDX6 | 60 | 2.65 | 0.017 | P02540 | 32 | 2.67 | 0.144 | I3LSZ7 | 181 |  |  |
|  |  | F1SE25 | 66 | 2.29 | 0.219 | P02540 | 45 | 2.38 | 0.078 | I3LTH8 | 119;119;125 |  |  |
|  |  | F1SEJ4 | 1216 | 2.23 | 0.291 | P02540 | 28 | 4.69 | 0.025 | I3LTU3 | 54;52 |  |  |
|  |  | F1SEJ4 | 678 | 2.40 | 0.052 | P02540 | 23 | 5.13 | 0.022 | I3LVD1 | 331;344 |  |  |
|  |  | F1SEJ4 | 812 | 3.08 | 0.012 | P06199 | 232 | 6.48 | 0.083 | I3LVD1 | 263;276 |  |  |
|  |  | F1SEN8 | 267 | 2.89 | 0.087 | P08059 | 533 | 2.51 | 0.140 | K7GMV0 | 118 |  |  |
|  |  | F1SEN8 | 190 | 2.54 | 0.079 | P11607 | 22 | 2.20 | 0.279 | K7GND6 | 168 |  |  |
|  |  | F1SEN8 | 173 | 2.60 | 0.057 | P11607 | 580 | 2.90 | 0.054 | K7GNJ7 | 1367 |  |  |
|  |  | F1SEN8 | 217 | 6.82 | 0.006 | P29269 | 20;25 | 2.43 | 0.215 | K7GNY8 | 469;479 |  |  |
|  |  | F1SEN8 | 44 | 3.80 | 0.005 | P61013 | 16 | 2.20 | 0.436 | K7GPB6 | 130;752;761 |  |  |
|  |  | F1SEN8 | 121 | 3.78 | 0.004 | P61013 | 17 | 7.42 | 0.006 | K7GPB6 | 134;756;765 |  |  |
|  |  | F1SF57 | 248 | 2.05 | 0.153 | P62802 | 89 | 2.02 | 0.090 | K7GRG2 | 63 |  |  |
|  |  | F1SF88 | 84 | 2.22 | 0.001 | P62802 | 48 | 2.07 | 0.067 | P00355 | 331;107 |  |  |
|  |  | F1SFI5 | 265 | 2.98 | 0.240 | P67872 | 209;229 | 3.50 | 0.008 | P02189 | 118 |  |  |
|  |  | F1SFP6 | 344 | 2.05 | 0.515 | P79293 | 748; 103 | 3.90 | 0.003 | P02540 | 432 |  |  |
|  |  | F1SFS5 | 487 | 2.90 | 0.002 | P80387 | 73;108 | 3.42 | 0.013 | P11607 | 21 |  |  |
|  |  | F1SGD2 | 197 | 2.38 | 0.082 | Q19QU3 | 153 | 2.13 | 0.351 | P11708 | 22;22 |  |  |
|  |  | F1SGF0 | 709 | 2.99 | 0.003 | Q1KYT0 | 40 | 2.48 | 0.053 | P19133 | 59;156 |  |  |
|  |  | F1SH10 | 162 | 2.41 | 0.107 | Q1KYT0 | 176 | 3.05 | 0.005 | P21999 | 323 |  |  |
|  |  | F1SH10 | 145 | 2.88 | 0.039 | Q29026 | 77 | 2.09 | 0.016 | P26234 | 601 |  |  |
|  |  | F1SH92 | 225;223 | 3.55 | 0.392 | Q29101 | 255 | 3.22 | 0.036 | P36887 | 339 |  |  |
|  |  | F1SHL9 | 46 | 2.74 | 0.278 | Q3ZD69 | 390 | 2.33 | 0.436 | P63053 | 14 |  |  |
|  |  | F1SHW1 | 540 | 3.12 | 0.047 | Q5GN48 | 3602;589;416 | 2.66 | 0.035 | Q19QU3 | 120 |  |  |
|  |  | F1SIB9 | 239 | 2.99 | 0.025 | Q5GN48 | 3541;528;355 | 6.86 | 0.006 | Q29101 | 328 |  |  |
|  |  | F1SID7 | 402 | 2.03 | 0.074 | Q5GN48 | 3612;599;426 | 2.59 | 0.002 | Q3ZD69 | 277 |  |  |
|  |  | F1SID7 | 273 | 2.31 | 0.051 | Q5GN48 | 3489;476 | 4.27 | 0.000 | Q5GN48 | 1438 |  |  |
|  |  | F1SID7 | 670 | 2.91 | 0.019 | Q5S1U1 | 100 | 4.65 | 0.184 | Q6Q781 | 143;75;53 |  |  |
|  |  | F1SID7 | 1180 | 6.96 | 0.007 | Q5XLD3 | 128 | 2.10 | 0.169 | Q6Q781 | 509;441;419 |  |  |
|  |  | F1SID7 | 546 | 2.60 | 0.003 | Q5XLD3 | 174 | 2.01 | 0.149 | Q6Q781 | 311;243;221 |  |  |
|  |  | F1SIU8 | 13 | 3.80 | 0.015 | Q5XLD3 | 199 | 2.35 | 0.022 | Q6S4N2 | 636 |  |  |
|  |  | F1SIY9 | 223 | 3.16 | 0.007 | Q684M5 | 107 | 2.95 | 0.007 | Q7JFN2 | 320 |  |  |
|  |  | F1SIY9 | 224 | 3.16 | 0.007 | Q68J42 | 554 | 2.56 | 0.045 | Q95266 | 492 |  |  |
|  |  | F1SJC2 | 48 | 2.09 | 0.283 | Q68J42 | 647 | 5.00 | 0.012 |  |  |  |  |
|  |  | F1SJE4 | 118 | 2.37 | 0.300 | Q6Q781 | 312;244;222 | 2.85 | 0.012 |  |  |  |  |
|  |  | F1SJI2;I3LK21 | 171;204 | 3.07 | 0.386 | Q71LE2 | 81 | 2.88 | 0.017 |  |  |  |  |
|  |  | F1SJP2 | 377 | 2.01 | 0.052 | Q7JFN2;Q95266 | 146;17;76 | 2.87 | 0.121 |  |  |  |  |
|  |  | F1SJR2 | 1803 | 2.86 | 0.240 | Q7JFN2;Q95266 | 276 | 4.82 | 0.077 |  |  |  |  |
|  |  | F1SJR2 | 675 | 2.89 | 0.227 | Q7M2W6 | 21 | 2.72 | 0.285 |  |  |  |  |
|  |  | F1SJR2 | 533 | 2.11 | 0.165 | Q7M2W6 | 59 | 2.45 | 0.281 |  |  |  |  |
|  |  | F1SJR2 | 1174 | 2.56 | 0.058 | Q7M2W6 | 19 | 2.48 | 0.102 |  |  |  |  |
|  |  | F1SJR2 | 1625 | 2.53 | 0.016 | Q7M2W6 | 85 | 2.02 | 0.024 |  |  |  |  |
|  |  | F1SK12 | 1049 | 2.19 | 0.163 | Q7YS99 | 206 | 4.86 | 0.021 |  |  |  |  |
|  |  | F1SK12 | 1162 | 3.22 | 0.036 | Q95266 | 490 | 3.75 | 0.426 |  |  |  |  |
|  |  | F1SK12 | 1398 | 3.36 | 0.005 | Q9GLG4 | 306 | 2.01 | 0.025 |  |  |  |  |
|  |  | F1SK12 | 1324 | 4.41 | 0.000 | Q9GLG4 | 179 | 5.12 | 0.001 |  |  |  |  |
|  |  |  |  |  |  |  |  |  |  |  |  |  |  |
|  | sham | A5GFU6 | 337;352 | 2.01 | 0.294 |  |  |  |  | A0A0A0MY57 | 1321;1361 | F1SJS9 | 462 |
|  |  | B0KYV5 | 365;135 | 2.42 | 0.251 |  |  |  |  | A0A0A0MY57 | 1322;1362 | F1SKB1 | 361;361 |
|  |  | F1RGK5 | 246 | 7.60 | 0.155 |  |  |  |  | D2JYW4 | 184 | F1SKL6 | 62 |
|  |  | F1RGK5 | 247 | 3.71 | 0.119 |  |  |  |  | D4P3C2 | 281 | F1SKX3 | 42 |
|  |  | F1RH92 | 234 | 2.99 | 0.370 |  |  |  |  | F1RFJ8 | 508;549 | F1SLU6 | 624 |
|  |  | F1RHL9 | 433 | 2.82 | 0.481 |  |  |  |  | F1RFJ8 | 507;548 | F1SM75 | 1370;1174 |
|  |  | F1RIF8 | 127 | 2.57 | 0.125 |  |  |  |  | F1RG90 | 37 | F1SM86 | 444 |
|  |  | F1RN87 | 586 | 2.99 | 0.027 |  |  |  |  | F1RGP4 | 302 | F1SM86 | 488 |
|  |  | F1RR78 | 1530 | 2.13 | 0.135 |  |  |  |  | F1RH92 | 496 | F1SMG3 | 66 |
|  |  | F1RU38 | 328 | 2.09 | 0.009 |  |  |  |  | F1RHJ3 | 157 | F1SMQ5 | 321 |
|  |  | F1RVD1 | 37;33 | 2.26 | 0.163 |  |  |  |  | F1RIU0 | 128 | F1SN53 | 24 |
|  |  | F1RVG6 | 386 | 2.87 | 0.073 |  |  |  |  | F1RJ25 | 36 | F1SP54 | 123 |
|  |  | F1RVL5 | 1088 | 2.06 | 0.454 |  |  |  |  | F1RK48 | 473 | F1SPG9 | 959 |
|  |  | F1RWD9 | 745 | 2.11 | 0.150 |  |  |  |  | F1RKJ3 | 13;13 | F1SQI6 | 154 |
|  |  | F1RZ24 | 520 | 2.04 | 0.161 |  |  |  |  | F1RM42 | 436 | F1SQL3 | 306;191;200 |
|  |  | F1RZN9 | 84 | 2.23 | 0.312 |  |  |  |  | F1RMN3 | 525 | F1SQX7 | 574;573 |
|  |  | F1S441 | 584 | 11.52 | 0.086 |  |  |  |  | F1RMY0 | 92;137;139 | F1SSL6 | 250 |
|  |  | F1S4J0 | 120 | 2.03 | 0.175 |  |  |  |  | F1RNP6 | 125 | F1SSY1 | 99 |
|  |  | F1S4V5 | 405 | 2.99 | 0.228 |  |  |  |  | F1RNW3 | 142 | F1SUF2 | 172 |
|  |  | F1S5L7 | 94 | 2.42 | 0.007 |  |  |  |  | F1RNW3 | 143 | F1SUW3 | 73 |
|  |  | F1S684 | 531 | 3.64 | 0.246 |  |  |  |  | F1RNZ1 | 40 | F1SV22 | 3871 |
|  |  | F1S8C6 | 211 | 2.46 | 0.261 |  |  |  |  | F1RP42 | 81;158;168 | F1SV59 | 69 |
|  |  | F1S924 | 141 | 2.58 | 0.399 |  |  |  |  | F1RPS8 | 32;52 | F1SV59 | 72 |
|  |  | F1S995 | 488 | 2.19 | 0.195 |  |  |  |  | F1RQM2 | 500 | I3L5S3 | 346 |
|  |  | F1S9D1 | 489 | 2.10 | 0.229 |  |  |  |  | F1RSZ5 | 26 | I3L691 | 402 |
|  |  | F1SAQ3 | 344 | 2.08 | 0.147 |  |  |  |  | F1RT70 | 234 | I3L8K4 | 182 |
|  |  | F1SAX3 | 15;16 | 3.19 | 0.183 |  |  |  |  | F1RTB3 | 50 | I3L9T1 | 1612 |
|  |  | F1SC49 | 1212 | 2.27 | 0.213 |  |  |  |  | F1RTR6 | 485 | I3L9T1 | 2704 |
|  |  | F1SCV8 | 1008 | 2.60 | 0.263 |  |  |  |  | F1RVD4 | 804 | I3L9T1 | 2705 |
|  |  | F1SFF6 | 245 | 2.63 | 0.381 |  |  |  |  | F1RVL5 | 669 | I3LBD6 | 192 |
|  |  | F1SGJ3 | 486 | 2.68 | 0.212 |  |  |  |  | F1RVM1 | 15 | I3LCW3 | 1310 |
|  |  | F1SI09 | 24 | 2.84 | 0.034 |  |  |  |  | F1RWW4 | 41 | I3LCW3 | 1369 |
|  |  | F1SID7 | 282 | 6.01 | 0.056 |  |  |  |  | F1RX46 | 546 | I3LFI3 | 321 |
|  |  | F1SK12 | 561 | 2.15 | 0.021 |  |  |  |  | F1RYS9 | 207 | I3LFV4 | 209 |
|  |  | F1SKD6 | 450 | 3.67 | 0.240 |  |  |  |  | F1RZD0 | 211 | I3LHY2 | 211;214 |
|  |  | F1SKQ0 | 603 | 4.23 | 0.258 |  |  |  |  | F1RZW0 | 512;352 | I3LJ91 | 42 |
|  |  | F1SKQ0 | 605 | 4.31 | 0.249 |  |  |  |  | F1RZW0 | 269;109 | I3LKP0 | 1008 |
|  |  | F1SM75 | 82 | 2.10 | 0.072 |  |  |  |  | F1RZW0 | 431;271 | I3LKQ1 | 2 |
|  |  | F1SQ46 | 45 | 6.19 | 0.029 |  |  |  |  | F1RZW0 | 254;94 | I3LMB2 | 600 |
|  |  | F1SQP0 | 729 | 2.51 | 0.594 |  |  |  |  | F1S078 | 137 | I3LND2 | 158 |
|  |  | F1SS51 | 57 | 3.65 | 0.310 |  |  |  |  | F1S0D6 | 111 | I3LNK9 | 340;340 |
|  |  | F1ST03 | 1366 | 2.23 | 0.370 |  |  |  |  | F1S1V5 | 397 | I3LPF0 | 198;198 |
|  |  | F1STQ3 | 788 | 2.57 | 0.072 |  |  |  |  | F1S2J5 | 352 | I3LQ69 | 469 |
|  |  | I3L5D5 | 292 | 2.13 | 0.238 |  |  |  |  | F1S415 | 401 | I3LRZ4 | 585;608;625 |
|  |  | I3L7C4 | 712 | 2.07 | 0.049 |  |  |  |  | F1S485 | 784 | I3LSM7 | 595 |
|  |  | I3L9T1 | 2716;2716 | 2.22 | 0.411 |  |  |  |  | F1S4G7 | 231 | I3LSM7 | 296 |
|  |  | I3LB34 | 88;237 | 2.35 | 0.403 |  |  |  |  | F1S4P0 | 276 | I3LUE7 | 335 |
|  |  | I3LCW3 | 1124 | 2.47 | 0.132 |  |  |  |  | F1S4Z9 | 101 | I3LUE7 | 328 |
|  |  | I3LCW3 | 1153 | 2.20 | 0.125 |  |  |  |  | F1S5B0 | 637 | K7GL02 | 106;107 |
|  |  | I3LFI3 | 330 | 3.57 | 0.127 |  |  |  |  | F1S5R6 | 4 | K7GM47 | 78 |
|  |  | I3LQF9 | 602 | 2.30 | 0.118 |  |  |  |  | F1S7V6 | 33 | K7GPA3 | 20 |
|  |  | I3LSN9 | 64;458 | 2.56 | 0.414 |  |  |  |  | F1S7Y2 | 530 | K7GRG2 | 73 |
|  |  | I3LU02 | 1196;1521;1434;1499 | 2.02 | 0.013 |  |  |  |  | F1S814 | 509 | K7GSX0 | 6;6 |
|  |  | I3LU70 | 127;127 | 2.14 | 0.469 |  |  |  |  | F1S9W6 | 230 | P02540 | 60 |
|  |  | I3LUL5 | 42 | 3.50 | 0.084 |  |  |  |  | F1S9W6 | 256 | P02540 | 59 |
|  |  | K7GNW0 | 115 | 6.75 | 0.342 |  |  |  |  | F1SBF0 | 840 | P02543 | 419 |
|  |  | O02705 | 454 | 2.79 | 0.317 |  |  |  |  | F1SBU9 | 510 | P02543 | 420 |
|  |  | O18734 | 128 | 3.22 | 0.310 |  |  |  |  | F1SBY5 | 1274 | P11708 | 242;179;74 |
|  |  | P02540 | 454 | 2.65 | 0.540 |  |  |  |  | F1SDA4 | 643 | P11708 | 189;126;21;65 |
|  |  | P09623 | 297 | 2.37 | 0.290 |  |  |  |  | F1SE69 | 101 | P29269 | 19;24 |
|  |  | P11607 | 649 | 2.09 | 0.326 |  |  |  |  | F1SEV8 | 448;448;86 | P79293 | 350;1157;1156 |
|  |  | P19620 | 24 | 2.26 | 0.388 |  |  |  |  | F1SFI4 | 331 | P79293 | 351;1158;1157 |
|  |  | P29700 | 135 | 2.34 | 0.108 |  |  |  |  | F1SFW7 | 614;615 | P80310 | 25;25 |
|  |  | Q2EN76 | 94 | 2.34 | 0.421 |  |  |  |  | F1SGD2 | 250 | Q06A98 | 25 |
|  |  | Q6Q781 | 36 | 2.14 | 0.386 |  |  |  |  | F1SI09 | 12 | Q0MUU2 | 47 |
|  |  | Q7YS99 | 198 | 2.15 | 0.179 |  |  |  |  | F1SID1 | 429 | Q2EN76 | 44 |
|  |  | Q95266 | 334 | 2.73 | 0.046 |  |  |  |  | F1SJP2 | 184 | Q3ZD69 | 301 |
|  |  | Q95266 | 1919 | 2.21 | 0.135 |  |  |  |  | F1SJR2 | 1440 | Q9MYT8 | 68 |
|  |  |  |  |  |  |  |  |  |  |  |  |  |  |
| RIPC | baseline | A5GFU6 | 337;352 | 3.01 | 0.115 | F1SMN1 | 47 | 2.21 | 0.306 | A0A0A0MY57 | 1321;1361 | F1SSY1 | 194 |
|  |  | B0KYV5 | 365;135 | 2.80 | 0.219 | F1SMN5 | 1888 | 2.11 | 0.239 | A0A0A0MY57 | 1322;1362 | F1ST98 | 95 |
|  |  | B2ZPK1 | 284 | 2.29 | 0.455 | F1SNM7 | 95 | 4.22 | 0.060 | D0G7F6 | 106;106 | I3L548 | 676;685 |
|  |  | D2SQP3 | 95 | 2.30 | 0.224 | F1SQS2 | 160;67 | 2.33 | 0.444 | F1RGH4 | 578 | I3L5N6 | 16 |
|  |  | F1RFF0 | 633 | 2.89 | 0.173 | F1SS51 | 57 | 2.31 | 0.451 | F1RGI7 | 195 | I3L5S3 | 346 |
|  |  | F1RFV9 | 769 | 2.33 | 0.035 | F1SS77 | 101;109 | 2.24 | 0.508 | F1RGU5 | 1460 | I3L691 | 402 |
|  |  | F1RGH4 | 556 | 3.98 | 0.230 | F1ST95 | 37;37;37 | 2.31 | 0.007 | F1RIN7 | 220 | I3L8Q0 | 292 |
|  |  | F1RGK5 | 246 | 2.34 | 0.679 | F1STJ9 | 2 | 2.27 | 0.065 | F1RJ25 | 36 | I3L9T1 | 589;589 |
|  |  | F1RGN8 | 126 | 2.79 | 0.039 | F1SU55 | 892 | 2.22 | 0.092 | F1RK61 | 299 | I3LA95 | 887;1219;1221 |
|  |  | F1RII7 | 71 | 3.47 | 0.011 | F1SU55 | 893 | 3.92 | 0.001 | F1RKJ9 | 159 | I3LA95 | 943;1275;1277 |
|  |  | F1RIP1 | 710 | 3.00 | 0.015 | F1SUN0 | 405 | 2.56 | 0.271 | F1RKW6 | 18 | I3LAR5 | 141 |
|  |  | F1RIU9 | 45 | 2.48 | 0.054 | F1SVB4 | 82;82 | 2.26 | 0.176 | F1RL91 | 15 | I3LB67 | 65;421 |
|  |  | F1RKH4 | 24 | 4.37 | 0.054 | F2Z517 | 77 | 2.66 | 0.430 | F1RN27 | 53 | I3LBS8 | 810 |
|  |  | F1RPD7 | 726 | 3.48 | 0.253 | F2Z5P0 | 17 | 2.18 | 0.396 | F1RNZ1 | 40 | I3LFI3 | 316 |
|  |  | F1RQM9 | 4609 | 2.31 | 0.248 | I3L594 | 174;194 | 2.66 | 0.028 | F1RNZ1 | 38 | I3LFX8 | 155;187;187 |
|  |  | F1RQM9 | 4610 | 2.31 | 0.248 | I3L5H6 | 311 | 2.29 | 0.380 | F1RP42 | 81;158;168 | I3LI05 | 4 |
|  |  | F1RRS8 | 693 | 2.25 | 0.520 | I3L6D7 | 715;720 | 5.81 | 0.062 | F1RQC5 | 627 | I3LJC8 | 95 |
|  |  | F1RS45 | 1513 | 2.16 | 0.273 | I3L6D7 | 694;699 | 2.65 | 0.036 | F1RRD7 | 812 | I3LJE2 | 27 |
|  |  | F1RVG6 | 386 | 2.26 | 0.160 | I3L8Q0 | 1366 | 4.06 | 0.051 | F1RTT0 | 152 | I3LJE2 | 645 |
|  |  | F1RWF5 | 86;103 | 2.56 | 0.147 | I3LA95 | 790;1122;1124 | 2.20 | 0.358 | F1RU31 | 490 | I3LND2 | 158 |
|  |  | F1RZN9 | 84;84 | 4.77 | 0.196 | I3LAQ3 | 33 | 2.72 | 0.153 | F1RU31 | 492 | I3LNK9 | 340;340 |
|  |  | F1S146 | 1700 | 2.31 | 0.019 | I3LAY6 | 300;364 | 5.31 | 0.003 | F1RUD6 | 40 | I3LNV3 | 83 |
|  |  | F1S1L1 | 94 | 2.19 | 0.552 | I3LCE6 | 1468 | 3.12 | 0.109 | F1RW49 | 145 | I3LSM7 | 296 |
|  |  | F1S1T6 | 305 | 2.15 | 0.035 | I3LDR9 | 302;147 | 4.29 | 0.034 | F1RW75 | 1803 | K7GL02 | 106;107 |
|  |  | F1S2M0 | 179;182 | 5.40 | 0.011 | I3LDS5 | 774;745;584 | 4.76 | 0.049 | F1RWD9 | 301;260 | K7GLA8 | 939;929 |
|  |  | F1S2V7 | 42 | 2.85 | 0.097 | I3LDU9 | 95 | 2.08 | 0.032 | F1S156 | 260 | K7GNW1 | 121;292 |
|  |  | F1S441 | 584 | 4.51 | 0.329 | I3LEP9 | 330;344 | 2.16 | 0.092 | F1S1H3 | 492 | P05383 | 264 |
|  |  | F1S4Z9 | 201 | 3.05 | 0.305 | I3LFZ9 | 938 | 3.25 | 0.075 | F1S415 | 401 | P08059 | 532 |
|  |  | F1S4Z9 | 189 | 14.18 | 0.010 | I3LGJ5 | 978 | 2.55 | 0.127 | F1S4G7 | 231 | P08059 | 214 |
|  |  | F1S7K3 | 281 | 2.13 | 0.337 | I3LJB8 | 425 | 2.54 | 0.052 | F1S4R6 | 537 | P79293 | 712;1519;1518 |
|  |  | F1S8D2 | 122 | 3.05 | 0.025 | I3LKJ3 | 79 | 2.51 | 0.026 | F1S5R6 | 4 | Q3ZD69 | 632 |
|  |  | F1S9Q1 | 326;328 | 2.03 | 0.078 | I3LL97 | 192 | 2.02 | 0.002 | F1S5Z3 | 508 | Q3ZD69 | 19 |
|  |  | F1SAM8 | 1636 | 2.66 | 0.035 | I3LLF1 | 3 | 2.81 | 0.358 | F1S620 | 657 | Q95266 | 331 |
|  |  | F1SBY5 | 391 | 2.04 | 0.183 | I3LR88 | 192 | 3.63 | 0.097 | F1S7K5 | 237 | Q95266 | 336 |
|  |  | F1SCI4 | 185 | 3.23 | 0.033 | I3LS02 | 42 | 5.14 | 0.009 | F1S7T1 | 871 | Q9MZ15 | 252 |
|  |  | F1SCU6 | 186 | 2.19 | 0.210 | I3LSI3 | 83 | 2.21 | 0.045 | F1S814 | 509 |  |  |
|  |  | F1SDF8 | 81 | 5.95 | 0.023 | I3LSM7 | 88 | 7.61 | 0.017 | F1S8C6 | 202 |  |  |
|  |  | F1SDL5 | 394 | 2.28 | 0.379 | I3LU02 | 1196;1521 | 4.63 | 0.023 | F1S8M9 | 308 |  |  |
|  |  | F1SDW4 | 134 | 5.28 | 0.100 | I3LU70 | 127;127 | 4.15 | 0.256 | F1SC49 | 55 |  |  |
|  |  | F1SE30 | 888 | 3.80 | 0.027 | I3LUL5 | 42 | 3.57 | 0.084 | F1SE69 | 101 |  |  |
|  |  | F1SEV8 | 495 | 2.15 | 0.122 | I3LVD1 | 331;344 | 2.77 | 0.018 | F1SEN8 | 41 |  |  |
|  |  | F1SF88 | 542 | 2.25 | 0.148 | K7GLE2 | 182;338 | 2.28 | 0.193 | F1SJR2 | 956 |  |  |
|  |  | F1SFF6 | 245 | 7.06 | 0.179 | K7GMJ6 | 134;110 | 3.35 | 0.034 | F1SLS8 | 420 |  |  |
|  |  | F1SFQ4 | 189 | 3.58 | 0.055 | K7GND6 | 786 | 2.22 | 0.345 | F1SLY4 | 393 |  |  |
|  |  | F1SGF0 | 767 | 2.21 | 0.031 | O18734 | 26 | 6.89 | 0.016 | F1SLY4 | 186 |  |  |
|  |  | F1SHL0 | 493 | 5.61 | 0.018 | P00889 | 226 | 2.05 | 0.329 | F1SMQ5 | 321 |  |  |
|  |  | F1SIB9 | 431;428 | 6.18 | 0.116 | P61013 | 16 | 5.84 | 0.080 | F1SN53 | 24 |  |  |
|  |  | F1SID7 | 282 | 9.48 | 0.047 | P79293 | 960;959 | 6.47 | 0.061 | F1SNY5 | 58 |  |  |
|  |  | F1SJR4 | 241 | 2.71 | 0.266 | Q2EN76 | 94;94 | 2.30 | 0.451 | F1SQX7 | 574;573 |  |  |
|  |  | F1SKQ0 | 603 | 5.29 | 0.206 | Q6Q781 | 143;75;53 | 3.40 | 0.213 | F1SS24 | 286 |  |  |
|  |  | F1SKQ0 | 605 | 5.39 | 0.198 | Q6Q781 | 36 | 4.35 | 0.136 | F1SSB0 | 136 |  |  |
|  |  | F1SKZ7 | 70;89 | 2.18 | 0.411 | Q6Q781 | 1919 | 2.21 | 0.237 | F1SSB0 | 138 |  |  |
|  |  | F1SLF0 | 219 | 2.01 | 0.427 | Q6Q781 | 962 | 6.47 | 0.061 | F1SSY1 | 99 |  |  |
|  |  | F1SMA1 | 463 | 2.93 | 0.034 |  |  |  |  | F1SSY1 | 193 |  |  |
|  |  |  |  |  |  |  |  |  |  |  |  |  |  |
|  | early reperfusion | A5GFS8 | 158 | 4.26 | 0.008 | F1SP32 | 160 | 13.77 | 0.000 | A5A769 | 64 | F1SQK1 | 869 |
|  |  | B2ZPK1 | 259 | 2.39 | 0.399 | F1SP82 | 34 | 18.75 | 0.368 | A5D9P0 | 140 | F1SQP0 | 729 |
|  |  | C1PIG4 | 96;41 | 4.15 | 0.020 | F1SPR6 | 711;706 | 3.46 | 0.368 | A5GFS8 | 159 | F1SS33 | 392;395 |
|  |  | D2JYW4 | 263 | 4.61 | 0.002 | F1SQ16 | 1263 | 6.53 | 0.009 | A9LM01 | 104;46 | F1SS99 | 368 |
|  |  | F1REW5 | 129 | 3.31 | 0.279 | F1SQ46 | 310;378 | 2.43 | 0.033 | A9LM01 | 90 | F1SSH3 | 63 |
|  |  | F1RFR1 | 625 | 2.85 | 0.020 | F1SQK1 | 870 | 18.96 | 0.014 | F1REW5 | 1000 | F1SSP0 | 15 |
|  |  | F1RG04 | 248 | 3.22 | 0.012 | F1SQK1 | 367 | 33.53 | 0.008 | F1RFB2 | 1155 | F1ST03 | 1366 |
|  |  | F1RG35 | 23 | 2.05 | 0.432 | F1SS29 | 45 | 2.41 | 0.054 | F1RFY6 | 1092 | F1ST98 | 93 |
|  |  | F1RG85 | 207 | 2.91 | 0.054 | F1STQ3 | 703;703 | 4.64 | 0.053 | F1RFY6 | 676 | F1STQ3 | 788;788 |
|  |  | F1RGD9 | 28 | 3.80 | 0.180 | F1SU06 | 51 | 2.05 | 0.607 | F1RG15 | 366 | F1SU33 | 268 |
|  |  | F1RGD9 | 27 | 7.80 | 0.015 | F1SU55 | 744 | 4.84 | 0.084 | F1RGE7 | 282 | F1SU55 | 191 |
|  |  | F1RGH4 | 776 | 5.06 | 0.230 | F1SU55 | 939 | 3.80 | 0.018 | F1RGE7 | 256 | F1SU55 | 440 |
|  |  | F1RGI7 | 188 | 2.20 | 0.073 | F1SU55 | 932 | 5.73 | 0.005 | F1RGH4 | 581 | F1SU55 | 200 |
|  |  | F1RGP1 | 14 | 2.88 | 0.152 | F1SU55 | 722 | 3.14 | 0.001 | F1RGH4 | 497 | F1SU55 | 201 |
|  |  | F1RHL9 | 594 | 2.56 | 0.330 | F1SUJ6 | 101 | 2.34 | 0.105 | F1RGJ2 | 690 | F1SU85 | 457 |
|  |  | F1RHL9 | 624 | 2.03 | 0.144 | F1SUR3 | 151 | 2.84 | 0.311 | F1RGN8 | 333 | F1SUH2 | 269 |
|  |  | F1RHL9 | 435 | 2.25 | 0.051 | F1SUT6 | 466 | 2.37 | 0.059 | F1RH92 | 145 | F1SUX4 | 1995 |
|  |  | F1RHM3 | 1940 | 2.13 | 0.075 | F1SV04 | 45 | 5.36 | 0.063 | F1RH92 | 230 | F1SUZ2 | 552 |
|  |  | F1RHM3 | 3933 | 2.68 | 0.073 | F1SV22 | 2121 | 2.99 | 0.292 | F1RHC6 | 55 | F1SUZ4 | 257 |
|  |  | F1RIC1 | 18 | 2.26 | 0.342 | F1SV46 | 115;78;79 | 2.20 | 0.297 | F1RHG1 | 1253 | F1SV11 | 1023 |
|  |  | F1RIN7 | 371 | 2.23 | 0.092 | F2Z4X6 | 139 | 3.27 | 0.369 | F1RHG1 | 1252 | F1SV22 | 5536 |
|  |  | F1RIP1 | 727 | 16.66 | 0.001 | F2Z4Z1 | 71 | 4.06 | 0.110 | F1RHL9 | 179 | F1SV46 | 72 |
|  |  | F1RIW3 | 223 | 6.22 | 0.011 | F2Z5C7 | 263 | 2.23 | 0.112 | F1RHM3 | 1937 | F1SVD5 | 101 |
|  |  | F1RJ93 | 172 | 4.72 | 0.003 | F2Z5G3 | 102 | 6.21 | 0.009 | F1RHM3 | 2908 | F2Z4X6 | 136 |
|  |  | F1RJI2 | 133 | 2.45 | 0.220 | F2Z5Q6 | 234;231 | 3.35 | 0.087 | F1RHM8 | 60 | F2Z5B6 | 247 |
|  |  | F1RK48 | 785 | 2.16 | 0.174 | F2Z5Q6 | 238;235 | 4.39 | 0.009 | F1RHN2 | 604 | F2Z5B6 | 162 |
|  |  | F1RK48 | 63 | 3.40 | 0.076 | F2Z5W6 | 27 | 2.76 | 0.016 | F1RHU2 | 88 | F2Z5H8 | 177 |
|  |  | F1RK58 | 2 | 2.06 | 0.079 | F6PSL2 | 117 | 2.62 | 0.075 | F1RHU2 | 92 | G8ENL4 | 331;92 |
|  |  | F1RKG8 | 185 | 25.85 | 0.001 | I3L548 | 618;627 | 4.72 | 0.017 | F1RHU2 | 212 | I3L548 | 650;659 |
|  |  | F1RKJ3 | 32 | 3.79 | 0.127 | I3L5N6 | 14 | 4.29 | 0.041 | F1RHU2 | 211 | I3L548 | 651;660 |
|  |  | F1RKJ3 | 11;11 | 2.50 | 0.046 | I3L7U1 | 418 | 10.43 | 0.007 | F1RI15 | 76 | I3L548 | 635;644 |
|  |  | F1RKX9 | 185 | 3.41 | 0.231 | I3L816 | 103;104 | 2.23 | 0.052 | F1RIC1 | 65 | I3L5C0 | 93 |
|  |  | F1RLL1 | 148 | 2.05 | 0.446 | I3L8D7 | 598 | 4.68 | 0.062 | F1RIC1 | 63 | I3L5D4 | 23 |
|  |  | F1RLL1 | 593;420 | 3.83 | 0.007 | I3L8K4 | 164 | 2.47 | 0.432 | F1RIN7 | 372 | I3L5D5 | 611 |
|  |  | F1RLP5 | 619 | 9.08 | 0.003 | I3L8K4 | 190 | 2.16 | 0.372 | F1RIP1 | 731 | I3L5D5 | 292 |
|  |  | F1RM62 | 59;15 | 4.03 | 0.035 | I3L8K4 | 107 | 2.13 | 0.322 | F1RIP1 | 730 | I3L5T3 | 155 |
|  |  | F1RM62 | 157 | 3.68 | 0.026 | I3L8Q0 | 259 | 2.98 | 0.439 | F1RJN0 | 217 | I3L6C1 | 898 |
|  |  | F1RMC1 | 372 | 6.85 | 0.015 | I3L8Q0 | 274 | 4.40 | 0.295 | F1RK02 | 6 | I3L6H7 | 220 |
|  |  | F1RN89 | 658 | 2.15 | 0.151 | I3L8Q0 | 1388 | 2.25 | 0.267 | F1RK61 | 209 | I3L788 | 237 |
|  |  | F1RN89 | 635 | 4.27 | 0.019 | I3L8Q0 | 276 | 3.10 | 0.249 | F1RKC0 | 281 | I3L7S4 | 113 |
|  |  | F1RNP6 | 100 | 2.42 | 0.338 | I3L8Q0 | 294 | 2.17 | 0.241 | F1RKY1 | 37 | I3L7U7 | 24 |
|  |  | F1RP31 | 225 | 5.92 | 0.156 | I3L8Q0 | 1738 | 7.55 | 0.014 | F1RLE5 | 6 | I3L801 | 125 |
|  |  | F1RR47 | 276 | 3.69 | 0.115 | I3L8Q0 | 354 | 13.17 | 0.014 | F1RLL1 | 602;429;565 | I3L806 | 596 |
|  |  | F1RRJ1 | 545 | 2.01 | 0.138 | I3L8Q0 | 355 | 5.77 | 0.011 | F1RLL1 | 603;430;566 | I3L806 | 801 |
|  |  | F1RRN1 | 457 | 5.89 | 0.042 | I3L8Q0 | 1343 | 10.69 | 0.001 | F1RLT0 | 841 | I3L8Q0 | 829 |
|  |  | F1RRS8 | 536 | 2.49 | 0.390 | I3L9F5 | 435 | 2.40 | 0.133 | F1RMZ7 | 282 | I3L8Q0 | 800 |
|  |  | F1RRV6 | 2 | 2.48 | 0.008 | I3L9I6 | 594 | 4.37 | 0.364 | F1RN75 | 191;357;395 | I3L8Q0 | 1965 |
|  |  | F1RSY4 | 403 | 2.11 | 0.571 | I3L9K5 | 19 | 3.05 | 0.386 | F1RNP6 | 504 | I3L9T1 | 2716 |
|  |  | F1RUJ0 | 202 | 14.16 | 0.002 | I3L9K5 | 223 | 2.21 | 0.315 | F1RNW3 | 154 | I3L9T1 | 2562 |
|  |  | F1RVG6 | 886;158 | 2.18 | 0.342 | I3L9R9 | 594 | 2.55 | 0.010 | F1RPS8 | 516;536 | I3L9T1 | 2227 |
|  |  | F1RVG6 | 387 | 2.24 | 0.205 | I3L9T1 | 1544 | 2.31 | 0.416 | F1RPY3 | 30 | I3L9T1 | 1779 |
|  |  | F1RVG6 | 950 | 7.05 | 0.006 | I3L9T1 | 1639 | 2.13 | 0.369 | F1RQ09 | 108 | I3L9T1 | 2503 |
|  |  | F1RVL5 | 1088 | 2.95 | 0.507 | I3L9T1 | 1351 | 2.04 | 0.148 | F1RQ28 | 591 | I3L9T1 | 2112 |
|  |  | F1RVL5 | 364 | 2.17 | 0.023 | I3L9T1 | 2115 | 2.19 | 0.144 | F1RQC5 | 378 | I3L9T1 | 1425 |
|  |  | F1RVZ0 | 596;604 | 3.53 | 0.041 | I3L9T1 | 3246;3251 | 3.24 | 0.137 | F1RQQ7 | 85 | I3L9T1 | 118 |
|  |  | F1RW75 | 1511 | 2.30 | 0.041 | I3L9T1 | 1899 | 4.20 | 0.137 | F1RQQ8 | 473 | I3L9T1 | 265 |
|  |  | F1RWC5 | 930 | 20.83 | 0.000 | I3L9T1 | 1901 | 4.20 | 0.137 | F1RR22 | 1038;1043 | I3L9T1 | 2208 |
|  |  | F1RWD9 | 1372;1242 | 2.31 | 0.352 | I3L9T1 | 1811 | 3.36 | 0.089 | F1RR78 | 1595 | I3L9T1 | 2453 |
|  |  | F1RWD9 | 228;214 | 2.84 | 0.256 | I3L9T1 | 2040 | 4.11 | 0.052 | F1RRE0 | 162 | I3L9T1 | 1424 |
|  |  | F1RWD9 | 1091;961 | 2.57 | 0.247 | I3L9T1 | 2451 | 8.26 | 0.032 | F1RRM9 | 228 | I3L9T1 | 3016;3021 |
|  |  | F1RWD9 | 1316;1186 | 4.70 | 0.233 | I3L9T1 | 3250;3255 | 5.02 | 0.029 | F1RRN1 | 609 | I3L9T1 | 1561 |
|  |  | F1RWD9 | 890;760 | 2.33 | 0.127 | I3L9T1 | 3035;3040 | 2.07 | 0.020 | F1RRS8 | 741 | I3L9T1 | 267 |
|  |  | F1RWD9 | 1197;1067 | 3.27 | 0.073 | I3L9T1 | 3208;3213 | 30.94 | 0.017 | F1RS42 | 840 | I3LAK5 | 586;641 |
|  |  | F1RWF5 | 234;251 | 2.38 | 0.212 | I3L9T1 | 3108;3113 | 2.82 | 0.009 | F1RS45 | 1515 | I3LAR5 | 415 |
|  |  | F1RWW4 | 124 | 7.80 | 0.046 | I3LA95 | 1199;1531 | 3.06 | 0.282 | F1RS65 | 543;570 | I3LAR5 | 63 |
|  |  | F1RWW4 | 123 | 8.46 | 0.030 | I3LAK5 | 588;643 | 4.79 | 0.217 | F1RSD1 | 60 | I3LAR5 | 360 |
|  |  | F1RWW4 | 326 | 8.67 | 0.004 | I3LAR5 | 421 | 10.67 | 0.248 | F1RSG8 | 81 | I3LAR5 | 351 |
|  |  | F1RX74 | 29 | 17.34 | 0.041 | I3LAR5 | 318 | 3.09 | 0.246 | F1RSR8 | 344 | I3LAY6 | 303;367 |
|  |  | F1RXG7 | 10 | 2.67 | 0.142 | I3LAR5 | 181 | 2.38 | 0.124 | F1RT70 | 235 | I3LAY6 | 504;568 |
|  |  | F1RXG7 | 13 | 3.31 | 0.142 | I3LAR5 | 243 | 5.62 | 0.041 | F1RTB6 | 115 | I3LB34 | 88;237 |
|  |  | F1RXZ2 | 71;96 | 4.26 | 0.095 | I3LAR5 | 319 | 18.40 | 0.020 | F1RTF0 | 1220 | I3LBD6 | 21 |
|  |  | F1RYA1 | 182 | 6.25 | 0.038 | I3LAR5 | 127 | 3.79 | 0.016 | F1RU38 | 327 | I3LC03 | 118 |
|  |  | F1RYQ4 | 300 | 6.02 | 0.016 | I3LAR5 | 149 | 3.11 | 0.008 | F1RU38 | 328 | I3LCU9 | 63 |
|  |  | F1RYS9 | 173 | 6.96 | 0.079 | I3LAR5 | 422 | 7.38 | 0.001 | F1RU40 | 596 | I3LCW3 | 873 |
|  |  | F1RZ13 | 682 | 2.08 | 0.209 | I3LAR5 | 31 | 16.31 | 0.000 | F1RUJ3 | 768 | I3LCW3 | 2380 |
|  |  | F1RZC6 | 1288 | 2.13 | 0.194 | I3LAR5 | 297 | 10.84 | 0.000 | F1RUR3 | 29 | I3LCW3 | 1154 |
|  |  | F1RZH1 | 39 | 6.35 | 0.033 | I3LAY6 | 95;159 | 2.36 | 0.313 | F1RUX3 | 633 | I3LDS5 | 542;519;352 |
|  |  | F1RZN9 | 73;73 | 2.91 | 0.002 | I3LAY6 | 248;312 | 13.93 | 0.005 | F1RVG6 | 797;67 | I3LDS5 | 1342;1305 |
|  |  | F1RZQ6 | 195;159 | 2.56 | 0.222 | I3LCL0 | 371 | 2.13 | 0.150 | F1RVG6 | 882;154 | I3LDS5 | 273;278 |
|  |  | F1RZQ6 | 42;42;42 | 9.06 | 0.000 | I3LCW3 | 1211 | 2.00 | 0.530 | F1RVG6 | 253 | I3LEG2 | 427 |
|  |  | F1RZU8 | 185 | 4.20 | 0.112 | I3LCW3 | 2563 | 2.91 | 0.186 | F1RVG6 | 251 | I3LEP9 | 319;333 |
|  |  | F1RZW0 | 228;68 | 2.68 | 0.455 | I3LCW3 | 1153 | 3.01 | 0.061 | F1RVL5 | 89 | I3LEU9 | 209 |
|  |  | F1RZW0 | 523;363 | 2.11 | 0.128 | I3LDR9 | 388;232 | 4.82 | 0.375 | F1RVZ3 | 295 | I3LFQ5 | 103 |
|  |  | F1RZW0 | 739 | 4.78 | 0.041 | I3LDU9 | 105 | 12.98 | 0.186 | F1RW75 | 1031 | I3LFQ5 | 778 |
|  |  | F1RZW0 | 259;99 | 2.08 | 0.020 | I3LDU9 | 222 | 2.44 | 0.151 | F1RW75 | 2613 | I3LG94 | 1117 |
|  |  | F1RZW0 | 328;168 | 4.27 | 0.004 | I3LDU9 | 129 | 3.68 | 0.009 | F1RW75 | 2404 | I3LGU7 | 182 |
|  |  | F1RZW0 | 432;272 | 25.91 | 0.001 | I3LE55 | 285 | 2.13 | 0.266 | F1RWD9 | 741 | I3LGU7 | 186 |
|  |  | F1RZW0 | 443;283 | 31.94 | 0.000 | I3LFI3 | 318 | 3.72 | 0.249 | F1RWD9 | 520;479 | I3LGU8 | 519;847 |
|  |  | F1S078 | 141 | 3.02 | 0.496 | I3LFI3 | 330 | 3.04 | 0.203 | F1RWE1 | 1163;1168 | I3LIB5 | 818 |
|  |  | F1S087 | 95 | 2.40 | 0.257 | I3LFI3 | 20 | 6.09 | 0.033 | F1RWF4 | 208 | I3LIC8 | 331 |
|  |  | F1S0L8 | 298 | 3.25 | 0.006 | I3LG33 | 468;213 | 2.18 | 0.085 | F1RWK0 | 461 | I3LKL3 | 364 |
|  |  | F1S146 | 3732 | 9.57 | 0.002 | I3LG67 | 117 | 3.25 | 0.064 | F1RWW4 | 171 | I3LKP0 | 406 |
|  |  | F1S146 | 3848 | 7.27 | 0.000 | I3LH91 | 228 | 3.53 | 0.498 | F1RWW4 | 111 | I3LKP0 | 738 |
|  |  | F1S156 | 598 | 2.00 | 0.044 | I3LH91 | 3 | 4.69 | 0.046 | F1RWW4 | 225 | I3LKP0 | 556 |
|  |  | F1S156 | 597 | 2.57 | 0.026 | I3LI59 | 45 | 8.92 | 0.001 | F1RWW4 | 183 | I3LKP0 | 783 |
|  |  | F1S156 | 591 | 2.48 | 0.009 | I3LIB5 | 644 | 2.33 | 0.521 | F1RX74 | 189 | I3LKP0 | 451 |
|  |  | F1S156 | 250 | 26.12 | 0.000 | I3LIB5 | 643 | 3.10 | 0.413 | F1RXZ6 | 273 | I3LKP0 | 603 |
|  |  | F1S1U5 | 451 | 5.71 | 0.007 | I3LIB5 | 684 | 3.32 | 0.093 | F1RYQ4 | 299 | I3LLD6 | 120 |
|  |  | F1S261 | 344 | 2.21 | 0.201 | I3LIV0 | 25;25 | 15.61 | 0.000 | F1RZ24 | 22 | I3LLX3 | 467 |
|  |  | F1S261 | 244 | 8.25 | 0.014 | I3LJX4 | 123 | 4.00 | 0.041 | F1RZK8 | 456 | I3LM30 | 498 |
|  |  | F1S269 | 159 | 2.53 | 0.192 | I3LKE2 | 223 | 15.66 | 0.000 | F1RZU8 | 2603 | I3LM30 | 2639;2517;105;821 |
|  |  | F1S409 | 608 | 2.20 | 0.019 | I3LKP0 | 873 | 8.85 | 0.034 | F1S086 | 398;408 | I3LMH4 | 1080 |
|  |  | F1S415 | 379 | 4.73 | 0.010 | I3LKP0 | 722 | 16.09 | 0.032 | F1S087 | 81 | I3LMM6 | 152 |
|  |  | F1S415 | 387 | 3.92 | 0.002 | I3LKP0 | 580 | 12.99 | 0.031 | F1S0D8 | 421 | I3LNW5 | 1022 |
|  |  | F1S415 | 388 | 3.92 | 0.002 | I3LKP0 | 600 | 2.30 | 0.028 | F1S156 | 229 | I3LPM5 | 12 |
|  |  | F1S445 | 272 | 3.47 | 0.095 | I3LKP0 | 1250 | 14.29 | 0.001 | F1S156 | 190 | I3LQ42 | 7 |
|  |  | F1S448 | 792 | 5.12 | 0.067 | I3LKP0 | 1020 | 10.38 | 0.000 | F1S269 | 196 | I3LQP1 | 172;170 |
|  |  | F1S485 | 667 | 6.36 | 0.011 | I3LLI0 | 5;5 | 7.76 | 0.199 | F1S2N4 | 534 | I3LQP1 | 175;173 |
|  |  | F1S4G7 | 718 | 3.39 | 0.001 | I3LLI8 | 116 | 2.08 | 0.311 | F1S2Q4 | 816 | I3LRZ4 | 381;404;421 |
|  |  | F1S4N2 | 84 | 2.40 | 0.455 | I3LM30 | 1594;1594 | 8.15 | 0.247 | F1S2V7 | 50 | I3LS02 | 95 |
|  |  | F1S4P0 | 190 | 2.25 | 0.165 | I3LM30 | 2420;2375 | 3.24 | 0.028 | F1S3M9 | 786;235 | I3LS18 | 246;279 |
|  |  | F1S4V5 | 605 | 3.07 | 0.369 | I3LM34 | 973 | 15.15 | 0.016 | F1S3P0 | 93 | I3LSI3 | 184 |
|  |  | F1S4V5 | 689 | 3.01 | 0.338 | I3LMU4 | 205 | 2.70 | 0.050 | F1S3P0 | 95 | I3LSM1 | 363;906 |
|  |  | F1S4V5 | 690 | 2.92 | 0.249 | I3LPY1 | 331 | 2.83 | 0.005 | F1S459 | 206 | I3LSN9 | 64;458 |
|  |  | F1S4V5 | 396 | 2.92 | 0.136 | I3LPY1 | 477 | 5.11 | 0.002 | F1S485 | 564 | I3LT10 | 32 |
|  |  | F1S4V5 | 426 | 3.03 | 0.003 | I3LQU0 | 310 | 3.75 | 0.414 | F1S485 | 475 | I3LT92 | 375 |
|  |  | F1S4V5 | 286 | 5.28 | 0.000 | I3LR51 | 36 | 16.50 | 0.047 | F1S485 | 578 | I3LU02 | 532;850 |
|  |  | F1S554 | 370 | 2.19 | 0.041 | I3LRA8 | 311 | 2.65 | 0.049 | F1S4P0 | 294 | I3LU02 | 250;552 |
|  |  | F1S554 | 512 | 9.38 | 0.015 | I3LRP0 | 300 | 3.40 | 0.196 | F1S4V5 | 458 | I3LU02 | 247;549 |
|  |  | F1S5B0 | 444 | 2.09 | 0.123 | I3LRX7 | 46;230 | 6.93 | 0.000 | F1S4Z9 | 181 | I3LU02 | 554;872 |
|  |  | F1S5L7 | 458 | 2.88 | 0.064 | I3LS02 | 94 | 2.10 | 0.382 | F1S5B0 | 666 | I3LUY9 | 330 |
|  |  | F1S6W7 | 577 | 2.31 | 0.028 | I3LS02 | 98 | 5.43 | 0.046 | F1S5R6 | 824 | I3LUY9 | 934 |
|  |  | F1S6Z6 | 554 | 7.82 | 0.000 | I3LS18 | 208;241 | 3.13 | 0.026 | F1S5R6 | 251 | I3LV20 | 26;194 |
|  |  | F1S7T1 | 2102 | 2.68 | 0.002 | I3LSM1 | 88;626 | 3.30 | 0.147 | F1S5Y7 | 482 | I3LVF3 | 521;580 |
|  |  | F1S7W0 | 1385 | 4.00 | 0.016 | I3LSP1 | 63 | 3.79 | 0.069 | F1S655 | 339 | K7GKF7 | 51 |
|  |  | F1S7X2 | 83 | 2.14 | 0.272 | I3LT56 | 180 | 2.87 | 0.044 | F1S655 | 338 | K7GKF7 | 49 |
|  |  | F1S7Y2 | 409 | 9.39 | 0.044 | I3LTN6 | 526 | 5.18 | 0.001 | F1S732 | 134 | K7GKW0 | 184 |
|  |  | F1S836 | 424 | 3.10 | 0.084 | I3LTN6 | 527 | 5.18 | 0.001 | F1S786 | 404;481 | K7GLA8 | 73;61 |
|  |  | F1S870 | 152 | 6.94 | 0.001 | I3LTU3 | 55;53 | 2.06 | 0.408 | F1S814 | 507 | K7GLA8 | 468;476;476;506 |
|  |  | F1S880 | 140 | 3.94 | 0.068 | I3LU02 | 594;912 | 2.59 | 0.266 | F1S814 | 505 | K7GLK3 | 88;150 |
|  |  | F1S8C6 | 220 | 6.31 | 0.015 | I3LU02 | 585;903 | 2.33 | 0.083 | F1S8C6 | 211 | K7GM47 | 416 |
|  |  | F1S8L9 | 59 | 4.88 | 0.042 | I3LU02 | 404;722 | 3.30 | 0.065 | F1S8C6 | 316 | K7GMV0 | 118 |
|  |  | F1S924 | 141 | 2.69 | 0.456 | I3LU02 | 597;915 | 4.59 | 0.018 | F1S8G6 | 305 | K7GN99 | 82 |
|  |  | F1S994 | 17 | 2.40 | 0.160 | I3LU70 | 138;138 | 5.99 | 0.001 | F1S8Y8 | 927 | K7GNJ7 | 3756;3758 |
|  |  | F1S995 | 564 | 2.25 | 0.213 | I3LUY9 | 1205 | 3.92 | 0.001 | F1S995 | 490 | K7GNJ7 | 2594 |
|  |  | F1S995 | 556 | 4.29 | 0.204 | I3LUY9 | 384 | 9.95 | 0.000 | F1S995 | 1015 | K7GNJ7 | 1367 |
|  |  | F1S995 | 521 | 5.16 | 0.019 | I3LV20 | 27;195 | 4.01 | 0.343 | F1S9D1 | 704 | K7GNY8 | 469;479 |
|  |  | F1S995 | 859 | 5.34 | 0.005 | I3LVD1 | 370;383 | 2.13 | 0.450 | F1S9K5 | 995 | K7GNZ0 | 28;133 |
|  |  | F1S9I0 | 1529 | 7.71 | 0.023 | I3LVK2 | 134 | 4.92 | 0.000 | F1SA50 | 611 | K7GP58 | 264;268 |
|  |  | F1SA44 | 275 | 3.20 | 0.197 | I3LVP5 | 150 | 10.78 | 0.004 | F1SA52 | 458 | K7GRG2 | 63 |
|  |  | F1SA60 | 39 | 2.21 | 0.173 | K7GKW0 | 108;26 | 2.34 | 0.012 | F1SB63 | 551 | K7GRY0 | 820 |
|  |  | F1SA60 | 40 | 2.21 | 0.173 | K7GLK3 | 90;152 | 4.24 | 0.000 | F1SC51 | 131 | O18734 | 128 |
|  |  | F1SAX3 | 15;16 | 3.73 | 0.144 | K7GMN8 | 200;230;231 | 2.61 | 0.025 | F1SCI4 | 186 | P00503 | 138 |
|  |  | F1SBQ5 | 467 | 2.24 | 0.131 | K7GMX1 | 122 | 3.47 | 0.062 | F1SCS1 | 151 | P02540 | 46 |
|  |  | F1SBY0 | 310 | 2.59 | 0.046 | K7GPB6 | 130;752 | 2.26 | 0.009 | F1SCX0 | 288 | P02540 | 47 |
|  |  | F1SBY5 | 1182 | 2.24 | 0.443 | K7GPB6 | 134;756 | 2.26 | 0.009 | F1SDC8 | 1402 | P02547 | 472;473 |
|  |  | F1SBY5 | 1107 | 2.10 | 0.094 | K7GSK7 | 174;176 | 4.32 | 0.068 | F1SDT0 | 97 | P59083 | 36 |
|  |  | F1SBY5 | 424 | 3.96 | 0.004 | K7GSX0 | 666 | 10.89 | 0.000 | F1SDV2 | 1067 | P62895 | 29 |
|  |  | F1SC49 | 205 | 2.21 | 0.020 | P02189 | 104 | 2.01 | 0.412 | F1SDX6 | 60 | P83686 | 137;172 |
|  |  | F1SC49 | 58 | 9.20 | 0.000 | P02189 | 4 | 2.19 | 0.256 | F1SDY8 | 516 | Q06A94 | 22 |
|  |  | F1SCM9 | 351 | 2.32 | 0.133 | P02189 | 71 | 2.03 | 0.186 | F1SE30 | 12 | Q19QU3 | 120 |
|  |  | F1SD97 | 184 | 6.78 | 0.084 | P02189 | 96 | 2.17 | 0.046 | F1SE30 | 1682 | Q29101 | 328 |
|  |  | F1SDC7 | 189 | 9.63 | 0.010 | P02540 | 439 | 4.05 | 0.373 | F1SEJ0 | 403;439 | Q29101 | 255;255 |
|  |  | F1SDV2 | 347 | 2.93 | 0.063 | P02540 | 433 | 8.26 | 0.278 | F1SEJ4 | 837 | Q29101 | 330 |
|  |  | F1SE25 | 66 | 10.54 | 0.000 | P02540 | 31 | 2.17 | 0.274 | F1SEN8 | 282 | Q5GN48 | 3486;473 |
|  |  | F1SEJ4 | 678 | 2.87 | 0.040 | P02540 | 362 | 2.23 | 0.115 | F1SEN8 | 241 | Q5GN48 | 3045 |
|  |  | F1SEN8 | 115 | 2.13 | 0.571 | P02540 | 425 | 5.30 | 0.026 | F1SEN8 | 238 | Q5S1U1 | 178 |
|  |  | F1SEN8 | 243 | 3.14 | 0.122 | P02540 | 23 | 20.58 | 0.007 | F1SGD2 | 828 | Q5S1U1 | 100 |
|  |  | F1SEN8 | 223 | 4.37 | 0.105 | P02540 | 45 | 14.85 | 0.006 | F1SGJ6 | 148 | Q5S1U1 | 85 |
|  |  | F1SEN8 | 121 | 2.28 | 0.052 | P02540 | 32 | 3.16 | 0.003 | F1SH92 | 704;700 | Q684M6 | 372 |
|  |  | F1SEN8 | 116 | 20.46 | 0.049 | P05383 | 263 | 2.05 | 0.313 | F1SHE5 | 869 | Q684M6 | 370 |
|  |  | F1SEN8 | 173 | 2.60 | 0.041 | P07802 | 76 | 4.06 | 0.007 | F1SHR1 | 1281 | Q6DUB7 | 25 |
|  |  | F1SEN8 | 180 | 2.02 | 0.038 | P07802 | 5 | 5.87 | 0.005 | F1SI06 | 31 | Q6Q781 | 57 |
|  |  | F1SEN8 | 182 | 3.05 | 0.021 | P11607 | 649 | 2.13 | 0.309 | F1SI65 | 1199 | Q6Q781 | 312;244;222 |
|  |  | F1SEN8 | 217 | 17.55 | 0.006 | P11607 | 580 | 6.25 | 0.039 | F1SI65 | 2083;2096 | Q6Q781 | 202;134;112 |
|  |  | F1SEN8 | 189 | 19.35 | 0.004 | P11708 | 217;154 | 2.42 | 0.032 | F1SI69 | 148 | Q6Q781 | 311;243;221 |
|  |  | F1SEN8 | 231 | 4.91 | 0.004 | P19620 | 24 | 3.19 | 0.394 | F1SIA4 | 74 | Q6S4N2 | 636;636 |
|  |  | F1SEN8 | 190 | 18.77 | 0.002 | P21999 | 323 | 2.03 | 0.241 | F1SID7 | 1180 | Q7JFN2 | 320 |
|  |  | F1SEN8 | 267 | 453.40 | 0.001 | P26234 | 567 | 2.75 | 0.143 | F1SIZ4 | 242;247 | Q7M2W6 | 21 |
|  |  | F1SFS5 | 487 | 2.12 | 0.058 | P79293 | 445 | 2.33 | 0.622 | F1SJR2 | 502 | Q7YS99 | 198 |
|  |  | F1SGD2 | 820 | 3.63 | 0.009 | P79293 | 70 | 3.87 | 0.336 | F1SJR2 | 533 | Q7YS99 | 118 |
|  |  | F1SH84 | 420 | 2.05 | 0.261 | P79293 | 255 | 11.77 | 0.170 | F1SJR2 | 1625 | Q95266 | 490 |
|  |  | F1SHR2 | 344 | 18.02 | 0.000 | P79293 | 669;1476;1475 | 2.70 | 0.148 | F1SJR2 | 1803 | Q95266 | 277 |
|  |  | F1SHW1 | 540 | 2.05 | 0.208 | P79293 | 792;1599;1598 | 3.23 | 0.093 | F1SJR2 | 1174 | Q95266 | 276 |
|  |  | F1SI65 | 2425;2438 | 2.23 | 0.218 | P79293 | 415;1222;1221 | 2.07 | 0.055 | F1SJR2 | 213 | Q95266 | 492 |
|  |  | F1SID7 | 402 | 2.00 | 0.162 | P79293 | 1084;1891 | 27.65 | 0.043 | F1SJR2 | 928 | Q9GLP1 | 1572 |
|  |  | F1SIL5 | 813 | 6.24 | 0.009 | P79293 | 210 | 12.08 | 0.002 | F1SJR2 | 929 |  |  |
|  |  | F1SIQ0 | 66 | 5.30 | 0.085 | P79293 | 629 | 2.82 | 0.001 | F1SKC4 | 285 |  |  |
|  |  | F1SIU8 | 13 | 2.79 | 0.045 | P80387 | 73;108 | 2.48 | 0.170 | F1SKD6 | 453 |  |  |
|  |  | F1SIW8 | 609 | 5.28 | 0.017 | Q19QT0 | 206 | 2.78 | 0.220 | F1SKP7 | 362 |  |  |
|  |  | F1SJC2 | 40 | 8.68 | 0.031 | Q1KYT0 | 40 | 2.73 | 0.068 | F1SL54 | 859 |  |  |
|  |  | F1SJI1 | 17 | 16.72 | 0.000 | Q29026 | 78 | 2.02 | 0.038 | F1SLA8 | 83 |  |  |
|  |  | F1SJP2 | 377 | 8.82 | 0.003 | Q29101 | 296 | 4.31 | 0.263 | F1SLU6 | 784 |  |  |
|  |  | F1SJR2 | 542 | 2.83 | 0.213 | Q29101 | 306 | 3.33 | 0.004 | F1SLY4 | 45 |  |  |
|  |  | F1SJR2 | 1441 | 5.40 | 0.101 | Q2HYU2 | 775 | 2.56 | 0.047 | F1SM65 | 42 |  |  |
|  |  | F1SJR2 | 138 | 2.12 | 0.057 | Q2VTP6 | 9 | 3.53 | 0.023 | F1SM75 | 353;251 |  |  |
|  |  | F1SJR2 | 205 | 3.19 | 0.031 | Q3ZD69 | 153 | 2.08 | 0.297 | F1SM75 | 86 |  |  |
|  |  | F1SJR2 | 208 | 2.47 | 0.027 | Q3ZD69 | 392;392 | 3.33 | 0.280 | F1SM75 | 354;252 |  |  |
|  |  | F1SJR2 | 485 | 4.91 | 0.009 | Q3ZD69 | 277 | 11.29 | 0.110 | F1SM83 | 703 |  |  |
|  |  | F1SJR2 | 675 | 58.90 | 0.007 | Q53DY5 | 2 | 3.35 | 0.304 | F1SM86 | 461 |  |  |
|  |  | F1SK12 | 1049 | 2.66 | 0.194 | Q53DY5 | 37 | 5.42 | 0.237 | F1SMA1 | 461 |  |  |
|  |  | F1SK12 | 1398 | 2.18 | 0.003 | Q5GN48 | 3602;589;416 | 2.61 | 0.120 | F1SMB6 | 137 |  |  |
|  |  | F1SK44 | 32 | 3.58 | 0.008 | Q5GN48 | 315;316;320 | 8.87 | 0.099 | F1SMN5 | 955 |  |  |
|  |  | F1SK83 | 342 | 2.02 | 0.236 | Q5GN48 | 3541;528;355 | 5.24 | 0.083 | F1SMN5 | 2295 |  |  |
|  |  | F1SKX3 | 442 | 4.06 | 0.131 | Q5GN48 | 3673 | 3.19 | 0.082 | F1SMQ4 | 22 |  |  |
|  |  | F1SLI3 | 629 | 3.75 | 0.008 | Q5GN48 | 3613;600;427 | 2.83 | 0.028 | F1SMR6 | 183 |  |  |
|  |  | F1SLY4 | 158 | 2.48 | 0.210 | Q5GN48 | 3612;599;426 | 2.02 | 0.023 | F1SMW9 | 273 |  |  |
|  |  | F1SLY4 | 155 | 4.77 | 0.092 | Q5GN48 | 3489;476 | 16.54 | 0.009 | F1SNF8 | 1275 |  |  |
|  |  | F1SM39 | 1108 | 2.34 | 0.302 | Q5S1U1 | 15 | 2.36 | 0.097 | F1SNW4 | 161 |  |  |
|  |  | F1SM75 | 83 | 2.04 | 0.582 | Q68J42 | 647 | 3.60 | 0.041 | F1SNW4 | 49 |  |  |
|  |  | F1SM75 | 116 | 2.21 | 0.413 | Q6Q781 | 374;306;284 | 2.67 | 0.102 | F1SNW4 | 42 |  |  |
|  |  | F1SM75 | 1152;956 | 5.33 | 0.187 | Q6Q781 | 509;441;419 | 5.78 | 0.079 | F1SNY5 | 59 |  |  |
|  |  | F1SM75 | 84 | 3.35 | 0.088 | Q6S4N2 | 633 | 17.23 | 0.009 | F1SP12 | 502 |  |  |
|  |  | F1SM86 | 421 | 2.27 | 0.308 | Q7JFN2 | 346;325;325 | 4.96 | 0.050 | F1SP32 | 155 |  |  |
|  |  | F1SMG3 | 65 | 2.00 | 0.145 | Q7M2W6 | 19 | 9.13 | 0.003 | F1SP81 | 324 |  |  |
|  |  | F1SMN5 | 1484 | 4.65 | 0.332 | Q7M2W6 | 59 | 9.36 | 0.001 | F1SQ46 | 286;354 |  |  |
|  |  | F1SMN5 | 2231 | 2.20 | 0.079 | Q7YS99 | 177 | 2.62 | 0.319 | F1SQ46 | 45;45 |  |  |
|  |  | F1SMN5 | 2597 | 9.68 | 0.062 | Q7YS99 | 521 | 3.00 | 0.129 | F1SQ87 | 600 |  |  |
|  |  | F1SMV6 | 234;593 | 14.76 | 0.002 | Q7YS99 | 121 | 4.26 | 0.127 | F1SQK1 | 1000 |  |  |
|  |  | F1SN89 | 937 | 2.11 | 0.038 | Q7YS99 | 206 | 4.42 | 0.065 | F1SQK1 | 457 |  |  |
|  |  | F1SN99 | 2664 | 4.62 | 0.207 | Q95266 | 337 | 2.11 | 0.176 | F1SQK1 | 889 |  |  |
|  |  | F1SNQ1 | 866 | 8.57 | 0.028 | Q95266 | 146 | 4.30 | 0.043 | F1SQK1 | 747 |  |  |
|  |  | F1SNX8 | 12 | 5.72 | 0.106 | Q95283 | 58;58 | 2.78 | 0.324 | F1SQK1 | 187 |  |  |
|  |  | F1SP24 | 13 | 2.54 | 0.062 | Q9GLG4 | 385;385 | 2.50 | 0.442 | F1SQK1 | 309 |  |  |
|  |  |  |  |  |  |  |  |  |  |  |  |  |  |
| sham | baseline | A0A0A0MY57 | 1321;1361 | 2.20 | 0.366 | F1SM75 | 1370;1174 | 3.05 | 0.164 | B6CVL5 | 92 | I3LQU0 | 310 |
|  |  | A0A0A0MY57 | 1322;1362 | 2.20 | 0.366 | F1SM86 | 421 | 2.24 | 0.317 | B6CVL5 | 88 | I3LSM1 | 363;906 |
|  |  | A4GR69 | 109 | 3.16 | 0.260 | F1SM86 | 89 | 3.37 | 0.183 | F1RFI1 | 312 | I3LTH8 | 119;125 |
|  |  | B2ZPK1 | 284 | 2.18 | 0.343 | F1SMA1 | 463 | 2.49 | 0.032 | F1RGH4 | 578 | I3LU60 | 15 |
|  |  | B5U335 | 84 | 5.86 | 0.023 | F1SMN1 | 47 | 22.18 | 0.001 | F1RGH4 | 556 | I3LU70 | 135 |
|  |  | B6VNT8 | 251 | 2.14 | 0.073 | F1SN53 | 24 | 3.60 | 0.006 | F1RGI7 | 768 | I3LVD1 | 331;344 |
|  |  | B6VNT8 | 205 | 2.77 | 0.007 | F1SNF8 | 1275 | 2.15 | 0.217 | F1RGN8 | 124 | K7GL02 | 141;142 |
|  |  | D2SQP3 | 95 | 5.64 | 0.022 | F1SNM7 | 95 | 7.57 | 0.001 | F1RH92 | 225 | K7GL12 | 444 |
|  |  | D4P3C2 | 399 | 2.88 | 0.005 | F1SNX8 | 55 | 6.09 | 0.003 | F1RHM3 | 1940 | K7GM70 | 382;257 |
|  |  | F1RFB2 | 1415 | 2.43 | 0.017 | F1SP24 | 17 | 2.17 | 0.091 | F1RHU2 | 88 | K7GMN8 | 264;294;295 |
|  |  | F1RFV9 | 769 | 2.12 | 0.261 | F1SP82 | 34 | 2.26 | 0.091 | F1RHU2 | 92 | K7GNW1 | 121;292 |
|  |  | F1RFX9 | 205 | 3.06 | 0.011 | F1SPI2 | 37 | 2.26 | 0.178 | F1RHW5 | 966 | K7GNZ3 | 161 |
|  |  | F1RFY6 | 1033 | 2.57 | 0.407 | F1SQL3 | 308;193;202 | 2.65 | 0.049 | F1RI15 | 229 | K7GRF6 | 96;189 |
|  |  | F1RFY6 | 1092 | 2.78 | 0.388 | F1SQN4 | 686;687 | 2.61 | 0.050 | F1RI15 | 230 | K7GS92 | 72;400 |
|  |  | F1RG61 | 682 | 3.53 | 0.054 | F1SQW0 | 141 | 2.07 | 0.034 | F1RIN7 | 372 | P00336 | 238;238 |
|  |  | F1RGH4 | 217 | 2.35 | 0.100 | F1SQX7 | 574;573 | 3.21 | 0.075 | F1RIN7 | 295 | P00355 | 331;107 |
|  |  | F1RGH4 | 472 | 2.41 | 0.008 | F1SRW8 | 334 | 2.30 | 0.216 | F1RIN7 | 296 | P02540 | 359 |
|  |  | F1RHL9 | 624 | 2.12 | 0.279 | F1SS33 | 403;406 | 2.49 | 0.055 | F1RIU9 | 45 | P02540 | 432 |
|  |  | F1RHM3 | 2388 | 3.57 | 0.014 | F1SS77 | 101;109 | 6.26 | 0.004 | F1RJU6 | 512 | P08059 | 212 |
|  |  | F1RHM3 | 2396 | 4.40 | 0.013 | F1SS77 | 59;67;62 | 6.25 | 0.001 | F1RK02 | 6 | P08059 | 215 |
|  |  | F1RIC1 | 47 | 2.23 | 0.186 | F1SSY1 | 99 | 2.70 | 0.172 | F1RK48 | 456 | P08059 | 248 |
|  |  | F1RIP1 | 710 | 3.42 | 0.016 | F1ST03 | 1366 | 2.34 | 0.099 | F1RL66 | 651 | P21999 | 323 |
|  |  | F1RIP6 | 366 | 2.22 | 0.186 | F1ST81 | 299 | 3.92 | 0.048 | F1RL91 | 15 | P29412 | 94;95 |
|  |  | F1RJ25 | 39 | 2.25 | 0.253 | F1ST95 | 37 | 2.62 | 0.007 | F1RLL1 | 602;429;565 | P67872 | 205;225 |
|  |  | F1RJ25 | 36 | 2.08 | 0.229 | F1STQ3 | 693 | 2.01 | 0.322 | F1RLL1 | 603;430;566 | P79293 | 1084;1891;1890 |
|  |  | F1RJU6 | 694 | 5.99 | 0.241 | F1STT6 | 238 | 2.23 | 0.200 | F1RM07 | 1179 | P79293 | 800;1607;1606 |
|  |  | F1RJV5 | 145 | 2.47 | 0.274 | F1SU55 | 893 | 2.62 | 0.185 | F1RPT5 | 315 | P79293 | 386 |
|  |  | F1RK00 | 874 | 2.38 | 0.041 | F1SU55 | 892 | 4.73 | 0.069 | F1RQC5 | 510 | P79293 | 186;993;992 |
|  |  | F1RK48 | 472 | 2.28 | 0.125 | F2Z5B6 | 283;283;283 | 2.70 | 0.291 | F1RQC5 | 627 | Q19QU3 | 120 |
|  |  | F1RK61 | 299 | 2.44 | 0.147 | F2Z5I2 | 136 | 2.27 | 0.005 | F1RQM9 | 2601 | Q29092 | 306 |
|  |  | F1RK87 | 516 | 2.40 | 0.002 | F2Z5I9 | 2;3 | 2.30 | 0.053 | F1RQQ7 | 91 | Q29101 | 326 |
|  |  | F1RKH4 | 24 | 19.13 | 0.000 | I3L548 | 729;738 | 2.34 | 0.072 | F1RQT0 | 368 | Q2HYU1 | 417 |
|  |  | F1RLT0 | 841 | 2.07 | 0.017 | I3L594 | 174;194 | 3.78 | 0.022 | F1RR78 | 2000 | Q68J42 | 552 |
|  |  | F1RM62 | 59;15 | 2.15 | 0.444 | I3L5D5 | 583 | 5.74 | 0.163 | F1RR78 | 874 | Q95266 | 330 |
|  |  | F1RM62 | 157 | 12.48 | 0.026 | I3L5S3 | 346 | 2.51 | 0.136 | F1RRD7 | 812 | Q9N0F1 | 82 |
|  |  | F1RMA6 | 592 | 3.44 | 0.130 | I3L691 | 402 | 2.21 | 0.001 | F1RRD7 | 128 |  |  |
|  |  | F1RN59 | 956 | 2.15 | 0.246 | I3L6D7 | 744;749 | 3.14 | 0.043 | F1RRJ1 | 295;296 |  |  |
|  |  | F1RNP6 | 100 | 2.94 | 0.368 | I3L6D7 | 715;720 | 3.14 | 0.008 | F1RS45 | 1513 |  |  |
|  |  | F1RNW3 | 142 | 2.18 | 0.317 | I3L6Q5 | 2 | 2.66 | 0.008 | F1RSR8 | 419 |  |  |
|  |  | F1RNW3 | 143 | 2.18 | 0.317 | I3L6S5 | 313 | 4.31 | 0.115 | F1RT57 | 100 |  |  |
|  |  | F1RNW3 | 152 | 2.87 | 0.054 | I3L8Q0 | 1366 | 3.15 | 0.196 | F1RTT0 | 152 |  |  |
|  |  | F1RNZ1 | 40 | 2.70 | 0.113 | I3L8Q0 | 831 | 2.20 | 0.074 | F1RU31 | 492 |  |  |
|  |  | F1RPD7 | 726 | 4.91 | 0.090 | I3L8Q0 | 708 | 2.42 | 0.047 | F1RUR3 | 29 |  |  |
|  |  | F1RPS7 | 66 | 2.63 | 0.067 | I3L8Q0 | 259 | 3.33 | 0.010 | F1RUY2 | 470;144 |  |  |
|  |  | F1RPS8 | 45;65 | 2.16 | 0.032 | I3L8Q0 | 766 | 2.32 | 0.008 | F1RVG6 | 731 |  |  |
|  |  | F1RPX2 | 176 | 2.32 | 0.139 | I3L8Q0 | 1368 | 3.02 | 0.004 | F1RVZ0 | 596;604 |  |  |
|  |  | F1RQB6 | 230 | 2.02 | 0.029 | I3L9K5 | 223 | 5.89 | 0.170 | F1RW75 | 1803 |  |  |
|  |  | F1RR22 | 1038;1043 | 2.47 | 0.215 | I3L9K5 | 181 | 2.02 | 0.159 | F1RW75 | 1511 |  |  |
|  |  | F1RR78 | 1031 | 2.15 | 0.251 | I3L9K5 | 214 | 2.43 | 0.028 | F1RW78 | 268 |  |  |
|  |  | F1RR78 | 1029 | 2.50 | 0.153 | I3L9K5 | 19 | 2.61 | 0.009 | F1RWD9 | 301;260 |  |  |
|  |  | F1RR78 | 1197 | 2.39 | 0.031 | I3L9T1 | 2267;2267 | 2.81 | 0.375 | F1RWD9 | 304;263 |  |  |
|  |  | F1RRS8 | 693 | 2.93 | 0.101 | I3L9T1 | 2704;2704 | 2.46 | 0.312 | F1RWF4 | 207 |  |  |
|  |  | F1RRS8 | 741 | 2.04 | 0.033 | I3L9T1 | 2705;2705 | 2.46 | 0.312 | F1RWZ2 | 239 |  |  |
|  |  | F1RRZ3 | 241 | 4.03 | 0.012 | I3L9T1 | 2513;2513 | 2.24 | 0.008 | F1RXG7 | 10 |  |  |
|  |  | F1RS42 | 840 | 6.74 | 0.514 | I3LA44 | 433 | 6.42 | 0.007 | F1RYA4 | 47 |  |  |
|  |  | F1RSG8 | 81 | 2.43 | 0.039 | I3LA95 | 887;1219;1221 | 7.73 | 0.006 | F1RYL9 | 689 |  |  |
|  |  | F1RTR6 | 485 | 3.76 | 0.189 | I3LAQ3 | 33 | 3.52 | 0.197 | F1RYP5 | 825 |  |  |
|  |  | F1RTR6 | 468 | 3.01 | 0.048 | I3LAR5 | 119 | 2.17 | 0.296 | F1RZ24 | 504 |  |  |
|  |  | F1RUY2 | 115 | 2.89 | 0.131 | I3LAR5 | 55 | 2.28 | 0.081 | F1S0X4 | 539 |  |  |
|  |  | F1RVC9 | 37 | 2.38 | 0.001 | I3LAR5 | 23 | 2.72 | 0.005 | F1S146 | 1406 |  |  |
|  |  | F1RVG6 | 669 | 5.41 | 0.036 | I3LAY6 | 16;80 | 3.21 | 0.003 | F1S2N4 | 534 |  |  |
|  |  | F1RWD9 | 479;438 | 2.06 | 0.040 | I3LAY6 | 300;364 | 9.04 | 0.001 | F1S2V7 | 42 |  |  |
|  |  | F1RWD9 | 305;264 | 15.89 | 0.010 | I3LBD0 | 254 | 3.26 | 0.000 | F1S2V7 | 5 |  |  |
|  |  | F1RWF4 | 208 | 2.45 | 0.137 | I3LBD6 | 192 | 6.44 | 0.285 | F1S3P0 | 95 |  |  |
|  |  | F1RWK0 | 216 | 3.54 | 0.041 | I3LC46 | 676 | 4.56 | 0.066 | F1S3P0 | 93 |  |  |
|  |  | F1RWW4 | 41 | 2.24 | 0.212 | I3LCU0 | 13;244 | 2.45 | 0.027 | F1S409 | 606 |  |  |
|  |  | F1RWX6 | 581 | 2.70 | 0.015 | I3LCW3 | 2431 | 2.85 | 0.346 | F1S414 | 943 |  |  |
|  |  | F1RX81 | 674;675 | 2.50 | 0.272 | I3LCW3 | 873 | 2.18 | 0.185 | F1S415 | 292 |  |  |
|  |  | F1RYA4 | 125 | 3.00 | 0.024 | I3LCW3 | 2380 | 2.77 | 0.009 | F1S4R6 | 537 |  |  |
|  |  | F1RYY6 | 237 | 2.25 | 0.039 | I3LDR9 | 216;61 | 2.05 | 0.157 | F1S4V5 | 660 |  |  |
|  |  | F1RZ71 | 85 | 7.05 | 0.162 | I3LDR9 | 331 | 2.12 | 0.138 | F1S4V5 | 432 |  |  |
|  |  | F1RZP9 | 824 | 2.69 | 0.072 | I3LDR9 | 302;147 | 3.40 | 0.070 | F1S5C7 | 423 |  |  |
|  |  | F1RZQ8 | 506 | 2.93 | 0.111 | I3LDS5 | 1297 | 2.74 | 0.015 | F1S5C7 | 425 |  |  |
|  |  | F1RZR9 | 8 | 2.42 | 0.091 | I3LDS5 | 774;745;584 | 9.73 | 0.002 | F1S5F9 | 2637 |  |  |
|  |  | F1RZW0 | 622;462 | 3.45 | 0.095 | I3LDU9 | 95 | 4.88 | 0.001 | F1S5F9 | 2639 |  |  |
|  |  | F1RZW0 | 89 | 4.05 | 0.054 | I3LEJ3 | 1018;1022 | 3.80 | 0.220 | F1S5M9 | 677 |  |  |
|  |  | F1RZW0 | 512;352 | 4.25 | 0.039 | I3LEJ3 | 813;817 | 3.70 | 0.204 | F1S5R6 | 251 |  |  |
|  |  | F1S0V1 | 239;266 | 3.36 | 0.052 | I3LEJ3 | 1053;1062 | 4.67 | 0.121 | F1S5Z3 | 508 |  |  |
|  |  | F1S146 | 1404 | 3.46 | 0.014 | I3LEJ3 | 686;690 | 4.93 | 0.006 | F1S684 | 385 |  |  |
|  |  | F1S156 | 889 | 2.06 | 0.042 | I3LEP9 | 330;344 | 2.17 | 0.055 | F1S6R2 | 213;213 |  |  |
|  |  | F1S156 | 212 | 2.02 | 0.007 | I3LEP9 | 278;292 | 2.32 | 0.023 | F1S786 | 406;483 |  |  |
|  |  | F1S1T6 | 305 | 2.13 | 0.156 | I3LFV4 | 176 | 3.07 | 0.020 | F1S7J6 | 343;339 |  |  |
|  |  | F1S1V5 | 397 | 2.56 | 0.400 | I3LG67 | 146 | 2.18 | 0.147 | F1S7J6 | 344;340 |  |  |
|  |  | F1S288 | 197 | 3.84 | 0.080 | I3LG67 | 144 | 2.09 | 0.023 | F1S7K5 | 237 |  |  |
|  |  | F1S2M0 | 179;182 | 15.75 | 0.002 | I3LGE5 | 1056 | 3.02 | 0.167 | F1S7T1 | 2672 |  |  |
|  |  | F1S2Y2 | 511 | 2.23 | 0.029 | I3LGJ5 | 1373 | 2.52 | 0.145 | F1S7W0 | 1474 |  |  |
|  |  | F1S357 | 130 | 3.38 | 0.010 | I3LHS7 | 96 | 2.20 | 0.153 | F1S7W0 | 380 |  |  |
|  |  | F1S376 | 900 | 2.75 | 0.019 | I3LIB5 | 928 | 4.24 | 0.001 | F1S7Y2 | 554 |  |  |
|  |  | F1S3F2 | 30 | 2.52 | 0.135 | I3LII3 | 57 | 3.86 | 0.040 | F1S8A4 | 795 |  |  |
|  |  | F1S3M9 | 521 | 13.65 | 0.120 | I3LIQ0 | 75 | 3.11 | 0.051 | F1S8P9 | 431 |  |  |
|  |  | F1S3Y2 | 64 | 4.73 | 0.005 | I3LJB8 | 425 | 4.52 | 0.006 | F1SA85 | 634 |  |  |
|  |  | F1S415 | 401 | 6.59 | 0.102 | I3LJE2 | 647 | 2.70 | 0.075 | F1SB18 | 227 |  |  |
|  |  | F1S441 | 565 | 4.61 | 0.001 | I3LK59 | 40 | 2.04 | 0.084 | F1SB28 | 810 |  |  |
|  |  | F1S485 | 784 | 2.38 | 0.104 | I3LKJ3 | 79 | 2.53 | 0.097 | F1SBQ0 | 76 |  |  |
|  |  | F1S4G7 | 973 | 2.03 | 0.193 | I3LKP0 | 868 | 2.16 | 0.061 | F1SC49 | 55 |  |  |
|  |  | F1S4M3 | 990 | 2.21 | 0.028 | I3LM30 | 474 | 2.15 | 0.143 | F1SC49 | 410 |  |  |
|  |  | F1S4M3 | 975 | 6.06 | 0.002 | I3LM43 | 356 | 2.11 | 0.171 | F1SDL5 | 394 |  |  |
|  |  | F1S4N2 | 150 | 3.35 | 0.015 | I3LM43 | 377 | 2.21 | 0.111 | F1SDY9 | 637 |  |  |
|  |  | F1S4V5 | 605 | 2.70 | 0.063 | I3LMH4 | 723 | 3.98 | 0.095 | F1SE30 | 888 |  |  |
|  |  | F1S4V5 | 471 | 3.62 | 0.004 | I3LQ16 | 35;30 | 2.10 | 0.136 | F1SEN8 | 282 |  |  |
|  |  | F1S4Z9 | 189 | 11.80 | 0.000 | I3LQ42 | 8 | 2.74 | 0.096 | F1SF12 | 89 |  |  |
|  |  | F1S4Z9 | 201 | 11.08 | 0.000 | I3LQF9 | 241 | 3.96 | 0.029 | F1SF12 | 74 |  |  |
|  |  | F1S5R6 | 824 | 2.18 | 0.077 | I3LQS0 | 287 | 2.37 | 0.111 | F1SGF0 | 767 |  |  |
|  |  | F1S5R6 | 5 | 5.05 | 0.001 | I3LS02 | 42 | 5.75 | 0.016 | F1SGT3 | 161 |  |  |
|  |  | F1S684 | 512 | 2.04 | 0.149 | I3LS66 | 246 | 3.00 | 0.051 | F1SHU7 | 203;242 |  |  |
|  |  | F1S684 | 397 | 3.66 | 0.005 | I3LSF0 | 461 | 4.37 | 0.004 | F1SIQ0 | 96 |  |  |
|  |  | F1S6Q1 | 114 | 2.14 | 0.156 | I3LSI3 | 184 | 2.62 | 0.232 | F1SJ90 | 281 |  |  |
|  |  | F1S786 | 300;377 | 2.78 | 0.152 | I3LSM7 | 88 | 7.38 | 0.001 | F1SJX1 | 179 |  |  |
|  |  | F1S7V6 | 33 | 2.75 | 0.158 | I3LU60 | 18 | 3.33 | 0.009 | F1SK12 | 25 |  |  |
|  |  | F1S7W0 | 473 | 3.39 | 0.024 | I3LU70 | 127 | 2.19 | 0.076 | F1SK12 | 1297 |  |  |
|  |  | F1S8C1 | 25 | 2.19 | 0.112 | I3LUL5 | 90 | 3.53 | 0.009 | F1SKX3 | 442 |  |  |
|  |  | F1S8D2 | 122 | 2.10 | 0.262 | I3LUR1 | 162 | 2.75 | 0.007 | F1SKX3 | 440 |  |  |
|  |  | F1S8P9 | 465 | 2.39 | 0.018 | I3LVD1 | 341;354 | 2.52 | 0.086 | F1SKZ8 | 86 |  |  |
|  |  | F1S8S9 | 1322 | 2.27 | 0.342 | K7GKW0 | 108 | 2.33 | 0.185 | F1SLR5 | 1535 |  |  |
|  |  | F1S8Y5 | 14 | 4.79 | 0.086 | K7GKW0 | 39 | 2.14 | 0.183 | F1SLY4 | 393 |  |  |
|  |  | F1S995 | 564 | 2.02 | 0.077 | K7GL02 | 228 | 2.59 | 0.001 | F1SLY4 | 186 |  |  |
|  |  | F1S995 | 497 | 2.15 | 0.004 | K7GLE2 | 182;338;395 | 3.46 | 0.192 | F1SM00 | 359;313 |  |  |
|  |  | F1S9D1 | 413 | 3.12 | 0.006 | K7GMG7 | 128;180;105 | 2.57 | 0.035 | F1SM75 | 86 |  |  |
|  |  | F1SA66 | 134 | 14.22 | 0.000 | K7GMJ6 | 110;116 | 2.22 | 0.100 | F1SM75 | 224;122 |  |  |
|  |  | F1SAM8 | 1636 | 2.75 | 0.067 | K7GMJ9 | 93;193;230;275 | 3.10 | 0.015 | F1SMQ5 | 324 |  |  |
|  |  | F1SB57 | 45 | 2.07 | 0.289 | K7GMN8 | 262;292;293 | 2.49 | 0.052 | F1SMQ7 | 327 |  |  |
|  |  | F1SBQ5 | 213 | 3.35 | 0.352 | K7GMN8 | 201;231;232 | 3.00 | 0.050 | F1SMZ7 | 70 |  |  |
|  |  | F1SBQ5 | 227 | 3.14 | 0.048 | K7GNW0 | 115 | 3.95 | 0.410 | F1SQK1 | 187 |  |  |
|  |  | F1SBQ5 | 484 | 3.59 | 0.000 | K7GNZ3 | 166 | 4.36 | 0.049 | F1SQK9 | 254 |  |  |
|  |  | F1SBY5 | 391 | 2.45 | 0.037 | K7GP58 | 126;130 | 2.59 | 0.093 | F1SR84 | 18 |  |  |
|  |  | F1SBY5 | 1274 | 5.49 | 0.002 | O18734 | 128 | 2.18 | 0.148 | F1SS24 | 286 |  |  |
|  |  | F1SC51 | 91 | 3.07 | 0.041 | O18734 | 26 | 6.90 | 0.056 | F1SSL6 | 36 |  |  |
|  |  | F1SCI4 | 185 | 3.54 | 0.101 | P00336 | 18;18 | 2.14 | 0.136 | F1SSY1 | 194 |  |  |
|  |  | F1SCM9 | 296 | 2.42 | 0.378 | P00355 | 182 | 2.10 | 0.536 | F1SSY1 | 193 |  |  |
|  |  | F1SCM9 | 351 | 3.24 | 0.142 | P00503 | 138 | 3.18 | 0.336 | F1ST98 | 95 |  |  |
|  |  | F1SDF8 | 81 | 4.12 | 0.041 | P00503 | 312 | 2.75 | 0.118 | F1SU55 | 440 |  |  |
|  |  | F1SDT0 | 101 | 2.36 | 0.049 | P00889 | 226 | 2.02 | 0.150 | F1SUP1 | 44 |  |  |
|  |  | F1SDW4 | 134 | 2.07 | 0.258 | P02189 | 68 | 2.27 | 0.160 | F1SUP1 | 42 |  |  |
|  |  | F1SDX6 | 60 | 2.53 | 0.121 | P02189 | 71 | 2.50 | 0.083 | F1SUZ2 | 771 |  |  |
|  |  | F1SDX6 | 68 | 3.26 | 0.111 | P02189 | 133 | 2.19 | 0.058 | F1SV04 | 45 |  |  |
|  |  | F1SEA2 | 48 | 2.82 | 0.026 | P02540 | 28 | 3.79 | 0.056 | F1SV26 | 15 |  |  |
|  |  | F1SEJ4 | 116 | 2.14 | 0.041 | P02547 | 472;473 | 2.96 | 0.319 | F1SV46 | 402;355;356 |  |  |
|  |  | F1SF57 | 248 | 2.21 | 0.036 | P06199 | 232 | 10.67 | 0.028 | F2Z5B6 | 215 |  |  |
|  |  | F1SFC9 | 741 | 2.12 | 0.109 | P07802 | 82 | 3.30 | 0.001 | I3L5H6 | 311 |  |  |
|  |  | F1SFF6 | 245 | 3.43 | 0.004 | P08059 | 532 | 2.66 | 0.614 | I3L5H6 | 230 |  |  |
|  |  | F1SFI5 | 265 | 2.08 | 0.428 | P11607 | 553 | 2.26 | 0.007 | I3L606 | 308;331 |  |  |
|  |  | F1SFP6 | 344 | 2.40 | 0.077 | P29269 | 20 | 2.84 | 0.081 | I3L606 | 309;332 |  |  |
|  |  | F1SFQ4 | 188 | 2.90 | 0.091 | P61013 | 16 | 2.61 | 0.367 | I3L6D7 | 694;699 |  |  |
|  |  | F1SGD2 | 6 | 2.05 | 0.042 | P61013 | 17 | 6.69 | 0.013 | I3L8K4 | 113 |  |  |
|  |  | F1SGD2 | 151 | 2.21 | 0.005 | P79274 | 55 | 2.46 | 0.041 | I3L8Q0 | 1167 |  |  |
|  |  | F1SH10 | 145 | 2.00 | 0.196 | P79293 | 1192 | 2.35 | 0.299 | I3L8Q0 | 255 |  |  |
|  |  | F1SHF6 | 357 | 2.20 | 0.102 | P79293 | 711;1518 | 2.19 | 0.201 | I3L8X2 | 339 |  |  |
|  |  | F1SHL0 | 493 | 4.12 | 0.006 | P79293 | 70 | 2.23 | 0.182 | I3L9T1 | 2115 |  |  |
|  |  | F1SHL9 | 46 | 2.73 | 0.291 | P79293 | 212;1019;1018 | 2.57 | 0.177 | I3L9T1 | 589;589 |  |  |
|  |  | F1SHL9 | 86 | 2.10 | 0.016 | P79293 | 915;1722;1721 | 2.96 | 0.137 | I3LA73 | 95;737 |  |  |
|  |  | F1SHW1 | 540 | 2.16 | 0.183 | P79293 | 378;378 | 2.32 | 0.136 | I3LA95 | 943;1275 |  |  |
|  |  | F1SI24 | 110 | 3.02 | 0.011 | P79293 | 891;1698 | 2.40 | 0.102 | I3LAR5 | 141 |  |  |
|  |  | F1SI65 | 2083;2096 | 3.33 | 0.014 | P79293 | 1370;559 | 3.17 | 0.073 | I3LB67 | 65;421 |  |  |
|  |  | F1SIB9 | 239 | 2.09 | 0.215 | P79293 | 1261;1260 | 2.03 | 0.072 | I3LC46 | 624 |  |  |
|  |  | F1SIB9 | 431;428 | 2.25 | 0.108 | P79293 | 735;1542;1541 | 2.69 | 0.041 | I3LCT5 | 1183 |  |  |
|  |  | F1SID1 | 429 | 2.81 | 0.006 | P79293 | 748 | 2.85 | 0.019 | I3LCW3 | 1301 |  |  |
|  |  | F1SID7 | 273 | 2.18 | 0.126 | P79293 | 415;1222;1221 | 7.81 | 0.000 | I3LCW3 | 872 |  |  |
|  |  | F1SID7 | 1018 | 2.53 | 0.081 | Q06A98 | 25 | 9.49 | 0.360 | I3LEP9 | 319;333 |  |  |
|  |  | F1SIK9 | 406 | 3.18 | 0.045 | Q06A98 | 26 | 2.18 | 0.008 | I3LEZ7 | 53 |  |  |
|  |  | F1SJG2 | 323 | 2.70 | 0.240 | Q06AA0 | 305 | 2.16 | 0.042 | I3LEZ7 | 54 |  |  |
|  |  | F1SJI2 | 171;204 | 4.94 | 0.180 | Q19QU3 | 153 | 4.18 | 0.005 | I3LG94 | 742 |  |  |
|  |  | F1SJI2 | 129;162 | 3.65 | 0.008 | Q2HYU1 | 148 | 4.15 | 0.370 | I3LGB5 | 360 |  |  |
|  |  | F1SJR2 | 1687 | 2.64 | 0.077 | Q3YLA6 | 199 | 3.00 | 0.030 | I3LGE5 | 758 |  |  |
|  |  | F1SK12 | 1324 | 4.08 | 0.005 | Q3ZD69 | 390 | 8.40 | 0.071 | I3LH91 | 228 |  |  |
|  |  | F1SK44 | 86 | 2.62 | 0.211 | Q5S1U1 | 84 | 2.94 | 0.332 | I3LI05 | 4 |  |  |
|  |  | F1SKB1 | 361;361 | 2.97 | 0.270 | Q68J42 | 554 | 3.36 | 0.195 | I3LIB5 | 813 |  |  |
|  |  | F1SL54 | 859 | 3.50 | 0.109 | Q71LE2 | 81 | 3.02 | 0.036 | I3LLF1 | 3 |  |  |
|  |  | F1SLF0 | 219 | 7.53 | 0.020 | Q8WNW3 | 665 | 2.07 | 0.259 | I3LM30 | 2469;2424 |  |  |
|  |  | F1SLI3 | 736 | 4.34 | 0.183 | Q95266 | 337 | 2.52 | 0.014 | I3LM43 | 374 |  |  |
|  |  | F1SLY4 | 15 | 3.81 | 0.020 | Q9MZ16 | 215 | 2.01 | 0.310 | I3LMJ1 | 88 |  |  |
|  |  | F1SM75 | 116 | 4.15 | 0.244 | Q9TUZ0 | 699 | 2.48 | 0.247 | I3LNF8 | 932 |  |  |
|  |  |  |  |  |  |  |  |  |  |  |  |  |  |
|  | early reperfusion | A9LM01 | 90 | 6.15 | 0.015 | F1SKQ0 | 603 | 2.06 | 0.589 | F1RFB2 | 1155 | I3L801 | 125 |
|  |  | A9LM01 | 104;46 | 43.30 | 0.000 | F1SKQ0 | 605 | 2.06 | 0.589 | F1RFJ8 | 508;549 | I3L806 | 596 |
|  |  | B0KYV5 | 365;135 | 2.43 | 0.160 | F1SKX3 | 42 | 2.91 | 0.169 | F1RFJ8 | 507;548 | I3L806 | 801 |
|  |  | C1PIG4 | 392 | 3.90 | 0.186 | F1SM75 | 84 | 2.04 | 0.023 | F1RFY6 | 676 | I3L9K5 | 58 |
|  |  | C1PIG4 | 393 | 3.67 | 0.180 | F1SMN5 | 2037 | 2.19 | 0.120 | F1RG67 | 196 | I3L9T1 | 2112 |
|  |  | D2JYW4 | 263;263 | 3.53 | 0.004 | F1SMN5 | 2231 | 2.13 | 0.054 | F1RG90 | 37 | I3L9T1 | 3208;3213 |
|  |  | F1REW5 | 129 | 5.95 | 0.009 | F1SMV6 | 234;593 | 7.27 | 0.001 | F1RGE7 | 256 | I3L9T1 | 2562 |
|  |  | F1RFX9 | 203 | 2.27 | 0.211 | F1SN53 | 18 | 7.09 | 0.034 | F1RHA0 | 304;340 | I3L9T1 | 217 |
|  |  | F1RGD9 | 27 | 2.23 | 0.029 | F1SP32 | 160 | 7.45 | 0.004 | F1RHC6 | 55 | I3L9T1 | 2716 |
|  |  | F1RGD9 | 28 | 4.13 | 0.009 | F1SP81 | 324 | 10.41 | 0.003 | F1RHJ3 | 157 | I3L9T1 | 1425 |
|  |  | F1RGE7 | 282 | 2.67 | 0.082 | F1SPR6 | 711;706 | 2.52 | 0.021 | F1RHL9 | 179 | I3L9T1 | 118 |
|  |  | F1RGH4 | 581 | 24.07 | 0.010 | F1SQ16 | 1263 | 4.05 | 0.132 | F1RHM8 | 60 | I3L9T1 | 1424 |
|  |  | F1RGJ2 | 690 | 2.48 | 0.295 | F1SQ46 | 45 | 12.53 | 0.004 | F1RIC1 | 65 | I3LA95 | 1199;1531 |
|  |  | F1RGP1 | 1283 | 2.18 | 0.103 | F1SQK1 | 870 | 4.25 | 0.016 | F1RIC1 | 63 | I3LAR5 | 421 |
|  |  | F1RH92 | 496 | 6.16 | 0.007 | F1SQK1 | 367 | 5.72 | 0.010 | F1RIP1 | 730 | I3LAR5 | 351 |
|  |  | F1RH92 | 145 | 10.65 | 0.000 | F1SQP0 | 729 | 4.04 | 0.215 | F1RIU0 | 128 | I3LC03 | 118 |
|  |  | F1RHG1 | 1253 | 5.65 | 0.007 | F1SS29 | 45 | 2.95 | 0.012 | F1RJN0 | 217 | I3LCW3 | 1369 |
|  |  | F1RHM3 | 1937 | 2.21 | 0.052 | F1SSL6 | 250 | 2.47 | 0.076 | F1RK61 | 209 | I3LCZ5 | 65 |
|  |  | F1RI15 | 76 | 43.43 | 0.005 | F1SSP0 | 15 | 5.56 | 0.001 | F1RKG8 | 52 | I3LDS5 | 1342;1305 |
|  |  | F1RII7 | 73 | 2.28 | 0.117 | F1ST39 | 654 | 2.67 | 0.001 | F1RLE5 | 6 | I3LDS5 | 273;278 |
|  |  | F1RIP1 | 731 | 4.71 | 0.009 | F1STQ3 | 703 | 2.48 | 0.068 | F1RLP5 | 147 | I3LFI3 | 321 |
|  |  | F1RIP1 | 727 | 4.88 | 0.000 | F1SU55 | 744 | 4.58 | 0.038 | F1RLP5 | 670 | I3LFV4 | 209 |
|  |  | F1RIW3 | 223 | 2.94 | 0.189 | F1SU55 | 939 | 3.11 | 0.001 | F1RLV6 | 532 | I3LG94 | 1117 |
|  |  | F1RJ93 | 172 | 4.86 | 0.002 | F1SV22 | 3871 | 3.18 | 0.504 | F1RNW3 | 154 | I3LI16 | 226 |
|  |  | F1RKG8 | 185 | 13.66 | 0.001 | F2Z4X6 | 136 | 8.07 | 0.006 | F1RPY3 | 30 | I3LKL3 | 364 |
|  |  | F1RKJ3 | 32 | 2.24 | 0.131 | F2Z5Q6 | 238;235 | 3.67 | 0.015 | F1RRE0 | 162 | I3LKP0 | 406 |
|  |  | F1RLG5 | 189 | 2.15 | 0.174 | F6PSL2 | 130 | 2.48 | 0.132 | F1RSD1 | 60 | I3LKP0 | 1196 |
|  |  | F1RLG5 | 188 | 2.53 | 0.110 | I3L5C0 | 91 | 12.27 | 0.004 | F1RTB3 | 50 | I3LKP0 | 556 |
|  |  | F1RLP5 | 619 | 4.20 | 0.001 | I3L5D5 | 292 | 4.79 | 0.065 | F1RTB6 | 115 | I3LKP0 | 451 |
|  |  | F1RMC1 | 372 | 2.87 | 0.055 | I3L6C1 | 841 | 4.76 | 0.008 | F1RU38 | 327 | I3LKP0 | 577 |
|  |  | F1RN89 | 635 | 3.59 | 0.023 | I3L6H7 | 220 | 4.11 | 0.014 | F1RUJ3 | 768 | I3LKU2 | 46 |
|  |  | F1RNP6 | 504 | 2.39 | 0.133 | I3L6H7 | 218 | 3.67 | 0.009 | F1RVD4 | 528 | I3LLD6 | 120 |
|  |  | F1RQ09 | 108 | 3.37 | 0.013 | I3L788 | 237 | 2.09 | 0.124 | F1RVD4 | 804 | I3LM34 | 973 |
|  |  | F1RR06 | 47 | 2.54 | 0.313 | I3L8D7 | 598 | 2.05 | 0.106 | F1RVG6 | 253 | I3LQP1 | 172;170 |
|  |  | F1RR47 | 276 | 2.03 | 0.266 | I3L8Q0 | 829 | 3.49 | 0.045 | F1RW75 | 1031 | I3LQP1 | 175;173 |
|  |  | F1RRN1 | 457 | 3.42 | 0.111 | I3L8Q0 | 1965 | 5.73 | 0.022 | F1RW75 | 2613 | I3LRZ4 | 585;608;625 |
|  |  | F1RU38 | 328 | 13.37 | 0.021 | I3L8Q0 | 354 | 6.18 | 0.021 | F1RW75 | 2404 | I3LS18 | 246;279 |
|  |  | F1RUJ0 | 202 | 5.56 | 0.001 | I3L8Q0 | 800 | 8.09 | 0.012 | F1RWD9 | 1372;1242 | I3LSM7 | 296 |
|  |  | F1RVG6 | 950 | 5.69 | 0.019 | I3L8Q0 | 1343 | 5.13 | 0.010 | F1RWW4 | 111 | I3LT10 | 32 |
|  |  | F1RWC5 | 930 | 5.06 | 0.006 | I3L8Q0 | 355 | 4.60 | 0.003 | F1RWW4 | 225 | I3LT92 | 375 |
|  |  | F1RWD9 | 890;760 | 2.01 | 0.217 | I3L8Q0 | 1738 | 7.78 | 0.001 | F1RX46 | 546 | I3LTN6 | 526 |
|  |  | F1RWD9 | 1316;1186 | 2.16 | 0.173 | I3L9R9 | 594 | 2.12 | 0.195 | F1RX74 | 189 | I3LU02 | 554;872 |
|  |  | F1RWD9 | 245;231 | 2.20 | 0.092 | I3L9T1 | 2040 | 2.69 | 0.196 | F1RXZ6 | 267 | I3LU02 | 532;850 |
|  |  | F1RWD9 | 520;479 | 8.04 | 0.040 | I3L9T1 | 1899 | 2.31 | 0.173 | F1RXZ6 | 273 | I3LU02 | 250;552 |
|  |  | F1RWD9 | 1091;961 | 5.94 | 0.005 | I3L9T1 | 1901 | 2.31 | 0.173 | F1RYS9 | 207 | I3LV20 | 26;194 |
|  |  | F1RWE1 | 1163;1168 | 2.36 | 0.242 | I3L9T1 | 1811 | 2.33 | 0.067 | F1RZ24 | 22 | I3LVF3 | 521;580 |
|  |  | F1RWW4 | 124 | 2.24 | 0.060 | I3L9T1 | 1612 | 2.14 | 0.045 | F1RZU8 | 2603 | K7GKW0 | 184 |
|  |  | F1RWW4 | 171 | 3.74 | 0.013 | I3L9T1 | 2503 | 12.23 | 0.042 | F1RZW0 | 269;109 | K7GL02 | 106;107 |
|  |  | F1RWW4 | 326 | 3.26 | 0.007 | I3L9T1 | 1544 | 2.66 | 0.033 | F1RZW0 | 431;271 | K7GLA8 | 73;61 |
|  |  | F1RX74 | 29 | 16.08 | 0.001 | I3L9T1 | 3250;3255 | 5.01 | 0.010 | F1S078 | 137 | K7GLA8 | 468;476 |
|  |  | F1RXG7 | 13 | 2.21 | 0.009 | I3L9T1 | 265 | 19.51 | 0.001 | F1S156 | 250 | K7GNJ7 | 3564;3566 |
|  |  | F1RXZ2 | 63;88 | 2.77 | 0.055 | I3L9T1 | 2451 | 5.30 | 0.001 | F1S156 | 229 | K7GRY0 | 820 |
|  |  | F1RXZ2 | 71;96 | 6.52 | 0.013 | I3L9T1 | 2227 | 5.12 | 0.001 | F1S156 | 190 | P02540 | 439 |
|  |  | F1RYA1 | 182 | 3.66 | 0.043 | I3LAR5 | 318 | 2.96 | 0.143 | F1S2Q4 | 816 | P02540 | 46 |
|  |  | F1RYA4 | 5 | 7.49 | 0.003 | I3LAR5 | 415 | 4.08 | 0.055 | F1S2V7 | 50 | P02540 | 47 |
|  |  | F1RYS9 | 173 | 15.56 | 0.000 | I3LAR5 | 127 | 3.56 | 0.020 | F1S376 | 902 | P07802 | 5;5 |
|  |  | F1RZH1 | 39 | 6.10 | 0.007 | I3LAR5 | 360 | 4.57 | 0.020 | F1S3I5 | 308 | P11607 | 648 |
|  |  | F1RZN9 | 84 | 2.41 | 0.209 | I3LAR5 | 243 | 3.06 | 0.019 | F1S448 | 792 | P11708 | 189;126;21;65 |
|  |  | F1RZQ6 | 42 | 12.26 | 0.000 | I3LAR5 | 422 | 5.12 | 0.009 | F1S485 | 564 | P79293 | 656;1463;1462 |
|  |  | F1RZU8 | 186 | 2.01 | 0.043 | I3LAR5 | 63 | 6.74 | 0.002 | F1S4G7 | 656 | P79293 | 1764 |
|  |  | F1RZW0 | 739 | 2.27 | 0.266 | I3LAR5 | 31 | 14.51 | 0.001 | F1S4P0 | 294 | P80310 | 25;25 |
|  |  | F1RZW0 | 443;283 | 11.93 | 0.002 | I3LAR5 | 297 | 5.81 | 0.000 | F1S4Z9 | 181 | P83686 | 137;172 |
|  |  | F1S078 | 141 | 2.99 | 0.023 | I3LAY6 | 248;312 | 29.58 | 0.000 | F1S554 | 512 | Q06A94 | 22 |
|  |  | F1S087 | 95 | 3.33 | 0.088 | I3LBD6 | 21 | 7.80 | 0.000 | F1S5B0 | 29 | Q29101 | 255 |
|  |  | F1S0L8 | 298 | 2.36 | 0.110 | I3LCW3 | 1153 | 41.52 | 0.021 | F1S5Y7 | 482 | Q29221 | 9 |
|  |  | F1S146 | 3848 | 2.57 | 0.029 | I3LDU9 | 129 | 2.95 | 0.102 | F1S6W7 | 577 | Q5GN48 | 3541;528;355 |
|  |  | F1S156 | 351 | 13.61 | 0.147 | I3LFI3 | 330 | 9.83 | 0.008 | F1S7T1 | 872 | Q5S1U1 | 100 |
|  |  | F1S1U5 | 451 | 3.61 | 0.023 | I3LGU7 | 182 | 3.62 | 0.002 | F1S8G6 | 305 | Q684M6 | 370 |
|  |  | F1S261 | 244 | 4.88 | 0.018 | I3LGU7 | 186 | 3.94 | 0.001 | F1S8P4 | 1950 | Q6Q781 | 202;134;112 |
|  |  | F1S269 | 159 | 2.65 | 0.047 | I3LH91 | 3 | 2.69 | 0.021 | F1S995 | 1015 | Q7JFN2 | 346;325;325 |
|  |  | F1S3M9 | 786;235 | 4.62 | 0.046 | I3LIB5 | 815 | 20.47 | 0.080 | F1S9K5 | 995 | Q7JFN2 | 325 |
|  |  | F1S415 | 379 | 2.55 | 0.147 | I3LIV0 | 25 | 5.09 | 0.000 | F1SBU9 | 510 | Q7M2W6 | 21 |
|  |  | F1S441 | 584 | 3.50 | 0.301 | I3LKE2 | 223 | 17.33 | 0.001 | F1SBY5 | 1272 |  |  |
|  |  | F1S445 | 272 | 3.95 | 0.003 | I3LKP0 | 1008 | 6.08 | 0.058 | F1SC51 | 137 |  |  |
|  |  | F1S485 | 475 | 3.19 | 0.067 | I3LKP0 | 722 | 3.01 | 0.041 | F1SCS1 | 151 |  |  |
|  |  | F1S485 | 667 | 4.97 | 0.002 | I3LKP0 | 1020 | 4.84 | 0.012 | F1SCX0 | 288 |  |  |
|  |  | F1S4J0 | 120 | 2.55 | 0.054 | I3LKP0 | 873 | 3.64 | 0.003 | F1SE30 | 1682;162 |  |  |
|  |  | F1S4P0 | 276 | 3.47 | 0.003 | I3LKP0 | 1250 | 8.23 | 0.001 | F1SEN8 | 238 |  |  |
|  |  | F1S4V5 | 726 | 2.09 | 0.321 | I3LKP0 | 738 | 15.09 | 0.001 | F1SEN8 | 217 |  |  |
|  |  | F1S4V5 | 286 | 2.84 | 0.006 | I3LKP0 | 580 | 8.66 | 0.001 | F1SEN8 | 241 |  |  |
|  |  | F1S4Z9 | 200 | 4.97 | 0.050 | I3LM30 | 1594;1594 | 5.13 | 0.007 | F1SEV8 | 448;86 |  |  |
|  |  | F1S4Z9 | 101 | 3.88 | 0.016 | I3LM30 | 2420;2375 | 3.27 | 0.000 | F1SFI4 | 331 |  |  |
|  |  | F1S554 | 515 | 2.09 | 0.311 | I3LMU4 | 205 | 3.70 | 0.025 | F1SFW7 | 614;615 |  |  |
|  |  | F1S5B0 | 637 | 2.95 | 0.020 | I3LPS9 | 33 | 30.11 | 0.002 | F1SGD2 | 828 |  |  |
|  |  | F1S5P2 | 347 | 2.42 | 0.046 | I3LPY1 | 477 | 3.07 | 0.010 | F1SHR1 | 1281 |  |  |
|  |  | F1S6Z6 | 554 | 3.57 | 0.000 | I3LRA8 | 311;311 | 2.59 | 0.206 | F1SI09 | 12 |  |  |
|  |  | F1S7T1 | 871 | 4.32 | 0.101 | I3LRX7 | 46;230 | 4.92 | 0.008 | F1SI65 | 1199 |  |  |
|  |  | F1S7T1 | 2102 | 2.80 | 0.060 | I3LSN9 | 64;458 | 3.98 | 0.061 | F1SID7 | 1180 |  |  |
|  |  | F1S7Y2 | 530 | 2.09 | 0.159 | I3LU02 | 247;549;481 | 2.22 | 0.411 | F1SIY9 | 223 |  |  |
|  |  | F1S814 | 117 | 2.53 | 0.421 | I3LU02 | 594;912;825 | 2.42 | 0.040 | F1SIY9 | 224 |  |  |
|  |  | F1S814 | 115 | 2.98 | 0.086 | I3LU02 | 404;722;654 | 2.95 | 0.007 | F1SJR2 | 675 |  |  |
|  |  | F1S870 | 152 | 11.50 | 0.009 | I3LU02 | 597;915;828 | 4.07 | 0.002 | F1SJR2 | 1440 |  |  |
|  |  | F1S8C6 | 222 | 8.34 | 0.096 | I3LU70 | 138;138 | 7.06 | 0.004 | F1SJR2 | 1174 |  |  |
|  |  | F1S8C6 | 211 | 5.42 | 0.067 | I3LUY9 | 934 | 9.58 | 0.007 | F1SJR2 | 501 |  |  |
|  |  | F1S8C6 | 202 | 8.24 | 0.003 | I3LUY9 | 1205 | 4.36 | 0.006 | F1SJR2 | 1625 |  |  |
|  |  | F1S8C6 | 220 | 8.31 | 0.000 | I3LUY9 | 384 | 5.28 | 0.001 | F1SJS9 | 462 |  |  |
|  |  | F1S8L9 | 59 | 2.24 | 0.004 | I3LVP5 | 150 | 5.70 | 0.026 | F1SK12 | 1049 |  |  |
|  |  | F1S924 | 141 | 4.53 | 0.005 | I7KJP5 | 134 | 2.17 | 0.056 | F1SKL6 | 62 |  |  |
|  |  | F1S995 | 859 | 2.26 | 0.008 | K7GKF7 | 51 | 2.13 | 0.237 | F1SLA8 | 83 |  |  |
|  |  | F1S9I0 | 1529 | 5.69 | 0.039 | K7GLK3 | 88;150 | 2.72 | 0.062 | F1SLU6 | 624 |  |  |
|  |  | F1SA44 | 275 | 2.26 | 0.216 | K7GLK3 | 90;152 | 2.42 | 0.018 | F1SLU6 | 784 |  |  |
|  |  | F1SAX3 | 15;16 | 9.93 | 0.002 | K7GM47 | 416 | 4.18 | 0.087 | F1SLY4 | 158 |  |  |
|  |  | F1SBY5 | 424 | 3.17 | 0.006 | K7GMX1 | 122 | 2.71 | 0.082 | F1SM65 | 42 |  |  |
|  |  | F1SC49 | 58 | 2.69 | 0.038 | K7GN99 | 82;82 | 6.16 | 0.005 | F1SMA1 | 461 |  |  |
|  |  | F1SCV8 | 1008 | 2.26 | 0.324 | K7GNJ7 | 3756;3758 | 8.89 | 0.000 | F1SMB6 | 137 |  |  |
|  |  | F1SD97 | 184 | 5.26 | 0.126 | K7GP58 | 264;268 | 2.34 | 0.052 | F1SMN5 | 2597 |  |  |
|  |  | F1SDQ0 | 363 | 2.93 | 0.050 | K7GSK7 | 174;176 | 2.82 | 0.180 | F1SMQ4 | 22 |  |  |
|  |  | F1SDQ0 | 365 | 3.46 | 0.010 | K7GSX0 | 666 | 6.02 | 0.013 | F1SMW9 | 273 |  |  |
|  |  | F1SE25 | 66 | 2.28 | 0.235 | P02540 | 60 | 2.16 | 0.160 | F1SNW4 | 42 |  |  |
|  |  | F1SE30 | 12 | 11.22 | 0.002 | P02540 | 59 | 2.16 | 0.160 | F1SNX8 | 12 |  |  |
|  |  | F1SEJ0 | 403;439 | 7.44 | 0.000 | P02540 | 425 | 3.64 | 0.073 | F1SP12 | 502 |  |  |
|  |  | F1SEN8 | 180 | 2.15 | 0.295 | P02540 | 23 | 8.56 | 0.017 | F1SP32 | 155 |  |  |
|  |  | F1SEN8 | 223 | 3.02 | 0.077 | P02540 | 45 | 10.27 | 0.001 | F1SPM7 | 243;261 |  |  |
|  |  | F1SEN8 | 243 | 2.31 | 0.065 | P02543 | 426 | 4.68 | 0.135 | F1SQ16 | 1261 |  |  |
|  |  | F1SEN8 | 189 | 9.62 | 0.058 | P02543 | 419 | 5.42 | 0.061 | F1SQ46 | 310;378 |  |  |
|  |  | F1SEN8 | 231 | 3.74 | 0.020 | P02543 | 420 | 5.42 | 0.061 | F1SQ87 | 600 |  |  |
|  |  | F1SEN8 | 116 | 5.66 | 0.004 | P07802 | 76 | 2.13 | 0.031 | F1SQI6 | 154 |  |  |
|  |  | F1SEN8 | 267 | 12.48 | 0.001 | P11607 | 580 | 2.57 | 0.116 | F1SQK1 | 1000 |  |  |
|  |  | F1SEN8 | 190 | 19.72 | 0.001 | P59083 | 36 | 18.23 | 0.007 | F1SQK1 | 457 |  |  |
|  |  | F1SGD2 | 291 | 2.18 | 0.240 | P79293 | 210 | 9.09 | 0.013 | F1SQK1 | 747 |  |  |
|  |  | F1SGD2 | 820 | 3.43 | 0.061 | Q29101 | 306 | 2.38 | 0.051 | F1SQK1 | 889 |  |  |
|  |  | F1SGJ3 | 106 | 3.71 | 0.114 | Q3ZD69 | 636 | 3.14 | 0.177 | F1SS33 | 392;395 |  |  |
|  |  | F1SGS6 | 306 | 6.80 | 0.002 | Q5GN48 | 3613;600;427 | 2.43 | 0.202 | F1SU33 | 268 |  |  |
|  |  | F1SHR2 | 344 | 21.34 | 0.003 | Q5GN48 | 3673 | 3.02 | 0.068 | F1SU85 | 459 |  |  |
|  |  | F1SI09 | 24 | 6.34 | 0.003 | Q5GN48 | 3486;473 | 3.94 | 0.039 | F1SUD5 | 618 |  |  |
|  |  | F1SI69 | 148 | 3.26 | 0.161 | Q5S1U1 | 15 | 2.44 | 0.046 | F1SUH2 | 269 |  |  |
|  |  | F1SID7 | 282 | 2.37 | 0.471 | Q5S1U1 | 178 | 6.46 | 0.000 | F1SUX4 | 1995 |  |  |
|  |  | F1SIL5 | 813 | 3.46 | 0.027 | Q684M6 | 372 | 3.48 | 0.053 | F1SUZ2 | 552 |  |  |
|  |  | F1SIQ0 | 66 | 4.82 | 0.002 | Q6Q781 | 374;306;284 | 2.20 | 0.112 | F1SUZ4 | 257 |  |  |
|  |  | F1SJC2 | 40 | 4.34 | 0.163 | Q6Q781 | 312;244;222 | 5.81 | 0.001 | F1SV11 | 1023 |  |  |
|  |  | F1SJI1 | 17 | 10.24 | 0.000 | Q6S4N2 | 633 | 4.41 | 0.015 | F1SV22 | 2121 |  |  |
|  |  | F1SJR2 | 213 | 3.31 | 0.132 | Q7M2W6 | 19 | 2.75 | 0.131 | F1SV22 | 5536 |  |  |
|  |  | F1SJR2 | 134 | 6.98 | 0.061 | Q7M2W6 | 59 | 5.03 | 0.065 | F1SV26 | 95;92 |  |  |
|  |  | F1SJR2 | 1441 | 3.35 | 0.044 | Q7YS99 | 118 | 4.10 | 0.022 | F1SV46 | 548;498;501 |  |  |
|  |  | F1SJR2 | 533 | 4.95 | 0.018 | Q7YS99 | 121 | 6.48 | 0.014 | F2Z5G3 | 102 |  |  |
|  |  | F1SJR2 | 502 | 16.75 | 0.014 | Q95266 | 276 | 4.33 | 0.124 | F2Z5Q6 | 234;231 |  |  |
|  |  | F1SJR2 | 205 | 3.39 | 0.003 | Q95266 | 277 | 4.33 | 0.124 | G8ENL4 | 331;92 |  |  |
|  |  | F1SK12 | 561 | 2.35 | 0.015 | Q95266 | 334 | 2.47 | 0.055 | I3L5D4 | 23 |  |  |
|  |  | F1SK44 | 32 | 2.53 | 0.012 | Q99028 | 182;266 | 2.05 | 0.439 | I3L5T3 | 155 |  |  |
|  |  | F1SKP8 | 423 | 2.06 | 0.072 |  |  |  |  | I3L6C1 | 898 |  |  |

All proteins having ≥2-fold higher phosphorylation between groups (remote ischemic preconditioning (RIPC)/sham) at baseline and at eary reperfusion or between time points (baseline/early reperfusion) with RIPC or with sham and those detected exclusively in one group or timepoint in porcine left ventricular biopsies after phosphopeptide enrichment. When proteins were exclusively detected in one group or timepoint, -fold higher expression and p-value are not indicated, -fold higher expression and p-value versus sham. All proteins were compared by unpaired (between RIPC and sham) and paired (between baseline and early reperfusion) Student's t-tests.
